# Molecular mechanism of engineered *Zymomonas mobilis* to furfural and acetic acid stress

**DOI:** 10.1186/s12934-023-02095-1

**Published:** 2023-05-02

**Authors:** Samina Shabbir, Weiting Wang, Mohsin Nawaz, Prerona Boruah, Muhammad Fakhar-e-Alam Kulyar, Mao Chen, Bo Wu, Panting Liu, Yonghua Dai, Lingling Sun, Qiyu Gou, Renbin Liu, Guoquan Hu, Tahira Younis, Mingxiong He

**Affiliations:** 1 Graduate School of Chinese Academy of Agricultural Science, Beijing, 100081 People’s Republic of China; 2 Department of Chemistry, The Women University Multan, Multan, Pakistan; 3 Biomass Energy Technology Research Centre, Key Laboratory of Development and Application of Rural Renewable Energy (Ministry of Agriculture and Rural Affairs), Biogas Institute of Ministry of Agriculture and Rural Affairs, Section 4-13, Renmin Rd. South, Chengdu, 610041 People’s Republic of China; 4 Institute of Environment and Ecology, School of the Environment and Safety Engineering, Jiangsu University, Zhenjiang, 212013 China; 5 School of Biotechnology and Bioinformatics, DY PATIL Deemed to Be University, Navi Mumbai, India; 6 College of Veterinary Medicine, Huazhong Agricultural University, Wuhan, 430070 People’s Republic of China; 7 Department of Biochemistry and Biotechnology, The Women University Multan, Multan, Pakistan; 8 Chengdu National Agricultural Science and Technology Center, Chengdu, People’s Republic of China

**Keywords:** Acetic acid, Furfural, *Zymomonas mobilis*, Lignocellulosic biomass, RNA-Seq, Proteomics

## Abstract

Acetic acid and furfural (AF) are two major inhibitors of microorganisms during lignocellulosic ethanol production. In our previous study, we successfully engineered *Zymomonas mobilis 532* (ZM532) strain by genome shuffling, but the molecular mechanisms of tolerance to inhibitors were still unknown. Therefore, this study investigated the responses of ZM532 and its wild-type *Z. mobilis* (ZM4) to AF using multi-omics approaches (transcriptomics, genomics, and label free quantitative proteomics). Based on RNA-Seq data, two differentially expressed genes, ZMO_RS02740 (up-regulated) and ZMO_RS06525 (down-regulated) were knocked out and over-expressed through CRISPR-Cas technology to investigate their roles in AF tolerance. Overall, we identified 1865 and 14 novel DEGs in ZM532 and wild-type ZM4. In contrast, 1532 proteins were identified in ZM532 and wild-type ZM4. Among these, we found 96 important genes in ZM532 involving acid resistance mechanisms and survival rates against stressors. Furthermore, our knockout results demonstrated that growth activity and glucose consumption of mutant strains *ZM532∆ZMO_RS02740* and *ZM4∆ZMO_RS02740* decreased with increased fermentation time from 42 to 55 h and ethanol production up to 58% in ZM532 than that in *ZM532∆ZMO_RS02740*. Hence, these findings suggest *ZMO_RS02740* as a protective strategy for ZM ethanol production under stressful conditions.

**Supplementary Information:**

The online version contains supplementary material available at 10.1186/s12934-023-02095-1.

## Introduction

The rising global population and climate change have been described as major threats to life on our planet. According to an estimation, the population will reach 10 billion by 2030, which would urge climate change to a dangerous extent, mainly associated with human activities, e.g., burning fossil fuels (coal, oil, and gas) [1]. The desire for the world to curb human-induced climate change and ensure sustainable energy through environment friendly resources has gained much attention in the last decade [2]. Plant feedstocks fermentation is alternative to fossil fuel for renewable and sustainable energy. It can mitigate climate change and enhance energy security [3]. Substrate utilization and production capacity of microorganisms are critical to the production process of bioethanol. The biosynthesis of lignocellulosic biomass primarily through yeast strains is mainly industrial biocatalysts due to its economic, social sustainability, and environmental benefits [4]. Interestingly, engineered *Escherichia coli* (*E. coli*), *Zymomonas mobilis* (*Z. mobilis*), and *Bacillus subtilis* have successfully been deployed for industrial biofuel catalysts [5]. The best-known alcohol fermenting microbes are *Saccharomyces cerevisiae* and *Z. mobilis,* which can ferment hexose sugars and sucrose into ethanol but are inhibited by end products [6, 7]. End products also inhibit pentose-fermenting species such as *Pichia stipitis*, *Candida shehatae*, and *Pachysolen tannophilus* [8]. Despite their ability to withstand inhibitory compounds, filamentous fungi are unsuitable candidates for biofuel development due to their long generation times, low yields, and productivities [9]. As a result, a microorganism that is inhibited by end products and takes longer to hydrolyze lignocellulosic biomass is not suitable for industrial-scale biofuel production [10]. To use significant quantities of substrates, the optimal strain must possess certain characteristics, including the ability to achieve high cell mass growth and biofuel production rates in biomass-derived hydrolysates [6, 11], the ability to use a wide variety of pentose and hexose sugars, the ability to withstand high temperatures and low pH [12], and strong tolerance to inhibitors and end products.

*Zymomonas mobilis* is famous for higher ethanol efficacy and energy-generating potential at the industrial level. Its 1 mol of ATP is produced by a glucose molecule using the Enter-Doudoroff (ED) pathway [13]. However, various inhibitors are naturally produced during the hydrolysis process that inhibits cell growth and efficiency of microbial fermentation, such as furfural, vanillin, acetic acid, 5-hydroxymethyl furfural, aldehydes, phenols, and other organic acids [14, 15]. These inhibitors are detrimental to *Z. mobilis* growth and ethanol fermentation [16]. Acetic acid and furfural (AF) are major inhibitors that damage the stability of the membrane and intracellular homeostasis, resulting in lower pH, osmotic stress, and reduced carbohydrate metabolism [17]. So, to avoid lower production capacity and yield reduction, industries extricate inhibitors chemically from the lignocellulosic biomass, but such process often enhances the production cost. In regards, genome-resequencing analysis has also been used to explore the key genetic variations that are responsive to modified phenotypes in robust *Z. mobilis* mutants induced by mutations or adaptation [18–20].

Transcriptomics, proteomics, and metabolomics are examples of omics technologies used in reverse genetics methods that can enhance our understanding of biological systems and have become the latest trend in molecular research [21]. Hence, the technological revolution can assist us in evaluating how microorganisms react to various environmental stresses and improving strategies to enhance or change their genotype to perform efficiently in the presence of inhibitors. Many genetic approaches, including forward and reverse genetics, have been applied to develop inhibitor-resistant *Z. mobilis* strains [22, 23]. Moreover, several genes have also been cloned to study their involvement in ethanol production by the mean of their expression level. For example, *hfq* (*ZMO0347*) encoding RNA chaperone, and *nhaA* (*ZMO0119*) encoding sodium proton antiporter protein are used for enhancing the ability of *Z. mobilis* (*AcR*) to produce sodium acetate [24, 25]. Similarly, overexpression of *ZMO1696*, *ZMO1116*, and *ZMO1885* is used against phenolic aldehyde inhibitors in ZM4 [26]. The recently developed acid tolerant mutant strains have single nucleotide variants (SNVs) in glutamine-fructose-6-phosphate aminotransferase (encoding ZMO0056) and DNA repair proteins. A strain known as RadA (encoding *ZMO0589*) contributes to acid tolerance in mutant strains [27]. According to some studies, adaptive evolution and forward genetics may develop mutants that overcome inhibition caused by furfural, acetic acid, and other inhibitors in corn stover hydrolysate [22, 28, 29]. Also, several studies have reported the role of key genes/transcriptional factors in improving furfural resistance [30–36].

In our previous studies, we developed a resistant strain, i.e., F34, that was found to be tolerant to 3.0 g/L furfural, AQ8-1, and 8.0 g/L acetic acid by mARTP mutagenesis in *Z. mobilis* [27]. Also, a mutant strain, i.e., ZM532 (derived from genome shuffling), had higher productivity (0.463 g/L/h) and shorter fermentation (30 h) than AQ8-1 and F34 [16]. But we had not explored the difference in global transcriptional profiling between mutant ZM532 and wild type ZM4, especially in rich media (RM) and media containing acetic acid and furfural, which is potentially important at the industrial level. Therefore, in the current study, we first used sanger sequencing technology to verify the mutations in strains. Afterward, we applied transcriptomics and proteomics to unravel molecular mechanisms under AF and RM conditions. Further, we knocked out and overexpressed differentially expressed genes (DEGs) to study their modulating role. Hence, our findings in ethanol production might play an important role in genetic engineering and synthetic biology.

## Material and methods

### Bacterial strains and fermentation conditions and preparation of cell samples for transcriptome and proteome

This study used *Z. mobilis* strains (ZM4 and its mutant ZM532). The glycerol stocks of ZM4 and ZM532 were grown at 30 °C and maintained on two agar rich medium (RM) containing plates (20 g/L glucose, 10 g/L yeast extract, 2 g/L KH_2_PO_4_, 1 g/L (NH_4_)_2_SO_4_, 2 g/L MgSO_4_·7H_2_O, and 15 g/L agar) until colonies were grown and stored at 4 °C. Then, both strains were cultured in RM at 30 °C without shaking for 16 h. Then, a single colony from both strains was sub-cultured to fresh inoculum 50 mL RM media (20 g/L glucose, 10 g/L yeast extract, 2 g/L KH_2_PO_4_, 1 g/L (NH_4_)_2_SO_4_, and 2 g/L MgSO_4_·7H_2_O) for 16 h at 30 °C without shaking (Inoculation into fermentation medium was conducted when the initial cell density of optical density 600 (OD_600_) for ZM4 and ZM532 were between 0.1 and 0.2). Cell pellets were extracted by centrifugation at 3000×*g* for 4 min at 4 °C and then inoculated 50.0 mL of RM in a 100 mL flask in groups without inhibitors for 8 h and with inhibitors (AF) for 36 h fermentation period without shaking at 30 °C. The groups of ZM4 and ZM532 susceptible to acetic and furfural acids combination were named as AFZM4 (AFZM4_1, AFZM4_2, and AFZM4_3), and AF532 (AF532_1, AF532_2, and AF532_3), respectively, while other groups without inhibitors, the cells grown in RM were considered as control groups designated as RMZM4 (RMZM4_1, RMZM4_2, and RMZM4_3) and RM532 (RM532_1, RM532_2, and RM532_3). Both fermentations and culturing were performed in triplicates. Based on the previous experiment, the concentrations of AF combinations were set at 5.0 g/L (acetic acid) and 3.0 g/L (furfural) to study the responses of ZM4 and mutant ZM532 [16]. The cells at the exponential growth phase of ZM532 and ZM4 were collected and stored at − 80 °C. The collected cell pellets were used for subsequent transcriptome, proteome, and qPCR experiments.

### RNA extraction, library preparation, and sequencing

Total RNAs were extracted using the RNA isolation kit from the ZM4 and ZM532 cells cultured in RM and AF medium. (Tiangen, China). The RNA purity, concentration (ng/ul), and integrity were evaluated using the Nanodrop spectrophotometer (Qubit 2.0, Agilent 2100). Briefly, the mRNA was purified from a total amount of 3 μg RNA per sample using poly-T oligo-attached magnetic beads and fragmented using divalent cations under elevated temperature. Random hexamer primer and M-MuLV reverse transcriptase were used for cDNA first-strand synthesis [23, 37]. Subsequently, second-strand cDNA synthesis was performed using DNA polymerase I and RNase H. After adenylation of 3′ ends of cDNA fragments, NEB Next Adaptor with hairpin loop structure were ligated to prepare for hybridization. Then, the cDNA fragments were purified with AMPure XP system (Beckman Coulter, Beverly, CA, USA) to select fragments of 150–200 bp in length. PCR was then performed, products were purified (AMPure XP system), and library quality was assessed on the Agilent Bioanalyzer 2100 system. The sample clustering was performed on a cBot Cluster Generation System using TruSeq PE Cluster Kit v3-cBot- HS (Illumina, San Diego, CA, USA) according to the manufacturer’s instructions. The high-throughput sequencing was conducted by Illumina Hiseq 2000 platform after passing through some screening phases. The transcript sequences of *Z. mobilis* used for the study have been deposited in the Gene Expression Omnibus (GEO) repository of the National Center for Biotechnology Information (NCBI) GEO accession number: GSE168900.

### Reads mapping to the reference genome and quantification of gene expression

Raw data (raw reads) were interpreted via in-house perl scripts, and clean data was extracted by eliminating reads comprising adapters. Then, the clean data of Q20, Q30, and guanine-cytosine (GC) content were computed. Complete genome annotation files downloaded from the genome website Bowtie2-2.2.3 (ftp://ftp.ncbi.nlm.nih.gov/genomes/bacteria/Zymomonas_mobilis/) were used to construct a reference genome index and match clean reads to the reference genome [38]. Novel genes, operons, and transcription start sites were identified by Rockhopper [34]. Then, extracted the 5′UTR (3′UTR) sequences. Then, RBS finder [39] and TransTermHP [40] were used to predict SD sequences and terminator sequences, respectively. IntaRNA was used to predict the sRNA targets. And then, we used RNAfold to predict RNA secondary structures [41, 42]. The mapping of clean reads to each gene was counted using HTSeq v0.6.1. The fragments per kilobase of exon per million fragments mapped (FPKM) reads of every single gene were determined as described earlier [43].

### Differentially expression genes analyses

The edger software package modified the read counts for each sequenced library via one standardized scaling factor. DEGs analyses of two conditions were performed using the DESeq package in R (1.18.0) [44]. Then using Benjamini & Hochberg approach, the* p*-values were adjusted. Genes with fold change (FC) > 1.5 and a false discovery rate (FDR; < 0.05) were considered DEGs.

### Validation of differentially expressed genes by quantitative PCR

The RNA from the AF and RM groups was extracted and used to construct the cDNA library. First-strand synthesis was performed using MonScript (Monad) according to the manufacturer’s instructions. With three biological replicates to perform the expression of ZM4-AF and ZM532-AF (resistance group) and ZM4-RM and ZM532-RM (control group) by qPCR (BIO-RAD, Richmond, CA, USA). The reaction phase was denaturation for 15 min at 95 °C, followed by 40 amplification cycles for 10 s at 95 °C and 30 s at 53 °C. Using the delta-delta- Ct (${2}^{-\Delta \Delta Ct}$) method with 16S RNA as a reference control, relative gene expressions were computed **(**Additional file 1: Table S2). The student t-test (*p* < 0.05) was used for mean comparisons. Results were shown in a bar chart with the means and their standard deviation (M ± SD).

### Total protein extraction and protein quality test

ZM4 and ZM532 samples (cell pallets)were independently ground in liquid nitrogen and lysed with a lysis buffer (consisting of 6 M Urea and 0.2% SDS, 100 mM NH_4_HCO_3_, pH 8.0), accompanied by 5 min of ice ultrasound. The lysate was centrifuged at 12,000*g* for 15 min at 4 °C, and the supernatants were transmitted to a clean tube. The extracts from each sample were reduced to 10 mM DTT for 1 h at 56 °C and alkylated with iodoacetamide under dark room temperature for 1 h. Samples were thoroughly vortexed with 4 × the volume of precooled acetone and incubated at − 20 °C for at least 2 h. Samples were then centrifuged and precipitated. They were washed twice with cold acetone, and pellets were dissolved with a dissolution buffer of 0.1 M triethylammonium bicarbonate (TEAB, pH 8.5) and 6 M urea [45–47].

### Protein quality test

BSA standard protein solution was prepared according to the Bradford protein quantitative kit's instructions, with gradient concentration ranging from 0 to 0.5 g/L. BSA standard protein solutions and sample solutions with different dilution multiples were added to a 96-well plate to fill up the volume to 20 µL. Each gradient was repeated three times. The plate was added 180 μL G250 dye solution quickly and placed at room temperature for 5 min. The absorbance at 595 nm was detected. The standard curve was drawn with the absorbance of the standard protein solution, and the protein concentration of the sample was calculated. 20 μg of the protein sample was loaded to 12% SDS-PAGE gel electrophoresis, wherein the concentrated gel was performed at 80 V for 20 min, and the separation gel was performed at 120 V for 90 min. The gel was stained by coomassie brilliant blue R-250 and decolored until the bands were visualized clearly.

### Trypsin treatment

120 μg of each protein sample was taken, and the volume was made up to 100 μL with lysis buffer, 3 μL of 1 μg/μL trypsin, and 500 μL of 100 mM TEAB buffer was added; the sample was mixed and digested at 37 °C overnight. An equal volume of 1% formic acid was mixed with the digested sample and centrifuged at 12,000*g* for 5 min at room temperature. The supernatant was slowly loaded to the C18 desalting column, washed with 1 mL of washing solution (0.1% formic acid, 4% acetonitrile) 3 times, then eluted twice by 0.4 mL of elution buffer (0.1% formic acid, 75% acetonitrile). The eluents were combined and lyophilized.

### LC–MS/MS analysis

Mobile phase A (100% water, 0.1% formic acid) and B solution (80% acetonitrile, 0.1% formic acid) were prepared. The lyophilized powder was dissolved in 10 μL of solution A, centrifuged at 15,000 rpm for 20 min at 4 °C, and 1 μg of the supernatant was injected into a home-made C18 Nano-Trap column (2 cm × 75 μm, 3 μm). Peptides were separated in a home-made analytical column (15 cm × 150 μm, 1.9 μm), using a linear gradient elution as listed in Table 1. The separated peptides were analyzed by Q Exactive series mass spectrometer (Thermo Fisher), with ion source of Nano spray Flex™ (ESI), spray voltage of 2.3 kV and ion transport capillary temperature of 320 °C. Full scan range from m/z350to 1500 with a resolution of 60,000 (at m/z200), an automatic gain control (AGC) target value was 3 × 10^6^ and a maximum ion injection time was 20 ms. The top20 (40) precursors of the highest abundant in the full scan were selected and fragmented by higher energy collisional dissociation (HCD) and analyzed in MS/MS, where a resolution was 15,000 (at m/z200), the automatic gain control (AGC) target value was 5 × 10^4^, the maximum ion injection period was 45 min, the intensity threshold was 2.2 × 10^4^, the normalised collision energy was 27 percent, and the dynamic exclusion parameter was 20 s.

**Table 1:** SNP in re-sequence ZM532 by comparing previous published ten genome-shuffled mutant strain and* Z. mobilis* ZM4 (GenBank: AE008692.2)

Locus	Ref^a^	Previous^b^	Current^c^	Status	Ten genome shuffled^d^	Gene/Product
*CDS*						
51,967	C	T	T	Confirmed	+	ZMO_RS00235/glutamine-fructose-6-phosphate aminotransferase
590,452	G	A	A	Confirmed	+	ZMO_RS02620/DNA repair protein Rad A
849,208	C	T	T	Confirmed	+	ZMO_RS03765/arginine-tRNA ligase
849,311	C	A	A	Confirmed	+	
971,308	A	G	–	No SNP found	−	ZMO_RS09165/1S5/1S1182 family transposase
971,369	A	G	–	No SNP found	−	
*Intergenic regions*						
971,059	T	A	A	Confirmed	+	ZMO_RS09160–ZMO_RS09165
971,715	C	–	T	New SNP	+	
971,717	T	–	G	New SNP	+	
975,503	T	G	G	Confirmed	+	ZMO_RS04290–ZMO_RS04295
975,506	G	A	A	Confirmed	+	Monofunctional biosynthetic peptidoglycan
975,509	C	T	T	Confirmed	+	Transglycosylase/cytochrome c
975,523	C	T	T	Confirmed	+	
975,525	A	T	T	Confirmed	+	
975,528	T	G	G	Confirmed	+	
975,532	A	T	T	Confirmed	+	
975,537	A	C	C	Confirmed	+	
975,540	G	T	T	Confirmed	+	
975,544	A	–	G	New SNP	+	
975,545	G	–	A	New SNP	+	
975,547	T	G	G	Confirmed	+	
975,899	T		C	New SNP	+	ZMO_RS04295
1,612,575	G	A	–	No SNP found	−	ZMO_RS07065–ZMO_RS07070
1,612,744	G	–	A	New SNP	+	Alpha/betahydrolase/Trna-Met
2,055,763	T	C	C	Confirmed	+	ZMO_RS09095-END
2,055,333	G	–	A	New SNP	+	Uroporphyrinogen decarboxylase/END

^a^Reference genome ZM4

^b^Wang et al. [16]

^c^Current study with ZM532 strain

^d^ ± indicate the presence/absence of variation in the genome, respectively

The raw data of MS detection was named as “Raw”.

### Label-free quantitative protein analysis

The result of each fraction was searched separately by the search engines for *Z.-mobilis*-NCBI databases: Proteome Discoverer 2.2. (PD 2.2, Thermo). The search parameters were set as follows: the tolerance of precursor ion mass was 10 ppm, and the tolerance of product ion mass was 0.02 Da. Carbamidomethyl was mentioned in PD 2.2 as a fixed amendment. The oxidation of methionine (M) and acetylation of N-terminus were identified in PD 2.2 as variable modifications. A maximum of 2 missing cleavage sites were allowed. At least 1 distinct peptide with no more than 1.0% false discovery rate (FDR) contains the protein identified. Related peptides that could not be identified by an MS/MS analysis were categorized in the same category of proteins. Based on the intensity used for label-free quantification, precursor ions were quantified using a label-free method. The Mann–Whitney Test for proteins whose quantitation differs significantly between experimental and control groups (*p* < 0.05 and log2FC > 1.5) was evaluated as the differentially expressed proteins. GO analysis was performed using an interproscan-5 program against a non-redundant protein database (such as Pfam, PRINTS, ProDom, SMART, ProSiteProfiles, and PANTHER) [48]. Criteria for analysis of GO and Kyoto Encyclopedia of Genes and Genomes (KEGG) were followed as illustrated by [49]. All sequencing phases were performed by Novogene Sequencing Company (Chengdu, China). The mass spectrometry proteomics data have been deposited to the ProteomeXchange Consortium via the PRIDE [50] partner repository with the dataset identifier PXD030417.

### Genomic DNA isolation and re-confirmation of previously identified 19 SNPs in mutant strain ZM532

ZM532 and ZM4, 5 mL of cells were harvested from overnight culture by centrifugation at 13,500 rpm for genomic DNA (gDNAs) extraction via Bacterial DNA Kit (Omega, Bio-tek, USA). The quality and concentration of gDNAs were estimated by Qubit 3 Fluorometer and gel electrophoresis (0.25% agarose, 120 V/cm, 40 min), respectively. To ensure that the ZM532 is associated with *Z. mobilis*, ZM532 genes were amplified by PCR. From 5.0 μL overnight culture, fresh cells were harvested, washed, and re-suspended in 10 μL of ddH_2_O. PCR conditions and reactions were set and performed following the manufacturer’s instructions with minor modifications (Toyobo, Japan) with primers (Additional file 1: Table S1). Amplicons were sent to GENEWIZ Inc. (Chengdu, China) for sequencing. After sequencing, BioEdit 7.0 software [51] was used to analyze the data against the reference genome of strain ZM4 (NC_006526.2) to identify single nucleotide polymorphism (SNP)/insertion-deletion (indel) in ZM532.

### Construction of plasmids, strains, and culture conditions

ZMO_RS02740 and ZMO_RS06525 in RNAseq were selected for verification through Type I-F clustered regularly interspaced short palindromic repeats (CRISPR)-CRISPR associated protein (Cas) (CRISPR–Cas) system technology following recommended procedure (Additional file 1: Fig. S1) [52], encoding chemotaxis protein Mot A and major facilitator superfamily (MFS) proteins) in stress tolerance by knock out. pEZ15Asp was used as a backbone vector. sgRNA fragments were ligated with a linear vector pEZ15Asp linearized through Gibson assembly method, yielding plasmids Pmini-T-ZMO-RS02740, carrying an artificial mini-CRISPR array. Donor DNA fragments containing up stream (ZMO-RS02740, 500 bp) gfp marker and its promoter pdc (1020 bp) downstream (ZMO-RS02740, 500 bp) regions were obtained by overlap extension PCR amplification using primers. The PCR products were linked with Pmini-T-ZMO-RS02740 vector through Gibson assembly after generating the genome editing plasmid, Pmini-T-ZMO-RS02740. The correct plasmids were electroporated into ZM4 and ZM532 competent cells by using the previously described method [53]. Transformants were cultured on RM agar plates with spectinomycin (100 μg/mL). After 4–5 days of incubation at 30 °C, positive clones were detected by colony PCR with check primer and DNA sequencing (Tsingke, Chengdu, China). Similarly, the Pmini-T-ZMO-RS06525 plasmid was constructed using the same approach (Additional file 1: Table S3). All DNA manipulation, such as the transformation of *E. coli*, plasmid preparation from *E. coli*, ligation, digestion of restriction enzyme, and agarose gel electrophoresis, were conducted according to standard protocols [54]. Cell growth, ethanol production, and glucose consumption by recombinant strains were calculated under furfural (3.0 g/L), and acetic acid (5.0 g/L) stress conditions.

### Analytical methods

Concentrations of ethanol production and glucose consumption were determined using the High-performance liquid chromatography (HPLC, Agilent 1200) with column (HPX-87H), while UV Spectrophotometer was used to estimate the cell density at OD600. Fresh cultures were incubated at 30 ͦ. At specific periods, 1uL of culture was harvested by centrifugation at 4500×*g* for 2 min, and the extracts were collected and diluted 10 times. HPLC (Agilent 1200) was used to estimate ethanol production and glucose consumption at 0.6 mL/min flow rate with 5 mM H_2_SO_4_, and 35 °C column temperature with 20.0 μL volume of injection, respectively. The following formulas were used to calculate ethanol productivity and yield.

1 $$\text{Ethanol productivity}=\text{Ethanol titer}/\text{fermentation time}.$$

2 $$\text{Ethanol yield}=\text{Ethanol titer}/\text{glucose consumed}.$$

The theoretical ethanol yield is 0.51 g/g of sugars consumed.

### Evaluation of candidate operons under AF conditions

We selected ZMO-RS02740 and ZMO-RS06525 amplified from mutant strain ZM532 and ZM4 gDNAs using specific primers (Additional file 1: Table S4). With *Ptet* promoter, PCR products were cloned into shutter vector pEZ15Asp [55] via Gibson assembly process [56] using overlapping primers consisting of 18–20 nucleotides. With right plasmid constructions, recombinant strains were detected by colony PCR with primer checks and confirmed by Sanger sequencing (Tsingke, Chengdu, China). These control plasmids were pEZ15Asp-ZM402740, pEZ15Asp-ZM53202740, pEZ15Asp-ZM406525, and pEZ15Asp-ZM53206525, respectively. While treatment plasmids were named ZM4-02740, ZM532-02740, ZM4-06525, and ZM532-06525, respectively. The right plasmids were then transformed into competent ZM532∆ZMO_RS02740, ZM4∆ZMO_RS02740, ZM532∆ZMO_RS06525, and ZM4∆ZMO_RS06525 mutant cells via electroporation using previously described method [53]. After getting mutants, cell growth was calculated under furfural (3.0 g/L), and acetic acid (5.0 g/L) stress conditions.

### Statistical analysis

According to student t-test statistics, the data was significant as the value obtained was *p* < 0.05, and the data expression is mean ± SD (Mean ± SD).

## Results

### Re-sequencing of previously identified 19 mutations for confirmation

We identified 23 single nucleotide polymorphisms (SNPs) (4 CDS and 19 within the intergenic region) via Sanger sequencing. Six SNPs are novel in this study (Table 1). Wang et al. [16] identified 19 identical SNPs in wild type ZM4 in the CDS (6) and 13 in the intergenic regions, respectively (Table 1).

The four SNPs in the CDS regions caused amino acid (AA) variation, resulting in synonymous and non-synonymous mutations (Table 1). In ZMO_RS03765 (arginine-tRNA ligase), one non-synonymous and synonymous AA change was identified at the same time. In contrast, in ZMO_RS00235 and ZMO_RS02620, two non-synonymous AA changes were detected, which are linked to glutamine-fructose-6-phosphate aminotransferase and DNA repair protein RadA, respectively (Table 1). As synonymous (silent) mutations are largely invisible to natural selection [57], while nonsynonymous (amino-acid-replacing) mutations may be under strong selective pressure, comparison of the rates of fixation of those two types of mutations provides a powerful tool for understanding the mechanisms of DNA sequence evolution. For example, variable nonsynonymous/synonymous rate ratios among lineages may indicate adaptive evolution [58] or relaxed selective constraints along certain lineages [59]. Likewise, models of variable nonsynonymous/synonymous rate ratios among sites may provide important insights into functional constraints at different amino acid sites and may be used to detect sites under positive selection [60]. Moreover, if SNPs change either the function of a gene or its expression, and the change provides greater fitness for a population (i.e., a higher capacity to survive and/or reproduce in a given environment), the change will be favored by natural selection [61]. Sometimes nonsynonymous mutations are actually positive changes. Natural selection may favor this new expression of the gene, and the individual may have developed a favorable adaptation from the mutation. While the gene ZMO_RS02620 encodes a DNA repair protein called RadA, which is necessary for cellular survival when cells are exposed to acid stress [62]. Jeong et al. [63] reported that under acid stress in *E. coli* O157: H7 strand disintegrates and DNA integrity was retained by Dps and RecA-mediated repairs, indicating that DNA repair can play an important role in acid tolerance. Wang et al. [16] identified two non-synonymous AA changes in ZMO_RS09165 (IS5/IS1128 family transposase), but these were absent in the present study. Conversely, we found SNPs in the intergenic regions of the pairs of genes: ZMO_RS09160 and ZMO_RS09165; ZMO_RS04290 and ZMO_RS04295; and ZMO_RS07065 and ZMO_RS07070 in ZM532 mutant. There was also a frame shift mutation in ZMO_RS04405, which codes for ABC transporter substrate-binding protein because of two single nucleotide deletions in the CDS region (Additional file 1: Table S5). According to Ask et al. [64], ABC transporters PDR5 and YOR1 operate in the efflux of ions and are activated by xenobiotics under the transcriptional control of Pdr1p and Pdr3p in *S. cerevisiae*, and can likely function in transporting furfural out of the cell, thus removing the stress caused by this agent. We also detected a 21 bp deletion in the ZMO_RS05590 (hypothetical protein-coding gene), while the previous studies identified two distinct deletions (21 bp and 28 bp) in different locations of the same gene. Additionally, there was a7 bp deletion in ZMO_RS07255 (carbamoyl phosphate synthase large subunit) and 1 bp InDel in the intergenic region of ZMO_RS06410-ZMO_RS06415 (Additional file 1: Table S5). In short, genes involved in the same mutations on the parental and mutant strains have been reported previously and may play a critical role against acids stressors.

### Overview of transcriptome under inhibitors (AF)

The RNA-seq yielded a total of 32.79 Gb clean data, averaging 2.73 Gb for each sample with 91% of bases recording Q30 and above, with a q ≥ 20 (an error probability of 0.03%) (Additional file 1: Table S6). The GC-contents in the four distinct groups ranged from 48.26–49.15. A gene was considered DEG after comparing the gene expression profiles between RM and AF treatments with FC > 1.5 and FDR corrected *p* < 0.05. A total of 1865 and 14 novel DEGs were identified.

### Differentially expressed genes in ZM532 and ZM4 and their expression profiles

**Fig. 1:**
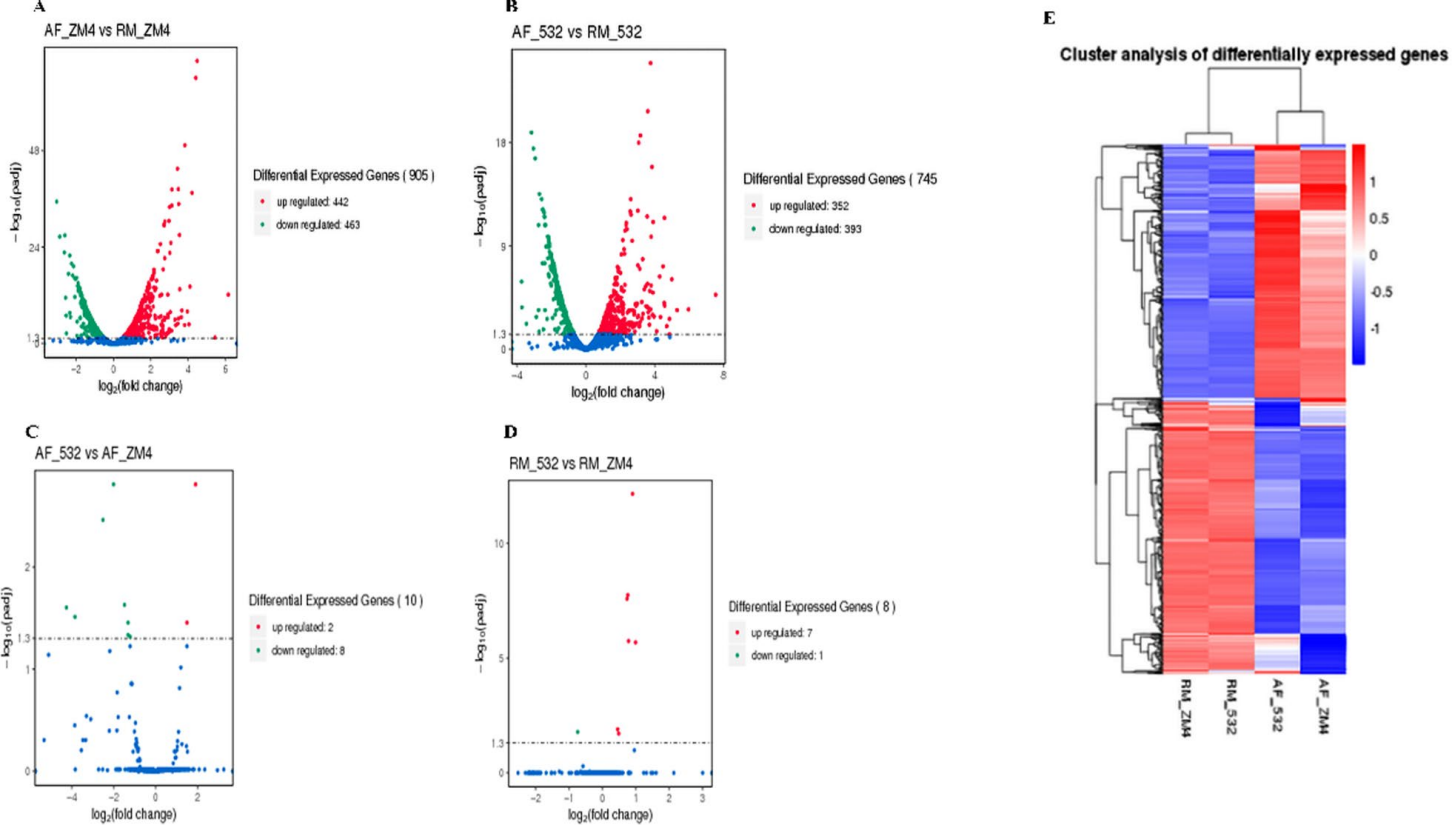
**A** Identification of the differentially expressed genes (DEG). Volcano plot depicting the up and down-regulated genes between RM and AF in **A** AF_ZM532_vs_RM_532, **B** AF_ZM4_vs_RM_ZM4, **C** AF_ZM532_vs_AF_ZM4, **D** RM_ZM532_vs_RM_ZM and **E** Heatmap clustering analysis of the samples based on log2 fold change obtained from FPKM data of the DEGs

We identified 745 and 905 DEGs in ZM532 and ZM4, respectively (Fig. 1A). Among these, 352 DEGs were up-regulated; while 393 were down-regulated in AF_ZM532_vs_RM_532, respectively. However, 442 DEGs were up-regulated, and 463 were down-regulated in AF_ZM4_vs_RM_ZM4 (Fig. 1A, B). In addition, 2 up and 8 down-regulated genes in AF_ZM532_vs_AF_ZM4; while 7 up and 1 down-regulated gene in RM_ZM532_vs_RM_ZM **(**Fig. 1C, D). The higher number of DEGs detected in the strain ZM4 suggests an intense transcriptional alteration in response to the inhibitors due to ZM4 relative sensitivity. We performed hierarchical cluster analysis based on the log2FC and FPKM values to validate the DEGs from ZM4 and ZM532 strains (Fig. 1E). The analysis clustered the DEGs into two main groups with the two strains clustering together regardless of the treatments. This implies that very few DEGs were distinguishable between these two strains in their response to AF treatments.

Subsequently, we searched for candidate genes involved in the tolerance mechanism against the inhibitors by comparing the DEGs between the two strains. We detected 98 DEGs exclusively involved in the AF resistance in ZM532, including 42 up-regulated DEGs in response to the inhibitors and associated with oxido-reductase activity (Additional file 1: Fig. S2A). Additional 647 DEGs were mutually detected in both samples. While in AF_ZM532_vs_AF_ZM4 (purple) and RM_ZM532_vs_RM_ZM4, only 1 DEGs was co-detected in both strains. We identified 7 DEGs to RM_ZM532_vs_RM_ZM4 and 9 to AF_ZM532_vs_AF_ZM4 (Additional file 1: Fig. S2B). The most up- and down-regulated DEGs were ZMO_RS02740 (log_2_FC = 6.05) and ZMO_RS06525 (log_2_FC = − 2.373) in the ZM532 strain. These candidate DEGs represent important resources for further functional validation in AF resistance in *Z. mobilis*.

### Label free data and functional annotation of the proteins under AF

The proteomes of the two *Z. mobilis* strains (ZM4 and its mutant ZM532) were generated under control and treatment conditions to elucidate the molecular response and tolerance to AF inhibitors. We successfully identified 1532 proteins in both samples (Table 2).

**Table 2:** Statistics on the Label free data

Total spectra	Matched spectrum	Peptides	Identified proteins
1,647,059	364,149	23,673	1532

**Fig. 2:**
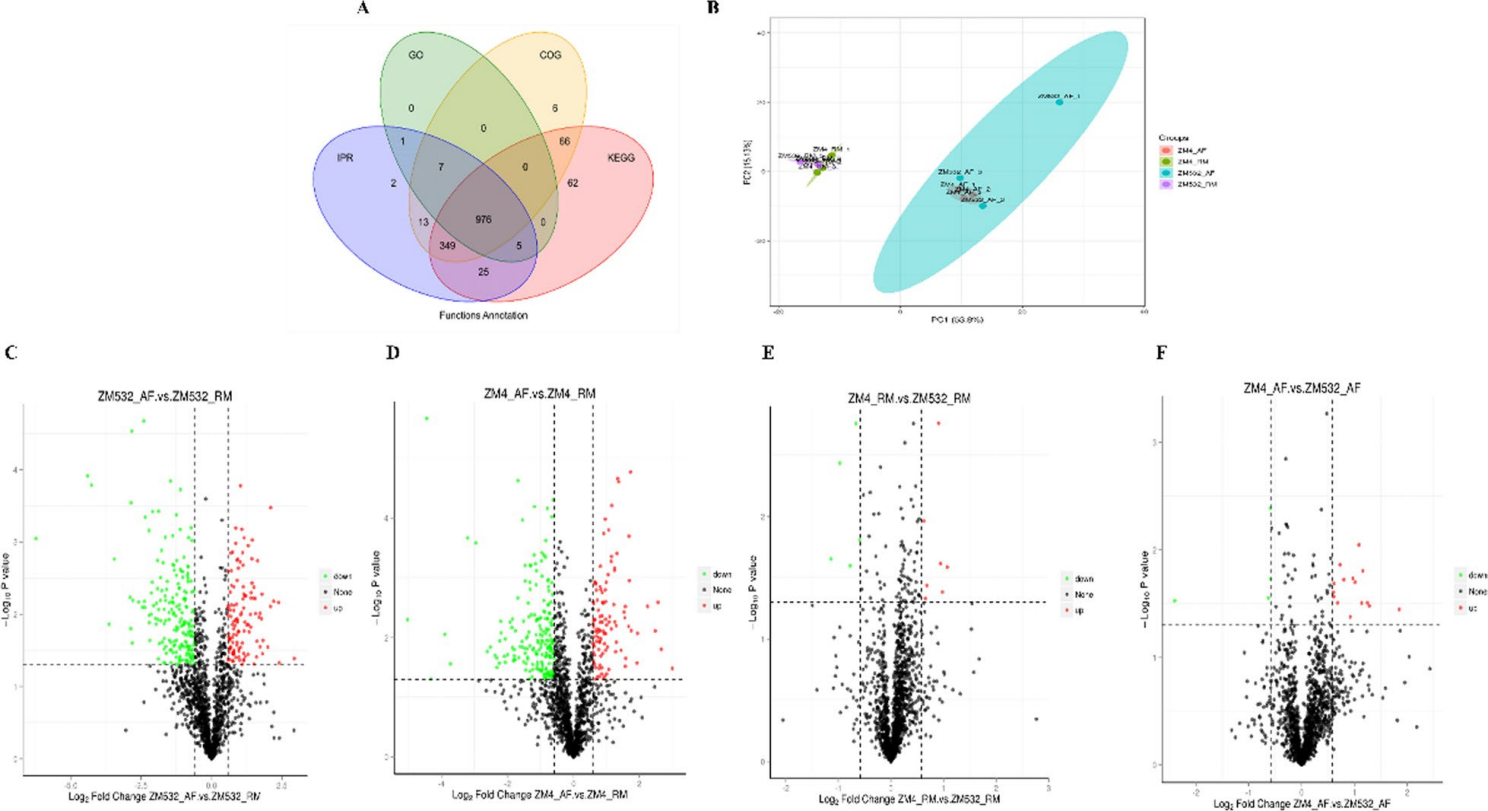
**A** Venn diagram depicting the shared and specific proteins functionally annotated in various databases; Identification of the DEPs; **B** PCA of the samples; Volcano plot depicting the up and down-regulated proteins between RM and AF in **C** ZM532_AF_vs_ZM532_RM; **D** ZM4_AF_vs_ZM4_RM; **E** ZM4_AF_vs_ZM532_AF; **F** ZM4_RM_vs_ZM532_RM

The mutant strain ZM532 was more resistant to the inhibitors than ZM4. The number of proteins detected in the AF samples was lower than in the RM samples (Additional file 1: Fig. S3A), suggesting that the treatment inhibited protein synthesis in Z. mobilis. Most of the identified proteins' mass distribution and protein length were between 10 and 60 kDa and 6–20 amino acids, respectively (Additional file 1: Fig. S3B-C). There was less variability in the majority of the detected proteins (CV < 0.2) (Additional file 1: Fig. S3D). The proteins were functionally annotated by BLASTP (E value ≤ 1e−4) using the Clusters of Orthologous Groups (COG), GO, KEGG, and Interpro (IPR) databases**.** We successfully annotated 99% of the total proteins in at least one database (Fig. 2A), and 976 proteins (76%) were annotated in all four databases. A protein's function is usually associated with its subcellular localization; the capability to predict subcellular localization directly from protein sequences benefits inferred protein functions. The statistical analysis of the proportion of subcellular location (Cell-mPLOC 2.0 website) of the differential protein is shown in Additional file 1: Fig. S4. The identified proteins demonstrated that 52.17% of proteins were found in resemblance with cytoplasmic proteins; 32.61% proteins significant hits for cell inner membrane proteins, followed by periplasm proteins (5.34%), extracellular proteins (2.57%), flagellum proteins (0.59%) and 0.20% proteins with nucleoid protein. Furthermore, principal component analysis (PCA) was performed based on the protein expression profile. The first two PCs accounted for more than half of the total variations, indicating that AF treatment significantly altered the proteome in both strains **(**Fig. 2B).

### Differentially expressed proteins in response to the inhibitors (AF)

The protein expression data were compared between ZM4 and ZM532 groups to detect the differentially expressed proteins (DEPs) based on the FC > 1.5 and *p* < 0.05. A total of 107 up and /204 down-regulated proteins out of 1477 were detected in ZM4_AF_vs_ZM4_RM; while 123 up-regulated and 205 down-regulated proteins out of 1474 were identified in ZM532_AF_vs_ZM532_RM, respectively (Fig. 2C, D). In addition, 16 up-regulated and 5 down-regulated proteins were identified out of 1462 in ZM4_AF_vs_ZM532_AF, while 8 up and 5 down-regulated proteins were observed out of 1491 in ZM4_RM_vs_ZM532_RM (Fig. 2E, F). Comparative analysis of the DEPs between ZM4 and ZM532 revealed 186 DEPs share the same pattern of the regulation (Additional file 1: Fig. S5). This suggests they represent the core proteome response to the inhibitors, regardless of the tolerance level of the *Z. mobilis* strains.

### Pathway enrichment analysis of the differentially expressed proteins

**Fig. 3:**
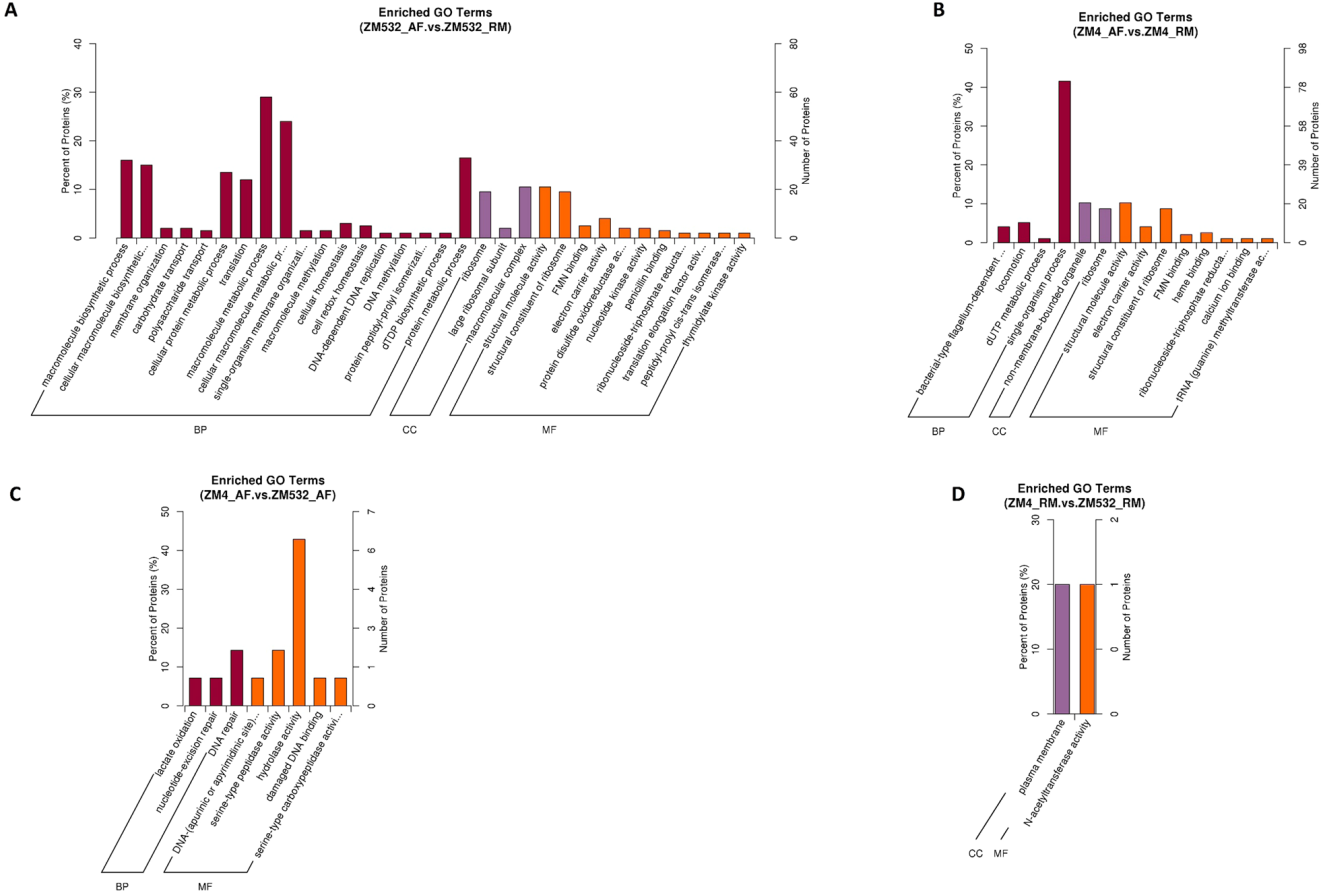
GO enrichment analysis of DEPs in GO enrichment of the DEPs with *p* < 0.05 in **A** ZM532_AF.vs.ZM532_RM; **B** ZM4_AF.vs.ZM4_RM; **C** ZM4_AF.vs.ZM532_AF; **D** ZM4_RM.vs.ZM532_RM

GO, and KEGG enrichment analyses were performed to understand the biological pathways activated in response to the inhibitors**.** For GO enrichment analysis, we mainly focused on molecular function and cellular component classes. In the two strains, DEPs related to ‘non-membrane-bounded organelle’, ‘large ribosomal subunit’, and ‘ribosome’ were the enriched terms in the GO cellular component class, indicating major alterations in the ribosome induced by the inhibitors. These proteins mainly contributed to ‘structural molecule activity, ‘electron carrier activity, and structural constituent of ribosome’ (molecular function terms), indicating that the inhibitors affect the structural integrity and normal ribosome activity (Fig. 3A, B). While in ZM4_AF_vs_ZM532_AF, DEPs mainly related to hydrolase activity, endonuclease activity, and damage DNA binding were more enriched molecular functions terms. Moreover, hydrolase activity is important against inhibitors, which involve several critical functions such as maturation, turnover, recycling, and autolysis. In addition, DNA damage binding activity has a direct relationship to decreased nucleotide excision repair, and endonucleases play a role in DNA repair in resistance strain ZM532 (Fig. 3C). But in the case of ZM4_RM_vs_ZM532_RM, most of DEPs linked with the plasma membrane at the cellular level, while at molecular level DEPs were involved in *N*-acetyletransferase activity (Fig. 3D).

A similar result was obtained concerning the KEGG enrichment analysis in the two strains, highlighting ‘ribosome’ as the most affected pathway (Additional file 1: Fig. S6A, B). In addition, flagellar assembly, peptidoglycan biosynthesis, and ribosome were enriched KEEG pathways in ZM4_AF_vs_ZM532_AF, while folate biosynthesis, an amino sugar, and nucleotide sugar metabolism and ABC transporter were the most enriched term in ZM4_RM_vs_ZM532_RM (Additional file 1: Fig. S6C, D).

The identified DEPs were mapped to the reference pathways in the KEGG database, and 21 different biological pathways were obtained in 4 major categories (Additional file 1: Fig. S6E). The KEGG pathways (which include 914 proteins) were members of a major group, metabolism, 153 were linked to genetic information processing, 59 were related to cellular processes, and 87 were related to environmental information (Additional file 1: Fig. S7). KEGG enrichment analysis in the two strains also affirms ‘ribosome’ as the most affected pathway in both strains.

Analysis of expression fold changes of the proteins involved in selected pathways revealed that the resistant strain (ZM532) strongly delayed the activity in the ribosome by reducing the synthesis of all ribosome-assembly proteins under AF treatment (Table 3), a mechanism known as hypometabolism [65]. In contrast, several ribosome-assembly proteins were either up-regulated or unaffected following AF treatment in ZM4 (Table 3). We speculate that the ability to limit ribosome activity is an effective adaptation mechanism against AF.

**Table 3:**
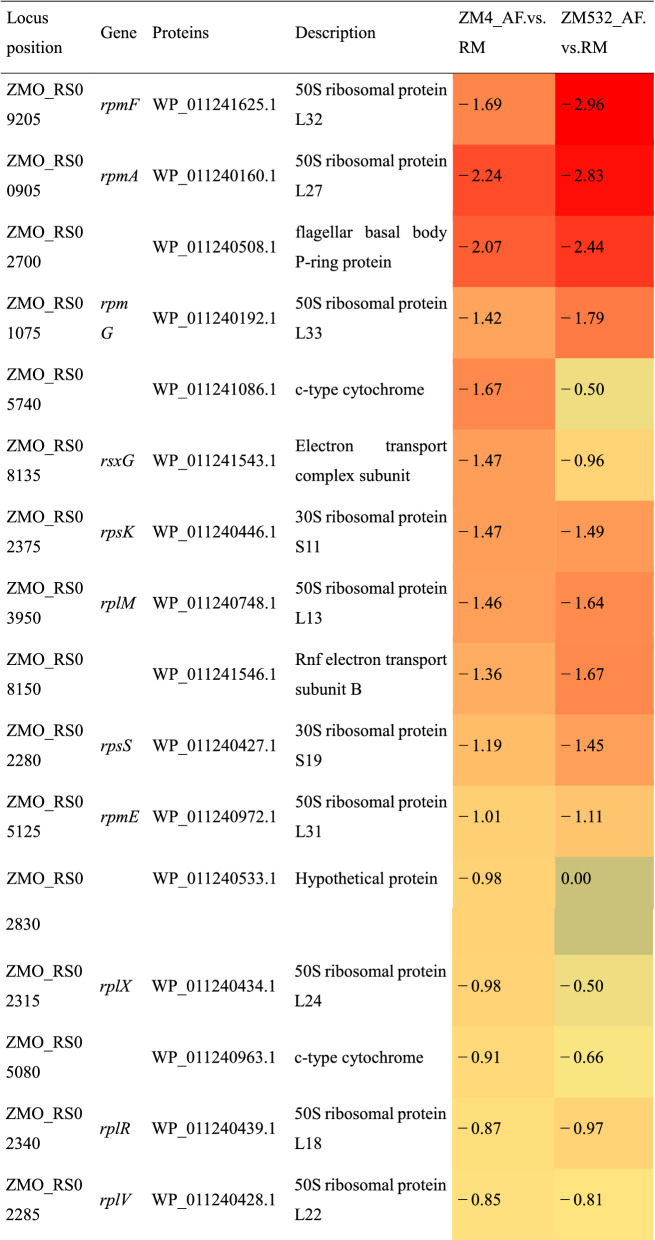

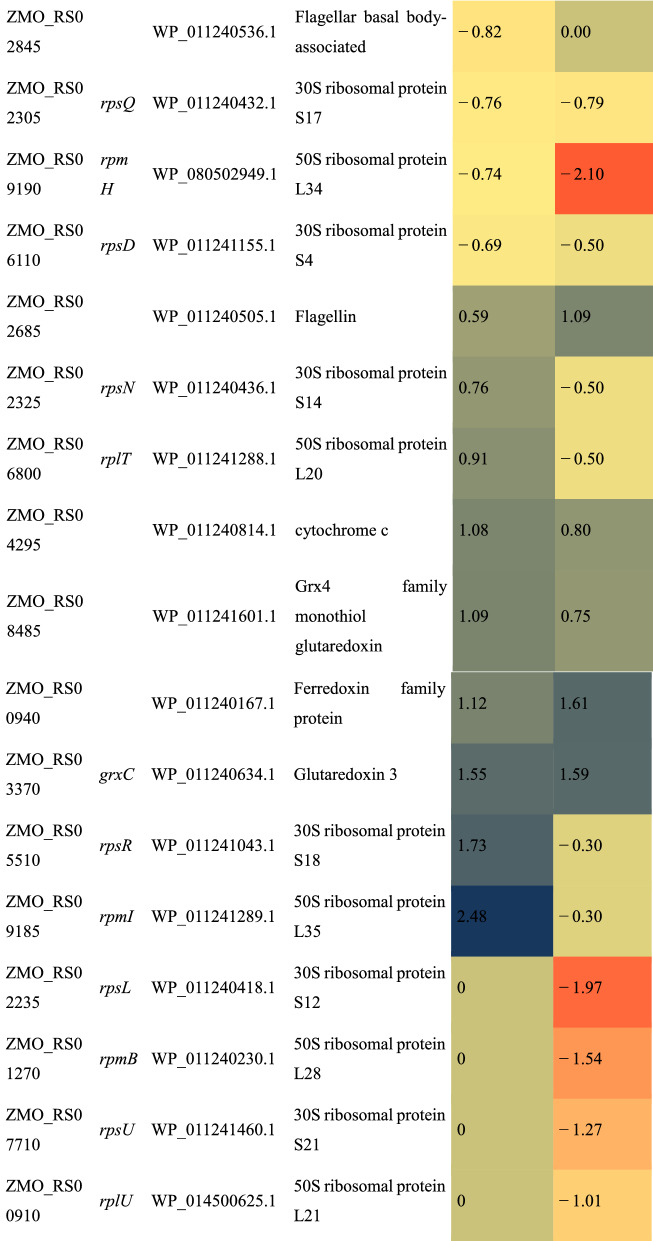

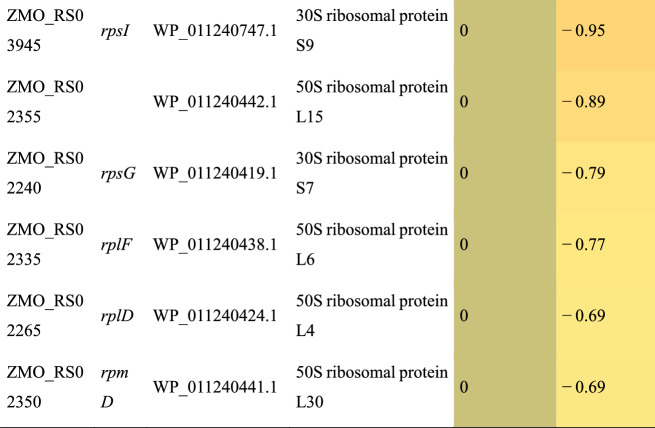
Log2FC expression of the proteins involved in ribosome assembly and functioning under inhibitor (AF) as compared to control (RM) treatment

Blue and red cells are up-regulated and down-regulated proteins, respectively (*p* < 0.05)

### Candidate genes and proteins involved *Z. mobilis* tolerance mechanism

Transcriptional and proteomic levels of ZMO_RS02930 and ZMO_RS08760 encode GroEL protein for adaptation to acidic stress. This protein (GroEL protein) is expressed higher in ZM532 than in ZM4. Two novel genes (Novel00013 and Novel00014), which encode GroEL protein, were up-regulated exclusively in the mutant strain ZM532, which may account for the robustness of our mutant strain against AF stresses and could lead to high ethanol production (Additional file 1: Table S7, S8, S9, S10). Our transcriptome and proteomics data showed that molecular chaperone complexes ZMO_RS06375 (ClpB), and ZMO_RS01740 (ClpA) were up-regulated in both strains (Additional file 1: Tables S7, S8, S9, S10). Other chaperone proteins, ZMO_RS04435 (Hsp20 family protein) and ZMO_RS03810 (peptidylprolyl isomerase), were up-regulated, and both proteins were exclusively found only in mutant strain ZM532 (Additional file 1: Tables S7, S10). Up-regulation of these genes and proteins may have contributed to the improved tolerance of *Z. mobilis* to acetic acid and furfural stressors.

Our transcriptomics and proteomics results demonstrated that sulfur encoding genes ZMO_RS06540 and ZMO_RS03345 were up-regulated in ZM532 and ZM4, but their expressions were higher in ZM532 compared to ZM4 (Additional file 1: Table S7, S8, S9, S10). As furfural and acetic acid could inhibit sulfur amino acid biosynthesis either by restricting the availability of reduced sulfur (H_2_S) from sulfate or by inhibiting the incorporation of reduced sulfur into cysteine. Up-regulation of these genes may have contributed to the improved tolerance of *Z. mobilis* to acetic acid and furfural stressors.

Up-regulated proteins, ZMO_RS00760 (Pgk), ZMO_RS05570 (gpmA), and ZMO_RS07905 (glucokinase) were found only in mutant strain ZM532 in both transcriptomics and proteomics data, while ZMO_RS06615 (pgl) was up-regulated in both strains (Additional file 1: Tables S7, S8, S9, S10). Moreover, alcohol dehydrogenase encoded ZMO_RS05560, which was up-regulated in both strains, but these genes were doubly expressed in ZM532 compared to ZM4. ZMO_RS03395 (Hydroxyacylglutathione hydrolase), ZMO_RS05445 (glucose-6-phosphate isomerase) and ZMO_RS03970 (galactose-1-epimerase) were up-regulated in ZM532 (Additional file 1: Tables S7, S10). These genes may partly account for the robustness of our mutant strain ZM532 against acetic acid and furfural stresses. DNA repair genes and proteins such as ZMO_RS07115 (RecF) and ZMO_RS01515 (DNA mismatch repair enzyme mutL) were up-regulated with higher expression levels in ZM532 than ZM4 (Additional file 1: Tables S7, S8, S9, S10). These indicate that *Z. mobilis* and ZM532 (mutant) could reduce DNA damage caused by furfural and acetic acid by activating relevant genes or DNA replication, base repair, and recombination. We also found the ZMO_RS03175 (ppk) gene, which encodes RNA degradosome polyphosphate kinase in ZM532 (Additional file 1: Tables S7, S10).

In the current omics (transcriptome and proteome) study, transcriptional response regulatory proteins (YebC/PmpR family DNA-binding transcriptional regulator), ZMO_RS00645, ZMO_RS05270, ZMO_RS06920 (TetR family transcriptional regulator), ZMO_RS05215 (phosphate regulon transcriptional regulatory protein PhoB), ZMO_RS06945 (transcription anti-termination factor NusB) and ZMO_RS05270 transcriptional response regulator were up-regulated exclusively in ZM532 (Additional file 1: Tables S7, S10).

Proteins and genes associated with translation, ribosomal structure, and biogenesis ZMO_RS00305 (ybeY) and ZMO_RS04930 (tsaE) were up-regulated only in ZM532 (Additional file 1: Tables S7, S10). However, several proteins such as ZMO_RS07450 (tilS), ZMO_RS03355, ZMO_RS00625 (trmB), and ZMO_RS06475 were down-regulated under the stress conditions (Additional file 1: Tables S7, S8, S9, S10).

Moreover, we identified four key mutations ZMO_RS00235, ZMO_RS03765 ZMO_RS06410 and ZMO_RS04295 in our transcriptome and proteomic data (Additional file 1: Tables S7, S8, S9, S10), which encode glutamine-fructose-6-phosphate aminotransferase, arginine-tRNA ligase, FUSC family protein and cytochrome c (Table 1) as described earlier and our current sanger re-sequencing data. The gene ZMO_RS00235 was up-regulated in the mutant strain, which may contribute to AF stress tolerance. Moreover, ZMO_RS03765 is associated with arginine biosynthesis, which is crucial for acid stress. However, no concrete evidence has been adduced for the role of arginine in acid resistance; the cell wall/membrane itself may be important to maintain cell integrity. As previously reported by Ryan et al., ADI genes allow Listeria monocytogenes to survive under acidic conditions; with arginine, their expression is higher at low pH. Based on research conducted by Huang et al. (2015) L-arginine used to suppress the biofilm formation of Streptococcus mutants but there is no clear evidence that biofilm contributes to acid tolerance, cell wall/membrane is necessary to maintain cell integrity. In addition, ZMO_RS06410 might improve fusidic acid resistance and methicillin resistance. It may also be useful for *Z. mobilis* to survive acid stress. One of the genes, ZMO RS04295 encodes Monofunctional biosynthetic peptidoglycan transglycosylase (MBPT) and cytochrome c to promote glycan chain synthesis in bacterial cell walls, and its function is identical to that of DNA polymerases (Baker et al., 2010) (Additional file 1: Tables S7, S8, S9, S10). This could be important to preserve the integrity and tolerance of the cells to the inhibitors. These mutations have roles in acids tolerance as cytochrome C may provide some protective layer sheet against AF stresses. In addition, ZMO_RS04890 encoded TatD family hydrolase were up-regulated in the mutant strain ZM532 and was suppressed on wild-type strain ZM4 under acidic condition may be crucial against acids resistance. Follow-up studies further showed that TatD bears 3′–5′ exonuclease activity that processes single-stranded DNA in DNA repair (Additional file 1: Tables S7, S8, S9, S10). Since TatD is an evolutionarily conserved protein, it should have an important cellular role. However, our understanding of this protein is largely hampered due to a lack of knowledge of its biological functions and structure-to-function relationship, this warrant future study to provide evidence for TatD in DNA repair. ZMO_RS01205, OstA encoded organic solvent tolerance protein was up-regulated gene in mutant strain ZM532 and suppressed in wild-type strain ZM4 under acidic conditions (Additional file 1: Tables S7, S8, S10). In the future, it will be critical to investigate genes' single and combined effects on the increase in Ost activity in response to salt and acid stress. Moreover, it will also assist in identifying the transcriptional regulator proteins which are important in the Ost mechanisms in ZM4. We identified up-regulated gene ZMO_RS08390, encoding carbohydrate porin compared to resistance strain with wild-type (AF_ZM532_vs_AF_ZM4) (Additional file 1: Tables S7, S10). Porins are proteins on the outer membrane of the bacteria cell wall that regulate cellular permeability and drug resistance; a systematic approach to porin roles in ZM4 physiology and acid resistance (AF) does not exist yet.

### Correlations between transcriptomic and proteome

Integrative molecular approaches such as genome, transcriptome, and proteome may help us understand toxicant's effects at multiple levels of the biological organization while also facilitating risk assessment.

**Fig. 4:**
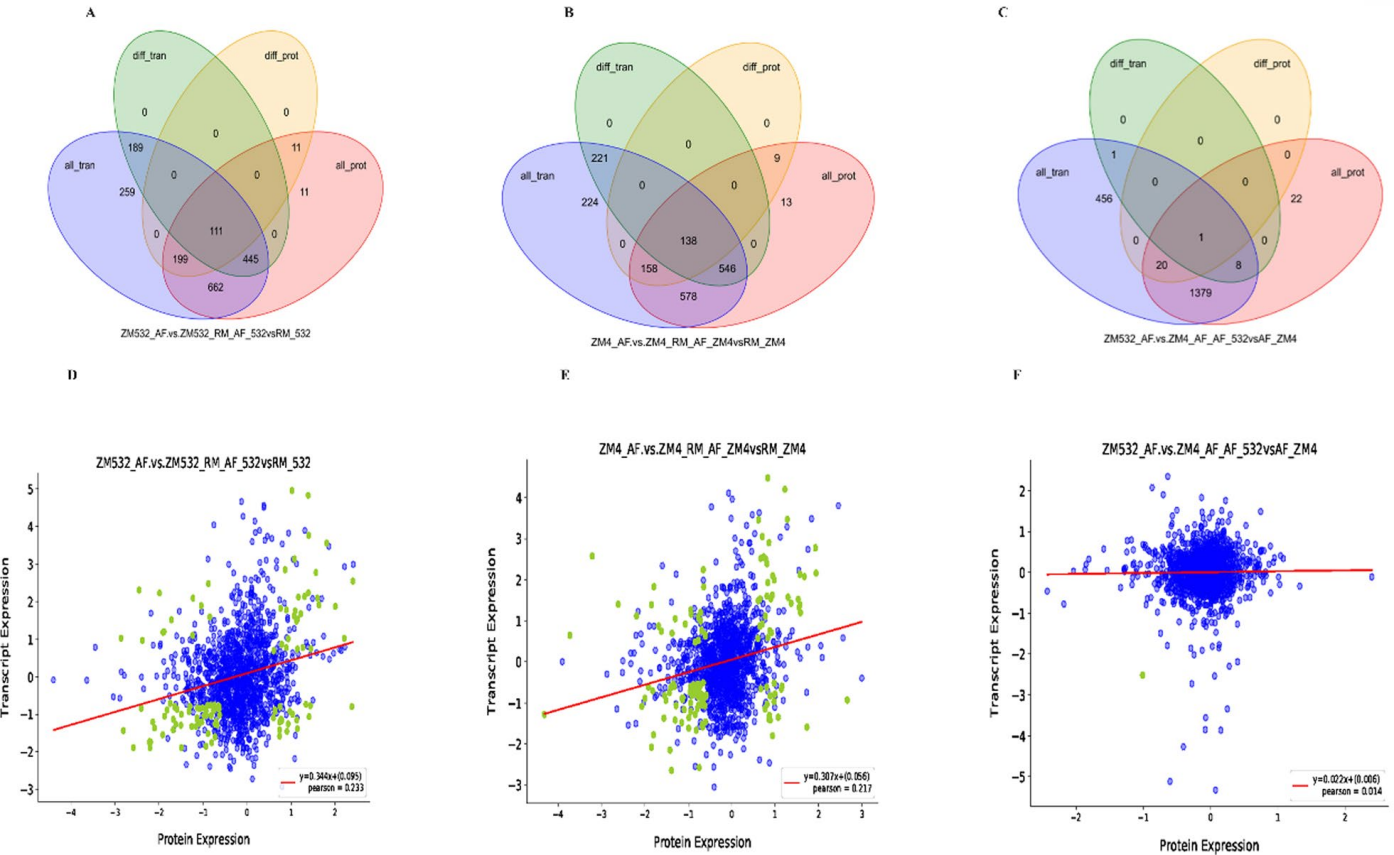
Correlation analysis between transcriptome and proteome. **A**-**C** The Venn diagram is determined by transcriptome and proteome expression., all the transcripts in our transcriptome data; different_trancripts, differentially expressed transcripts between ZM532_AF_vs_ZM532_RM_AF_532_vs_RM_532, ZM4_AF_vs_ZM4_RM_AF_ZM4_vs_RM_ZM4 and ZM532_AF_vs_ZM4_AF_AF_532_vs_AF_ZM4; different_proteins, distinct expressed proteins between ZM4 and ZM532; all_prot, all the proteins in our proteome data; **D**–**F** Analysis of correlations between transcriptome and proteome expression levels

The transcriptome data was combined with the proteome data to identify corresponding relationships. A total of 662, 578, 1379 IDs were identified in ZM532_AF_vs_ZM532_RM_AF_532_vs_RM_532, ZM4_Af_vs_ZM4_RM_AF_ZM4_vs_RM_ZM4 and ZM532_AF_vs_ZM4_AF_AF_532_vs_AF_ZM4 by both RNA-seq and proteomics (Fig. 4A–C). In the three groups, 111, 138, and 1 unique protein related to transcriptome DEGs were identified, respectively. Correlation analysis was performed between the multiple genes (proteins) identified by transcriptome and proteome study in the three groups (Fig. 4A–C). Among mRNA and the corresponding protein, the Pearson correlation coefficient was positive (Pearson = 0.233, 0.217, and 0.014) for all groups (Fig. 4D–F). As a result, we suggest that it is critical to assess protein expression to understand phenotypic changes and not rely solely on the transcriptional level.

### Verification of RNAseq candidate genes involved in *Z. mobilis* tolerance mechanism

**Fig. 5:**
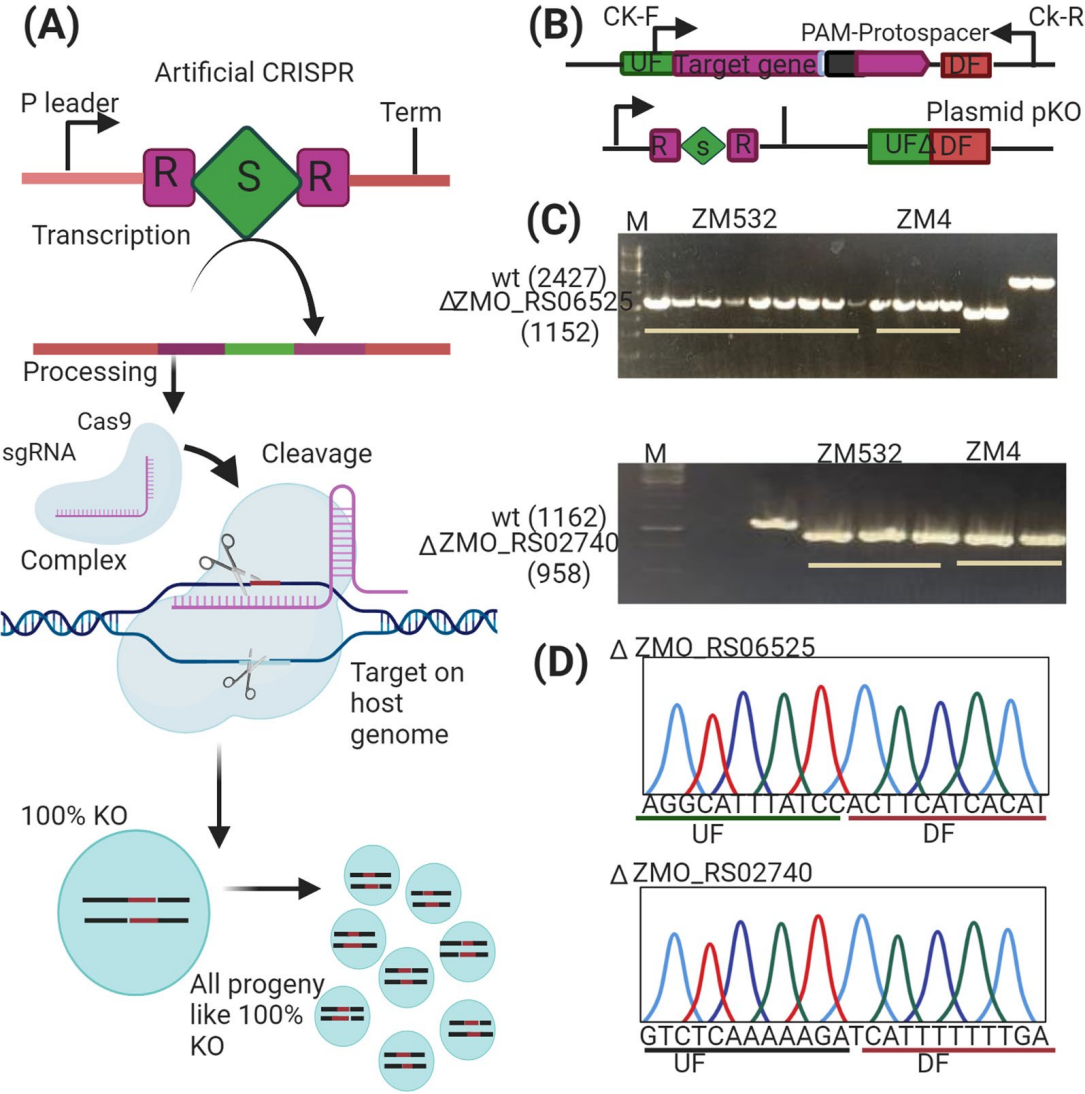
Establishment of the Type I-F CRISPR-based genome engineering system for *Z. mobilis.* **A** A self-targeting plasmid contained an artificial CRISPR locus. **B** Design of the self-targeting CRISPR and the donor DNA in knockout plasmids; **C** deletion of mutants by screening of colony PCR. **D** Confirmation by Sanger sequencing

CRISPR–Cas Type I-F edited *Z. mobilis* revealed that the protospacer-bearing plasmids had significant interference activity. For self-targeting and genome engineering, we transferred the DNA cleavage of interest to an adjacent protospacer motif (PAM)-flanking sequence on the chromosome. The ZMO_RS02740 (204 bp) and ZMO_RS06525 (1275 bp) were selected as engineering targets. Plasmids were primarily constructed to import a leader-repeat-spacer-repeat cassette of an artificial CRISPR expression individually (Fig. 5A). A donor DNA comprising of two homology arms for supporting homologous recombination engineered to carry expected mutations to improve the reliability of selected genotypes by self-targeting (Fig. 5B). By using genome engineering plasmids pKO-ZMO_RS02740 and pKO-ZMO_RS06525 (Fig. 5B), both target genes were successfully deleted in ZM4 and ZM532 (Fig. 5C, D). The genotypes of randomly selected transformants in ZM532 and ZM4 were analyzed by colony PCR and Sanger sequencing, confirming the deletion of both genes (Fig. 5C, D).

### Cell growth, glucose consumption, and ethanol production of mutant strains ∆ZMO_RS02740 and ∆ZMO_RS06525 under AF conditions

**Fig. 6:**
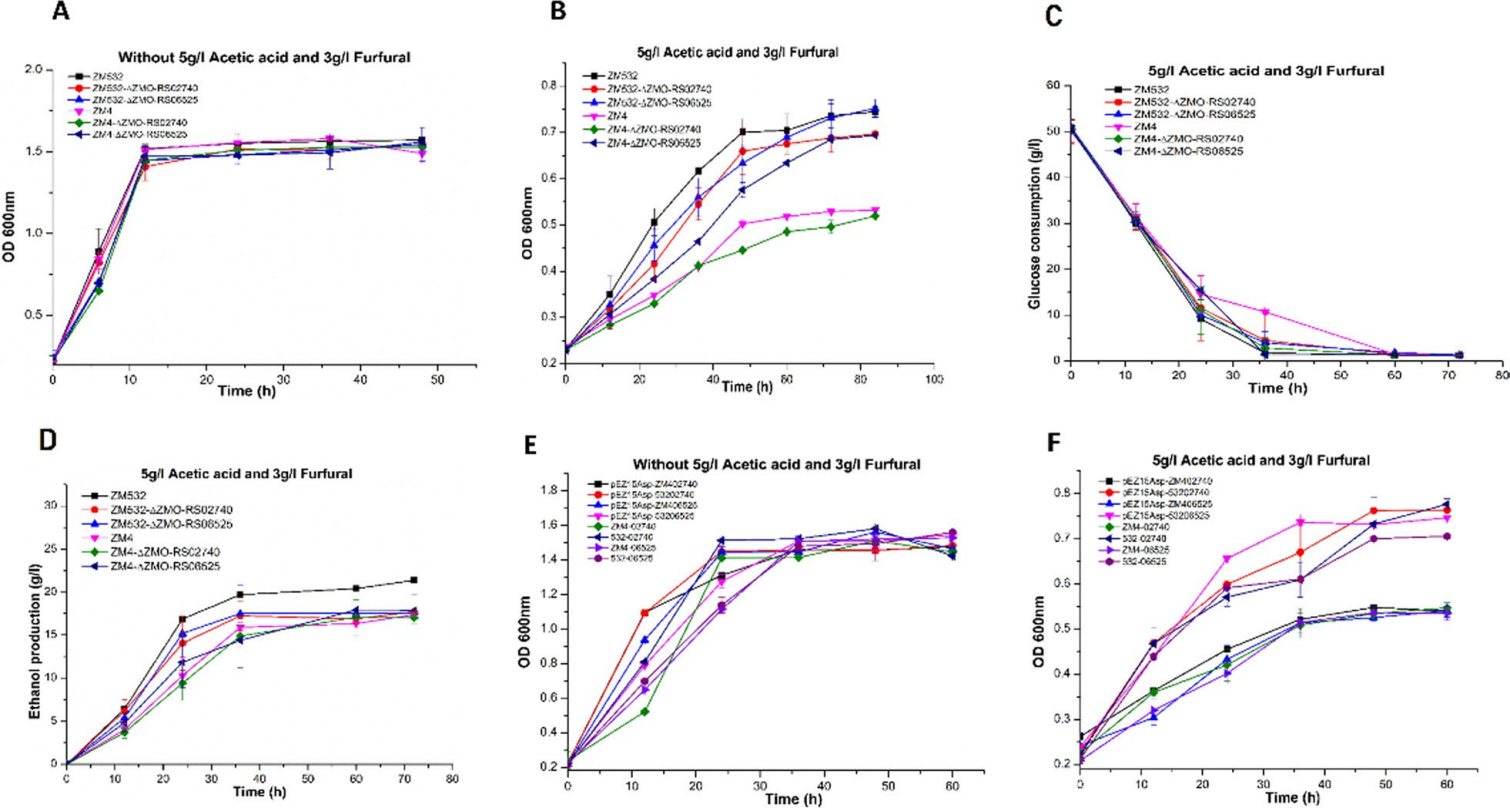
**A** Cell growth of Control ZM532, ZM4; and Knockout Mutants ZM532∆ZMO_RS02740; ZM4∆ZMO_RS02740; ZM532∆ZMO_RS06525; and ZM4∆ZMO_RS06525 under RM; **B** cell growth; **C** glucose consumption and **D** ethanol production; wild type ZM4; ZM532 and Knockout Mutants ZM532∆ZMO_RS02740; ZM4∆ZMO_RS02740; ZM532∆ZMO_RS0652 and ZM4∆ZMO_RS06525 under AF stress conditions; **E** cell growth of Control strains with empty vector such as pEZ15Asp-ZM402740; pEZ15Asp-ZM53202740; pEZ15Asp-ZM406525 and pEZ15Asp-ZM53206525; and overexpress mutants with *ptet* promoter such as ZM4-02740; ZM532-02740; ZM4-06525 and ZM532-06525 under RM; **F** Cell growth of Control strains with empty vector such as pEZ15Asp-ZM402740; pEZ15Asp-ZM53202740; pEZ15Asp-ZM406525 and pEZ15Asp-ZM53206525; and overexpress mutants with *ptet* promoter such as ZM4-02740; ZM532-02740; ZM4-06525 and ZM532-06525 under AF stress conditions. The results are demonstrated in arbitrary (means ± SD). The error bars represent the standard deviation. Three replicates were performed for each strain

Four mutant strains (ZM532∆ZMO_RS02740, ZM4∆ZMO_RS02740, ZM532∆ZMO_RS06525, and ZM4∆ZMO_RS06525) were investigated under RM and AF conditions, respectively. AF affects glucose consumption, cell growth, and ethanol production (Fig. 6A–D). With the same initial OD600, when strains were cultivated for 36 h, the ZM4∆ZMO_RS06525 OD600 values were increased by 5.6% compared with the wild-type strain ZM4 under the same initial OD600. This OD600 value decreased when ZMO-RS02740 was knocked out in ZM532 and ZM4. The growth activity and glucose consumption of mutant strains ZM532∆ZMO_RS02740 and ZM4∆ZMO_RS02740 were decreased and thus, increasing fermentation time from 42 h in ZM532 to 55 h. Ethanol production was 58% higher in ZM532 than that in ZM532∆ZMO_RS02740. However, in ZMO_RS06525 knockout in ZM4, the time of fermentation was significantly decreased from 60 h for ZM4 to 42 h for ZM4∆ZMO_RS06525, which contributed to a 45.54% increase in ethanol production (Table 4). These results highlight that the mutant, ZM532 has more ability to convert sugar to ethanol and withstand toxic conditions. These observations are consistent with our transcriptome results.

**Table 4:** Fermentation time of glucose consumption (Time), ethanol titer, yield, and productivity of wild-type ZM4, ZM532 and other mutant strains

Strain	Glucose consumed g/L	Time (h)	Ethanol			Theoretical value ratio (%)
			Titer (g/L)	Yield (g/g glucose)	Productivity (g/L/h)	
50 g/L glucose + 5 g/L acetic acid + 3 g/L furfural						
ZM532 (Control)	50.40 ± 0.86	42	21.39 ± 0.570	0.424 ± 0.005	0.509 ± 0.010	83
ZM532∆ZM0-RS02740	50.06 ± 0.17	55	17.71 ± 0.001	0.353 ± 0.010	0.322 ± 0.010	69
ZM532∆ZM0-RS06525	50.56 ± 0.69	55	17.50 ± 0.057	0.346 ± 0.001	0.318 ± 0.001	67
ZM4 (Control)	50.05 ± 0.23	60	17.57 ± 0.050	0.349 ± 0.010	0.290 ± 0.001	68
ZM4∆ZM0-RS02740	50.24 ± 0.63	55	17.37 ± 0.100	0.345 ± 0.010	0.320 ± 0.001	67
ZM4∆ZM0-RS06525	50.03 ± 0.55	42	17.87 ± 0.100	0.357 ± 0.010	0.430 ± 0.010	69

Values are the means and standard deviations of representative experiment with three technical replicates

### Evaluation of candidate resistance genes under AF tolerance by a complementary study

Further, four plasmids bearing candidate operons were constructed based on a shuttle vector pEZ15Asp with *Ptet* as the promoter to investigate the impact of these genetic variants on combined AF resistance. These plasmid constructs were then separately transferred into competent cells of ZM532 and ZM4, including the empty vector pEZ15Asp as the control. Besides, recombinant strains' expression profiles were examined without stress and with stress (AF) conditions to analyze their effect on cell growth. Hence, these results suggested that the ZM406525 encoding an MFS containing recombinant strain failed to contribute to the resistance of acids in ZM4 and ZM532, which is consistent with our RNA-Seq outcome (Fig. 6F); while all strains had approximately similar growth rates under normal conditions (Fig. 6E). In addition, the up-regulated expression of ZMORS02740 (Chemotaxis protein Mot A) was similar to our RNA-seq results (Fig. 6F).

### qPCR validation of differentially expressed genes under inhibitory (AF) conditions

The results of qPCR showed three DEGs (ZMO_RS02740, ZMO-RS00080, and ZMO-RS08110) were up-regulated in ZM532, while ZMO-RS03395, ZMO-RS08600, and ZMO_RS06525 were down-regulated in the same strain ZM532. Conversely, among the selected DEGs in ZM4, ZMO-RS00065 and ZMO-RS02800 were up-regulated, while ZMO-RS01385 and ZMO-RS03775 were down-regulated, which are in consonance with the transcriptome results (Additional file 1: Fig. S8; Table S7, S8). These genes had high expression either as up or down-regulated in RNA-seq results, giving a clue for their potential for functional validation in our subsequent experiments.

## Discussion

Lignocellulose inhibitors are composed of aldehydes such as Hydroxymethylfurfural, furfural, and weak acids, particularly acetic acid [17]. Ethanol and the toxicity of these inhibitors are influenced by bacterial cells, lipid structure and fluidity, membrane permeability, and physiological processes, including intake of nutrients, electron transport chain, and absorption and energy transduction [66]. Resistance to these inhibitors is a complex phenotype controlled by mysterious regulatory mechanisms. One of the main challenges of cost-competitive bioethanol production from lignocellulosic biomass is the development of resistant strains toward stresses. Exploiting the global regulatory landscape may show different impacts on bacterial metabolism leading to the overlap of cell stress responses. Synthesis of resistant strains by functional and evolutionary engineering is a valuable way to distinguish genetic elements important to the resistance of inhibitors [67]. In our previous study, we constructed a mutant ZM532 by genome shuffling, which is superior to the parental strain and *Z. mobilis*. However, the molecular mechanisms underlying the enhanced tolerance and shortened fermentation time were largely unknown. Therefore, genetic changes, proteins, and gene expression profiles under AF stress or without stress conditions were investigated using transcriptomics and proteomics to unravel the molecular mechanisms in the wild type ZM4 and mutant strain ZM532. We also identified 1865 and 14 novel DEGs in ZM532 and wild-type ZM4, while 1532 proteins were identified in ZM532 and wild-type ZM4 by label free proteome using the genome of parental strain ZM4 (ATCC 31821) as cited in [68]. We identified one of the most important up-regulated genes, ZMO_RS08390, encoding carbohydrate porin compared to resistance strain with wild-type. Porins are proteins on the outer membrane of the bacteria's cell wall that regulate cellular permeability and drug resistance [69]. However, a number of studies on porin resistance to antibiotics are available [70, 71], but a systematic approach to porin roles in ZM4 physiology and AF resistance does not exist yet. Porins primary natural function is to transport polar nutrients, such as amino acids, carbohydrates, and other ions [72]. Moreover, porins play an important role in Gram-negative bacterial envelope integrity by facilitating the passive transport of various chemicals. For example, non-specific porins, such as OmpA, found in outer membrane proteins, promote the passive transport of many small molecules [73, 74]. Additionally, this protein is related to peptidoglycan via a flexible periplasmic motif that interacts non-covalently with peptidoglycans [75]. Because porins are linked to antibiotic resistance in Gram-negative bacteria, which enables them to passively diffuse drugs throughout the outer membrane. Although prior research suggested that porins regulate antibiotic resistance, the contribution of porin in resistance to acids (AF) is largely unknown and has not been studied yet. In addition, ZMO_RS04890 encoded TatD family hydrolase, was up-regulated gene found only in mutant strain ZM532 and participates in DNA fragmentation during apoptosis in *S. cerevisiae* [76] and *Trypanosoma brucei* [77]. The previous study showed that TatD-knockout cells are less resistant to the DNA damaging agent hydrogen peroxide [78]. Hydrogen peroxide may induce various DNA lesions, double-strand breaks, oxidation, deaminated bases and sugar modifications [79, 80]. TatD has ability to remove deaminated nucleotide from DNA chain, inferring that it may be involved in H_2_O_2_-induced-DNA repair [78]. We also found ZMO_RS01205, OstA encoded organic solvent tolerance protein was up-regulated in mutant strain ZM532 in our omics data (transcriptome and proteome). An earlier studies reported gene ostA is one of the genes contributing to the level of organic solvent tolerance [81, 82].

Macromolecule recombination, replication, and repair are central molecular mechanisms for regulating and maintaining genetic information in microbes. Bacterial proteins, cell membranes, and DNAs are usually damaged under acidic conditions. Repair and resistance genes and proteins such as RecF, Recj, and DNA mismatch repair enzyme mutL and PPK could be improved to overcome these acidic destructions of macromolecules. We found DNA repair genes and proteins such as ZMO_RS07115 (RecF), ZMO_RS05530 (Recj), and DNA mismatch repair enzyme ZMO_RS01515 (mutL) were up-regulated with higher expression levels in ZM532 than ZM4. This indicates ZM4 and ZM532 (mutant) could reduce DNA damage caused by AF by activating relevant genes or DNA replication, base repair, and recombination. Our results are in line with those previously reported [21, 32, 83]. We also found ZMO_RS03175 (ppk) and was up-regulated in ZM532 (mutant) and ZM4 under AF stress which is consistent with findings of previous studies [31, 32, 83]. Up-regulation of these proteins is important for cell recovery from DNA damage caused by these inhibitors.

In the current study, transcriptional response regulatory proteins and genes ZMO_RS05270, ZMO_RS00645 (YebC/PmpR family DNA-binding transcriptional regulator), ZMO_RS06920 (TetR family transcriptional regulator), ZMO_RS05215 (phosphate regulon transcriptional regulatory protein PhoB*)*, transcription anti-termination factor ZMO_RS06945 (NusB) and ZMO_RS05270 (transcriptional response regulator) were up-regulated exclusively in ZM532. Proteins associated with translation, ribosomal structure, and biogenesis, such as ZMO_RS00305 (ybeY) and ZMO_RS04930 (tsaE) were up-regulated only in ZM532. However, several proteins such as ZMO_RS07450 (tilS), ZMO_RS03355, ZMO_RS00625 (trmB), and ZMO_RS06475 were down-regulated under the stress conditions (Additional file 1: Tables S7, S10). This agrees with the transcriptomic results of furfural and acetate-challenged *Z. mobilis* [21, 31, 84]. Down-regulation of these proteins suggests the overall synthesis of proteins to minimize cell growth [31]. This may be partly attributed to external stress that causes mRNA degradation and inhibits translation [85].

The Transcriptional and proteomic levels of ZMO_RS02930 and ZMO_RS08760 encode GroEL protein to adapt to acidic stress [83]. However, this protein (GroEL protein) was more highly expressed in ZM532 than in ZM4. An earlier study revealed that dank is critical for microbe survival in environmental stress conditions [86]. Besides, dank play a significant role in refolding of damaged proteins. Two novel genes (Novel00013 and Novel00014), which encode GroEL protein, were up-regulated exclusively in the mutant strain ZM53, which may account for the robustness of our mutant strain against AF stresses and could lead to high ethanol production (Additional file 1: Tables S7, S8, S9, S10). Previous studies have confirmed that these proteins are necessary for the normal growth of *E. coli* under toxic antibiotics [17, 31] and temperature stress conditions [87]. Our transcriptomics and the proteomic result showed that the expression level of Clp protease complex, like ZMO_RS01740 (clpA) and ZMO_RS06375 (clpB) were up-regulated in both ZM4 and mutant ZM532, but the expression level of Clp protease was higher in mutant ZM532 compared with ZM4 (Additional file 1: Tables S7, S8, S9, S10). These may be involved in protein remodeling and reactivation [83, 88–90] to enhance the expression of these proteins to protect DNA and protein from damage in acidic cytoplasm. However, our transcriptomics results demonstrated that sulfur encoding genes (ZMO_RS06540 and ZMO_RS03345) were up-regulated in ZM532 and ZM4 but their expressions were higher in ZM532 compared to ZM4 (Additional file 1: Tables S7, S8). As AF could inhibit sulfhur amino acid biosynthesis either by restricting the availability of reduced sulfur (H2S) from sulfate or by inhibiting the incorporation of reduced sulfur into cysteine. The inhibition of sulfate reduction is unlikely to represent the initial action of furfural that inhibits growth [91]. Up-regulation of these genes may have contributed to the improved tolerance of *Z. mobilis* to AF stressors.

Our omics data (transcriptomic and proteomic) showed that molecular chaperone ZMO_RS04435 (Hsp20 family protein), which regulates bacteria growth and survival under different stresses, was up-regulated in the mutant ZM532 (Additional file 1: Tables S7, S10). Hsp20 stabilizes archaea and bacterial membrane lipids and small HSPs in microbial pathogenesis [92–95]. However, chaperone ZMO_RS03810 (peptidylprolyl isomerase), which can maintain the overall reduction in the level and folding of OMPs and the induction of the periplasmic and ZMO_RS07675 (tetratricopeptide repeat protein) involves sensing and treatment of defective or incomplete protein structures under stress responses as previously discussed [92, 94] both proteins exclusively found only in mutant strain ZM532 (Additional file 1: Tables S7, S10). For inhibitor tolerance of *Z. mobilis* cells, control of these stress response molecular chaperones may be helpful.

The most critical part of living organisms is carbon metabolism. Up-regulated proteins are involved in the central carbon metabolism pathway's ED and TCA cycle routes. Although only one mole of ATP per single mole of glucose is provided by the ED route, the ED pathway in *Z. Mobilis* is almost twice the thermodynamically favorable pathway of Embden-Meyerhof-Parnas (EMP) in *E. coli* or *S. cerevisiae* [96]. Up-regulated proteins, Pgk, gpmA, and ZMO_RS07905 (glucokinase) were found only in mutant strain ZM532 in our omics data, while ZMO_RS06615 (pgl) was up-regulated in both strains (Additional file 1: Tables S7, 8, S9, S10). ZMO_RS03395 (hydroxyacylglutathione hydrolase), ZMO_RS05565 (2-hydroxy acid dehydrogenase), ZMO_RS05445 (glucose-6-phosphate isomerase), ZMO_RS03970 (galactose-1-epimerase), and ZMO_RS05445 (glucose-6-phosphate isomerase) were up-regulated in ZM532 (Additional file 1: Tables S7, S10). These genes may partly account for the robustness of our mutant strain ZM532 against AF stresses. The up-regulation of these genes stimulates more ATPs for acidic tolerance, as established by previous reports [21, 83].

Besides, recombinant strains' expression and knockout profile were examined in without and with stress (AF) conditions to analyze their effect on cell growth. Since the production of ethanol in *Z. mobilis* is closely linked to cell growth and substantially reduced by the inhibitory effects of toxic compounds [97]. Hence, these results suggested that the ZM406525 encoding an MFS containing recombinant strain failed to contribute to the resistance of acids in ZM4 and ZM532 when overexpressed; while after knockout of this gene, growth activity and glucose consumption increased, which is consistent with our RNA-Seq outcome**.** Many MFS transporters are essential for microorganisms to grow under stress conditions. Several superfamily transporters of major facilitators are important for microorganisms to develop under conditions of stress [98]. Gram-negative bacteria can reduce their entry by establishing a low permeability barrier to restrict the intracellular concentration of toxic inhibitors [99]. This non-specific phenomenon, such as the down-regulation of ZMO06525, which encodes an MFS transporter protein, was present in ZM4, while all strains had approximately similar growth rates under normal conditions. In addition, the up-regulated expression of ZMORS02740 (Chemotaxis protein, Mot A) was similar to our RNA-seq results. But for the *Ptet* promoter, the fermentation time of ZMORS02740 was reduced compared to mutant strain ZM532, which may be ZMORS02740 coordinating with some other genes and linker genes for acids resistance. When we combined this gene with *Ptet* promoter, their balance was disturbed, resulting in reduced fermentation time.

Our results revealed that the strain ZM532 is more capable of converting biomass to ethanol, and enhanced fitness in the toxicant-containing environment will benefit from this. Thus, ZM532 can enhance bioethanol production under AF conditions with ZM4 as a biocatalyst within a shorter fermentation period and greater productivity than ZM4. Overall, the *Z. mobilis* AF tolerance molecular mechanism presented in this study may be useful to synthetic biology focused on enhancing biological processes involved in ethanol production.

## Supplementary Information

**Additional file 1. ** Figure S1 Schematic procedure used in the knock-out of ZM532, ZMO_RS02740 and ZMO_RS06525 in ZM4 and ZM532. Figure S2 Venn diagram depicting the unique and shared differentially expressed genes between the two Z. mobilis, ZM532 strains, (**A**) AF_ZM532vsRM_532 (yellow) and its wild type AF_ZM4 vsRM_ZM4 (purple); (**B**) AF_ZM532vsAF_ZM4 (purple) and RM_ZM532vsRM_ZM4 in response to acetic acid and furfural combine treatments. Figure S3 Overview of the quantitative mass spectrometry results (**A**) Number of proteins identified in each sample; (**B**) Distribution of peptide length range; (**C**) Protein molecular weight distribution; (**D**) Reproducibility between biological replicates. Figure S4 Represented DEPs subcellular localization analysis. Figure S5 Venn diagram showing the shared and specific (DEP) between ZM4 and ZM532 in response to acetic acid and furfural treatments. Figure 6 KEEG enrichment analysis of the DEPs p < 0.05 in (**A**) ZM532_AF_vsZM532_RM and (**B**) ZM4_AF_vs_ZM4_RM; (**C**) AF_ZM532_vs_AF_ZM4; (**D**) RM_ZM532_vs_RM_ZM; (**E**) COG functional classification of the DE proteins. The proteins with significant homologies in the COG database were classified into 21 COG categories. Capital letters on the x-axis indicate COG categories on the right side of the histogram. Figure S7 KEGG pathway annotation for *Z. Mobilis*. The abscissa represents the number of proteins; the pathway categories are shown on the y-axis. Figure S8 Transcript abundance of 10 selected differentially expressed genes (DEGs) in the two samples (brown bar represents either ZM4 or ZM532 which gave similar expression; gray bar represents expression in the wild type, ZM4; ash bar represents expression in the mutant strain, ZM532. The error bar represents standard error of the three technical repeats. Table S1. List of Primer pairs used in study. Table S2. List of primers used for qPCR experiment. Table S3. List of primers, strains and plasmids. Table S4. List of primers, strains and plasmids. Table S5. INDEL in re-sequence ZM532 by comparing with previous published ten genome-shuffled mutant strain and Z. mobilis ZM4 (GenBank: AE008692.2). Table S6. Overview of the transcriptome sequencing dataset and quality check. Table S7 Differentially Expressed Genes of ZM532 strain in rich media and media with acetic + furfural treatments. Table S8 Differentially Expressed Genes of wild type ZM4 in rich media and media with acetic + furfural treatment. Table S9 Differentially Expressed proteins of ZM4 strain in rich media and media with acetic + furfural treatments. Table S10 Differentially Expressed proteins of ZM532 strain in rich media and media with acetic + furfural treatments.

## Data Availability

The *Z. mobilis* 532 has been deposited at Guangdong Microbial Culture Center (GDMCC) under the Accession Number GDMCC60527. The datasets generated and/or analyzed during the current study are included in this article, its supplementary information files and in the [NCBI] repository with Accession: GSE168900 [https://www.ncbi.nlm.nih.gov/geo/query/acc.cgi?acc=GSE168900]. Reviewer can access proteomics data by following login information, Username: reviewer_pxd030417@ebi.ac.uk, Password: S0ufj19u.
