# Supplementary material for: Molecular mechanism of engineered Zymomonas mobilis to furfural and acetic acid stress
Source: Microb Cell Fact. 2023 May 2;22:88. doi: 10.1186/s12934-023-02095-1 (PMC10152622; doi:10.1186/s12934-023-02095-1)
Supplement: Supplementary file 1 — Additional file 1. Figure S1 Schematic procedure used in the knock-out of ZM532, ZMO_RS02740 and ZMO_RS06525 in ZM4 and ZM532. Figure S2 Venn diagram depicting the unique and shared differentially expressed genes between the two Z. mobilis, ZM532 strains, (A) AF_ZM532vsRM_532 (yellow) and its wild type AF_ZM4 vsRM_ZM4 (purple); (B) AF_ZM532vsAF_ZM4 (purple) and RM_ZM532vsRM_ZM4 in response to acetic acid and furfural combine treatments. Figure S3 Overview of the quantitative mass spectrometry results (A) Number of proteins identified in each sample; (B) Distribution of peptide length range; (C) Protein molecular weight distribution; (D) Reproducibility between biological replicates. Figure S4 Represented DEPs subcellular localization analysis. Figure S5 Venn diagram showing the shared and specific (DEP) between ZM4 and ZM532 in response to acetic acid and furfural treatments. Figure 6 KEEG enrichment analysis of the DEPs p < 0.05 in (A) ZM532_AF_vsZM532_RM and (B) ZM4_AF_vs_ZM4_RM; (C) AF_ZM532_vs_AF_ZM4; (D) RM_ZM532_vs_RM_ZM; (E) COG functional classification of the DE proteins. The proteins with significant homologies in the COG database were classified into 21 COG categories. Capital letters on the x-axis indicate COG categories on the right side of the histogram. Figure S7 KEGG pathway annotation for Z. Mobilis. The abscissa represents the number of proteins; the pathway categories are shown on the y-axis. Figure S8 Transcript abundance of 10 selected differentially expressed genes (DEGs) in the two samples (brown bar represents either ZM4 or ZM532 which gave similar expression; gray bar represents expression in the wild type, ZM4; ash bar represents expression in the mutant strain, ZM532. The error bar represents standard error of the three technical repeats. Table S1. List of Primer pairs used in study. Table S2. List of primers used for qPCR experiment. Table S3. List of primers, strains and plasmids. Table S4. List of primers, strains and plasmids. Table S5 [file 12934_2023_2095_MOESM1_ESM.docx]

**
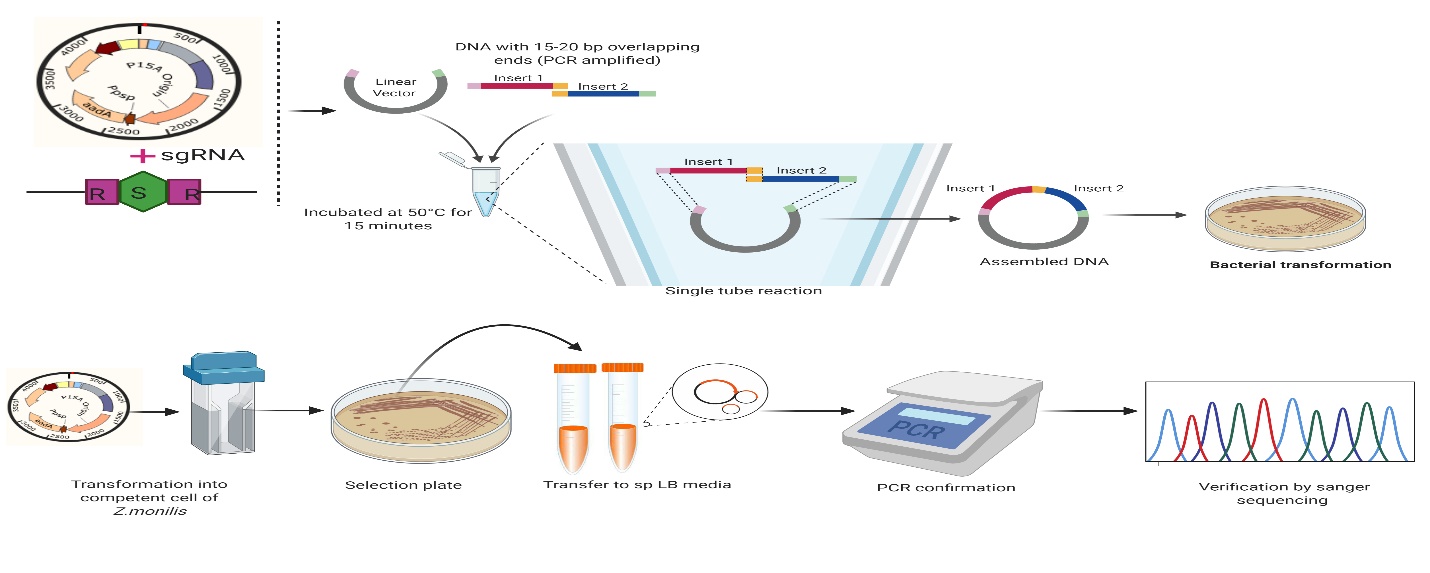
**

**Figure S1** Schematic procedure used in the knock-out of ZM532, ZMO_RS02740 and ZMO_RS06525 in ZM4 and ZM532.


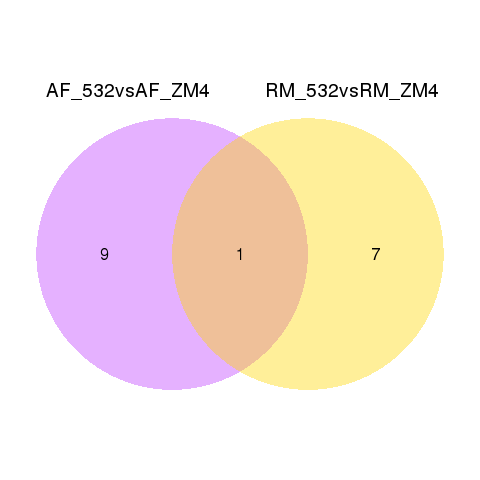

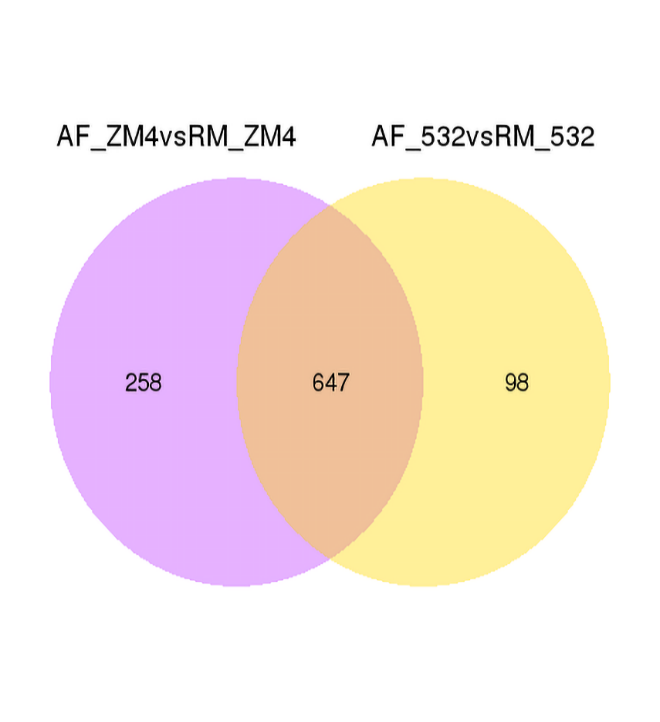


**Figure S2** Venn diagram depicting the unique and shared differentially expressed genes between the two *Z. mobilis*, ZM532 strains, **(A)** AF_ZM532vsRM_532 (yellow) and its wild type AF_ZM4 vsRM_ZM4 (purple); **(B)** AF_ZM532vsAF_ZM4 (purple) and RM_ZM532vsRM_ZM4 in response to acetic acid and furfural combine treatments


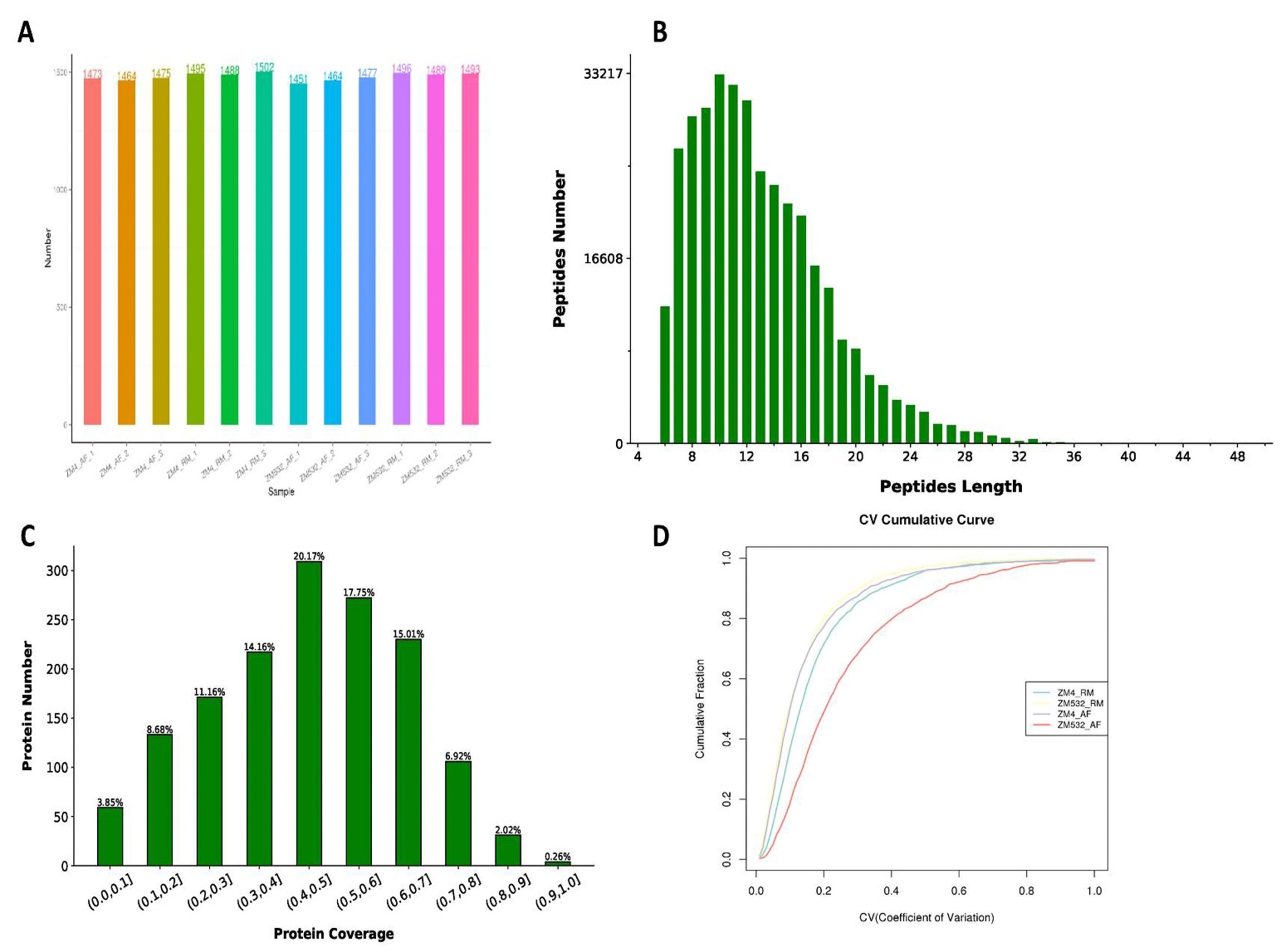


**Figure S3** Overview of the quantitative mass spectrometry results **(A)** Number of proteins identified in each sample; **(B)** Distribution of peptide length range; **(C)** Protein molecular weight distribution; **(D)** Reproducibility between biological replicates.


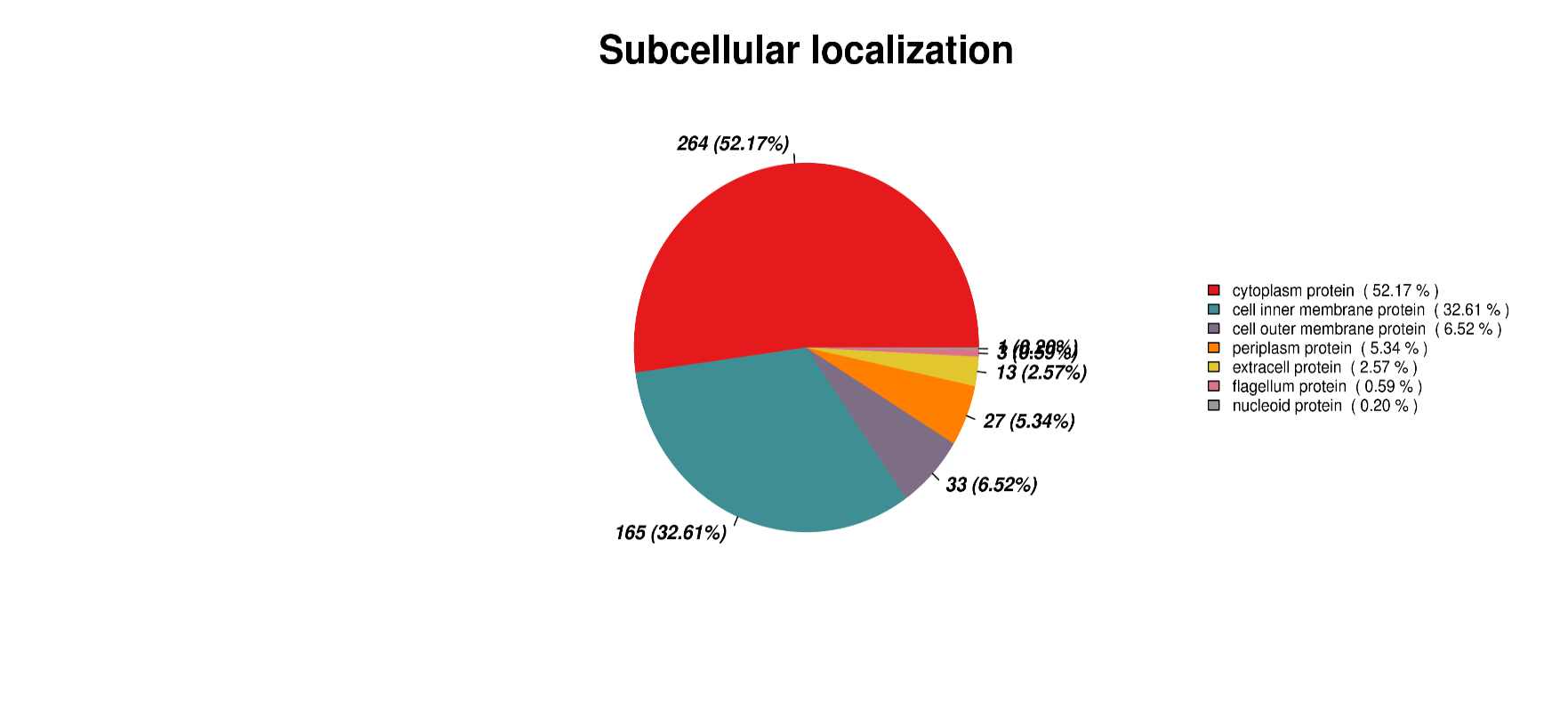


**Figure S4** Represented DEPs subcellular localization analysis.

**
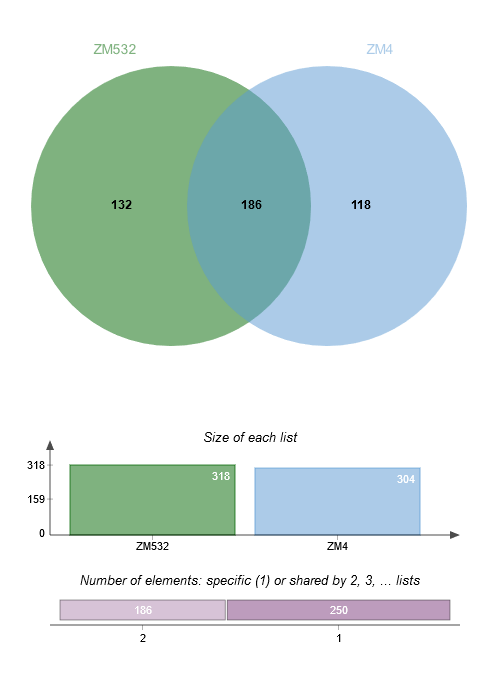
**

**Figure S5** Venn diagram showing the shared and specific (DEP) between ZM4 and ZM532 in response to acetic acid and furfural treatments.


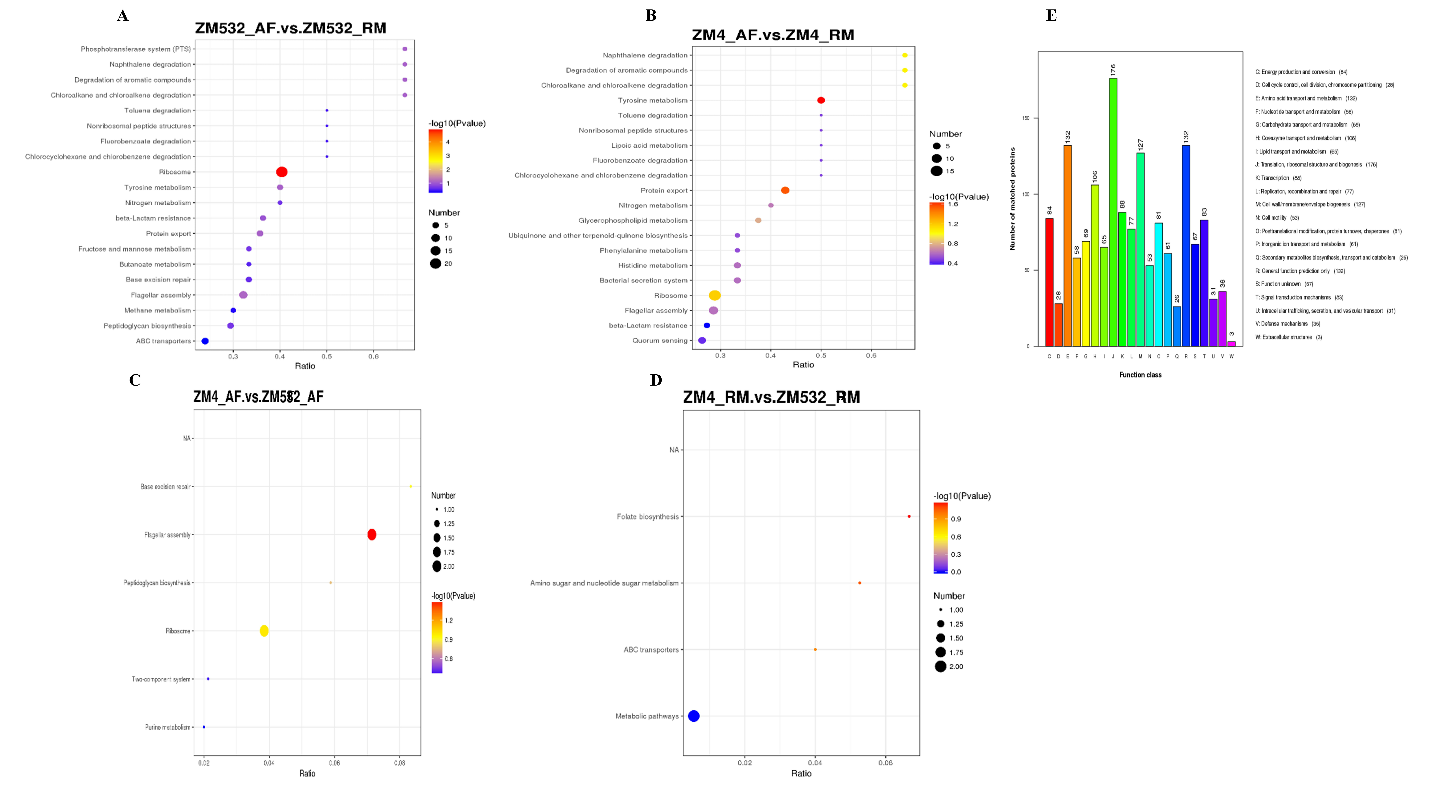


**Figure 6** KEEG enrichment analysis of the DEPs *p* < 0.05 in **(A)** ZM532_AF_vsZM532_RM and **(B)** ZM4_AF_vs_ZM4_RM; **(C)** AF_ZM532_vs_AF_ZM4; **(D)** RM_ZM532_vs_RM_ZM; **(E)** COG functional classification of the DE proteins. The proteins with significant homologies in the COG database were classified into 21 COG categories. Capital letters on the x-axis indicate COG categories on the right side of the histogram


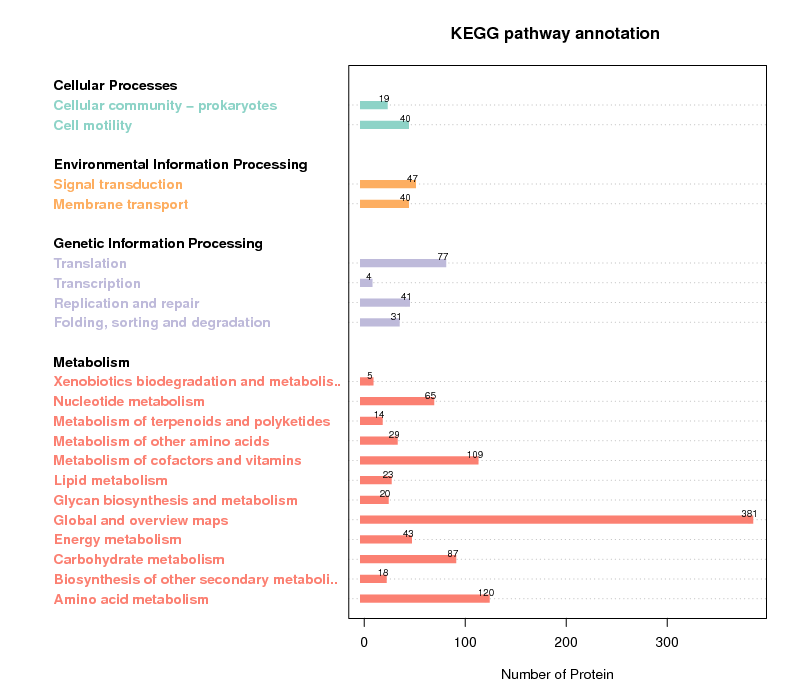


**Figure S7** KEGG pathway annotation for *Z. Mobilis*. The abscissa represents the number of proteins; the pathway categories are shown on the y-axis


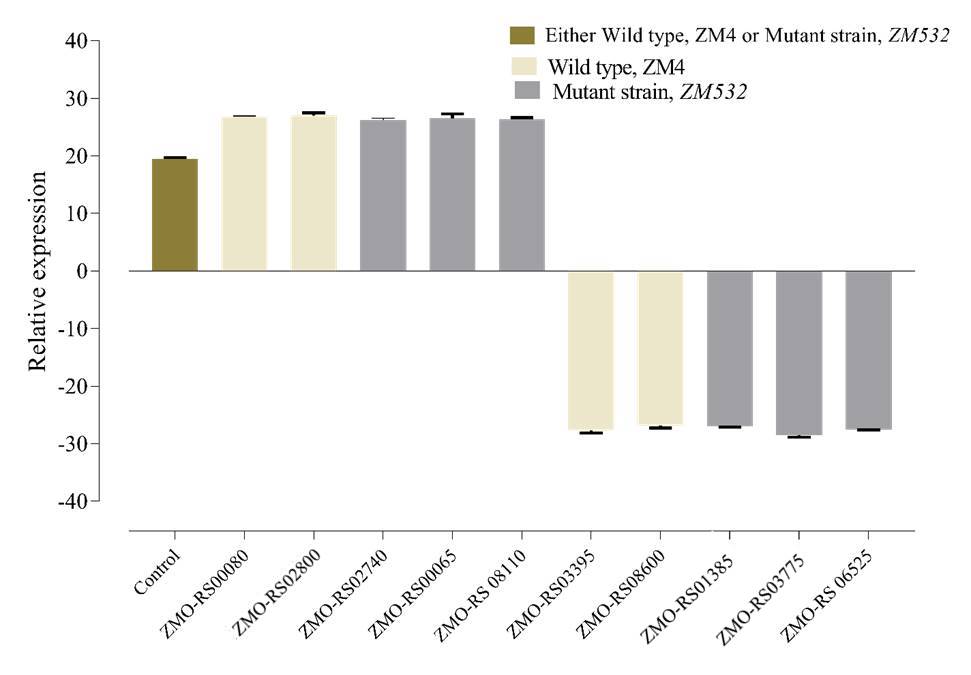


**Figure S8** Transcript abundance of 10 selected differentially expressed genes (DEGs) in the two samples (brown bar represents either ZM4 or ZM532 which gave similar expression; gray bar represents expression in the wild type, ZM4; ash bar represents expression in the mutant strain, ZM532*.* The error bar represents standard error of the three technical repeats

**Table S1. List of Primer pairs used in study**

| **Target Locus position**  **(Start to end)** | **Forward primer**  **(5΄ to 3΄)** | **Reverse primer**  **(5΄ to 3΄)** | **Product**  **size (bp)** | **Tm** |
| --- | --- | --- | --- | --- |
| 51467-52467 | GATGTCCTGTTCTTCTTGTTGT | TTGAAAGCGAAACTGATACCG | 941 | 56 ^o^ C |
| 589952-590952 | CATGGCCGATCAGGGATTAT | GGGTCGAAAAAGTTGCATTT | 991 | 57 ^o^ C |
| 848708-849708 | CTGTTCAGAAATCCGATGGTAG | TGGCAAGCTATCATCTTCATC | 953 | 56 ^o^ C |
| 970808-971808 | TCTGGTTTTGTCATAGCGTGT | GGCTCAAATCCAGTTAAATCC | 990 | 56 ^o^ C |
| 970559-971559 | TCGAGATTATCTTCCTGTGATTGC | AATTGAGAACAAGTTTTGTAGGGC | 911 | 58.8^o^ C |
| 975003-976003 | GATACACCTCTATAATCCGCC | CGGCATATTCTGCAAAGACA | 950 | 56 ^o^ C |
| 1612075-1613075 | TCTTGGGTTGGTTAATTGAGG | TTCACGCACAGGTGTCATAG | 953 | 56 ^o^ C |
| 2055263-2056263 | GTCAGAAGATCCAGACGATG | GAAAAAGACCTATTCAATAACCT | 871 | 56 ^o^ C |
| 1001780-1002780 | TGAATGGAGCGTTACATGGATA | GAACTCTGTTTGAAGGTTTGGT | 970 | 56 ^o^ C |
| 1265574-1266574 | TAATAATTCAGCGCATAGGCAG | CCTTGGGGAACAAAAACCAC | 930 | 56 ^o^ C |
| 1516628-1517628 | GTTTCCCCTAGGATTTCAGC | TTTTACGTCGTTATGGCAAACC | 930 | 56^o^ C |
| 1656969-1657969 | CCACCAATAAACGATCAGGC | CGTCAAGCGATGGATAAAATC | 920 | 56^o^ C |
| 1448318-1449318 | CAGCCCTAAAATCATACAGAGA | CTGGATTACTGTCAAAGGGG | 951 | 56^o^ C |

| **Gene name** | **Product size** | **Forward Primer (5′ to 3′)** | **Reverse Primer (5′ to 3′)** |  |
| --- | --- | --- | --- | --- |
| Control (16 SRNA) |  | CAACTATAGACCAGTAAGT | AGAACATAGAAGAGGTAAGT |  |
| ZMO 02740 | 102 | AAAGCTGTGCCAAATGGTTA | TGACGTCAGAAGATACATTAAGCC |  |
| ZMO 08600 | 106 | GCGCGATAGCCATCTGATTA | AACGTCATCCTTTCTGCTCC |  |
| ZMO 01385 | 106 | CATATGGCACCTGATTATGGT | TGGTTATCTCGCCGAAAATCA |  |
| ZMO 03775 | 136 | GCAGCATCTGGTGAGTTAACTC | GCAGCATCTGGTGAGTTAACTC |  |
| ZMO 03395 | 132 | GCAATAGACCTTTGTTTCGG | CATCATGTTCTTTTCTGCGG |  |
| ZMO 06525 | 106 | TTACCTCAGAGAATGTTGGC | CCGCTGGATCAAGAAAGATA |  |
| ZMO 00080 | 125 | TTTGGCGGCTATTCCTGATG | ATTCGACCCGTTTGATACCG |  |
| ZMO 00065 | 102 | AAGGGACAAAGCACAAACGA | CCGCGTGATTTTGATAAGGC |  |
| ZMO 02800 | 174 | GCTCTGATCGCTGGATTGGTCA | TCCGGTTAATTGCGCGGCTTT |  |
| ZMO 08110 | 132 | TGACGTCAGAAGATACATTAAGCC | ATGAAATGCTGCAAGAACGC |  |

**Table S2.** **List of primers used for qPCR experiment**

**Table S3. List of primers, strains and plasmids**

| **Description** | | **Remark/source** |
| --- | --- | --- |
| **Primers Pairs** |  |  |
| ZMO_RS 06525-Up500bp-F | tcaagctgcaagtGTCGACTGGTTTTCGGCGTGGGTCATTAACAAC | For up 500bp of ZMO-RS 06525 |
| ZMO_RS 06525-Up500bp-R | TCCCAAATATGTGATGAAGTGGATAAATGCCTTTTTGTGTTTTTTTGA |  |
| ZMO_RS 06525-Down500bp-F | ACTTCATCACATATTTGGGATGTAGGTTTTCGTGTTTTTTCAAGAAACA | For down 500bp of ZMO-RS 06525 |
| ZMO_RS 06525-Down500bp-R | tgcagcggccgctactagTTAACAGACACATTCCCAATACTGA |  |
| P1-Trans-ZMO1457-F | Actagtagcggccgctgcaggtcac | For gene with spacer +backbone |
| P1-Trans-ZMO1457-R | GTCGACacttgcagcttgatatagccggtaa |  |
| Terminator-F | Ctcgaacgcgccgaataagtaattcaggtttttttataaagacct | For Pmini backbone |
| Start-R | Ccagaaaatcttctgtatctacaatggctaattttattattagaatgtag |  |
| Mini-crisper-F | agatacagaagattttctGGTACCGTTCACTGC | For 136bp synthesis |
| Mini-crisper-R | cttattcggcgcgttcgaGGATC |  |
| Check-primer-F | gctcaccgtctgaattcgcggccgc | For verification |
| Check-primer-R | gcagaaaggcccacccgaaggtgagc |  |
| Knockout Final confirmation ZMO_RS 06525-F | AGTCTGAGGCTGATTTGGCA | For confirmation |
| Knockout Final confirmation ZMO_RS 06525-R | AGCTACGCCAAATATTCGATAGC |  |
| ZMO_RS02740-Up500bp-F | tgcaagtGTCGACGCCACGGAATTGGCGTTGG | For Up 500bp of ZMO_RS02740 |
| ZMO_RS02740-Up500bp-R | GCGTCAAAAAAATGATCTTTTTGAGACTCTATTTTAAGGGGGCG |  |
| ZMO_RS02740-Down500bp-F | TCATTTTTTTGACGCCGCCGCCTTTCTGCTGGTGCT | For down 500bp of ZMO_RS02740 |
| ZMO_RS02740-Down500bp-R | tgcagcggccgctactagTATAAGCCATAAAGCGTGGTTAAAATAGC |  |
| Knockout Final confirmation ZMO_RS02740-F | GACGCATTGGTATTCAGACGA | For confirmation |
| Knockout Final confirmation ZMO_RS02740-R | GTGGTTAAAATAGCCATCGC |  |
| **Strains** |  |  |
| ZM4 | ATCC31821 |  |
| ZM532 |  | In this study |
| *ZM4∆ZMO_RS02740* | Carrying plasmid pmini-T | In this study |
| *ZM4∆ZMO_RS06525* | Carrying plasmid pmini-T | In this study |
| *ZM532∆ZMO_RS06525* | Carrying plasmid pmini-T | In this study |
| *ZM532∆ZMO_RS02740* | Carrying plasmid pmini-T |  |
| **Plasmids** |  |  |
| Pmini-T-ZM4∆ZMO_RS02740 | *E. coli* replication, *Z. mobilis* replication, sgRNA expression unit, donor DNA and spe | In this study |
| Pmini-T-ZM4∆ZMO_RS06525 | *E. coli* replication, *Z. mobilis* replication, sgRNA expression unit, donor DNA and spe | In this study |
| Pmini-T-ZM532∆ZMO_RS06525 | *E. coli* replication, *Z. mobilis* replication, sgRNA expression unit, donor DNA and spe | In this study |
| Pmini-T-ZM532∆ZMO_RS02740 | *E. coli* replication, *Z. mobilis* replication, sgRNA expression unit, donor DNA and spe | In this study |

**Table S4. List of primers, strains and plasmids**

| **Description** | | **Remark/source** |
| --- | --- | --- |
| **Primers Pairs** |  |  |
| 06526-F | ATATATGGAGTAAGCAATGATTACGGGAATATTTTTTCTATTTTTTG | For amplification of gene ZMO-06525 |
| 06525-R | TCTAAGTTTATTTAAAAACTACATCCCAAATATGTGATGAAGTTTAT |  |
| Ppdc-06526-F | TTCAAGGTGTCCCGTTCCTTTTTCCCCT | For amplification of 300bp of *pdc* promoter |
| Ppdc-06526-R | ATATTCCCGTAATCATTGCTTACTCCATATATTCAAAACACTATG |  |
| Tpdc-06526-F | CATCACATATTTGGGATGTAGTTTTTAAATAAACTTAGAGCTTAAGGCG | For amplification of 300bp of *Tpdc* promoter |
| Tpdc-06526-R | gccgctactagtaGCACTGACTTCAATAATTCAGCCCTTACG |  |
| pEZ15 asp-F | AATTATTGAAGTCAGTGCtactagtagcggccgctgcagGTCACACTGGC | For amplification of backbone |
| pEZ15asp-R | AGGAACGGGACACCTTGAAGTCGACGGATCCCCGGGTACCGAG |  |
| Confirmation P and 06526-F1 | ATCCGTCGACTTCAAGGTGT | For verification |
| Confirmation p and 06526-R1 | CCAGATTTTTTGCCACTGGAC |  |
| Confirmation T and 06526-F2 | ATCTGGATAGCTTTTGCGAT | For verification |
| Confirmation T and 06526-R2 | cgctactagtaGCACTGACT |  |
| Ppdc-02740-F | TTCAAGGTGTCCCGTTCCTTTTTCCCCT | For amplification of 300bp of *pdc* promoter |
| Ppdc-02740-R | TTGAATAGAAATCCACACATTGCTTACTCCATATATTCAAAACACTATGTCT |  |
| Tpdc-02740-F | GTATCTTCTGACGTCAGTTAGTTTTTAAATAAACTTAGAGCTTAAGGCG | For amplification of 300bp of *Tpdc* promoter |
| Tpdc-02740-R | gccgctactagtaGCACTGACTTCAATAATTCAGCCCTTACG |  |
| ZMO-RS02740-F | GTTTTGAATATATGGAGTAAGCAATGTGTGGATTTCTATTCAAAAGC | For amplification of gene ZMO-02740 |
| ZMO-RS02740-R | CTTAAGCTCTAAGTTTATTTAAAAACTAACTGACGTCAGAAGATACAT |  |
| Confirmation P and 02740-F3 | GATCCGTCGACTTCAAGGTG | For verification |
| Confirmation P and 02740-R3 | AGCAAAGCGGCAACTATCG |  |
| Confirmation T and 02740-F4 | GCTTTGCTTTGCCGGATGC | For verification |
| Confirmation T and 02740-R4 | ctactagtaGCACTGACTTCA |  |
| Control-06525-F | CCGGGGATCCGTCGACATGATTACGGGAATATTT | For amplification of 1588bp fragment of ZMO-06525 |
| Control-06525-R | agcggccgctactagtaCTACATCCCAAATATG |  |
| Control-b.b-06525-F | ATCACATATTTGGGATGTAGtactagtagcggccgctgc | For backbone amplification |
| Control- b.b-06525-R | AAATATTCCCGTAATCATGTCGACGGATCCCCGGGTAC |  |
| Control Chk-P-g-06525-F | GGAAATTCGTTGAATCCTGC | For confirmation |
| Control Chk-P-g-06525-R | TAGTATATAAACGCAGAAAGGCC |  |
| Control-02740-F | ATGTGTGGATTTCTATTCAAAAGC | For amplification of 215bp fragment of ZMO-02740 |
| Control-02740-R | cagcggccgctactagtaCTAACTGACGTCAGAAGATAC |  |
| Control Chk-P-g-02740-F | CATagatctcGAGCTCGGTA | For confirmation |
| Control Chk-P-g-02740-R | cgctgcagGTCACACTGGCT |  |
| Control.b.b.02740-F | ATCTTCTGACGTCAGTTAGtactagtagcggccgctg | For backbone amplification |
| Control.b.b.02740-R | GAATAGAAATCCACACATGTCGACGGATCCCCGGGT |  |
| **Strains** |  |  |
| ZM4 | ATCC31821 |  |
| ZM532 |  | In this study |
| *ZM4∆ZMO_RS02740* | Carrying plasmid pmini-T | In this study |
| *ZM4∆ZMO_RS06525* | Carrying plasmid pmini-T | In this study |
| *ZM532∆ZMO_RS06525* | Carrying plasmid pmini-T | In this study |
| *ZM532∆ZMO_RS02740* | Carrying plasmid pmini-T | In this study |
| **Plasmids** |  |  |
| pEZ15Asp | Spectinomycin resistance marker | In this study |
| pEZ15Asp- AF1 | pEZ15Asp plasmid carrying *ZM4* gene *ZMO_RS02740* with P*pdc*  promoter and T*pdc* terminator | In this study |
| pEZ15Asp- AF2 | pEZ15Asp plasmid carrying *ZM4* gene *ZMO_RS06525* with P*pdc*  promoter and T*pdc* terminator | In this study |
| pEZ15Asp- AF3  pEZ15Asp- AF4 | pEZ15Asp plasmid carrying *ZM532* gene *ZMO_RS02740* with P*pdc*  promoter and T*pdc* terminator  pEZ15Asp plasmid carrying *ZM532* gene *ZMO_RS06525* with P*pdc*  promoter and T*pdc* terminator | In this study  In this study |
| pEZ15Asp- AF6 | pEZ15Asp plasmid carrying *ZM4* gene *ZMO_RS02740* without P*pdc*  promoter and T*pdc* terminator | In this study |
| pEZ15Asp- AF6 | pEZ15Asp plasmid carrying *ZM4* gene *ZMO_RS06525* without P*pdc*  promoter and T*pdc* terminator | In this study |
| pEZ15Asp- AF7 | pEZ15Asp plasmid carrying *ZM532* gene *ZMO_RS02740* without P*pdc*  promoter and T*pdc* terminator | In this study |
| pEZ15Asp- AF8 | pEZ15Asp plasmid carrying *ZM532* gene *ZMO_RS06525* without P*pdc*  promoter and T*pdc* terminator | In this study |

**Table S5. INDEL in re-sequence *ZM532* by comparing with previous published ten genome-shuffled mutant strain and *Z. mobilis* ZM4 (GenBank: AE008692.2)**

| **Locus** | **Type ^a^** | **Previous ^b^** | **Current ^c^** | **Status** | **Gene/Product** |
| --- | --- | --- | --- | --- | --- |
| 1002280 | D1 | - | + | Confirmed | ZMO_RS04405 |
| 1002287 | D1 | + | + | Confirmed | /ABC transporter substrate-binding protein |
| 1266074 | D28 | + | - | Not confirmed | ZMO_RS05590/hypothetical protein |
| 1266081-12266101 | D21 | - | + | Confirmed |  |
| 1517128 | D40 | - | - |  | ZMO_RS09180/ hypothetical protein |
| 1517136 | D32 | - | - |  |  |
| 1517144 | D24 | - | - |  |  |
| 1517152 | D16 | + | - | Not confirmed |  |
| 1517160 | D8 | - | - |  |  |
| 1657469-16574475 | D7 | + | + | Confirmed | ZMO_RS07255  /carbamoyl phosphate synthase large subunit |
| 1448818 | D1 | + | + | Confirmed | ZMO_RS06410- ZMO_RS06415  FUSE family protein  /DNA polymerase III subunit delta |

^a^ D: deletion followed by 1umber of base-pair. ^b^ Wang et al. [2019]. ^c^ Current study with *ZM532* strain. +/- indicate the presence/absence of variation in the genome, respectively.

**Table S6. Overview of the transcriptome sequencing dataset and quality check**

| Sample name | Raw reads | Clean reads | clean bases | Error rate (%) | Q20 (%) | Q30 (%) | GC content (%) |
| --- | --- | --- | --- | --- | --- | --- | --- |
| RM532_1 | 16137106 | 15918234 | 2.39G | 0.03 | 97.16 | 91.93 | 49.28 |
| RM532_2 | 16619608 | 16372048 | 2.46G | 0.03 | 97.01 | 91.63 | 49.14 |
| RM532_3 | 19209180 | 18895996 | 2.83G | 0.03 | 96.83 | 91.32 | 49.04 |
| RMZM4_1 | 18795158 | 18539840 | 2.78G | 0.03 | 96.59 | 90.84 | 49.18 |
| RMZM4_2 | 18225000 | 18003918 | 2.7G | 0.03 | 96.92 | 91.46 | 49.06 |
| RMZM4_3 | 14626258 | 14406612 | 2.16G | 0.03 | 96.78 | 91.25 | 49.05 |
| AFZM4_1 | 16444490 | 16218684 | 2.43G | 0.02 | 98.41 | 95.05 | 49.21 |
| AFZM4_2 | 25023044 | 24841660 | 3.73G | 0.03 | 95.91 | 89.71 | 48.9 |
| AFZM4_3 | 25196588 | 25038602 | 3.76G | 0.03 | 95.84 | 89.57 | 48.53 |
| AF532_1 | 18180102 | 18003964 | 2.7G | 0.03 | 96.76 | 91.18 | 48.52 |
| AF532_2 | 17813314 | 17621376 | 2.64G | 0.03 | 96.28 | 90.24 | 48.03 |
| AF532_3 | 14901220 | 14705550 | 2.21G | 0.03 | 97.07 | 91.75 | 48.26 |

**Table S7** Differentially Expressed Genes of ZM532 strain in rich media and media with acetic + furfural treatments

| Gene_id | readcount_AF_532 | readcount_RM_532 | log2FoldChange | pval | padj | gene_name | description |
| --- | --- | --- | --- | --- | --- | --- | --- |
| Novel00001 | 3370.950505 | 1881.837266 | 0.84101 | 0.0074707 | 0.022135 | -//- |  |
| Novel00002 | 2178.181963 | 783.2085074 | 1.4757 | 0.0004704 | 0.0022964 | -//- |  |
| Novel00004 | 741.5079123 | 75.21289252 | 3.3014 | 0.00014483 | 0.00084196 | -//- |  |
| Novel00005 | 3465.016245 | 55.97973388 | 5.9518 | 4.91E-05 | 0.00033932 | -//- |  |
| Novel00006 | 5154.56044 | 8830.680037 | -0.77668 | 0.0092392 | 0.026437 | -//- |  |
| Novel00007 | 3151.790202 | 143.0210193 | 4.4619 | 2.24E-09 | 5.88E-08 | -//- |  |
| Novel00008 | 232.6674145 | 720.2405379 | -1.6302 | 7.02E-06 | 6.64E-05 | -//- |  |
| Novel00009 | 1197.181291 | 532.2713056 | 1.1694 | 0.00039143 | 0.0019526 | -//- |  |
| Novel00011 | 2185.340066 | 7219.104705 | -1.724 | 6.39E-05 | 0.00042196 | -//- |  |
| Novel00012 | 1906.281827 | 201.2380094 | 3.2438 | 7.92E-06 | 7.39E-05 | -//- |  |
| Novel00013 | 950.0116925 | 83.42994473 | 3.5093 | 2.41E-05 | 0.00018467 | -//- |  |
| Novel00014 | 1541.886949 | 139.9684315 | 3.4615 | 2.25E-07 | 3.28E-06 | -//- |  |
| ZMO_RS00015 | 520.5587191 | 274.153898 | 0.92507 | 0.011229 | 0.030605 | -- | adenylyl-sulfate kinase |
| ZMO_RS00020 | 1920.201433 | 819.604153 | 1.2283 | 0.00012606 | 0.00074941 | -- | sulfate adenylyltransferase subunit CysN |
| ZMO_RS00025 | 1532.292207 | 465.230399 | 1.7197 | 7.50E-05 | 0.00047945 | -- | sulfate adenylyltransferase subunit 2 |
| ZMO_RS00035 | 2087.037778 | 1026.705271 | 1.0234 | 0.001051 | 0.0044795 | -- | phosphoadenosine phosphosulfate reductase |
| ZMO_RS00040 | 5051.420375 | 2489.124824 | 1.0211 | 0.00066557 | 0.003049 | -- | assimilatory sulfite reductase (NADPH) hemoprotein subunit |
| ZMO_RS00045 | 7479.52016 | 2266.158729 | 1.7227 | 0.013039 | 0.034713 | -- | assimilatory sulfite reductase (NADPH) flavoprotein subunit |
| ZMO_RS00050 | 644.3831289 | 178.4164255 | 1.8527 | 0.015962 | 0.041241 | -- | M48 family peptidase |
| ZMO_RS00065 | 272.6047713 | 544.1581517 | -0.99722 | 0.0077692 | 0.022872 | -- | non-canonical purine NTP pyrophosphatase |
| ZMO_RS00075 | 17061.12534 | 2209.905638 | 2.9487 | 0.0013756 | 0.0055662 | hrcA | HrcA family transcriptional regulator |
| ZMO_RS00080 | 8509.477776 | 1605.935849 | 2.4057 | 9.97E-05 | 0.00061645 | -- | nucleotide exchange factor GrpE |
| ZMO_RS00085 | 6063.707716 | 3035.012648 | 0.9985 | 0.018589 | 0.046334 | -- | methyltransferase domain-containing protein |
| ZMO_RS00095 | 1755.435037 | 3376.919061 | -0.94388 | 0.0031516 | 0.010822 | -- | heparinase |
| ZMO_RS00130 | 2467.625273 | 5443.523911 | -1.1414 | 0.00030669 | 0.0015915 | -- | bifunctional phosphoribosylaminoimidazolecarboxamide formyltransferase/IMP cyclohydrolase PurH |
| ZMO_RS00140 | 3107.397139 | 1024.019705 | 1.6015 | 0.01432 | 0.037687 | -- | sel1 repeat family protein |
| ZMO_RS00165 | 497.7301572 | 146.0747136 | 1.7687 | 0.0006585 | 0.0030241 | -- | DUF1491 domain-containing protein |
| ZMO_RS00170 | 1501.972307 | 295.121241 | 2.3475 | 0.0014507 | 0.005806 | -- | nitrogen regulatory protein |
| ZMO_RS00175 | 6834.070509 | 1063.533215 | 2.6839 | 5.94E-05 | 0.00039998 | -- | ribosomal subunit interface protein |
| ZMO_RS00195 | 479.0755014 | 219.0611525 | 1.1289 | 0.0028153 | 0.0099352 | -- | septum formation inhibitor Maf |
| ZMO_RS00200 | 962.2442733 | 485.8394957 | 0.98592 | 0.014452 | 0.037925 | -- | sel1 repeat family protein |
| ZMO_RS00235 | 8247.218949 | 2326.676548 | 1.8256 | 5.34E-06 | 5.16E-05 | -- | glutamine--fructose-6-phosphate aminotransferase |
| ZMO_RS00250 | 807.9767449 | 344.458804 | 1.23 | 0.00052792 | 0.0025124 | -- | glycerate kinase |
| ZMO_RS00290 | 3120.775465 | 695.7961492 | 2.1652 | 1.48E-05 | 0.0001262 | -- | glutaredoxin 2 |
| ZMO_RS00305 | 4212.507722 | 1659.047009 | 1.3443 | 1.80E-05 | 0.0001845 | -- | rRNA maturation RNase YbeY |
| ZMO_RS00320 | 4660.383292 | 2179.411312 | 1.0965 | 0.00039223 | 0.0019526 | -- | chemotaxis protein CheW |
| ZMO_RS00325 | 2273.338577 | 1176.796525 | 0.94995 | 0.0025921 | 0.0093368 | -- | response regulator |
| ZMO_RS00380 | 1093.403313 | 545.2581679 | 1.0038 | 0.0020618 | 0.0077683 | -- | NAD(P)-dependent oxidoreductase |
| ZMO_RS00395 | 412.7057445 | 870.3099657 | -1.0764 | 0.0016694 | 0.0065111 | -- | hypothetical protein |
| ZMO_RS00410 | 370.9125445 | 1007.332746 | -1.4414 | 3.83E-05 | 0.00027198 | -- | MFS transporter |
| ZMO_RS00420 | 1211.974515 | 202.4289227 | 2.5819 | 0.0012884 | 0.0052589 | -- | NAD dependent epimerase/dehydratase |
| ZMO_RS00435 | 9210.744672 | 2892.751841 | 1.6709 | 0.011852 | 0.032064 | -- | 3-isopropylmalate dehydratase large subunit |
| ZMO_RS00440 | 2546.509357 | 917.065347 | 1.4734 | 0.0037221 | 0.012477 | -- | 3-isopropylmalate dehydratase small subunit |
| ZMO_RS00445 | 795.4545485 | 1740.554329 | -1.1297 | 0.0004762 | 0.0022964 | -- | hypothetical protein |
| ZMO_RS00465 | 2220.694224 | 3775.159914 | -0.76553 | 0.014631 | 0.038231 | -- | elongation factor 3 |
| ZMO_RS00475 | 3048.772097 | 999.2240804 | 1.6093 | 7.48E-07 | 9.42E-06 | -- | aminodeoxychorismate synthase%2C component I |
| ZMO_RS00480 | 795.702493 | 274.881583 | 1.5334 | 1.80E-05 | 0.00014844 | -- | type 1 glutamine amidotransferase |
| ZMO_RS00485 | 1401.23371 | 771.8967433 | 0.86022 | 0.0089349 | 0.025686 | -- | aminotransferase |
| ZMO_RS00495 | 2880.392007 | 1279.382391 | 1.1708 | 0.00019423 | 0.0010826 | -- | hydroxylamine reductase |
| ZMO_RS00500 | 490.4706274 | 115.1409583 | 2.0908 | 0.011175 | 0.030502 | -- | Na+/H+ antiporter NhaA |
| ZMO_RS00515 | 3414.267683 | 577.6769449 | 2.5632 | 8.07E-15 | 9.89E-13 | -- | hypothetical protein |
| ZMO_RS00525 | 1918.201211 | 649.8340684 | 1.5616 | 0.0070599 | 0.021191 | -- | Cys-tRNA(Pro) deacylase |
| ZMO_RS00545 | 119.6963266 | 625.6564488 | -2.386 | 1.06E-09 | 3.35E-08 | -- | acid phosphatase |
| ZMO_RS00550 | 215.1419603 | 730.0528672 | -1.7627 | 9.98E-07 | 1.21E-05 | -- | metallophosphoesterase |
| ZMO_RS00560 | 3318.585309 | 574.363898 | 2.5305 | 0.0022611 | 0.0083742 | -- | sel1 repeat family protein |
| ZMO_RS00565 | 93.67226743 | 30.64080285 | 1.6122 | 0.016311 | 0.041848 | -- | sel1 repeat family protein |
| ZMO_RS00610 | 3480.034732 | 1806.340953 | 0.94603 | 0.0022072 | 0.008241 | -- | L-aspartate oxidase |
| ZMO_RS00625 | 675.5191271 | 1673.939924 | -1.3092 | 7.62E-05 | 0.00048426 | -- | tRNA (guanosine(46)-N7)-methyltransferase TrmB |
| ZMO_RS00645 | 3351.286646 | 8629.872134 | -1.3646 | 1.32E-05 | 0.00011429 | -- | YebC/PmpR family DNA-binding transcriptional regulator |
| ZMO_RS00650 | 385.6723432 | 884.9758253 | -1.1983 | 0.00053733 | 0.0025506 | -- | crossover junction endodeoxyribonuclease RuvC |
| ZMO_RS00690 | 876.3803177 | 1546.360986 | -0.81925 | 0.011585 | 0.031483 | -- | hypothetical protein |
| ZMO_RS00710 | 362.7410049 | 160.9914042 | 1.172 | 0.0085041 | 0.024641 | -- | hypothetical protein |
| ZMO_RS00715 | 8498.330678 | 2583.187771 | 1.718 | 1.07E-07 | 1.80E-06 | -- | NAD(P)-dependent oxidoreductase |
| ZMO_RS00735 | 1469.923993 | 548.7808409 | 1.4214 | 0.000275 | 0.0014517 | -- | cell division protein ZapA |
| ZMO_RS00740 | 1165.243856 | 134.3680943 | 3.1164 | 0.0022061 | 0.008241 | -- | nuclear transport factor 2 family protein |
| ZMO_RS00750 | 4695.109035 | 8383.036122 | -0.83631 | 0.0065247 | 0.019864 | -- | transketolase |
| ZMO_RS00760 | 8406.028826 | 2630.973876 | 1.6758 | 0.0019475 | 0.0074378 | -- | phosphoglycerate kinase |
| ZMO_RS00775 | 4310.049822 | 8089.731074 | -0.90839 | 0.0026183 | 0.0094124 | -- | adenosylhomocysteinase |
| ZMO_RS00780 | 5370.092708 | 9536.559846 | -0.82852 | 0.0079544 | 0.023342 | -- | energy-dependent translational throttle protein EttA |
| ZMO_RS00785 | 6326.854028 | 56800.28784 | -3.1663 | 2.13E-22 | 1.30E-19 | -- | hypothetical protein |
| ZMO_RS00815 | 916.5880501 | 1979.713291 | -1.1109 | 0.00038155 | 0.0019203 | -- | MurR/RpiR family transcriptional regulator |
| ZMO_RS00850 | 2808.727729 | 4991.27893 | -0.82949 | 0.0066205 | 0.020069 | -- | peptide chain release factor 2 |
| ZMO_RS00855 | 3513.053809 | 974.1418647 | 1.8505 | 2.11E-06 | 2.28E-05 | -- | LexA repressor |
| ZMO_RS00870 | 6259.577357 | 11962.58326 | -0.93439 | 0.002323 | 0.0085346 | -- | methyl-accepting chemotaxis protein |
| ZMO_RS00875 | 689.4463601 | 44.67334659 | 3.948 | 0.00026842 | 0.0014334 | -- | MerR family DNA-binding transcriptional regulator |
| ZMO_RS00900 | 8490.148921 | 1753.306049 | 2.2757 | 7.45E-13 | 4.72E-11 | -- | N-acetyltransferase |
| ZMO_RS00905 | 11198.47586 | 18748.55482 | -0.74348 | 0.012159 | 0.032799 | -- | 50S ribosomal protein L27 |
| ZMO_RS00910 | 7729.938135 | 23097.78921 | -1.5792 | 2.91E-07 | 4.11E-06 | -- | 50S ribosomal protein L21 |
| ZMO_RS00925 | 5296.781791 | 184.99941 | 4.8395 | 0.00048257 | 0.0023146 | -- | 5-formyltetrahydrofolate cyclo-ligase |
| ZMO_RS00935 | 3754.837964 | 1949.316616 | 0.94578 | 0.0029762 | 0.010355 | -- | helicase |
| ZMO_RS00940 | 2175.265782 | 6776.099775 | -1.6393 | 2.04E-07 | 2.99E-06 | -- | ferredoxin family protein |
| ZMO_RS00970 | 1003.480283 | 1712.6291 | -0.7712 | 0.014877 | 0.038789 | -- | KR domain-containing protein |
| ZMO_RS00975 | 1316.452835 | 3720.699787 | -1.4989 | 2.14E-06 | 2.29E-05 | -- | DNA polymerase I |
| ZMO_RS00980 | 558.3548316 | 1469.790631 | -1.3964 | 2.91E-05 | 0.00021713 | -- | polysaccharide biosynthesis protein |
| ZMO_RS00995 | 3892.54403 | 888.5136556 | 2.1312 | 0.00014539 | 0.00084251 | -- | hypothetical protein |
| ZMO_RS01020 | 2693.110512 | 1383.134451 | 0.96133 | 0.0028439 | 0.0099889 | -- | ATP-binding protein |
| ZMO_RS01060 | 5122.233973 | 17531.21133 | -1.7751 | 1.33E-08 | 2.80E-07 | -- | HU family DNA-binding protein |
| ZMO_RS01065 | 6176.271768 | 511.0428549 | 3.5952 | 3.34E-05 | 0.00024359 | -- | HslU--HslV peptidase proteolytic subunit |
| ZMO_RS01070 | 14558.62799 | 1808.761857 | 3.0088 | 1.81E-09 | 4.90E-08 | -- | ATP-dependent protease ATPase subunit HslU |
| ZMO_RS01075 | 6842.271965 | 11880.97678 | -0.79611 | 0.00861 | 0.024869 | -- | 50S ribosomal protein L33 |
| ZMO_RS01115 | 4324.401138 | 2059.437121 | 1.0703 | 0.00047444 | 0.0022964 | -- | DNA-binding response regulator |
| ZMO_RS01120 | 1342.207145 | 592.7934289 | 1.179 | 0.00037804 | 0.0019079 | -- | ATP-binding protein |
| ZMO_RS01130 | 494.970785 | 158.9365879 | 1.6389 | 4.09E-05 | 0.00028917 | -- | cytochrome C biogenesis protein CcmB |
| ZMO_RS01190 | 4044.437271 | 11133.54827 | -1.4609 | 1.89E-06 | 2.09E-05 | -- | S-adenosylmethionine synthase |
| ZMO_RS01205 | 569.9193855 | 1231.442595 | -1.1115 | 0.0011858 | 0.0049507 | -- | organic solvent tolerance protein OstA |
| ZMO_RS01210 | 600.9319794 | 1736.0863 | -1.5306 | 3.92E-06 | 3.93E-05 | -- | LPS export ABC transporter periplasmic protein LptC |
| ZMO_RS01215 | 2261.991523 | 4565.376133 | -1.0131 | 0.0032993 | 0.011265 | -- | ribonuclease D |
| ZMO_RS01220 | 21565.37877 | 46553.79408 | -1.1102 | 0.0013559 | 0.0054983 | -- | cold-shock protein |
| ZMO_RS01245 | 3259.531703 | 531.1896611 | 2.6174 | 1.66E-14 | 1.79E-12 | -- | SIMPL domain-containing protein |
| ZMO_RS01255 | 5033.085234 | 252.6641917 | 4.3161 | 0.00029853 | 0.0015579 | -- | DUF445 domain-containing protein |
| ZMO_RS01260 | 305.4152814 | 1254.242538 | -2.038 | 4.68E-09 | 1.10E-07 | -- | prolipoprotein diacylglyceryl transferase |
| ZMO_RS01285 | 2856.005815 | 849.060711 | 1.7501 | 4.69E-06 | 4.61E-05 | -- | hypothetical protein |
| ZMO_RS01290 | 1139.749639 | 2596.612276 | -1.1879 | 0.00028087 | 0.0014783 | -- | phosphoribosylamine--glycine ligase |
| ZMO_RS01305 | 25.86849197 | 90.62270192 | -1.8087 | 0.0060477 | 0.018766 | -- | aryl-sulfate sulfotransferase |
| ZMO_RS01310 | 121.7048536 | 449.1338013 | -1.8838 | 4.39E-06 | 4.38E-05 | -- | ferrochelatase |
| ZMO_RS01315 | 276.9658642 | 554.1313512 | -1.0005 | 0.0075303 | 0.022276 | -- | stress responsive protein |
| ZMO_RS01320 | 572.028094 | 1162.263699 | -1.0228 | 0.0022341 | 0.008308 | -- | glycosyltransferase family 1 protein |
| ZMO_RS01330 | 3052.996566 | 261.2933671 | 3.5465 | 2.93E-14 | 2.84E-12 | -- | KR domain-containing protein |
| ZMO_RS01335 | 2735.145863 | 610.1049775 | 2.1645 | 3.44E-05 | 0.00024886 | -- | pyrroline-5-carboxylate reductase |
| ZMO_RS01340 | 2740.917741 | 952.9054208 | 1.5243 | 2.09E-06 | 2.27E-05 | -- | amidohydrolase |
| ZMO_RS01345 | 1270.072532 | 333.1700245 | 1.9306 | 7.05E-05 | 0.00045753 | -- | 2-amino-thiazoline-4-carboxylic acid hydrolase |
| ZMO_RS01360 | 2838.052783 | 361.1661779 | 2.9742 | 1.23E-05 | 0.00010887 | -- | NAD(P)-dependent oxidoreductase |
| ZMO_RS01375 | 237.8819844 | 501.6243416 | -1.0764 | 0.0031368 | 0.010791 | -- | dihydrofolate reductase |
| ZMO_RS01410 | 1715.446519 | 4381.237137 | -1.3528 | 1.88E-05 | 0.00015266 | -- | elongation factor P |
| ZMO_RS01415 | 1356.515391 | 4446.738361 | -1.7128 | 9.36E-08 | 1.59E-06 | -- | inositol monophosphatase |
| ZMO_RS01455 | 3799.093858 | 7189.106338 | -0.92016 | 0.005826 | 0.018201 | -- | pyridoxal phosphate-dependent aminotransferase |
| ZMO_RS01495 | 1271.609215 | 547.3161305 | 1.2162 | 0.00040251 | 0.0019984 | -- | chromosome partitioning protein ParB |
| ZMO_RS01510 | 2473.698589 | 1485.257805 | 0.73596 | 0.01851 | 0.046199 | -- | KR domain-containing protein |
| ZMO_RS01515 | 2703.154493 | 1533.520207 | 0.8178 | 0.0084784 | 0.024605 | -- | DNA mismatch repair endonuclease MutL |
| ZMO_RS01525 | 1221.958257 | 2075.543567 | -0.76429 | 0.017785 | 0.044754 | -- | rod shape-determining protein MreC |
| ZMO_RS01550 | 9875.314655 | 791.6884157 | 3.6408 | 0.00034245 | 0.0017475 | -- | excinuclease ABC subunit B |
| ZMO_RS01600 | 1088.847796 | 7149.61719 | -2.7151 | 0.010271 | 0.028718 | -- | levansucrase |
| ZMO_RS01605 | 220.3848353 | 2915.649355 | -3.7257 | 2.95E-05 | 0.00021941 | -- | extracellular sucrase |
| ZMO_RS01615 | 41419.05173 | 6027.08518 | 2.7808 | 0.0015577 | 0.0061537 | -- | endopeptidase La |
| ZMO_RS01645 | 117.7389365 | 654.5532179 | -2.4749 | 0.0061864 | 0.019068 | -- | hypothetical protein |
| ZMO_RS01695 | 35.04954323 | 236.6503258 | -2.7553 | 5.71E-05 | 0.00038573 | -- | hypothetical protein |
| ZMO_RS01700 | 15.09624924 | 201.2663348 | -3.7368 | 7.42E-08 | 1.30E-06 | -- | hypothetical protein |
| ZMO_RS01705 | 18.5877043 | 137.9466019 | -2.8917 | 0.00028165 | 0.0014783 | -- | hypothetical protein |
| ZMO_RS01740 | 54804.1513 | 4062.129358 | 3.754 | 4.79E-05 | 0.00033312 | -- | ATP-dependent Clp protease ATP-binding subunit ClpA |
| ZMO_RS01750 | 10145.16705 | 5735.373531 | 0.82283 | 0.0074458 | 0.022099 | -- | GcrA cell cycle regulator |
| ZMO_RS01775 | 769.1492316 | 3273.196547 | -2.0894 | 1.25E-10 | 4.99E-09 | -- | MarC family protein |
| ZMO_RS01800 | 588.221418 | 256.5389854 | 1.1972 | 0.0012911 | 0.0052589 | -- | YdcF family protein |
| ZMO_RS01810 | 1164.148265 | 2635.791629 | -1.179 | 0.00019141 | 0.001072 | -- | protein TyrC |
| ZMO_RS01815 | 2990.803933 | 5454.392623 | -0.86689 | 0.0046824 | 0.014985 | -- | histidinol-phosphate aminotransferase |
| ZMO_RS01845 | 2567.52896 | 437.9258295 | 2.5516 | 0.0011966 | 0.0049732 | -- | SUF system Fe-S cluster assembly protein |
| ZMO_RS01850 | 1151.822747 | 226.5941392 | 2.3457 | 0.0069716 | 0.020961 | -- | iron-sulfur cluster assembly accessory protein |
| ZMO_RS01855 | 1922.755764 | 895.2603337 | 1.1028 | 0.00068757 | 0.0031187 | -- | pyrimidine 5'-nucleotidase |
| ZMO_RS01870 | 456.074377 | 1488.454469 | -1.7065 | 3.75E-07 | 5.18E-06 | -- | guanylate kinase |
| ZMO_RS01875 | 10573.75224 | 3647.645245 | 1.5355 | 0.00027265 | 0.0014471 | -- | glycine zipper 2TM domain-containing protein |
| ZMO_RS01895 | 1191.157579 | 500.6946188 | 1.2504 | 0.00019449 | 0.0010826 | -- | hypothetical protein |
| ZMO_RS01925 | 953.6177464 | 2087.637387 | -1.1304 | 0.00054274 | 0.0025696 | -- | MBOAT family protein |
| ZMO_RS01955 | 3393.868614 | 6982.548914 | -1.0408 | 0.00087504 | 0.003864 | -- | ribosome biogenesis GTPase Der |
| ZMO_RS01960 | 2642.445892 | 5221.866307 | -0.98269 | 0.0019839 | 0.0075456 | -- | formate--tetrahydrofolate ligase |
| ZMO_RS01965 | 739.1753177 | 1324.504028 | -0.84146 | 0.0093997 | 0.026771 | -- | (2Fe-2S)-binding protein |
| ZMO_RS01985 | 1837.10982 | 4114.848016 | -1.1634 | 0.00018391 | 0.0010363 | -- | proline--tRNA ligase |
| ZMO_RS01990 | 2979.501412 | 9174.523874 | -1.6226 | 2.54E-07 | 3.64E-06 | -- | CTP synthetase |
| ZMO_RS01995 | 1390.451912 | 3142.719428 | -1.1765 | 0.00021354 | 0.0011675 | -- | preprotein translocase subunit SecG |
| ZMO_RS02010 | 2651.839044 | 4554.868508 | -0.78042 | 0.015647 | 0.040655 | -- | anthranilate synthase component I |
| ZMO_RS02015 | 3438.757108 | 1041.238861 | 1.7236 | 4.85E-08 | 8.83E-07 | -- | hypothetical protein |
| ZMO_RS02030 | 2615.307712 | 1590.239772 | 0.71774 | 0.019517 | 0.048384 | -- | 6%2C7-dimethyl-8-ribityllumazine synthase |
| ZMO_RS02035 | 6516.012331 | 3283.541775 | 0.98874 | 0.001136 | 0.0047755 | -- | 3%2C4-dihydroxy-2-butanone-4-phosphate synthase |
| ZMO_RS02040 | 3062.429593 | 1577.751675 | 0.95681 | 0.00225 | 0.0083499 | -- | riboflavin synthase |
| ZMO_RS02085 | 871.9237222 | 321.5925628 | 1.439 | 0.0011016 | 0.0046518 | -- | MgtC/SapB family protein |
| ZMO_RS02090 | 4232.275974 | 2593.621154 | 0.70647 | 0.019589 | 0.048497 | -- | acyl-CoA dehydrogenase |
| ZMO_RS02095 | 1909.814426 | 1047.397712 | 0.86662 | 0.0060242 | 0.018725 | -- | hypothetical protein |
| ZMO_RS02100 | 1349.556436 | 752.514932 | 0.84269 | 0.0095807 | 0.02716 | -- | CoA ester lyase |
| ZMO_RS02115 | 88.52392642 | 20.91989595 | 2.0812 | 0.0040847 | 0.013447 | -- | hypothetical protein |
| ZMO_RS02120 | 2540.447113 | 925.9262988 | 1.4561 | 0.0018463 | 0.0070956 | -- | S9 family peptidase |
| ZMO_RS02135 | 3874.442294 | 10121.38215 | -1.3853 | 1.16E-05 | 0.00010312 | -- | type I glutamate--ammonia ligase |
| ZMO_RS02140 | 801.0547191 | 3347.146257 | -2.063 | 1.87E-10 | 6.86E-09 | -- | Bcr/CflA family drug resistance efflux transporter |
| ZMO_RS02150 | 1026.850567 | 563.9871148 | 0.86449 | 0.010522 | 0.029222 | -- | outer membrane protein assembly factor |
| ZMO_RS02165 | 4234.151278 | 8556.359327 | -1.0149 | 0.0010626 | 0.0045186 | -- | penicillin-binding protein |
| ZMO_RS02170 | 1151.431028 | 1990.558133 | -0.78975 | 0.011839 | 0.032064 | -- | hypothetical protein |
| ZMO_RS02175 | 178.9024415 | 435.682347 | -1.2841 | 0.0010191 | 0.0043855 | -- | GtrA family protein |
| ZMO_RS02180 | 938.2407092 | 2099.140572 | -1.1618 | 0.00026793 | 0.0014334 | -- | hypothetical protein |
| ZMO_RS02185 | 2558.835415 | 4787.012476 | -0.90364 | 0.0095552 | 0.02713 | -- | glycosyltransferase family 2 protein |
| ZMO_RS02190 | 598.7747947 | 1404.081244 | -1.2295 | 0.00082863 | 0.0036768 | -- | RNA pseudouridine synthase |
| ZMO_RS02195 | 315.2759435 | 574.9412291 | -0.8668 | 0.018407 | 0.046068 | -- | aminoacyl-tRNA hydrolase |
| ZMO_RS02220 | 1297.39934 | 632.7580541 | 1.0359 | 0.0043649 | 0.014217 | -- | acyl-CoA thioesterase |
| ZMO_RS02235 | 12261.80152 | 28071.96437 | -1.195 | 0.00010505 | 0.00064541 | -- | 30S ribosomal protein S12 |
| ZMO_RS02240 | 5461.083263 | 14882.90473 | -1.4464 | 7.26E-06 | 6.80E-05 | -- | 30S ribosomal protein S7 |
| ZMO_RS02245 | 43243.70697 | 113906.1291 | -1.3973 | 6.93E-06 | 6.60E-05 | -- | elongation factor G |
| ZMO_RS02250 | 67038.42532 | 147642.3763 | -1.139 | 0.00022413 | 0.0012217 | -- | elongation factor Tu |
| ZMO_RS02255 | 1946.2421 | 9137.388219 | -2.2311 | 2.71E-12 | 1.61E-10 | -- | 30S ribosomal protein S10 |
| ZMO_RS02260 | 13497.39025 | 45184.76781 | -1.7432 | 1.96E-08 | 4.04E-07 | -- | 50S ribosomal protein L3 |
| ZMO_RS02265 | 4773.660193 | 17399.44668 | -1.8659 | 2.45E-09 | 6.25E-08 | -- | 50S ribosomal protein L4 |
| ZMO_RS02270 | 1516.507612 | 4625.028918 | -1.6087 | 4.93E-07 | 6.66E-06 | -- | 50S ribosomal protein L23 |
| ZMO_RS02275 | 10766.6496 | 34009.74689 | -1.6594 | 8.57E-08 | 1.47E-06 | -- | 50S ribosomal protein L2 |
| ZMO_RS02280 | 595.0043202 | 1869.403662 | -1.6516 | 6.60E-07 | 8.53E-06 | -- | 30S ribosomal protein S19 |
| ZMO_RS02285 | 2234.758642 | 7519.50273 | -1.7505 | 3.33E-08 | 6.24E-07 | -- | 50S ribosomal protein L22 |
| ZMO_RS02290 | 8429.84002 | 28156.84282 | -1.7399 | 2.48E-08 | 4.85E-07 | -- | 30S ribosomal protein S3 |
| ZMO_RS02295 | 4101.513551 | 14556.92503 | -1.8275 | 5.77E-09 | 1.34E-07 | -- | 50S ribosomal protein L16 |
| ZMO_RS02300 | 72.53416614 | 249.3133439 | -1.7812 | 0.00016058 | 0.00091895 | -- | 50S ribosomal protein L29 |
| ZMO_RS02305 | 183.0349643 | 615.1518869 | -1.7488 | 3.34E-06 | 3.41E-05 | -- | 30S ribosomal protein S17 |
| ZMO_RS02310 | 2333.558169 | 9483.198561 | -2.0228 | 1.87E-07 | 2.79E-06 | -- | 50S ribosomal protein L14 |
| ZMO_RS02315 | 1458.515448 | 5842.593933 | -2.0021 | 2.99E-10 | 1.06E-08 | -- | 50S ribosomal protein L24 |
| ZMO_RS02320 | 5182.729098 | 22857.88869 | -2.1409 | 2.25E-08 | 4.60E-07 | -- | 50S ribosomal protein L5 |
| ZMO_RS02325 | 2176.169795 | 9927.923802 | -2.1897 | 2.09E-11 | 9.83E-10 | -- | 30S ribosomal protein S14 |
| ZMO_RS02330 | 1609.816331 | 9274.986231 | -2.5264 | 1.08E-09 | 3.35E-08 | -- | 30S ribosomal protein S8 |
| ZMO_RS02335 | 2272.586442 | 12754.64622 | -2.4886 | 6.64E-15 | 8.71E-13 | -- | 50S ribosomal protein L6 |
| ZMO_RS02340 | 731.2994489 | 3843.969091 | -2.3941 | 2.23E-13 | 1.53E-11 | -- | 50S ribosomal protein L18 |
| ZMO_RS02345 | 2484.393498 | 14849.79768 | -2.5795 | 4.74E-16 | 8.56E-14 | -- | 30S ribosomal protein S5 |
| ZMO_RS02350 | 135.239882 | 912.4747258 | -2.7543 | 2.24E-13 | 1.53E-11 | -- | 50S ribosomal protein L30 |
| ZMO_RS02355 | 6440.245456 | 18119.16268 | -1.4923 | 1.67E-06 | 1.86E-05 | -- | 50S ribosomal protein L15 |
| ZMO_RS02360 | 5576.73515 | 14617.33702 | -1.3902 | 8.34E-06 | 7.74E-05 | -- | preprotein translocase subunit SecY |
| ZMO_RS02365 | 1869.499571 | 5888.563563 | -1.6553 | 2.47E-07 | 3.58E-06 | -- | adenylate kinase |
| ZMO_RS02370 | 6782.31005 | 30673.77681 | -2.1772 | 3.55E-12 | 1.98E-10 | -- | 30S ribosomal protein S13 |
| ZMO_RS02375 | 2462.281901 | 14097.16448 | -2.5173 | 4.38E-15 | 6.70E-13 | -- | 30S ribosomal protein S11 |
| ZMO_RS02380 | 7421.678587 | 39743.04792 | -2.4209 | 2.81E-14 | 2.84E-12 | -- | DNA-directed RNA polymerase subunit alpha |
| ZMO_RS02385 | 4008.989451 | 17401.06266 | -2.1179 | 2.42E-11 | 1.11E-09 | -- | 50S ribosomal protein L17 |
| ZMO_RS02430 | 16505.47758 | 60484.17732 | -1.8736 | 1.81E-09 | 4.90E-08 | -- | polyribonucleotide nucleotidyltransferase |
| ZMO_RS02435 | 2228.224942 | 6957.642215 | -1.6427 | 1.82E-07 | 2.74E-06 | -- | 30S ribosomal protein S15 |
| ZMO_RS02440 | 1603.99425 | 4688.244087 | -1.5474 | 6.81E-07 | 8.62E-06 | -- | tRNA pseudouridine(55) synthase TruB |
| ZMO_RS02455 | 13749.82206 | 50951.3847 | -1.8897 | 1.12E-09 | 3.43E-08 | -- | translation initiation factor IF-2 |
| ZMO_RS02460 | 2653.258755 | 8841.160084 | -1.7365 | 2.39E-08 | 4.73E-07 | -- | DUF448 domain-containing protein |
| ZMO_RS02490 | 3616.194937 | 1356.881365 | 1.4142 | 0.008567 | 0.024784 | -- | TonB-dependent receptor |
| ZMO_RS02495 | 153.287822 | 29.76963617 | 2.3643 | 0.019644 | 0.048568 | -- | pyridoxal phosphate-dependent aminotransferase |
| ZMO_RS02500 | 830.3429891 | 1964.975552 | -1.2427 | 0.00013894 | 0.00081803 | -- | chorismate mutase |
| ZMO_RS02545 | 6628.99652 | 2693.70941 | 1.2992 | 2.13E-05 | 0.0001668 | -- | sulfonate ABC transporter permease |
| ZMO_RS02550 | 4318.422301 | 1722.507765 | 1.326 | 1.87E-05 | 0.0001526 | -- | nitrate ABC transporter ATP-binding protein |
| ZMO_RS02565 | 385.9048911 | 1233.112795 | -1.676 | 1.23E-06 | 1.45E-05 | -- | 16S rRNA (guanine(966)-N(2))-methyltransferase RsmD |
| ZMO_RS02570 | 1598.224274 | 5585.378516 | -1.8052 | 1.20E-08 | 2.56E-07 | -- | RNA-binding protein S4 |
| ZMO_RS02585 | 1528.224919 | 3118.684236 | -1.0291 | 0.0014283 | 0.0057541 | -- | bifunctional folylpolyglutamate synthase/dihydrofolate synthase |
| ZMO_RS02630 | 1063.524727 | 457.8365545 | 1.2159 | 0.00033142 | 0.0016959 | -- | iron-sulfur cluster assembly scaffold protein |
| ZMO_RS02635 | 330.1832114 | 126.9622575 | 1.3789 | 0.0012889 | 0.0052589 | -- | DUF423 domain-containing protein |
| ZMO_RS02645 | 1147.98593 | 3256.418127 | -1.5042 | 2.25E-06 | 2.39E-05 | -- | shikimate kinase |
| ZMO_RS02675 | 898.5425919 | 2099.748623 | -1.2246 | 0.00010232 | 0.00063076 | -- | flagellar motor protein MotB |
| ZMO_RS02680 | 1350.936583 | 3076.09826 | -1.1871 | 0.00013088 | 0.00077556 | -- | flagellar motor stator protein MotA |
| ZMO_RS02690 | 1950.440985 | 4365.861499 | -1.1625 | 0.00015082 | 0.00086849 | -- | flagellar hook-associated protein FlgK |
| ZMO_RS02695 | 317.4423061 | 1173.125008 | -1.8858 | 5.20E-08 | 9.37E-07 | -- | flagellar protein FlgJ |
| ZMO_RS02700 | 1221.493382 | 4491.445624 | -1.8785 | 3.99E-09 | 9.56E-08 | -- | flagellar P-ring protein |
| ZMO_RS02705 | 697.1169584 | 2306.433536 | -1.7262 | 1.80E-07 | 2.74E-06 | -- | flagellar L-ring protein |
| ZMO_RS02710 | 1416.126247 | 4444.123921 | -1.6499 | 2.91E-07 | 4.11E-06 | -- | flagellar basal-body rod protein FlgG |
| ZMO_RS02715 | 2225.057782 | 7635.312905 | -1.7788 | 2.29E-08 | 4.63E-07 | -- | flagellar basal-body rod protein FlgF |
| ZMO_RS02720 | 7290.11239 | 17782.192 | -1.2864 | 4.41E-05 | 0.00030914 | -- | flagellar hook protein FlgE |
| ZMO_RS02740 | 24.31350546 | 0.366201671 | 6.053 | 5.12E-09 | 3.79E-07 | -- | hypothetical protein |
| ZMO_RS02795 | 2778.309452 | 1046.034241 | 1.4093 | 1.29E-05 | 0.00011268 | -- | flagellin |
| ZMO_RS02825 | 1574.648741 | 2931.888012 | -0.8968 | 0.0037712 | 0.012573 | fliI | FliI/YscN family ATPase |
| ZMO_RS02830 | 248.4753143 | 472.2277006 | -0.92638 | 0.013553 | 0.035979 | -- | hypothetical protein |
| ZMO_RS02835 | 637.7656244 | 1219.751285 | -0.93549 | 0.0043893 | 0.014221 | -- | hypothetical protein |
| ZMO_RS02880 | 1070.969751 | 570.1938893 | 0.90939 | 0.0073019 | 0.021801 | -- | flagellar biosynthesis protein FlhB |
| ZMO_RS02885 | 3127.434572 | 1750.364891 | 0.83732 | 0.0083234 | 0.024307 | -- | flagellar hook protein FliD |
| ZMO_RS02905 | 1107.466832 | 3229.764691 | -1.5442 | 1.12E-06 | 1.33E-05 | -- | adenosine deaminase |
| ZMO_RS02910 | 1544.085101 | 2776.930353 | -0.84674 | 0.0071645 | 0.02147 | -- | phosphoribosyltransferase |
| ZMO_RS02930 | 125460.8507 | 16645.4114 | 2.914 | 1.94E-05 | 0.00015596 | -- | molecular chaperone DnaK |
| ZMO_RS02935 | 9299.243752 | 2481.190492 | 1.9061 | 5.11E-06 | 4.97E-05 | -- | molecular chaperone DnaJ |
| ZMO_RS02940 | 2340.102364 | 6084.177777 | -1.3785 | 1.40E-05 | 0.00012057 | -- | adenylosuccinate lyase |
| ZMO_RS02960 | 5888.423974 | 1372.719698 | 2.1008 | 3.25E-05 | 0.0002381 | -- | DUF1318 domain-containing protein |
| ZMO_RS02990 | 1786.336589 | 984.7054429 | 0.85924 | 0.011135 | 0.030483 | -- | excinuclease ABC subunit C |
| ZMO_RS03000 | 757.4539515 | 181.8209308 | 2.0586 | 3.03E-07 | 4.25E-06 | -- | hypothetical protein |
| ZMO_RS03005 | 1833.805223 | 1043.504322 | 0.8134 | 0.010877 | 0.029868 | -- | LysR family transcriptional regulator |
| ZMO_RS03025 | 703.0801114 | 355.9802269 | 0.98189 | 0.0064863 | 0.019793 | -- | type I-F CRISPR-associated endonuclease Cas1 |
| ZMO_RS03030 | 3212.472252 | 1714.582083 | 0.90583 | 0.003849 | 0.012763 | -- | type I-F CRISPR-associated helicase Cas3 |
| ZMO_RS03055 | 1891.113891 | 1082.592984 | 0.80475 | 0.011465 | 0.031203 | -- | histidine kinase |
| ZMO_RS03060 | 3050.133556 | 14196.92819 | -2.2186 | 3.28E-10 | 1.14E-08 | -- | acetolactate synthase large subunit |
| ZMO_RS03075 | 6015.291211 | 31543.36212 | -2.3906 | 3.32E-14 | 3.05E-12 | -- | glucose-fructose oxidoreductase |
| ZMO_RS03080 | 1663.614927 | 4130.654507 | -1.312 | 4.94E-05 | 0.00033989 | -- | FADH(2)-oxidizing methylenetetrahydrofolate--tRNA-(uracil(54)-C(5))-methyltransferase TrmFO |
| ZMO_RS03085 | 8597.218018 | 14885.90769 | -0.79201 | 0.010364 | 0.028935 | -- | DNA gyrase subunit A |
| ZMO_RS03090 | 13617.3007 | 996.3339256 | 3.7727 | 2.57E-12 | 1.57E-10 | -- | organic hydroperoxide resistance protein |
| ZMO_RS03095 | 12299.566 | 2940.549011 | 2.0644 | 5.28E-07 | 7.09E-06 | -- | hypothetical protein |
| ZMO_RS03105 | 666.8265327 | 1285.953705 | -0.94746 | 0.0049432 | 0.015765 | -- | hypothetical protein |
| ZMO_RS03125 | 1017.718066 | 441.7319957 | 1.2041 | 0.00044084 | 0.002177 | -- | hypothetical protein |
| ZMO_RS03135 | 4854.106499 | 12948.62098 | -1.4155 | 4.69E-06 | 4.61E-05 | -- | acyl-CoA synthetase |
| ZMO_RS03140 | 812.5692201 | 304.7443787 | 1.4149 | 0.0053102 | 0.016732 | -- | recombinase RecX |
| ZMO_RS03155 | 543.84034 | 1005.497493 | -0.88665 | 0.010809 | 0.029726 | -- | phosphoribosylglycinamide formyltransferase |
| ZMO_RS03160 | 2205.790586 | 3786.013788 | -0.77938 | 0.013878 | 0.036787 | -- | phosphoribosylformylglycinamidine cyclo-ligase |
| ZMO_RS03175 | 4659.558624 | 2747.214649 | 0.76222 | 0.011845 | 0.032064 | -- | RNA degradosome polyphosphate kinase |
| ZMO_RS03185 | 874.7294607 | 1726.721665 | -0.98113 | 0.0028178 | 0.0099352 | -- | ribonuclease D |
| ZMO_RS03190 | 3324.711467 | 9298.92134 | -1.4838 | 2.06E-06 | 2.26E-05 | -- | aspartate--tRNA(Asp/Asn) ligase |
| ZMO_RS03195 | 1001.049041 | 1774.741503 | -0.8261 | 0.010616 | 0.029369 | -- | hypothetical protein |
| ZMO_RS03240 | 6102.077183 | 13571.71317 | -1.1532 | 0.00013966 | 0.00081969 | -- | 50S ribosomal protein L11 |
| ZMO_RS03245 | 7742.72136 | 18737.58313 | -1.275 | 3.16E-05 | 0.00023337 | -- | 50S ribosomal protein L1 |
| ZMO_RS03250 | 10607.11208 | 46185.00087 | -2.1224 | 1.33E-11 | 6.45E-10 | -- | 50S ribosomal protein L10 |
| ZMO_RS03255 | 9765.954014 | 43949.10207 | -2.17 | 6.48E-12 | 3.40E-10 | -- | 50S ribosomal protein L7/L12 |
| ZMO_RS03265 | 104418.658 | 62687.60721 | 0.73613 | 0.014219 | 0.037499 | -- | DNA-directed RNA polymerase subunit beta |
| ZMO_RS03275 | 131.0380884 | 49.00902108 | 1.4189 | 0.0099234 | 0.028002 | -- | acetyltransferase |
| ZMO_RS03285 | 2857.396031 | 6644.094185 | -1.2174 | 0.00010616 | 0.00065008 | -- | acetyl-CoA carboxylase biotin carboxylase subunit |
| ZMO_RS03290 | 874.2897798 | 2236.560187 | -1.3551 | 3.44E-05 | 0.00024886 | -- | acetyl-CoA carboxylase biotin carboxyl carrier protein |
| ZMO_RS03310 | 11231.41307 | 941.9696987 | 3.5757 | 2.09E-24 | 1.92E-21 | -- | CsbD family protein |
| ZMO_RS03315 | 1728.836449 | 3054.843311 | -0.8213 | 0.0088948 | 0.02562 | -- | peptide chain release factor 3 |
| ZMO_RS03320 | 383.2018813 | 714.7947573 | -0.89942 | 0.010015 | 0.028175 | -- | transporter |
| ZMO_RS03345 | 14896.34406 | 1155.974841 | 3.6878 | 0.0019898 | 0.0075522 | -- | cysteine synthase A |
| ZMO_RS03355 | 1100.609559 | 2155.681139 | -0.96984 | 0.010128 | 0.042681 | -- | RluA family pseudouridine synthase |
| ZMO_RS03370 | 5653.109468 | 1310.151194 | 2.1093 | 1.70E-05 | 0.00014153 | -- | glutaredoxin 3 |
| ZMO_RS03375 | 5748.066536 | 1379.157429 | 2.0593 | 1.48E-10 | 5.66E-09 | -- | sterol-binding protein |
| ZMO_RS03380 | 1140.007732 | 2951.738399 | -1.3725 | 1.54E-05 | 0.00013053 | -- | tetratricopeptide repeat protein |
| ZMO_RS03395 | 1492.232065 | 878.9691677 | 0.76359 | 0.017023 | 0.043192 | -- | hydroxyacylglutathione hydrolase |
| ZMO_RS03400 | 2047.459628 | 999.3437613 | 1.0348 | 0.0011387 | 0.0047758 | -- | lactoylglutathione lyase |
| ZMO_RS03430 | 313.7935771 | 112.9270265 | 1.4744 | 0.0049262 | 0.015738 | -- | hypothetical protein |
| ZMO_RS03435 | 974.0874269 | 2037.557934 | -1.0647 | 0.00084061 | 0.003721 | -- | tRNA 2-thiouridine(34) synthase MnmA |
| ZMO_RS03440 | 815.307289 | 1776.715615 | -1.1238 | 0.00047314 | 0.0022964 | -- | magnesium transporter |
| ZMO_RS03450 | 874.1933891 | 432.0998932 | 1.0166 | 0.0028642 | 0.010041 | -- | LysR family transcriptional regulator |
| ZMO_RS03475 | 821.4265966 | 1598.65243 | -0.96065 | 0.0036751 | 0.012342 | -- | RND transporter |
| ZMO_RS03480 | 1844.364183 | 3929.365814 | -1.0912 | 0.00048842 | 0.0023365 | -- | LysR family transcriptional regulator |
| ZMO_RS03485 | 4488.596418 | 8161.159518 | -0.86251 | 0.0067476 | 0.02036 | gatB | Asp-tRNA(Asn)/Glu-tRNA(Gln) amidotransferase GatCAB subunit B |
| ZMO_RS03490 | 3648.824854 | 7366.092895 | -1.0135 | 0.0025623 | 0.0092731 | gatA | Asp-tRNA(Asn)/Glu-tRNA(Gln) amidotransferase GatCAB subunit A |
| ZMO_RS03495 | 578.0820563 | 1023.811559 | -0.8246 | 0.015908 | 0.041216 | -- | Asp-tRNA(Asn)/Glu-tRNA(Gln) amidotransferase subunit GatC |
| ZMO_RS03560 | 112.5220244 | 235.5210137 | -1.0656 | 0.01808 | 0.045373 | -- | ABC transporter permease |
| ZMO_RS03565 | 308.0290419 | 733.1925105 | -1.2511 | 0.0006701 | 0.0030545 | -- | ABC transporter ATP-binding protein/permease |
| ZMO_RS03570 | 309.7727406 | 724.8510289 | -1.2265 | 0.00064868 | 0.0029865 | -- | secretion protein HlyD |
| ZMO_RS03600 | 1072.58951 | 3470.624717 | -1.6941 | 1.18E-07 | 1.94E-06 | -- | 30S ribosomal protein S12 methylthiotransferase RimO |
| ZMO_RS03655 | 3134.821913 | 9499.617403 | -1.5995 | 3.75E-07 | 5.18E-06 | -- | phosphoribosylformylglycinamidine synthase subunit PurL |
| ZMO_RS03675 | 1073.044594 | 2092.204464 | -0.96331 | 0.0027684 | 0.0098177 | -- | ribosomal RNA small subunit methyltransferase H |
| ZMO_RS03685 | 1220.447084 | 2894.745293 | -1.246 | 8.08E-05 | 0.00050995 | -- | penicillin-binding protein 2 |
| ZMO_RS03690 | 968.2691806 | 2233.605433 | -1.2059 | 0.00020338 | 0.0011186 | -- | UDP-N-acetylmuramoyl-L-alanyl-D-glutamate--2%2C6-diaminopimelate ligase |
| ZMO_RS03695 | 1074.168105 | 2527.141463 | -1.2343 | 0.00010745 | 0.00065576 | -- | UDP-N-acetylmuramoyl-tripeptide--D-alanyl-D-alanine ligase |
| ZMO_RS03700 | 662.8615107 | 1583.442243 | -1.2563 | 0.0001213 | 0.00072583 | -- | phospho-N-acetylmuramoyl-pentapeptide-transferase |
| ZMO_RS03705 | 1079.472891 | 2827.030595 | -1.389 | 1.15E-05 | 0.00010253 | -- | UDP-N-acetylmuramoyl-L-alanine--D-glutamate ligase |
| ZMO_RS03710 | 679.5113648 | 1726.912405 | -1.3456 | 3.24E-05 | 0.0002381 | -- | putative lipid II flippase FtsW |
| ZMO_RS03715 | 1143.862496 | 2368.500637 | -1.0501 | 0.00082685 | 0.0036768 | -- | undecaprenyldiphospho-muramoylpentapeptide beta-N- acetylglucosaminyltransferase |
| ZMO_RS03720 | 1478.075598 | 3791.818136 | -1.3592 | 1.59E-05 | 0.00013319 | -- | UDP-N-acetylmuramate--L-alanine ligase |
| ZMO_RS03725 | 1117.604842 | 2368.862353 | -1.0838 | 0.00066771 | 0.0030512 | -- | UDP-N-acetylenolpyruvoylglucosamine reductase |
| ZMO_RS03730 | 991.040118 | 2203.785506 | -1.153 | 0.00026995 | 0.0014374 | -- | D-alanine--D-alanine ligase |
| ZMO_RS03735 | 631.4230755 | 1474.918719 | -1.224 | 0.00015199 | 0.00087252 | -- | cell division protein FtsQ |
| ZMO_RS03765 | 2489.635921 | 4988.139564 | -1.0026 | 0.0013913 | 0.0056174 | -- | arginine--tRNA ligase |
| ZMO_RS03775 | 5088.935461 | 12844.44891 | -1.3357 | 1.91E-05 | 0.00015493 | -- | alanine--tRNA ligase |
| ZMO_RS03805 | 737.6291152 | 1393.854646 | -0.91811 | 0.0046502 | 0.014908 | -- | tRNA preQ1(34) S-adenosylmethionine ribosyltransferase-isomerase QueA |
| ZMO_RS03810 | 398.3162351 | 114.8660992 | 1.794 | 6.45E-07 | 1.13E-05 | -- | peptidylprolyl isomerase |
| ZMO_RS03820 | 2651.868767 | 8692.425853 | -1.7127 | 6.56E-08 | 1.17E-06 | -- | polyprenyl synthetase family protein |
| ZMO_RS03825 | 471.4609634 | 1273.15527 | -1.4332 | 2.73E-05 | 0.00020669 | -- | exodeoxyribonuclease 7 small subunit |
| ZMO_RS03830 | 2414.82408 | 7784.885407 | -1.6888 | 7.22E-08 | 1.28E-06 | -- | DUF1013 domain-containing protein |
| ZMO_RS03850 | 5388.383735 | 3103.817731 | 0.79581 | 0.0081057 | 0.023748 | -- | nucleoid-associated protein%2C YbaB/EbfC family |
| ZMO_RS03855 | 569.948528 | 1326.557348 | -1.2188 | 0.00030927 | 0.0015968 | -- | dCTP deaminase |
| ZMO_RS03870 | 2440.879753 | 6169.885496 | -1.3378 | 0.00019667 | 0.0010915 | -- | cation transporter |
| ZMO_RS03875 | 1483.72706 | 4402.681859 | -1.5692 | 4.64E-07 | 6.31E-06 | -- | NAD-dependent dehydratase |
| ZMO_RS03880 | 752.0928666 | 1793.135579 | -1.2535 | 0.00012225 | 0.00072912 | -- | glycosyl transferase family 2 |
| ZMO_RS03885 | 895.339054 | 1699.747901 | -0.92481 | 0.0040831 | 0.013447 | -- | squalene synthase HpnC |
| ZMO_RS03890 | 815.3603964 | 1857.463039 | -1.1878 | 0.00020293 | 0.0011186 | -- | squalene synthase HpnD |
| ZMO_RS03895 | 798.7106109 | 2069.444071 | -1.3735 | 2.06E-05 | 0.00016204 | -- | hypothetical protein |
| ZMO_RS03900 | 991.101869 | 3796.633944 | -1.9376 | 1.21E-09 | 3.66E-08 | -- | squalene--hopene cyclase |
| ZMO_RS03910 | 4881.913806 | 13344.22907 | -1.4507 | 2.16E-06 | 2.30E-05 | -- | hopanoid biosynthesis associated radical SAM protein HpnH |
| ZMO_RS03915 | 3603.948404 | 8790.758634 | -1.2864 | 2.55E-05 | 0.00019423 | -- | 4-hydroxy-3-methylbut-2-enyl diphosphate reductase |
| ZMO_RS03945 | 2623.997135 | 21676.0757 | -3.0463 | 1.06E-20 | 3.25E-18 | -- | 30S ribosomal protein S9 |
| ZMO_RS03950 | 2893.712735 | 22467.14367 | -2.9568 | 9.03E-20 | 2.37E-17 | -- | 50S ribosomal protein L13 |
| ZMO_RS03955 | 3183.754677 | 110.9040234 | 4.8433 | 0.019679 | 0.048588 | -- | MerC domain-containing protein |
| ZMO_RS03970 | 3307.557789 | 464.0200402 | 2.8335 | 0.010039 | 0.028199 | -- | galactose-1-epimerase |
| ZMO_RS03980 | 1195.015597 | 385.2008755 | 1.6333 | 0.00030255 | 0.0015745 | -- | ATP-dependent DNA helicase |
| ZMO_RS03995 | 196.0075089 | 48.92680077 | 2.0022 | 0.0018197 | 0.0070228 | -- | hypothetical protein |
| ZMO_RS04030 | 11945.22113 | 5235.413282 | 1.1901 | 0.0062167 | 0.019104 | -- | 2-isopropylmalate synthase |
| ZMO_RS04035 | 2539.058323 | 821.4861225 | 1.628 | 0.0014994 | 0.0059491 | -- | beta-galactosidase |
| ZMO_RS04055 | 3033.077644 | 5971.069634 | -0.97721 | 0.0015083 | 0.0059715 | -- | LPS biosynthesis protein |
| ZMO_RS04070 | 2770.614014 | 5232.801921 | -0.91738 | 0.00257 | 0.0092751 | -- | sugar transporter |
| ZMO_RS04090 | 2924.639024 | 474.3291302 | 2.6243 | 0.0010968 | 0.0046423 | -- | copper-translocating P-type ATPase |
| ZMO_RS04095 | 823.7442687 | 59.96150108 | 3.7801 | 1.80E-07 | 2.74E-06 | -- | heavy metal transport/detoxification protein |
| ZMO_RS04105 | 7732.57814 | 573.6197288 | 3.7528 | 6.84E-29 | 1.26E-25 | -- | catalase |
| ZMO_RS04115 | 1385.481152 | 288.739648 | 2.2625 | 0.003783 | 0.01259 | -- | hypothetical protein |
| ZMO_RS04120 | 1541.956792 | 669.7187126 | 1.2031 | 0.00034783 | 0.00177 | -- | hypothetical protein |
| ZMO_RS04125 | 614.1773308 | 334.6028193 | 0.87621 | 0.015953 | 0.041241 | -- | ASCH domain-containing protein |
| ZMO_RS04150 | 805.719 | 382.2905954 | 1.0756 | 0.0017639 | 0.006836 | -- | leucyl/phenylalanyl-tRNA--protein transferase |
| ZMO_RS04160 | 28.60800991 | 136.5562019 | -2.255 | 0.00047163 | 0.0022964 | -- | hypothetical protein |
| ZMO_RS04180 | 2244.497636 | 1148.887747 | 0.96615 | 0.0018768 | 0.0071978 | -- | glutathione S-transferase |
| ZMO_RS04210 | 557.5068944 | 145.0949572 | 1.942 | 8.25E-05 | 0.00051879 | -- | NAD-dependent epimerase |
| ZMO_RS04220 | 2030.226119 | 785.2911552 | 1.3703 | 2.14E-05 | 0.00016683 | -- | sucrose-6-phosphate hydrolase |
| ZMO_RS04225 | 58.15547214 | 142.0186023 | -1.2881 | 0.014037 | 0.037124 | -- | tRNA-Gln |
| ZMO_RS04240 | 8597.452923 | 30759.65152 | -1.8391 | 3.53E-09 | 8.77E-08 | -- | trigger factor |
| ZMO_RS04245 | 839.7794066 | 3089.980521 | -1.8795 | 8.63E-09 | 1.91E-07 | -- | glycosyl transferase 2 protein |
| ZMO_RS04250 | 21997.09875 | 4793.304132 | 2.1982 | 0.0006424 | 0.0029725 | -- | ATP-dependent Clp protease proteolytic subunit |
| ZMO_RS04255 | 25484.18908 | 8244.312983 | 1.6281 | 7.65E-05 | 0.00048432 | -- | ATP-dependent Clp protease ATP-binding subunit ClpX |
| ZMO_RS04295 | 18086.87932 | 6158.720081 | 1.5542 | 4.11E-06 | 5.90E-05 | -- | cytochrome c |
| ZMO_RS04320 | 1156.085484 | 2100.195947 | -0.86128 | 0.0074467 | 0.022099 | emrB | MFS transporter |
| ZMO_RS04335 | 690.2421238 | 1230.684624 | -0.83429 | 0.0099971 | 0.028167 | -- | NCS2 family permease |
| ZMO_RS04340 | 650.6770537 | 1181.926196 | -0.86113 | 0.010531 | 0.029222 | -- | purine nucleoside permease |
| ZMO_RS04350 | 2738.052194 | 5251.545074 | -0.93959 | 0.0023592 | 0.008633 | -- | hopanoid biosynthesis associated glycosyl transferase HpnI |
| ZMO_RS04360 | 2615.669315 | 4682.586435 | -0.84013 | 0.0063477 | 0.019435 | -- | ChbG/HpnK family deacetylase |
| ZMO_RS04365 | 332.7602212 | 717.8631155 | -1.1092 | 0.00124 | 0.0051072 | -- | membrane protein |
| ZMO_RS04375 | 5448.084121 | 1478.926605 | 1.8812 | 0.001498 | 0.0059491 | -- | aldo/keto reductase |
| ZMO_RS04385 | 300.6721315 | 1252.919045 | -2.059 | 2.35E-09 | 6.09E-08 | -- | TonB-dependent receptor |
| ZMO_RS04415 | 795.6215853 | 1763.438486 | -1.1482 | 0.00057768 | 0.002721 | -- | 5'-nucleotidase SurE |
| ZMO_RS04420 | 1918.134208 | 4640.458574 | -1.2746 | 6.07E-05 | 0.00040521 | -- | serine--tRNA ligase |
| ZMO_RS04425 | 5597.845615 | 11133.63616 | -0.99198 | 0.001013 | 0.0043786 | -- | RNA polymerase-binding protein DksA |
| ZMO_RS04435 | 107304.8731 | 586.8183454 | 7.5146 | 1.51E-06 | 1.72E-05 | -- | Hsp20 family protein |
| ZMO_RS04445 | 12136.08269 | 5012.86224 | 1.2756 | 2.22E-05 | 0.00017108 | -- | hypothetical protein |
| ZMO_RS04460 | 880.4108302 | 265.7068223 | 1.7283 | 1.42E-06 | 1.65E-05 | -- | peptide-methionine (S)-S-oxide reductase |
| ZMO_RS04470 | 5079.377194 | 27373.27523 | -2.43 | 1.12E-14 | 1.29E-12 | -- | 5-methyltetrahydropteroyltriglutamate--homocysteine S-methyltransferase |
| ZMO_RS04500 | 796.6352264 | 1635.09314 | -1.0374 | 0.0011915 | 0.0049633 | -- | dihydropteroate synthase |
| ZMO_RS04505 | 2023.970447 | 1099.037177 | 0.88095 | 0.0053992 | 0.016954 | -- | hypothetical protein |
| ZMO_RS04510 | 2089.763443 | 1008.869537 | 1.0506 | 0.00098469 | 0.0042662 | -- | FAD-binding oxidoreductase |
| ZMO_RS04515 | 2057.876758 | 5853.338036 | -1.5081 | 1.11E-06 | 1.32E-05 | -- | multidrug transporter |
| ZMO_RS04520 | 1334.813561 | 311.3147228 | 2.1002 | 6.51E-05 | 0.00042557 | -- | DUF4186 domain-containing protein |
| ZMO_RS04530 | 277.1218397 | 64.29259924 | 2.1078 | 0.014228 | 0.037499 | -- | DNA gyrase inhibitor YacG |
| ZMO_RS04535 | 249.0371945 | 819.1851886 | -1.7178 | 1.99E-06 | 2.19E-05 | -- | hypothetical protein |
| ZMO_RS04540 | 447.4610602 | 1416.358593 | -1.6624 | 1.02E-06 | 1.22E-05 | -- | septum formation inhibitor Maf |
| ZMO_RS04545 | 148.6494973 | 615.3033478 | -2.0494 | 1.09E-07 | 1.81E-06 | -- | translation initiation factor IF-1 |
| ZMO_RS04570 | 5172.526728 | 19359.7227 | -1.9041 | 1.55E-09 | 4.44E-08 | -- | deoxyhypusine synthase |
| ZMO_RS04575 | 4567.690251 | 16555.42404 | -1.8578 | 1.62E-09 | 4.59E-08 | -- | type III PLP-dependent enzyme |
| ZMO_RS04595 | 7576.383278 | 18346.17273 | -1.2759 | 3.14E-05 | 0.00023263 | -- | ribonucleoside triphosphate reductase |
| ZMO_RS04605 | 1225.761415 | 2487.003195 | -1.0207 | 0.0012614 | 0.0051723 | -- | anaerobic ribonucleoside-triphosphate reductase activating protein |
| ZMO_RS04610 | 1852.69796 | 3274.080951 | -0.82146 | 0.007644 | 0.022576 | -- | ABC transporter ATP-binding protein |
| ZMO_RS04615 | 844.9131418 | 1829.945135 | -1.1149 | 0.00049259 | 0.0023504 | -- | ABC transporter permease |
| ZMO_RS04620 | 1315.088944 | 2399.098229 | -0.86733 | 0.0061001 | 0.018865 | -- | 23S rRNA (adenine(2503)-C(2))-methyltransferase RlmN |
| ZMO_RS04625 | 14715.90287 | 2921.051456 | 2.3328 | 1.23E-13 | 9.79E-12 | -- | class I SAM-dependent methyltransferase |
| ZMO_RS04630 | 28495.75504 | 2904.67853 | 3.2943 | 4.25E-10 | 1.45E-08 | -- | calcium-binding protein |
| ZMO_RS04635 | 3245.990699 | 7234.735689 | -1.1563 | 0.00019926 | 0.0011026 | -- | argininosuccinate synthase |
| ZMO_RS04645 | 5716.227117 | 14166.9944 | -1.3094 | 2.01E-05 | 0.00016079 | -- | ribonucleotide-diphosphate reductase subunit alpha |
| ZMO_RS04660 | 665.1303354 | 323.096044 | 1.0417 | 0.003213 | 0.010991 | -- | NUDIX hydrolase |
| ZMO_RS04665 | 694.3051393 | 389.1129953 | 0.83538 | 0.017569 | 0.044333 | -- | TPM domain-containing protein |
| ZMO_RS04670 | 1875.748285 | 1039.260141 | 0.85191 | 0.0073105 | 0.021801 | -- | LemA family protein |
| ZMO_RS04675 | 621.1521182 | 2016.863313 | -1.6991 | 1.71E-07 | 2.64E-06 | -- | large conductance mechanosensitive channel protein MscL |
| ZMO_RS04690 | 2272.705479 | 1304.144364 | 0.80131 | 0.010454 | 0.02914 | -- | phosphate ABC transporter substrate-binding protein PstS |
| ZMO_RS04725 | 873.8717284 | 1760.277987 | -1.0103 | 0.0020575 | 0.0077683 | -- | CCA tRNA nucleotidyltransferase |
| ZMO_RS04730 | 702.8067821 | 1214.140776 | -0.78874 | 0.0163 | 0.041848 | -- | N-acetyltransferase |
| ZMO_RS04735 | 792.9950862 | 1594.836977 | -1.008 | 0.0024181 | 0.0088137 | -- | dihydroneopterin aldolase |
| ZMO_RS04740 | 26725.03397 | 2560.021073 | 3.384 | 4.22E-07 | 5.79E-06 | -- | superoxide dismutase |
| ZMO_RS04745 | 339.6999012 | 112.3022147 | 1.5969 | 0.0045812 | 0.014739 | -- | phage shock protein operon transcriptional activator |
| ZMO_RS04750 | 771.9559414 | 114.8636577 | 2.7486 | 0.001976 | 0.0075308 | -- | cell envelope biogenesis protein TonB |
| ZMO_RS04755 | 2419.513975 | 215.5707692 | 3.4885 | 2.41E-05 | 0.00018467 | -- | phage shock protein PspA |
| ZMO_RS04760 | 147.3803066 | 12.00435168 | 3.6179 | 9.38E-05 | 0.00058808 | -- | envelope stress response membrane protein PspB |
| ZMO_RS04765 | 609.3088805 | 75.69624601 | 3.0089 | 0.00030944 | 0.0015968 | -- | envelope stress response membrane protein PspC |
| ZMO_RS04770 | 479.385021 | 58.1639964 | 3.043 | 0.00023051 | 0.0012528 | -- | hypothetical protein |
| ZMO_RS04805 | 5097.203655 | 9634.195422 | -0.91846 | 0.0021499 | 0.0080764 | -- | signal recognition particle protein |
| ZMO_RS04810 | 2087.78252 | 6698.085247 | -1.6818 | 1.12E-07 | 1.85E-06 | -- | 30S ribosomal protein S16 |
| ZMO_RS04815 | 658.0867871 | 2155.675468 | -1.7118 | 1.90E-07 | 2.82E-06 | -- | ribosome maturation factor RimM |
| ZMO_RS04820 | 2246.705421 | 6327.492102 | -1.4938 | 1.01E-05 | 9.13E-05 | -- | tRNA (guanosine(37)-N1)-methyltransferase TrmD |
| ZMO_RS04825 | 1796.771479 | 3614.583404 | -1.0084 | 0.0026999 | 0.0096491 | -- | 50S ribosomal protein L19 |
| ZMO_RS04835 | 382.6263041 | 740.5654945 | -0.95269 | 0.0050062 | 0.015911 | -- | hypothetical protein |
| ZMO_RS04860 | 1362.302538 | 634.1295362 | 1.1032 | 0.0012025 | 0.0049865 | -- | lytic murein transglycosylase |
| ZMO_RS04865 | 449.9414361 | 204.7907427 | 1.1356 | 0.0038244 | 0.012704 | -- | septal ring lytic transglycosylase RlpA family protein |
| ZMO_RS04910 | 6979.010478 | 2091.04412 | 1.7388 | 0.0002577 | 0.0013883 | -- | thioredoxin |
| ZMO_RS04930 | 1271.53992 | 697.5974634 | 0.86611 | 0.01027 | 0.028718 | -- | tRNA (adenosine(37)-N6)-threonylcarbamoyltransferase complex ATPase subunit type 1 TsaE |
| ZMO_RS04950 | 7734.163512 | 14950.1731 | -0.95085 | 0.0015961 | 0.0062651 | -- | Lrp/AsnC family transcriptional regulator |
| ZMO_RS04960 | 602.9729125 | 1055.607668 | -0.80791 | 0.016194 | 0.041687 | -- | MFS transporter |
| ZMO_RS04970 | 6313.532828 | 2824.543922 | 1.1604 | 0.00013622 | 0.00080461 | -- | NAD(P)/FAD-dependent oxidoreductase |
| ZMO_RS05005 | 789.866661 | 360.6601284 | 1.131 | 0.0065796 | 0.019978 | -- | MerR family transcriptional regulator |
| ZMO_RS05045 | 2128.886344 | 979.3381248 | 1.1202 | 0.00048105 | 0.0023133 | -- | hypothetical protein |
| ZMO_RS05050 | 4451.4754 | 2364.607232 | 0.91268 | 0.0030148 | 0.010449 | -- | CinA family protein |
| ZMO_RS05065 | 2355.341004 | 681.4625551 | 1.7892 | 3.04E-08 | 5.82E-07 | -- | carbonic anhydrase |
| ZMO_RS05070 | 999.7601011 | 305.2032518 | 1.7118 | 1.00E-06 | 1.21E-05 | -- | hypothetical protein |
| ZMO_RS05090 | 1637.224983 | 891.3495083 | 0.87719 | 0.0058982 | 0.018364 | -- | tRNA (adenosine(37)-N6)-dimethylallyltransferase MiaA |
| ZMO_RS05105 | 8305.542856 | 17930.8337 | -1.1103 | 0.00031665 | 0.0016294 | -- | ketol-acid reductoisomerase |
| ZMO_RS05125 | 3306.372594 | 13246.79587 | -2.0023 | 1.38E-10 | 5.38E-09 | -- | 50S ribosomal protein L31 |
| ZMO_RS05130 | 2199.974659 | 4280.722981 | -0.96037 | 0.0023201 | 0.0085346 | -- | beta-hydroxyacyl-ACP dehydratase |
| ZMO_RS05135 | 2631.710895 | 4600.483234 | -0.80578 | 0.011106 | 0.030452 | -- | molecular chaperone Skp |
| ZMO_RS05140 | 4404.279617 | 8575.515552 | -0.96132 | 0.0017481 | 0.0068037 | -- | outer membrane protein assembly factor BamA |
| ZMO_RS05170 | 2862.845796 | 4790.519648 | -0.74273 | 0.017327 | 0.043853 | -- | UMP kinase |
| ZMO_RS05175 | 6953.306002 | 16123.59916 | -1.2134 | 9.77E-05 | 0.00060656 | -- | elongation factor Ts |
| ZMO_RS05185 | 419.8811688 | 24.69595008 | 4.0876 | 0.0092151 | 0.026409 | -- | hypothetical protein |
| ZMO_RS05190 | 1650.096119 | 530.9493096 | 1.6359 | 1.28E-06 | 1.50E-05 | -- | aspartate/glutamate racemase family protein |
| ZMO_RS05195 | 1033.884129 | 1767.01279 | -0.77324 | 0.01461 | 0.038231 | -- | CDP-diacylglycerol--serine O-phosphatidyltransferase |
| ZMO_RS05215 | 653.8197758 | 290.028961 | 1.1727 | 0.00094634 | 0.0041293 | -- | phosphate regulon transcriptional regulatory protein PhoB |
| ZMO_RS05245 | 1297.551448 | 504.1642306 | 1.3638 | 4.86E-05 | 0.00033686 | -- | beta-N-acetylhexosaminidase |
| ZMO_RS05250 | 2009.011542 | 1058.867655 | 0.92396 | 0.0033585 | 0.011446 | -- | succinylarginine dihydrolase |
| ZMO_RS05270 | 2372.836077 | 1246.425275 | 0.92882 | 0.0036243 | 0.012216 | -- | DNA-binding response regulator |
| ZMO_RS05275 | 1828.937151 | 871.390178 | 1.0696 | 0.0010194 | 0.0043855 | -- | phosphoribosyl-AMP cyclohydrolase |
| ZMO_RS05280 | 1806.203201 | 1059.762741 | 0.76922 | 0.017355 | 0.043853 | -- | RNA methyltransferase |
| ZMO_RS05285 | 4749.26739 | 2017.274859 | 1.2353 | 0.00036134 | 0.0018336 | -- | bifunctional ADP-dependent NAD(P)H-hydrate dehydratase/NAD(P)H-hydrate epimerase |
| ZMO_RS05290 | 957.0548109 | 539.8603962 | 0.82602 | 0.015248 | 0.039674 | -- | N-formylglutamate amidohydrolase |
| ZMO_RS05315 | 8060.044658 | 1617.067194 | 2.3174 | 2.02E-13 | 1.53E-11 | -- | lytic transglycosylase domain-containing protein |
| ZMO_RS05320 | 3011.974744 | 422.2647649 | 2.8345 | 0.0024852 | 0.0090401 | -- | formamidopyrimidine-DNA glycosylase |
| ZMO_RS05390 | 2021.157649 | 3656.79175 | -0.8554 | 0.0078194 | 0.022983 | glyA | serine hydroxymethyltransferase |
| ZMO_RS05395 | 191.2079555 | 472.1287062 | -1.304 | 0.0010226 | 0.0043892 | -- | transcriptional regulator NrdR |
| ZMO_RS05400 | 366.2005995 | 1080.110121 | -1.5605 | 1.04E-05 | 9.38E-05 | -- | rRNA methyltransferase |
| ZMO_RS05405 | 1032.068667 | 304.6314338 | 1.7604 | 0.010718 | 0.029563 | -- | hypothetical protein |
| ZMO_RS05415 | 730.4953171 | 282.2426263 | 1.3719 | 0.00014047 | 0.00082177 | -- | nitrilase |
| ZMO_RS05430 | 4473.26011 | 8937.279287 | -0.99851 | 0.0011351 | 0.0047755 | trkD | potassium transporter Kup |
| ZMO_RS05445 | 37543.51951 | 11663.57279 | 1.6866 | 3.29E-08 | 6.23E-07 | -- | glucose-6-phosphate isomerase |
| ZMO_RS05460 | 1462.082073 | 736.7294041 | 0.98882 | 0.0025236 | 0.0091616 | -- | hypothetical protein |
| ZMO_RS05505 | 7998.512021 | 30420.96578 | -1.9273 | 6.88E-10 | 2.26E-08 | -- | 30S ribosomal protein S6 |
| ZMO_RS05510 | 153.5278736 | 693.6936979 | -2.1758 | 1.36E-08 | 2.83E-07 | -- | 30S ribosomal protein S18 |
| ZMO_RS05515 | 4674.408627 | 23882.04795 | -2.3531 | 2.09E-13 | 1.53E-11 | -- | 50S ribosomal protein L9 |
| ZMO_RS05525 | 1255.096037 | 2447.884968 | -0.96374 | 0.0026928 | 0.0096426 | -- | GTP cyclohydrolase I FolE |
| ZMO_RS05540 | 1162.746276 | 528.2013781 | 1.1384 | 0.005257 | 0.016593 | -- | glycosyltransferase family 1 protein |
| ZMO_RS05555 | 5597.606706 | 1390.024884 | 2.0097 | 0.0057637 | 0.018037 | -- | transcriptional repressor |
| ZMO_RS05560 | 88038.26026 | 7406.404139 | 3.5713 | 1.24E-07 | 1.99E-06 | -- | zinc-dependent alcohol dehydrogenase |
| ZMO_RS05570 | 1037.655511 | 258.7589911 | 2.0036 | 1.05E-08 | 2.29E-07 | -- | 2,3-diphosphoglycerate-dependent phosphoglycerate mutase |
| ZMO_RS05585 | 401.3290484 | 1211.424078 | -1.5938 | 2.62E-06 | 2.75E-05 | -- | 50S ribosomal protein L36 |
| ZMO_RS05605 | 267.8885161 | 814.0256933 | -1.6034 | 1.13E-05 | 0.00010148 | -- | DUF3617 domain-containing protein |
| ZMO_RS05660 | 350.4398748 | 143.0186718 | 1.293 | 0.001464 | 0.0058466 | -- | mandelate racemase/muconate lactonizing enzyme family protein |
| ZMO_RS05670 | 4811.432893 | 9468.113119 | -0.97661 | 0.0016322 | 0.0063793 | -- | GMP synthase (glutamine-hydrolyzing) |
| ZMO_RS05675 | 1030.450954 | 2984.374624 | -1.5342 | 1.36E-06 | 1.58E-05 | -- | hypothetical protein |
| ZMO_RS05690 | 4411.654369 | 781.3540273 | 2.4973 | 0.0074443 | 0.022099 | -- | uroporphyrinogen-III C-methyltransferase |
| ZMO_RS05720 | 868.0204837 | 1468.00367 | -0.75805 | 0.016638 | 0.042391 | -- | endolytic transglycosylase MltG |
| ZMO_RS05725 | 8129.577439 | 15246.28 | -0.90721 | 0.0025644 | 0.0092731 | -- | beta-ketoacyl-[acyl-carrier-protein] synthase II |
| ZMO_RS05735 | 522.0210243 | 2267.339705 | -2.1188 | 7.75E-11 | 3.23E-09 | -- | LacI family DNA-binding transcriptional regulator |
| ZMO_RS05745 | 1107.559484 | 647.1391126 | 0.77524 | 0.016604 | 0.042391 | -- | GMC family oxidoreductase |
| ZMO_RS05750 | 742.6829789 | 403.9068908 | 0.87872 | 0.010059 | 0.02821 | -- | sorbitol dehydrogenase |
| ZMO_RS05765 | 604.6628021 | 103.5014352 | 2.5465 | 6.38E-05 | 0.00042196 | -- | GlsB/YeaQ/YmgE family stress response membrane protein |
| ZMO_RS05790 | 3694.937021 | 2195.990293 | 0.75068 | 0.012952 | 0.034532 | -- | ComF family protein |
| ZMO_RS05835 | 2905.018754 | 1399.836382 | 1.0533 | 0.00089301 | 0.0039152 | -- | pyrroline-5-carboxylate reductase |
| ZMO_RS05840 | 2248.295572 | 1302.286431 | 0.78778 | 0.016982 | 0.043149 | -- | hypothetical protein |
| ZMO_RS05845 | 2206.85126 | 791.8965275 | 1.4786 | 0.00064743 | 0.0029865 | -- | hypothetical protein |
| ZMO_RS05855 | 751.1799971 | 1899.56722 | -1.3384 | 5.33E-05 | 0.00036506 | -- | fumarate hydratase |
| ZMO_RS05865 | 5938.169058 | 2853.265585 | 1.0574 | 0.00069436 | 0.003134 | -- | leucyl aminopeptidase |
| ZMO_RS05870 | 1967.043265 | 3539.849728 | -0.84766 | 0.0058659 | 0.018295 | -- | LPS-assembly protein LptD |
| ZMO_RS05895 | 497.1929854 | 932.2978187 | -0.90698 | 0.006219 | 0.019104 | -- | polysaccharide biosynthesis protein |
| ZMO_RS05900 | 482.7359275 | 1023.766885 | -1.0846 | 0.0011593 | 0.0048512 | -- | hypothetical protein |
| ZMO_RS05915 | 693.6913355 | 1846.871097 | -1.4127 | 1.55E-05 | 0.00013053 | -- | RNA methyltransferase |
| ZMO_RS05920 | 6181.86589 | 15226.45226 | -1.3005 | 2.57E-05 | 0.00019545 | -- | IMP dehydrogenase |
| ZMO_RS05975 | 9561.177552 | 2683.975026 | 1.8328 | 2.75E-09 | 6.92E-08 | -- | NAD(P)H:quinone oxidoreductase |
| ZMO_RS05985 | 638.6320387 | 206.7120462 | 1.6274 | 0.00060593 | 0.0028323 | -- | hypothetical protein |
| ZMO_RS05990 | 309.1411468 | 128.1103001 | 1.2709 | 0.0027345 | 0.0097263 | -- | LysE family translocator |
| ZMO_RS05995 | 819.3793657 | 85.44801374 | 3.2614 | 0.00042168 | 0.0020879 | -- | MFS transporter |
| ZMO_RS06000 | 2184.305688 | 475.0801732 | 2.2009 | 4.64E-11 | 1.98E-09 | -- | zinc metalloprotease |
| ZMO_RS06010 | 11801.03639 | 497.8685825 | 4.567 | 2.05E-05 | 0.00016204 | -- | aldo/keto reductase |
| ZMO_RS06015 | 2167.026695 | 5432.463695 | -1.3259 | 2.91E-05 | 0.00021713 | -- | aminopeptidase N |
| ZMO_RS06020 | 1156.608037 | 2238.108834 | -0.95238 | 0.0039285 | 0.01298 | -- | EamA/RhaT family transporter |
| ZMO_RS06035 | 1879.125958 | 922.5437547 | 1.0264 | 0.0017636 | 0.006836 | -- | competence/damage-inducible protein A |
| ZMO_RS06045 | 31942.57216 | 4403.431251 | 2.8588 | 1.39E-05 | 0.00011973 | -- | BAX inhibitor (BI)-1/YccA family protein |
| ZMO_RS06055 | 20217.43015 | 91442.9082 | -2.1773 | 3.02E-12 | 1.73E-10 | -- | flagellar motor protein MotB |
| ZMO_RS06075 | 10425.31816 | 23800.07331 | -1.1909 | 7.28E-05 | 0.00046947 | -- | 30S ribosomal protein S20 |
| ZMO_RS06090 | 7212.72413 | 17223.82666 | -1.2558 | 4.45E-05 | 0.00031058 | -- | hypothetical protein |
| ZMO_RS06110 | 7834.569486 | 29143.8984 | -1.8953 | 1.26E-09 | 3.75E-08 | -- | 30S ribosomal protein S4 |
| ZMO_RS06130 | 692.2085332 | 107.7182962 | 2.6839 | 6.98E-12 | 3.56E-10 | -- | short-chain dehydrogenase |
| ZMO_RS06135 | 32426.40635 | 1386.882898 | 4.5473 | 4.13E-14 | 3.61E-12 | -- | hypothetical protein |
| ZMO_RS06165 | 234.597004 | 57.3596711 | 2.0321 | 2.01E-05 | 0.00016079 | -- | AraC family transcriptional regulator |
| ZMO_RS06175 | 178.2195893 | 80.30430505 | 1.1501 | 0.020087 | 0.04953 | -- | aspartate aminotransferase family protein |
| ZMO_RS06180 | 1391.419192 | 3541.073242 | -1.3476 | 2.17E-05 | 0.00016783 | -- | GTPase Era |
| ZMO_RS06190 | 1019.866455 | 517.5592372 | 0.97858 | 0.0042585 | 0.01395 | -- | hypothetical protein |
| ZMO_RS06195 | 5071.28846 | 1704.555121 | 1.573 | 1.63E-05 | 0.00013595 | -- | DNA-binding response regulator |
| ZMO_RS06235 | 1298.685052 | 602.2699194 | 1.1086 | 0.00090653 | 0.003965 | -- | N-formylglutamate amidohydrolase |
| ZMO_RS06240 | 12017.57561 | 5962.890896 | 1.0111 | 0.00075699 | 0.0033834 | -- | hypothetical protein |
| ZMO_RS06255 | 747.3515982 | 1544.14542 | -1.0469 | 0.0015742 | 0.0062054 | -- | hypothetical protein |
| ZMO_RS06260 | 2082.498486 | 622.1131567 | 1.7431 | 1.36E-07 | 2.17E-06 | -- | exodeoxyribonuclease III |
| ZMO_RS06265 | 1626.732653 | 398.0157787 | 2.0311 | 3.23E-06 | 3.34E-05 | -- | iron-sulfur cluster assembly accessory protein |
| ZMO_RS06310 | 8350.045026 | 940.7557676 | 3.1499 | 5.46E-22 | 2.51E-19 | -- | bacterioferritin |
| ZMO_RS06315 | 3257.747375 | 5438.269399 | -0.73927 | 0.014886 | 0.038789 | -- | transcriptional repressor |
| ZMO_RS06320 | 5345.715887 | 12158.87659 | -1.1856 | 0.00011316 | 0.00068155 | -- | MucR family transcriptional regulator |
| ZMO_RS06335 | 4584.611282 | 950.7626838 | 2.2696 | 0.012368 | 0.033315 | -- | NifU family protein |
| ZMO_RS06345 | 14177.34437 | 40987.9295 | -1.5316 | 8.63E-07 | 1.08E-05 | -- | ATP-dependent helicase |
| ZMO_RS06350 | 841.3194033 | 422.4292235 | 0.99394 | 0.004262 | 0.01395 | -- | 4-hydroxybenzoate octaprenyltransferase |
| ZMO_RS06355 | 628.1683801 | 1212.375049 | -0.94861 | 0.003888 | 0.012869 | -- | 5-(carboxyamino)imidazole ribonucleotide mutase |
| ZMO_RS06360 | 1603.891978 | 3016.096785 | -0.91111 | 0.0043264 | 0.014117 | -- | 5-(carboxyamino)imidazole ribonucleotide synthase |
| ZMO_RS06375 | 124997.1802 | 3973.563022 | 4.9753 | 4.31E-08 | 7.91E-07 | -- | ATP-dependent chaperone ClpB |
| ZMO_RS06385 | 1638.571953 | 470.1339261 | 1.8013 | 1.22E-07 | 1.98E-06 | -- | DNA repair protein RadC |
| ZMO_RS06390 | 810.5847478 | 456.2065873 | 0.82928 | 0.01923 | 0.047802 | -- | diacylglycerol kinase |
| ZMO_RS06395 | 3750.199837 | 7869.030495 | -1.0692 | 0.0027291 | 0.0097263 | -- | TolC family protein |
| ZMO_RS06410 | 671.0365391 | 1531.72915 | -1.1907 | 0.00038708 | 0.0019375 | -- | FUSC family protein |
| ZMO_RS06425 | 4603.446451 | 9402.253127 | -1.0303 | 0.00096266 | 0.0041806 | -- | leucine--tRNA ligase |
| ZMO_RS06435 | 1113.515109 | 341.8870524 | 1.7035 | 8.82E-07 | 1.10E-05 | -- | LysE family translocator |
| ZMO_RS06440 | 349.5245802 | 163.3312275 | 1.0976 | 0.0061105 | 0.018865 | -- | Lrp/AsnC family transcriptional regulator |
| ZMO_RS06455 | 1818.698661 | 3885.790992 | -1.0953 | 0.00063004 | 0.0029375 | -- | FAD-binding oxidoreductase |
| ZMO_RS06470 | 2173.141799 | 7272.610011 | -1.7427 | 3.89E-08 | 7.21E-07 | -- | glycine--tRNA ligase subunit beta |
| ZMO_RS06475 | 1554.24574 | 3736.088942 | -1.2653 | 6.06E-05 | 0.00040521 | -- | glycine--tRNA ligase subunit alpha |
| ZMO_RS06480 | 379.9920626 | 199.4383608 | 0.93003 | 0.017622 | 0.044407 | -- | TraB/GumN family protein |
| ZMO_RS06495 | 1448.423179 | 163.7899308 | 3.1446 | 0.00095365 | 0.0041513 | -- | mannitol dehydrogenase family protein |
| ZMO_RS06500 | 1295.015276 | 551.8327311 | 1.2307 | 0.00025648 | 0.0013857 | -- | deoxyribodipyrimidine photo-lyase |
| ZMO_RS06505 | 535.1043675 | 1711.780797 | -1.6776 | 5.61E-07 | 7.41E-06 | -- | purine nucleoside permease |
| ZMO_RS06510 | 765.7302682 | 1864.837927 | -1.2841 | 7.07E-05 | 0.00045753 | -- | MFS transporter |
| ZMO_RS06525 | 585.3990883 | 3032.496775 | -2.373 | 5.59E-05 | 0.00037902 | -- | MFS transporter |
| ZMO_RS06530 | 553.2393239 | 1135.87906 | -1.0378 | 0.0056409 | 0.017683 | -- | hypothetical protein |
| ZMO_RS06540 | 1780.164969 | 547.6728455 | 1.7006 | 0.012739 | 0.034114 | -- | sulfurtransferase |
| ZMO_RS06555 | 993.2380268 | 222.2628712 | 2.1599 | 0.00060548 | 0.0028323 | -- | TonB-dependent receptor |
| ZMO_RS06560 | 233.6504633 | 75.20787346 | 1.6354 | 0.00047628 | 0.0022964 | -- | hypothetical protein |
| ZMO_RS06565 | 492.7160981 | 1687.59765 | -1.7761 | 8.33E-08 | 1.44E-06 | -- | membrane protein |
| ZMO_RS06570 | 4395.52612 | 934.2533539 | 2.2342 | 0.00045418 | 0.0022368 | -- | alpha/beta hydrolase |
| ZMO_RS06580 | 3640.471269 | 7507.590156 | -1.0442 | 0.0068609 | 0.020662 | -- | hypothetical protein |
| ZMO_RS06590 | 11446.34609 | 30505.36737 | -1.4142 | 3.46E-05 | 0.00024938 | -- | translational GTPase TypA |
| ZMO_RS06595 | 1460.144567 | 608.0205255 | 1.2639 | 0.00088906 | 0.0039151 | -- | hypothetical protein |
| ZMO_RS06645 | 2683.963039 | 795.822562 | 1.7538 | 0.019347 | 0.048027 | -- | helicase UvrD |
| ZMO_RS06650 | 4361.923141 | 301.4038396 | 3.8552 | 0.00018201 | 0.0010288 | -- | HD domain-containing protein |
| ZMO_RS06675 | 12494.10383 | 27953.89556 | -1.1618 | 9.50E-05 | 0.00059355 | -- | membrane protein |
| ZMO_RS06680 | 13051.91202 | 34453.81762 | -1.4004 | 4.74E-06 | 4.63E-05 | -- | porin family protein |
| ZMO_RS06715 | 483.5569173 | 1433.917842 | -1.5682 | 5.95E-06 | 5.69E-05 | -- | histidine triad nucleotide-binding protein |
| ZMO_RS06720 | 472.8643186 | 857.5615787 | -0.85881 | 0.015715 | 0.040775 | -- | phosphoribosyl-ATP pyrophosphatase |
| ZMO_RS06725 | 620.7263476 | 1348.269117 | -1.1191 | 0.0010368 | 0.0044397 | -- | imidazole glycerol phosphate synthase cyclase subunit |
| ZMO_RS06735 | 720.4620352 | 1490.722794 | -1.049 | 0.0094393 | 0.026842 | -- | imidazole glycerol phosphate synthase subunit HisH |
| ZMO_RS06755 | 1904.814672 | 834.9644896 | 1.1899 | 0.00025601 | 0.0013857 | -- | hypothetical protein |
| ZMO_RS06760 | 1328.599526 | 5501.875162 | -2.05 | 1.76E-10 | 6.61E-09 | -- | inorganic diphosphatase |
| ZMO_RS06765 | 1908.561153 | 3691.108438 | -0.95157 | 0.002229 | 0.0083055 | -- | histidine--tRNA ligase |
| ZMO_RS06770 | 1298.609522 | 3024.533415 | -1.2197 | 0.0001173 | 0.00070417 | -- | peptide chain release factor 1 |
| ZMO_RS06775 | 620.531572 | 1242.393016 | -1.0015 | 0.0029952 | 0.010401 | -- | peptide chain release factor N(5)-glutamine methyltransferase |
| ZMO_RS06780 | 6583.182976 | 11388.88415 | -0.79077 | 0.0092719 | 0.026489 | -- | DUF4167 domain-containing protein |
| ZMO_RS06790 | 4324.634028 | 11184.7089 | -1.3709 | 1.28E-05 | 0.00011267 | -- | phenylalanine--tRNA ligase subunit beta |
| ZMO_RS06795 | 2706.25742 | 6504.721205 | -1.2652 | 6.44E-05 | 0.00042248 | -- | phenylalanine--tRNA ligase subunit alpha |
| ZMO_RS06800 | 4449.776476 | 16585.06362 | -1.8981 | 1.30E-09 | 3.79E-08 | -- | 50S ribosomal protein L20 |
| ZMO_RS06805 | 875.3372201 | 4016.182336 | -2.1979 | 2.85E-11 | 1.25E-09 | -- | histidinol phosphate phosphatase |
| ZMO_RS06810 | 3101.569889 | 12136.7325 | -1.9683 | 5.37E-10 | 1.79E-08 | -- | ribose-phosphate pyrophosphokinase |
| ZMO_RS06815 | 1148.714813 | 1953.634888 | -0.76614 | 0.017949 | 0.045106 | -- | DUF2093 domain-containing protein |
| ZMO_RS06835 | 646.0641339 | 1488.184351 | -1.2038 | 0.0002903 | 0.0015193 | -- | RND transporter |
| ZMO_RS06845 | 924.555406 | 1659.604611 | -0.84401 | 0.0083362 | 0.024307 | -- | acriflavin resistance protein |
| ZMO_RS06860 | 667.5294943 | 1297.181716 | -0.95848 | 0.0034734 | 0.011794 | -- | phosphoribosylformylglycinamidine synthase subunit PurS |
| ZMO_RS06865 | 2454.316483 | 4508.767757 | -0.87741 | 0.0050353 | 0.015976 | -- | phosphoribosylformylglycinamidine synthase subunit PurQ |
| ZMO_RS06870 | 2983.8348 | 343.784555 | 3.1176 | 4.01E-09 | 9.56E-08 | -- | hypothetical protein |
| ZMO_RS06905 | 3321.379491 | 1768.641941 | 0.90914 | 0.0037332 | 0.012492 | -- | cobaltochelatase subunit CobT |
| ZMO_RS06910 | 2744.137437 | 1392.287065 | 0.9789 | 0.0015888 | 0.0062497 | -- | cobaltochelatase subunit CobS |
| ZMO_RS06915 | 1459.475702 | 789.2265797 | 0.88694 | 0.018503 | 0.046199 | -- | J domain-containing protein |
| ZMO_RS06920 | 323.6031554 | 610.3732441 | -0.91547 | 0.010572 | 0.029293 | -- | TetR/AcrR family transcriptional regulator |
| ZMO_RS06925 | 1490.692887 | 2926.513125 | -0.9732 | 0.0022805 | 0.0084124 | -- | squalene--hopene cyclase |
| ZMO_RS06930 | 1521.072056 | 314.6498182 | 2.2733 | 0.0023839 | 0.0087061 | -- | BolA family transcriptional regulator |
| ZMO_RS06945 | 74.72115857 | 25.77695685 | 1.5354 | 0.00053327 | 0.0027716 | -- | ATP phosphoribosyltransferase |
| ZMO_RS06975 | 453.3013831 | 1231.257841 | -1.4416 | 2.03E-05 | 0.00016123 | -- | RNA pyrophosphohydrolase |
| ZMO_RS06980 | 473.2892996 | 1383.144796 | -1.5472 | 3.91E-06 | 3.93E-05 | -- | DUF481 domain-containing protein |
| ZMO_RS06995 | 1038.255135 | 260.0813071 | 1.9971 | 0.0028724 | 0.010051 | -- | YnfA family protein |
| ZMO_RS07000 | 218.612763 | 429.5227984 | -0.97436 | 0.0099139 | 0.028002 | -- | aminoglycoside phosphotransferase |
| ZMO_RS07010 | 2198.28778 | 815.772892 | 1.4301 | 0.00017836 | 0.0010113 | -- | pyruvate formate-lyase 1-activating enzyme |
| ZMO_RS07015 | 24086.90528 | 9041.049062 | 1.4137 | 2.79E-06 | 2.90E-05 | -- | formate C-acetyltransferase |
| ZMO_RS07025 | 4735.446489 | 8010.742202 | -0.75844 | 0.014625 | 0.038231 | -- | cytochrome ubiquinol oxidase subunit I |
| ZMO_RS07050 | 3695.368303 | 259.3480597 | 3.8328 | 6.10E-19 | 1.40E-16 | -- | NAD(P)-dependent oxidoreductase |
| ZMO_RS07055 | 165.1805127 | 57.96866123 | 1.5107 | 0.002836 | 0.0099803 | -- | MFS transporter |
| ZMO_RS07060 | 156.1324562 | 53.46679322 | 1.5461 | 0.00318 | 0.010899 | -- | 3-oxoacyl-ACP reductase |
| ZMO_RS07100 | 11789.00307 | 3239.799306 | 1.8635 | 2.88E-05 | 0.00021649 | -- | antibiotic biosynthesis monooxygenase |
| ZMO_RS07110 | 7787.928799 | 14022.77728 | -0.84846 | 0.0053269 | 0.016756 | -- | DNA topoisomerase (ATP-hydrolyzing) subunit B |
| ZMO_RS07115 | 323.6333436 | 155.1337059 | 1.0608 | 0.0083764 | 0.024386 | -- | DNA replication and repair protein RecF |
| ZMO_RS07120 | 2232.882836 | 611.5901708 | 1.8683 | 7.42E-09 | 1.68E-07 | -- | bacterioferritin |
| ZMO_RS07125 | 332.6048142 | 72.99480906 | 2.1879 | 6.74E-07 | 8.59E-06 | -- | hypothetical protein |
| ZMO_RS07130 | 13737.34978 | 711.9429148 | 4.2702 | 2.39E-08 | 4.73E-07 | -- | excinuclease ABC subunit A |
| ZMO_RS07135 | 3374.471659 | 1433.078862 | 1.2355 | 0.0001448 | 0.00084196 | -- | toll/interleukin-1 receptor domain-containing protein |
| ZMO_RS07165 | 147749.3126 | 60729.25041 | 1.2827 | 1.86E-05 | 0.0001526 | -- | alcohol dehydrogenase |
| ZMO_RS07175 | 3872.240604 | 6584.206189 | -0.76584 | 0.014387 | 0.037809 | -- | hopanoid biosynthesis-associated RND transporter HpnN |
| ZMO_RS07190 | 1603.313119 | 877.1778718 | 0.87011 | 0.014045 | 0.037124 | -- | peptidase |
| ZMO_RS07195 | 1334.684147 | 652.8910946 | 1.0316 | 0.012711 | 0.034087 | -- | TIGR01244 family phosphatase |
| ZMO_RS07200 | 412.814281 | 1149.42491 | -1.4773 | 1.49E-05 | 0.00012654 | -- | alkaline phosphatase family protein |
| ZMO_RS07205 | 3649.860446 | 6752.381561 | -0.88756 | 0.004387 | 0.014221 | -- | pyruvate dehydrogenase E1 component subunit beta |
| ZMO_RS07285 | 394.9366653 | 720.9183929 | -0.86821 | 0.012411 | 0.033381 | -- | ribonuclease P protein component |
| ZMO_RS07290 | 3023.971221 | 7517.520092 | -1.3138 | 3.53E-05 | 0.00025303 | -- | membrane protein insertase YidC |
| ZMO_RS07295 | 371.3709075 | 1003.580677 | -1.4342 | 0.0012558 | 0.0051609 | -- | GTP-binding protein |
| ZMO_RS07325 | 284.8917618 | 1430.363236 | -2.3279 | 2.56E-11 | 1.14E-09 | -- | hypothetical protein |
| ZMO_RS07335 | 1493.866589 | 722.3504046 | 1.0483 | 0.0096043 | 0.027185 | -- | transglycosylase |
| ZMO_RS07340 | 677.4924205 | 161.2379982 | 2.071 | 0.0067496 | 0.02036 | -- | hypothetical protein |
| ZMO_RS07345 | 13046.49566 | 3233.359105 | 2.0126 | 0.0012373 | 0.0051072 | -- | Tim44 domain-containing protein |
| ZMO_RS07400 | 190.5488848 | 641.0447554 | -1.7503 | 3.30E-06 | 3.39E-05 | -- | uracil-DNA glycosylase |
| ZMO_RS07405 | 5130.721546 | 9774.274595 | -0.92983 | 0.0035368 | 0.011965 | -- | gluconolactonase |
| ZMO_RS07420 | 2484.783244 | 4577.257976 | -0.88136 | 0.0046312 | 0.014873 | -- | 2-nitropropane dioxygenase |
| ZMO_RS07425 | 4304.293631 | 7891.286545 | -0.87448 | 0.0052302 | 0.016537 | -- | aspartate kinase |
| ZMO_RS07430 | 1473.712899 | 536.1329649 | 1.4588 | 8.68E-06 | 7.97E-05 | -- | bifunctional 2-polyprenyl-6-hydroxyphenol methylase/3-demethylubiquinol 3-O-methyltransferase UbiG |
| ZMO_RS07450 | 2046.771266 | 4806.851321 | -1.2317 | 0.0028107 | 0.016666 | -- | tRNA lysidine(34) synthetase TilS |
| ZMO_RS07480 | 996.4177722 | 2609.264338 | -1.3888 | 1.30E-05 | 0.00011355 | -- | 23S rRNA (pseudouridine(1915)-N(3))-methyltransferase RlmH |
| ZMO_RS07495 | 1683.81458 | 863.4970065 | 0.96347 | 0.0029282 | 0.010207 | -- | disulfide bond formation protein B |
| ZMO_RS07520 | 3669.673589 | 2125.536907 | 0.78782 | 0.0084498 | 0.02456 | -- | aldo/keto reductase |
| ZMO_RS07525 | 4619.257959 | 2095.201985 | 1.1406 | 0.00017325 | 0.00098534 | -- | protein disulfide-isomerase |
| ZMO_RS07545 | 871.0632926 | 181.8640743 | 2.2599 | 0.0044253 | 0.014312 | -- | LOG family protein |
| ZMO_RS07570 | 1753.914426 | 7062.777606 | -2.0097 | 2.90E-10 | 1.04E-08 | -- | phosphoserine transaminase |
| ZMO_RS07575 | 1058.228128 | 6908.896283 | -2.7068 | 1.48E-16 | 3.02E-14 | -- | phosphoglycerate dehydrogenase |
| ZMO_RS07580 | 2803.417691 | 6463.11121 | -1.205 | 0.000113 | 0.00068155 | -- | ATP phosphoribosyltransferase regulatory subunit |
| ZMO_RS07585 | 3023.483447 | 8661.109689 | -1.5183 | 1.66E-06 | 1.86E-05 | -- | adenylosuccinate synthetase |
| ZMO_RS07590 | 1430.803443 | 824.1446376 | 0.79586 | 0.016693 | 0.042472 | -- | folate-binding protein |
| ZMO_RS07595 | 2960.265586 | 1606.709263 | 0.88162 | 0.0042678 | 0.01395 | -- | dihydroorotase |
| ZMO_RS07600 | 3584.532655 | 790.932235 | 2.1802 | 0.0030793 | 0.010613 | -- | molecular chaperone DnaJ |
| ZMO_RS07620 | 7692.152075 | 12918.28108 | -0.74795 | 0.012794 | 0.034209 | -- | TonB-dependent receptor |
| ZMO_RS07630 | 2164.52159 | 145.2933037 | 3.897 | 9.97E-14 | 8.32E-12 | -- | zinc-binding alcohol dehydrogenase family protein |
| ZMO_RS07635 | 2263.565294 | 88.1712438 | 4.6821 | 0.0026604 | 0.0095452 | -- | transcriptional regulator |
| ZMO_RS07640 | 1673.574485 | 607.7732002 | 1.4613 | 0.0030225 | 0.010456 | -- | GTP cyclohydrolase II |
| ZMO_RS07650 | 3735.548876 | 1012.545049 | 1.8833 | 3.61E-09 | 8.85E-08 | -- | hypothetical protein |
| ZMO_RS07665 | 2034.661841 | 1154.30207 | 0.81777 | 0.0093971 | 0.026771 | -- | ubiquinone biosynthesis protein UbiH |
| ZMO_RS07670 | 4519.742212 | 731.4726143 | 2.6274 | 0.0014957 | 0.0059491 | -- | peptidase S16 |
| ZMO_RS07675 | 13520.05183 | 346.9113844 | 5.2844 | 5.57E-05 | 0.00037902 | -- | thioredoxin |
| ZMO_RS07680 | 509.1557844 | 1128.779644 | -1.1486 | 0.00069299 | 0.003134 | -- | orotate phosphoribosyltransferase |
| ZMO_RS07695 | 1749.596953 | 2936.577753 | -0.74711 | 0.018398 | 0.046068 | -- | signal peptidase I |
| ZMO_RS07705 | 2262.946301 | 4205.543867 | -0.89409 | 0.006108 | 0.018865 | -- | FKBP-type peptidyl-prolyl cis-trans isomerase |
| ZMO_RS07710 | 4725.826828 | 10515.3146 | -1.1539 | 0.00063376 | 0.0029474 | -- | 30S ribosomal protein S21 |
| ZMO_RS07715 | 948.236222 | 3411.585758 | -1.8471 | 1.09E-08 | 2.35E-07 | -- | biopolymer transporter ExbD |
| ZMO_RS07720 | 660.1869714 | 1913.36205 | -1.5352 | 3.86E-06 | 3.92E-05 | -- | biopolymer transporter ExbD |
| ZMO_RS07725 | 1864.887907 | 5212.911682 | -1.483 | 2.46E-06 | 2.60E-05 | -- | MotA/TolQ/ExbB proton channel family protein |
| ZMO_RS07730 | 2601.395445 | 6537.3545 | -1.3294 | 2.10E-05 | 0.00016492 | -- | energy transducer TonB |
| ZMO_RS07735 | 578.3120761 | 1370.729154 | -1.245 | 0.00020756 | 0.0011382 | -- | hypothetical protein |
| ZMO_RS07750 | 12975.56598 | 2133.547308 | 2.6045 | 5.12E-16 | 8.56E-14 | -- | VOC family protein |
| ZMO_RS07775 | 1397.141637 | 428.3997638 | 1.7054 | 4.54E-06 | 4.51E-05 | -- | ATP-dependent Clp protease adaptor ClpS |
| ZMO_RS07780 | 15555.1726 | 7022.781047 | 1.1473 | 0.00011305 | 0.00068155 | -- | pyridoxal phosphate-dependent aminotransferase |
| ZMO_RS07815 | 926.9945781 | 468.3377828 | 0.98501 | 0.0049952 | 0.015903 | -- | glycosyl transferase family 1 |
| ZMO_RS07820 | 2313.254481 | 833.1311231 | 1.4733 | 0.000383 | 0.0019223 | -- | DNA-binding response regulator |
| ZMO_RS07835 | 3006.321598 | 5772.999792 | -0.94132 | 0.0018998 | 0.0072706 | -- | elongation factor 4 |
| ZMO_RS07855 | 426.9376252 | 985.9195794 | -1.2074 | 0.0004685 | 0.002295 | -- | methylenetetrahydrofolate reductase [NAD(P)H] |
| ZMO_RS07860 | 813.4634158 | 1345.891643 | -0.72641 | 0.016203 | 0.041687 | -- | methyltransferase domain-containing protein |
| ZMO_RS07865 | 911.8152835 | 1820.294726 | -0.99736 | 0.0018419 | 0.0070933 | -- | ABC transporter |
| ZMO_RS07870 | 792.3617374 | 281.743706 | 1.4918 | 3.61E-05 | 0.00025801 | -- | proteinase IV |
| ZMO_RS07890 | 9827.193467 | 671.617288 | 3.8711 | 9.73E-07 | 1.19E-05 | -- | NAD-dependent succinate-semialdehyde dehydrogenase |
| ZMO_RS07900 | 2332.766948 | 6363.781249 | -1.4478 | 5.40E-06 | 5.19E-05 | -- | 2-keto-3-deoxygluconate permease |
| ZMO_RS07905 | 674.924867 | 292.6960673 | 1.2053 | 0.00074286 | 0.0033284 | -- | gluconokinase |
| ZMO_RS07935 | 1062.402183 | 2574.812248 | -1.2771 | 7.52E-05 | 0.00047945 | -- | hypothetical protein |
| ZMO_RS07940 | 1432.323372 | 3487.143846 | -1.2837 | 3.67E-05 | 0.00026134 | -- | peptidoglycan-binding protein |
| ZMO_RS07975 | 1129.357461 | 308.8007448 | 1.8708 | 0.00063687 | 0.0029544 | -- | NAD(P)H quinone oxidoreductase |
| ZMO_RS07980 | 2637.347976 | 873.4136419 | 1.5944 | 6.20E-07 | 8.13E-06 | -- | aldo/keto reductase |
| ZMO_RS07990 | 757.6706391 | 288.4851778 | 1.3931 | 0.00010861 | 0.00066067 | -- | hypothetical protein |
| ZMO_RS07995 | 14913.31342 | 3406.051213 | 2.1304 | 5.60E-12 | 3.03E-10 | -- | aminopeptidase N |
| ZMO_RS08010 | 29716.21412 | 14275.77529 | 1.0577 | 0.00032289 | 0.0016568 | -- | antibiotic biosynthesis monooxygenase |
| ZMO_RS08015 | 22523.78876 | 9124.491876 | 1.3036 | 1.71E-05 | 0.00014157 | -- | monooxygenase |
| ZMO_RS08050 | 1940.483029 | 711.8760909 | 1.4467 | 7.17E-06 | 6.76E-05 | -- | hypothetical protein |
| ZMO_RS08060 | 1210.60016 | 2102.054527 | -0.79608 | 0.012895 | 0.03443 | -- | dihydroxy-acid dehydratase |
| ZMO_RS08065 | 214.5720764 | 100.7459335 | 1.0907 | 0.016632 | 0.042391 | -- | LysR family transcriptional regulator |
| ZMO_RS08075 | 2238.525369 | 6476.580316 | -1.5327 | 9.39E-07 | 1.16E-05 | -- | hypothetical protein |
| ZMO_RS08085 | 3458.035149 | 7177.71874 | -1.0536 | 0.007714 | 0.022746 | -- | (d)CMP kinase |
| ZMO_RS08090 | 29742.89308 | 109468.7615 | -1.8799 | 1.97E-09 | 5.26E-08 | -- | 30S ribosomal protein S1 |
| ZMO_RS08100 | 2315.000726 | 1290.971487 | 0.84256 | 0.0082102 | 0.024016 | -- | integration host factor subunit beta |
| ZMO_RS08110 | 1734.840559 | 1031.162327 | 0.75053 | 0.017336 | 0.043853 | -- | haloacid dehalogenase |
| ZMO_RS08115 | 496.5023562 | 108.3668909 | 2.1959 | 0.0052106 | 0.016503 | -- | sugar O-acetyltransferase |
| ZMO_RS08170 | 764.1591732 | 225.5323324 | 1.7605 | 1.51E-06 | 1.72E-05 | -- | nitrogenase cofactor biosynthesis protein NifB |
| ZMO_RS08190 | 298.2462346 | 131.1328787 | 1.1855 | 0.013357 | 0.035509 | -- | SIR2 family protein |
| ZMO_RS08205 | 327.4672524 | 47.38446387 | 2.7889 | 0.00016501 | 0.00094138 | -- | nitrogenase molybdenum-iron protein alpha chain |
| ZMO_RS08210 | 341.7099422 | 84.40755727 | 2.0173 | 0.00089087 | 0.0039151 | -- | nitrogenase molybdenum-iron protein subunit beta |
| ZMO_RS08220 | 185.8253187 | 81.59457046 | 1.1874 | 0.012465 | 0.033477 | -- | nitrogenase iron-molybdenum cofactor biosynthesis protein NifN |
| ZMO_RS08225 | 67.42084243 | 17.14945548 | 1.975 | 0.0091138 | 0.026159 | -- | nitrogen fixation protein NifX |
| ZMO_RS08240 | 91.67414806 | 21.95128469 | 2.0622 | 0.0041547 | 0.013653 | -- | nitrogen fixation protein NifQ |
| ZMO_RS08285 | 1280.358288 | 371.0351609 | 1.7869 | 1.43E-07 | 2.26E-06 | -- | sigma E positive regulator RseC/MucC |
| ZMO_RS08290 | 7302.012207 | 3113.738344 | 1.2296 | 5.40E-05 | 0.00036875 | -- | FAD:protein FMN transferase |
| ZMO_RS08300 | 728.8064883 | 2253.012337 | -1.6282 | 5.41E-07 | 7.21E-06 | -- | PhnA protein |
| ZMO_RS08315 | 450.8874877 | 68.54396075 | 2.7177 | 0.0033964 | 0.011554 | -- | ABC transporter substrate-binding protein |
| ZMO_RS08335 | 252.5105157 | 576.245206 | -1.1903 | 0.0010821 | 0.0045908 | -- | dihydrodipicolinate synthase family protein |
| ZMO_RS08345 | 247.0561007 | 701.1224344 | -1.5048 | 4.25E-05 | 0.00029885 | -- | GMP synthase |
| ZMO_RS08355 | 757.9518351 | 192.6527932 | 1.9761 | 0.0020517 | 0.0077683 | -- | LysR family transcriptional regulator |
| ZMO_RS08390 | 1431.708818 | 549.4720513 | 1.3816 | 6.41E-05 | 0.00042196 | -- | carbohydrate porin |
| ZMO_RS08395 | 2307.829217 | 989.1616483 | 1.2223 | 0.0020636 | 0.0077683 | -- | VIT family protein |
| ZMO_RS08400 | 3146.936259 | 746.2438847 | 2.0762 | 8.41E-06 | 7.77E-05 | -- | nitronate monooxygenase |
| ZMO_RS08460 | 1758.031985 | 819.0879804 | 1.1019 | 0.00077489 | 0.003455 | -- | biotin--[acetyl-CoA-carboxylase] ligase |
| ZMO_RS08475 | 5754.387083 | 2614.988592 | 1.1379 | 0.0065313 | 0.019864 | -- | quinolinate synthase NadA |
| ZMO_RS08480 | 3844.730392 | 728.3018257 | 2.4003 | 0.0013342 | 0.0054222 | -- | DUF4230 domain-containing protein |
| ZMO_RS08485 | 6153.765399 | 880.8819131 | 2.8044 | 0.00014697 | 0.00084903 | -- | monothiol glutaredoxin%2C Grx4 family |
| ZMO_RS08490 | 401.9946589 | 109.7616974 | 1.8728 | 0.008898 | 0.02562 | -- | BolA family transcriptional regulator |
| ZMO_RS08495 | 3749.089977 | 819.2309857 | 2.1942 | 0.0016025 | 0.0062767 | -- | DUF1476 domain-containing protein |
| ZMO_RS08505 | 2124.739252 | 993.6648952 | 1.0965 | 0.00059326 | 0.0027873 | -- | glutamine cyclotransferase |
| ZMO_RS08510 | 4039.527788 | 7024.742803 | -0.79826 | 0.01116 | 0.030502 | -- | valine--tRNA ligase |
| ZMO_RS08535 | 862.2231122 | 406.0629978 | 1.0864 | 0.0017907 | 0.0069254 | -- | glyoxylate/hydroxypyruvate reductase A |
| ZMO_RS08540 | 1308.859906 | 571.4765846 | 1.1955 | 0.000259 | 0.0013912 | -- | hypothetical protein |
| ZMO_RS08545 | 12341.72077 | 1481.363464 | 3.0585 | 2.86E-21 | 1.05E-18 | -- | alkene reductase |
| ZMO_RS08575 | 3832.48709 | 6995.216699 | -0.86809 | 0.0063059 | 0.019339 | -- | threonine synthase |
| ZMO_RS08590 | 1184.453228 | 2350.838567 | -0.98895 | 0.0023351 | 0.0085622 | -- | adenosine kinase |
| ZMO_RS08595 | 1851.556093 | 3963.930664 | -1.0982 | 0.0004618 | 0.0022683 | -- | protein translocase subunit SecF |
| ZMO_RS08600 | 3056.044924 | 5823.246979 | -0.93016 | 0.0030708 | 0.010603 | -- | protein translocase subunit SecD |
| ZMO_RS08605 | 1197.793005 | 2213.304089 | -0.88582 | 0.0064344 | 0.019667 | -- | preprotein translocase subunit YajC |
| ZMO_RS08615 | 1756.622988 | 3992.641838 | -1.1845 | 0.0044775 | 0.01443 | -- | ketoacyl-ACP synthase III |
| ZMO_RS08620 | 4963.053348 | 13905.26788 | -1.4863 | 1.63E-06 | 1.85E-05 | -- | phosphate acyltransferase |
| ZMO_RS08650 | 7135.030912 | 19402.04522 | -1.4432 | 2.70E-06 | 2.82E-05 | -- | translation initiation factor IF-3 |
| ZMO_RS08665 | 4479.138266 | 1289.096188 | 1.7969 | 0.016531 | 0.042294 | -- | DUF177 domain-containing protein |
| ZMO_RS08670 | 6566.588369 | 21688.15249 | -1.7237 | 2.67E-08 | 5.17E-07 | -- | 50S ribosomal protein L25 |
| ZMO_RS08675 | 956.9042527 | 1960.068214 | -1.0345 | 0.0012376 | 0.0051072 | -- | peptidyl-tRNA hydrolase |
| ZMO_RS08710 | 2157.124975 | 531.5959145 | 2.0207 | 0.0028033 | 0.0099222 | -- | adenosylmethionine--8-amino-7-oxononanoate transaminase |
| ZMO_RS08720 | 3227.850311 | 740.4987223 | 2.124 | 9.32E-06 | 8.51E-05 | -- | hypothetical protein |
| ZMO_RS08725 | 2060.169812 | 717.2798328 | 1.5222 | 0.0043726 | 0.014217 | -- | primosomal protein N' |
| ZMO_RS08730 | 2390.272067 | 4913.118434 | -1.0395 | 0.00072111 | 0.0032468 | -- | capsular polysaccharide biosynthesis protein |
| ZMO_RS08755 | 28087.20641 | 1212.157006 | 4.5343 | 1.00E-05 | 9.11E-05 | -- | molecular chaperone GroES |
| ZMO_RS08760 | 151899.3595 | 11711.76295 | 3.6971 | 6.33E-07 | 8.24E-06 | -- | molecular chaperone GroEL |
| ZMO_RS08780 | 46.06757688 | 138.5296991 | -1.5884 | 0.0044629 | 0.014408 | -- | DNA repair protein RadC |
| ZMO_RS08785 | 340.809875 | 1144.767669 | -1.748 | 6.64E-07 | 8.53E-06 | -- | hypothetical protein |
| ZMO_RS08790 | 704.9909412 | 2101.118987 | -1.5755 | 1.67E-06 | 1.86E-05 | -- | hypothetical protein |
| ZMO_RS08795 | 1986.751126 | 4641.468598 | -1.2242 | 9.63E-05 | 0.00059938 | -- | type I restriction-modification protein subunit M |
| ZMO_RS08855 | 1629.256217 | 425.5504567 | 1.9368 | 0.0036396 | 0.012245 | -- | PLP-dependent aminotransferase family protein |
| ZMO_RS08860 | 334.2409422 | 57.29361223 | 2.5444 | 0.0010459 | 0.0044682 | -- | PhzF family phenazine biosynthesis protein |
| ZMO_RS08865 | 166.2264094 | 29.90781031 | 2.4746 | 6.98E-05 | 0.00045482 | -- | KR domain-containing protein |
| ZMO_RS08950 | 1910.954267 | 783.1913756 | 1.2869 | 0.0066793 | 0.020214 | -- | hypothetical protein |
| ZMO_RS08960 | 1578.049304 | 807.4477772 | 0.9667 | 0.0036217 | 0.012216 | -- | sensor histidine kinase |
| ZMO_RS08995 | 1387.994662 | 646.3999787 | 1.1025 | 0.0022776 | 0.0084124 | -- | 3-methyl-2-oxobutanoate hydroxymethyltransferase |
| ZMO_RS09000 | 1997.883258 | 943.1170087 | 1.083 | 0.0014417 | 0.0057953 | -- | pantoate--beta-alanine ligase |
| ZMO_RS09010 | 321.6135303 | 157.9619017 | 1.0258 | 0.012141 | 0.032798 | -- | ArgP/LysG family DNA-binding transcriptional regulator |
| ZMO_RS09020 | 398.4489243 | 830.4330547 | -1.0595 | 0.0027373 | 0.0097263 | -- | hypothetical protein |
| ZMO_RS09040 | 240.4375702 | 510.1736622 | -1.0853 | 0.003507 | 0.011886 | -- | ribosomal RNA small subunit methyltransferase G |
| ZMO_RS09045 | 908.8661222 | 2058.584701 | -1.1795 | 0.00018607 | 0.0010453 | -- | tRNA uridine 5-carboxymethylaminomethyl modification protein |
| ZMO_RS09050 | 2021.45161 | 251.8888894 | 3.0045 | 6.18E-15 | 8.71E-13 | -- | SMP-30/gluconolactonase/LRE family protein |
| ZMO_RS09055 | 2802.530082 | 535.6370716 | 2.3874 | 5.49E-13 | 3.60E-11 | -- | aldo/keto reductase |
| ZMO_RS09070 | 1729.236604 | 618.661052 | 1.4829 | 0.00072857 | 0.0032723 | -- | methylated-DNA--[protein]-cysteine S-methyltransferase |
| ZMO_RS09075 | 2580.289121 | 372.4113712 | 2.7926 | 7.48E-05 | 0.00047945 | -- | dienelactone hydrolase family protein |
| ZMO_RS09080 | 3672.806528 | 778.7230732 | 2.2377 | 1.21E-10 | 4.95E-09 | -- | quinone oxidoreductase |
| ZMO_RS09085 | 3395.399721 | 12511.18906 | -1.8816 | 1.76E-09 | 4.89E-08 | -- | transcription termination factor Rho |
| ZMO_RS09120 | 87382.4737 | 145577.2067 | -0.73637 | 0.010528 | 0.029222 | rnpB | RNase P RNA component class A |
| ZMO_RS09130 | 641.5109699 | 176.5121509 | 1.8617 | 0.00055396 | 0.002616 | -- | redox-sensitive transcriptional activator SoxR |
| ZMO_RS09145 | 409.4580087 | 186.7094205 | 1.1329 | 0.01636 | 0.041915 | -- | IS5/IS1182 family transposase |
| ZMO_RS09155 | 1786.283825 | 900.299332 | 0.98848 | 0.0021678 | 0.0081271 | -- | hypothetical protein |
| ZMO_RS09170 | 601.9882946 | 126.9021701 | 2.246 | 1.61E-07 | 2.50E-06 | -- | DUF465 domain-containing protein |
| ZMO_RS09185 | 2943.696496 | 10265.65034 | -1.8021 | 8.09E-09 | 1.81E-07 | -- | 50S ribosomal protein L35 |
| ZMO_RS09190 | 46.69922793 | 116.7388063 | -1.3218 | 0.018741 | 0.046649 | -- | 50S ribosomal protein L34 |
| ZMO_RS09195 | 29.3458759 | 94.61423439 | -1.6889 | 0.010664 | 0.029457 | -- | membrane protein insertion efficiency factor YidD |
| ZMO_RS09205 | 1929.847185 | 3930.672203 | -1.0263 | 0.0037583 | 0.012553 | -- | 50S ribosomal protein L32 |
| ZMO_RS09210 | 96.47240328 | 223.6807117 | -1.2133 | 0.00726 | 0.021721 | -- | hypothetical protein |
| sRNA00010 | 9.193023432 | 100.7087277 | -3.4535 | 0.0014495 | 0.005806 | -//- |  |
| sRNA00017 | 1619.376457 | 7218.510683 | -2.1563 | 1.01E-11 | 5.00E-10 | -//- |  |
| sRNA00026 | 1388.148597 | 5241.019781 | -1.9167 | 1.02E-09 | 3.29E-08 | -//- |  |
| sRNA00048 | 385.8752714 | 1501.064578 | -1.9598 | 6.53E-09 | 1.50E-07 | -//- |  |
| sRNA00059 | 106.5776222 | 12.49791317 | 3.0921 | 0.0028895 | 0.010091 | -//- |  |
| sRNA00061 | 43.03764132 | 165.1827633 | -1.9404 | 0.00037157 | 0.0018804 | -//- |  |

**Table S8** Differentially Expressed Genes of wild type ZM4 in rich media and media with acetic + furfural treatment

| Gene_id | readcount_AF_ZM4 | readcount_RM_ZM4 | log2FoldChange | pval | padj | gene_name | description |
| --- | --- | --- | --- | --- | --- | --- | --- |
| Novel00001 | 3292.079153 | 1964.012401 | 0.74519 | 0.00096 | 0.0029461 | -//- |  |
| Novel00002 | 2488.536883 | 832.6931357 | 1.5794 | 0.00209 | 0.0059221 | -//- |  |
| Novel00003 | 1617.802816 | 3382.42811 | -1.064 | 0.00067 | 0.0021589 | -//- |  |
| Novel00004 | 2044.428559 | 123.6668249 | 4.0472 | 3.27E-06 | 1.82E-05 | -//- |  |
| Novel00005 | 3767.053109 | 86.82150675 | 5.4392 | 0.01604 | 0.034595 | -//- |  |
| Novel00007 | 1698.402053 | 145.3033168 | 3.547 | 1.01E-15 | 3.14E-14 | -//- |  |
| Novel00008 | 314.5839411 | 820.9042816 | -1.3838 | 8.14E-08 | 6.21E-07 | -//- |  |
| Novel00009 | 2309.410957 | 862.7046242 | 1.4206 | 0.00998 | 0.023042 | -//- |  |
| ZMO_RS00025 | 1240.466318 | 751.3896337 | 0.72325 | 0.00327 | 0.0087299 | -- | sulfate adenylyltransferase subunit 2 |
| ZMO_RS00040 | 4639.419665 | 3100.126011 | 0.58162 | 0.01067 | 0.024396 | -- | assimilatory sulfite reductase (NADPH) hemoprotein subunit |
| ZMO_RS00045 | 4655.146783 | 2911.06735 | 0.67728 | 0.01167 | 0.026321 | -- | assimilatory sulfite reductase (NADPH) flavoprotein subunit |
| ZMO_RS00050 | 497.6780841 | 179.1760014 | 1.4738 | 1.09E-07 | 8.07E-07 | -- | M48 family peptidase |
| ZMO_RS00060 | 226.6475346 | 437.9173777 | -0.95021 | 0.00035 | 0.0012019 | -- | radical SAM protein |
| ZMO_RS00065 | 284.0389545 | 612.4831442 | -1.1086 | 1.59E-05 | 7.57E-05 | -- | non-canonical purine NTP pyrophosphatase |
| ZMO_RS00075 | 17178.13536 | 2285.179041 | 2.9102 | 3.08E-14 | 7.56E-13 | hrcA | HrcA family transcriptional regulator |
| ZMO_RS00080 | 10003.34723 | 1698.31161 | 2.5583 | 1.92E-08 | 1.64E-07 | -- | nucleotide exchange factor GrpE |
| ZMO_RS00085 | 6749.029036 | 3079.15581 | 1.1321 | 0.00581 | 0.014379 | -- | methyltransferase domain-containing protein |
| ZMO_RS00090 | 988.0633218 | 1659.279612 | -0.74788 | 0.00151 | 0.0044183 | -- | ribulose-phosphate 3-epimerase |
| ZMO_RS00095 | 2048.630921 | 3600.56906 | -0.81356 | 0.00027 | 0.00094201 | -- | heparinase |
| ZMO_RS00120 | 386.8805799 | 237.8629902 | 0.70176 | 0.0103 | 0.023694 | -- | EamA/RhaT family transporter |
| ZMO_RS00130 | 3196.786506 | 5785.763172 | -0.85588 | 0.00376 | 0.0097967 | -- | bifunctional phosphoribosylaminoimidazolecarboxamide formyltransferase/IMP cyclohydrolase PurH |
| ZMO_RS00140 | 1856.949275 | 1156.500079 | 0.68317 | 0.00294 | 0.0079901 | -- | sel1 repeat family protein |
| ZMO_RS00165 | 748.9781892 | 158.9682027 | 2.2362 | 5.96E-05 | 0.00024547 | -- | DUF1491 domain-containing protein |
| ZMO_RS00170 | 1536.053104 | 365.8833989 | 2.0698 | 9.88E-13 | 1.85E-11 | -- | nitrogen regulatory protein |
| ZMO_RS00175 | 7357.115425 | 1407.212843 | 2.3863 | 9.38E-26 | 8.21E-24 | -- | ribosomal subunit interface protein |
| ZMO_RS00195 | 440.9410026 | 249.4965121 | 0.82157 | 0.00153 | 0.0044514 | -- | septum formation inhibitor Maf |
| ZMO_RS00225 | 314.3683597 | 190.1011986 | 0.72569 | 0.01099 | 0.024957 | -- | MarR family transcriptional regulator |
| ZMO_RS00230 | 620.8792306 | 123.3459383 | 2.3316 | 7.22E-05 | 0.00028849 | -- | sulfite exporter TauE/SafE family protein |
| ZMO_RS00235 | 9786.749005 | 2655.870941 | 1.8816 | 5.96E-17 | 2.33E-15 | -- | glutamine--fructose-6-phosphate aminotransferase |
| ZMO_RS00250 | 883.8025172 | 377.4202209 | 1.2276 | 2.32E-06 | 1.34E-05 | -- | glycerate kinase |
| ZMO_RS00255 | 410.784654 | 725.84445 | -0.82128 | 0.00095 | 0.0029044 | -- | hypothetical protein |
| ZMO_RS00270 | 361.3044344 | 691.4018032 | -0.93631 | 0.00015 | 0.00056615 | -- | carbohydrate porin |
| ZMO_RS00285 | 175.1509674 | 98.7148491 | 0.82726 | 0.014 | 0.030966 | -- | tail tube protein |
| ZMO_RS00290 | 3379.997585 | 805.1384209 | 2.0697 | 4.12E-19 | 2.10E-17 | -- | glutaredoxin 2 |
| ZMO_RS00295 | 1722.915548 | 3354.538456 | -0.96126 | 1.65E-05 | 7.78E-05 | -- | hypothetical protein |
| ZMO_RS00305 | 691.8776932 | 470.9143444 | 0.55505 | 0.02165 | 0.044705 | -- | endoribonuclease YbeY |
| ZMO_RS00310 | 4946.446998 | 2831.598369 | 0.80478 | 0.00019 | 0.00070462 | -- | PhoH family protein |
| ZMO_RS00320 | 5002.670615 | 2262.903818 | 1.1445 | 3.43E-07 | 2.31E-06 | -- | chemotaxis protein CheW |
| ZMO_RS00325 | 2656.304078 | 1320.5778 | 1.0083 | 5.12E-06 | 2.69E-05 | -- | response regulator |
| ZMO_RS00345 | 4526.648496 | 2254.828353 | 1.0054 | 4.48E-06 | 2.41E-05 | -- | chemotaxis protein CheA |
| ZMO_RS00350 | 893.2860146 | 515.7117999 | 0.79256 | 0.00079 | 0.0024795 | -- | STAS domain-containing protein |
| ZMO_RS00365 | 209.6391579 | 399.5801487 | -0.93058 | 0.00077 | 0.0024357 | -- | hypothetical protein |
| ZMO_RS00395 | 237.0754914 | 791.6117144 | -1.7394 | 0.004 | 0.010366 | -- | hypothetical protein |
| ZMO_RS00410 | 426.2188948 | 1038.927656 | -1.2854 | 8.71E-08 | 6.58E-07 | -- | MFS transporter |
| ZMO_RS00420 | 1465.880959 | 196.3839437 | 2.9 | 0.00074 | 0.0023608 | -- | NAD dependent epimerase/dehydratase |
| ZMO_RS00435 | 6487.766493 | 3129.556711 | 1.0518 | 0.01973 | 0.041354 | -- | 3-isopropylmalate dehydratase large subunit |
| ZMO_RS00440 | 1883.62844 | 934.1775433 | 1.0117 | 0.02105 | 0.043698 | -- | 3-isopropylmalate dehydratase small subunit |
| ZMO_RS00445 | 911.8940603 | 1887.941718 | -1.0499 | 7.39E-06 | 3.75E-05 | -- | hypothetical protein |
| ZMO_RS00450 | 396.5755153 | 659.8441093 | -0.73453 | 0.00353 | 0.0092481 | -- | QacE family quaternary ammonium compound efflux SMR transporter |
| ZMO_RS00460 | 2372.196313 | 1407.399371 | 0.75319 | 0.00941 | 0.021953 | -- | glycosyl transferase family 2 |
| ZMO_RS00470 | 461.8873476 | 212.5709894 | 1.1196 | 3.68E-05 | 0.00016059 | -- | hypothetical protein |
| ZMO_RS00475 | 2971.866254 | 1143.577588 | 1.3778 | 1.29E-07 | 9.42E-07 | -- | aminodeoxychorismate synthase%2C component I |
| ZMO_RS00480 | 690.7046593 | 298.3313636 | 1.2112 | 1.69E-06 | 1.02E-05 | -- | type 1 glutamine amidotransferase |
| ZMO_RS00485 | 1262.980195 | 819.786256 | 0.62351 | 0.00724 | 0.017531 | -- | aminotransferase |
| ZMO_RS00495 | 2741.060812 | 1296.158839 | 1.0805 | 2.35E-06 | 1.36E-05 | -- | hydroxylamine reductase |
| ZMO_RS00500 | 234.9432496 | 121.8105088 | 0.94767 | 0.00278 | 0.0076115 | -- | Na+/H+ antiporter NhaA |
| ZMO_RS00510 | 2178.95222 | 1332.77215 | 0.7092 | 0.00149 | 0.0043652 | -- | replication-associated recombination protein A |
| ZMO_RS00515 | 3140.116207 | 742.2939287 | 2.0808 | 4.92E-05 | 0.00020601 | -- | hypothetical protein |
| ZMO_RS00525 | 1644.397768 | 785.8525057 | 1.0652 | 4.93E-06 | 2.60E-05 | -- | Cys-tRNA(Pro) deacylase |
| ZMO_RS00540 | 272.4979707 | 830.0080228 | -1.6069 | 2.14E-05 | 9.80E-05 | -- | TonB-dependent receptor |
| ZMO_RS00545 | 111.4807218 | 659.6847316 | -2.565 | 1.72E-13 | 3.64E-12 | -- | acid phosphatase |
| ZMO_RS00550 | 202.2579106 | 786.8423047 | -1.9599 | 5.66E-07 | 3.70E-06 | -- | metallophosphoesterase |
| ZMO_RS00560 | 2498.865139 | 567.1439527 | 2.1395 | 2.32E-19 | 1.22E-17 | -- | sel1 repeat family protein |
| ZMO_RS00600 | 2916.049373 | 1858.594823 | 0.6498 | 0.00271 | 0.0074517 | -- | YggS family pyridoxal phosphate-dependent enzyme |
| ZMO_RS00605 | 2774.44118 | 1586.756206 | 0.80612 | 0.00123 | 0.0036881 | -- | ABC transporter ATP-binding protein |
| ZMO_RS00610 | 4714.461298 | 2025.382843 | 1.2189 | 3.15E-08 | 2.56E-07 | -- | L-aspartate oxidase |
| ZMO_RS00620 | 1839.306445 | 3014.145167 | -0.71259 | 0.00219 | 0.0061661 | -- | DUF2141 domain-containing protein |
| ZMO_RS00625 | 696.6605335 | 1862.450468 | -1.4187 | 2.62E-09 | 2.79E-08 | -- | tRNA (guanosine(46)-N7)-methyltransferase TrmB |
| ZMO_RS00640 | 1286.259099 | 1986.298043 | -0.6269 | 0.01071 | 0.02445 | -- | DUF2312 domain-containing protein |
| ZMO_RS00645 | 5092.079301 | 9557.606377 | -0.90839 | 5.06E-05 | 0.0002107 | -- | YebC/PmpR family DNA-binding transcriptional regulator |
| ZMO_RS00650 | 567.8613312 | 989.1983434 | -0.80072 | 0.00063 | 0.0020201 | -- | crossover junction endodeoxyribonuclease RuvC |
| ZMO_RS00655 | 281.8014747 | 538.2160902 | -0.93351 | 0.00037 | 0.001257 | -- | Holliday junction branch migration protein RuvA |
| ZMO_RS00665 | 2136.060086 | 1331.896876 | 0.68147 | 0.00347 | 0.0091602 | -- | 2-hydroxyacid dehydrogenase |
| ZMO_RS00670 | 1420.828199 | 901.3751272 | 0.65653 | 0.01215 | 0.027326 | -- | Holliday junction branch migration DNA helicase RuvB |
| ZMO_RS00690 | 1031.303206 | 1643.068203 | -0.67192 | 0.0032 | 0.0085711 | -- | hypothetical protein |
| ZMO_RS00715 | 12897.7435 | 2860.49012 | 2.1728 | 3.93E-16 | 1.31E-14 | -- | NAD(P)-dependent oxidoreductase |
| ZMO_RS00735 | 1282.446445 | 616.0377324 | 1.0578 | 1.16E-05 | 5.62E-05 | -- | cell division protein ZapA |
| ZMO_RS00740 | 819.4982979 | 165.233961 | 2.3102 | 5.00E-18 | 2.19E-16 | -- | nuclear transport factor 2 family protein |
| ZMO_RS00750 | 7315.961476 | 10590.50903 | -0.53365 | 0.01404 | 0.031011 | -- | transketolase |
| ZMO_RS00765 | 6377.255307 | 2882.467657 | 1.1456 | 1.39E-07 | 1.01E-06 | -- | fructose-bisphosphate aldolase |
| ZMO_RS00775 | 4175.989152 | 8802.464226 | -1.0758 | 1.85E-06 | 1.11E-05 | -- | adenosylhomocysteinase |
| ZMO_RS00780 | 7040.890239 | 10478.9369 | -0.57366 | 0.01015 | 0.02338 | -- | energy-dependent translational throttle protein EttA |
| ZMO_RS00785 | 9088.163228 | 45144.94547 | -2.3125 | 2.87E-08 | 2.38E-07 | -- | hypothetical protein |
| ZMO_RS00815 | 669.2122376 | 2097.821053 | -1.6484 | 3.79E-11 | 5.38E-10 | -- | MurR/RpiR family transcriptional regulator |
| ZMO_RS00830 | 5491.571545 | 8539.917251 | -0.637 | 0.00298 | 0.0080664 | -- | ribonuclease E/G |
| ZMO_RS00835 | 380.2234009 | 616.9126319 | -0.69822 | 0.00772 | 0.018499 | -- | hypothetical protein |
| ZMO_RS00855 | 5257.422213 | 1077.478705 | 2.2867 | 2.21E-05 | 0.00010066 | -- | LexA repressor |
| ZMO_RS00860 | 3116.388534 | 1038.246933 | 1.5857 | 2.75E-12 | 4.59E-11 | -- | anthranilate phosphoribosyltransferase |
| ZMO_RS00865 | 2007.868529 | 857.0905931 | 1.2281 | 6.38E-08 | 4.89E-07 | -- | type 1 glutamine amidotransferase |
| ZMO_RS00870 | 8051.185671 | 13678.19719 | -0.7646 | 0.00077 | 0.0024285 | -- | methyl-accepting chemotaxis protein |
| ZMO_RS00875 | 534.4783922 | 45.88278641 | 3.5421 | 7.63E-30 | 8.76E-28 | -- | MerR family DNA-binding transcriptional regulator |
| ZMO_RS00885 | 2378.789112 | 1352.979389 | 0.81409 | 0.00037 | 0.0012569 | -- | NAD(P)-dependent oxidoreductase |
| ZMO_RS00890 | 5608.338308 | 3030.254362 | 0.88813 | 6.73E-05 | 0.0002719 | -- | glutamate 5-kinase |
| ZMO_RS00895 | 5550.450734 | 3982.5822 | 0.4789 | 0.02392 | 0.048794 | obgE | GTPase ObgE |
| ZMO_RS00900 | 10324.49658 | 2136.66015 | 2.2726 | 5.74E-16 | 1.85E-14 | -- | N-acetyltransferase |
| ZMO_RS00910 | 12637.20706 | 23831.20909 | -0.91517 | 2.54E-05 | 0.00011384 | -- | 50S ribosomal protein L21 |
| ZMO_RS00925 | 4332.720614 | 233.3410269 | 4.2148 | 1.15E-40 | 3.02E-38 | -- | 5-formyltetrahydrofolate cyclo-ligase |
| ZMO_RS00935 | 4046.349362 | 2138.876994 | 0.91977 | 4.18E-05 | 0.00017818 | -- | helicase |
| ZMO_RS00940 | 2714.293019 | 7257.691197 | -1.4189 | 4.76E-10 | 5.83E-09 | -- | ferredoxin family protein |
| ZMO_RS00965 | 989.7374673 | 1729.96284 | -0.80562 | 0.00049 | 0.0016118 | -- | homoserine O-acetyltransferase |
| ZMO_RS00975 | 1395.034925 | 3821.714078 | -1.4539 | 1.34E-10 | 1.74E-09 | -- | DNA polymerase I |
| ZMO_RS00980 | 557.9781593 | 1549.372633 | -1.4734 | 2.63E-10 | 3.29E-09 | -- | polysaccharide biosynthesis protein |
| ZMO_RS00995 | 3742.72748 | 927.7225752 | 2.0123 | 1.90E-14 | 4.93E-13 | -- | hypothetical protein |
| ZMO_RS01000 | 1114.468178 | 1710.596481 | -0.61814 | 0.00725 | 0.017545 | -- | 23S rRNA (guanosine(2251)-2'-O)-methyltransferase RlmB |
| ZMO_RS01015 | 12501.23633 | 8538.994201 | 0.54993 | 0.0109 | 0.024799 | -- | PDZ domain-containing protein |
| ZMO_RS01020 | 2877.117143 | 1507.565376 | 0.9324 | 2.48E-05 | 0.00011174 | -- | ATP-binding protein |
| ZMO_RS01055 | 911.3459474 | 1378.006791 | -0.59651 | 0.01241 | 0.027886 | -- | hypothetical protein |
| ZMO_RS01060 | 6492.36755 | 19035.94474 | -1.5519 | 2.12E-09 | 2.29E-08 | -- | HU family DNA-binding protein |
| ZMO_RS01065 | 5170.495588 | 642.4698912 | 3.0086 | 6.30E-37 | 1.05E-34 | -- | HslU--HslV peptidase proteolytic subunit |
| ZMO_RS01070 | 16701.39056 | 2114.605495 | 2.9815 | 1.71E-33 | 2.62E-31 | -- | ATP-dependent protease ATPase subunit HslU |
| ZMO_RS01075 | 7557.537768 | 11995.40227 | -0.66649 | 0.00335 | 0.0089106 | -- | 50S ribosomal protein L33 |
| ZMO_RS01090 | 3318.485193 | 6102.120885 | -0.87879 | 9.60E-05 | 0.00037316 | -- | major intrinsic protein |
| ZMO_RS01095 | 168.6534389 | 360.5573665 | -1.0962 | 5.18E-05 | 0.00021436 | -- | channel protein TolC |
| ZMO_RS01100 | 278.474309 | 564.3223028 | -1.019 | 0.00065 | 0.0020848 | -- | type I secretion system permease/ATPase |
| ZMO_RS01110 | 3902.213455 | 2481.489919 | 0.65309 | 0.00863 | 0.02036 | -- | D-lactate dehydrogenase |
| ZMO_RS01115 | 5810.191161 | 2461.053988 | 1.2393 | 1.61E-08 | 1.41E-07 | -- | DNA-binding response regulator |
| ZMO_RS01120 | 1292.453884 | 702.3871279 | 0.87977 | 0.00035 | 0.001193 | -- | ATP-binding protein |
| ZMO_RS01130 | 317.223849 | 181.2179626 | 0.80778 | 0.00744 | 0.017916 | -- | cytochrome C biogenesis protein CcmB |
| ZMO_RS01135 | 114.5021771 | 247.3674618 | -1.1113 | 0.00029 | 0.0010109 | -- | hypothetical protein |
| ZMO_RS01140 | 590.0283291 | 1674.637894 | -1.505 | 0.0001 | 0.00039276 | -- | hypothetical protein |
| ZMO_RS01145 | 944.8061093 | 1751.464071 | -0.89047 | 0.00776 | 0.018562 | -- | hypothetical protein |
| ZMO_RS01150 | 453.5004619 | 742.2434911 | -0.71079 | 0.01653 | 0.035545 | -- | hypothetical protein |
| ZMO_RS01190 | 4118.398613 | 12468.06712 | -1.5981 | 1.65E-12 | 2.94E-11 | -- | S-adenosylmethionine synthase |
| ZMO_RS01200 | 632.6322248 | 1136.392431 | -0.84502 | 0.00041 | 0.0013784 | -- | LPS export ABC transporter ATP-binding protein |
| ZMO_RS01205 | 690.0708216 | 1333.4352 | -0.95033 | 8.35E-05 | 0.00032844 | -- | organic solvent tolerance protein OstA |
| ZMO_RS01210 | 864.0533622 | 1827.515966 | -1.0807 | 4.51E-06 | 2.42E-05 | -- | LPS export ABC transporter periplasmic protein LptC |
| ZMO_RS01215 | 2915.949198 | 5105.840846 | -0.80818 | 0.00032 | 0.0010949 | -- | ribonuclease D |
| ZMO_RS01240 | 750.7668014 | 1150.757901 | -0.61615 | 0.00808 | 0.019239 | -- | RND transporter |
| ZMO_RS01245 | 2318.200115 | 991.3215539 | 1.2256 | 3.09E-08 | 2.52E-07 | -- | SIMPL domain-containing protein |
| ZMO_RS01255 | 3972.260587 | 278.7303123 | 3.833 | 7.66E-53 | 4.70E-50 | -- | DUF445 domain-containing protein |
| ZMO_RS01260 | 355.2365612 | 1340.166071 | -1.9156 | 2.22E-15 | 6.47E-14 | -- | prolipoprotein diacylglyceryl transferase |
| ZMO_RS01265 | 976.2937128 | 426.3516072 | 1.1953 | 1.91E-06 | 1.14E-05 | -- | sugar porter family MFS transporter |
| ZMO_RS01285 | 2496.921741 | 939.75295 | 1.4098 | 8.07E-10 | 9.27E-09 | -- | hypothetical protein |
| ZMO_RS01290 | 1839.14438 | 2821.541311 | -0.61745 | 0.00908 | 0.021256 | -- | phosphoribosylamine--glycine ligase |
| ZMO_RS01295 | 2749.556406 | 1511.385401 | 0.86333 | 0.00012 | 0.00046175 | -- | exodeoxyribonuclease VII large subunit |
| ZMO_RS01305 | 18.31846632 | 106.2967311 | -2.5367 | 1.11E-08 | 1.04E-07 | -- | aryl-sulfate sulfotransferase |
| ZMO_RS01310 | 117.1565728 | 494.9832431 | -2.0789 | 1.23E-13 | 2.73E-12 | -- | ferrochelatase |
| ZMO_RS01315 | 208.9319905 | 618.0113681 | -1.5646 | 4.73E-09 | 4.75E-08 | -- | stress responsive protein |
| ZMO_RS01320 | 562.0707638 | 1259.835069 | -1.1644 | 1.20E-06 | 7.49E-06 | -- | glycosyltransferase family 1 protein |
| ZMO_RS01325 | 389.0682342 | 801.3475569 | -1.0424 | 2.57E-05 | 0.00011492 | -- | capsular polysaccharide biosynthesis protein |
| ZMO_RS01330 | 2451.481157 | 425.7502013 | 2.5256 | 0.00377 | 0.0098068 | -- | KR domain-containing protein |
| ZMO_RS01335 | 1758.022232 | 770.2450357 | 1.1906 | 1.40E-07 | 1.01E-06 | -- | pyrroline-5-carboxylate reductase |
| ZMO_RS01340 | 2393.049583 | 1114.893855 | 1.1019 | 1.38E-06 | 8.43E-06 | -- | amidohydrolase |
| ZMO_RS01345 | 963.4098236 | 366.0800842 | 1.396 | 1.59E-08 | 1.40E-07 | -- | 2-amino-thiazoline-4-carboxylic acid hydrolase |
| ZMO_RS01375 | 228.4473082 | 517.0171102 | -1.1784 | 3.74E-05 | 0.0001619 | -- | dihydrofolate reductase |
| ZMO_RS01385 | 7615.612653 | 11173.67905 | -0.55307 | 0.01393 | 0.030882 | -- | isoleucine--tRNA ligase |
| ZMO_RS01410 | 2222.77955 | 4479.073585 | -1.0108 | 7.30E-06 | 3.72E-05 | -- | elongation factor P |
| ZMO_RS01415 | 2134.056008 | 4533.820669 | -1.0871 | 1.93E-06 | 1.14E-05 | -- | inositol monophosphatase |
| ZMO_RS01425 | 9816.257662 | 2839.460953 | 1.7896 | 3.25E-11 | 4.73E-10 | -- | DUF882 domain-containing protein |
| ZMO_RS01480 | 4450.32686 | 2652.470286 | 0.74657 | 0.00082 | 0.0025461 | -- | RNA chaperone Hfq |
| ZMO_RS01500 | 1764.453706 | 2483.266302 | -0.49302 | 0.01749 | 0.037296 | -- | DUF4011 domain-containing protein |
| ZMO_RS01510 | 2396.892733 | 1596.086567 | 0.58663 | 0.00778 | 0.018585 | -- | KR domain-containing protein |
| ZMO_RS01515 | 3179.779488 | 1704.357161 | 0.8997 | 6.97E-05 | 0.00028082 | -- | DNA mismatch repair endonuclease MutL |
| ZMO_RS01525 | 1337.584125 | 2291.2725 | -0.77652 | 0.0005 | 0.0016597 | -- | rod shape-determining protein MreC |
| ZMO_RS01530 | 298.2549919 | 502.536407 | -0.75268 | 0.00319 | 0.0085663 | -- | hypothetical protein |
| ZMO_RS01535 | 1728.325728 | 1089.991561 | 0.66506 | 0.00407 | 0.010518 | -- | penicillin-binding protein 2 |
| ZMO_RS01550 | 9898.955469 | 917.2935805 | 3.4318 | 6.49E-47 | 2.98E-44 | -- | excinuclease ABC subunit B |
| ZMO_RS01585 | 163.8927768 | 65.73010513 | 1.3181 | 0.0003 | 0.0010555 | -- | aspartate/glutamate racemase family protein |
| ZMO_RS01595 | 192.9544793 | 105.3540662 | 0.87301 | 0.00617 | 0.015211 | -- | hypothetical protein |
| ZMO_RS01615 | 36784.93606 | 6775.626147 | 2.4407 | 3.72E-08 | 2.97E-07 | -- | endopeptidase La |
| ZMO_RS01725 | 543.6946746 | 914.5934648 | -0.75033 | 0.00154 | 0.0044948 | -- | ribosomal RNA large subunit methyltransferase E |
| ZMO_RS01740 | 49010.74874 | 4487.640189 | 3.4491 | 1.88E-06 | 1.12E-05 | -- | ATP-dependent Clp protease ATP-binding subunit ClpA |
| ZMO_RS01750 | 10005.35622 | 6137.421139 | 0.70507 | 0.00141 | 0.0041564 | -- | GcrA cell cycle regulator |
| ZMO_RS01775 | 751.2781759 | 3590.588971 | -2.2568 | 2.09E-22 | 1.42E-20 | -- | MarC family protein |
| ZMO_RS01790 | 355.4652118 | 206.7689142 | 0.78169 | 0.00565 | 0.013993 | -- | cell division ATP-binding protein FtsE |
| ZMO_RS01800 | 521.4707135 | 309.8679554 | 0.75093 | 0.00464 | 0.011738 | -- | YdcF family protein |
| ZMO_RS01810 | 1765.21877 | 2758.967501 | -0.64428 | 0.00422 | 0.01084 | -- | protein TyrC |
| ZMO_RS01815 | 3892.781193 | 5957.828098 | -0.61399 | 0.0053 | 0.013221 | -- | histidinol-phosphate aminotransferase |
| ZMO_RS01845 | 1949.07085 | 475.9486207 | 2.0339 | 3.05E-13 | 6.29E-12 | -- | SUF system Fe-S cluster assembly protein |
| ZMO_RS01850 | 824.1647187 | 223.920424 | 1.8799 | 4.13E-13 | 8.25E-12 | -- | iron-sulfur cluster assembly accessory protein |
| ZMO_RS01855 | 2308.252337 | 1038.211165 | 1.1527 | 0.0001 | 0.00039276 | -- | pyrimidine 5'-nucleotidase |
| ZMO_RS01870 | 616.0106148 | 1582.056891 | -1.3608 | 1.37E-08 | 1.23E-07 | -- | guanylate kinase |
| ZMO_RS01875 | 8556.234772 | 3698.928412 | 1.2099 | 6.18E-08 | 4.77E-07 | -- | glycine zipper 2TM domain-containing protein |
| ZMO_RS01885 | 226.2054316 | 433.7804394 | -0.93933 | 0.00058 | 0.0018851 | -- | hypothetical protein |
| ZMO_RS01895 | 1636.767473 | 567.4211071 | 1.5284 | 8.13E-11 | 1.08E-09 | -- | hypothetical protein |
| ZMO_RS01900 | 4202.270948 | 1998.089657 | 1.0725 | 1.17E-06 | 7.34E-06 | -- | ATPase |
| ZMO_RS01925 | 1076.141782 | 2232.907151 | -1.0531 | 4.79E-06 | 2.55E-05 | -- | MBOAT family protein |
| ZMO_RS01930 | 254.4490563 | 448.7399768 | -0.8185 | 0.00223 | 0.0062425 | -- | hypothetical protein |
| ZMO_RS01935 | 6042.898068 | 2683.934517 | 1.1709 | 0.01424 | 0.031337 | -- | cell wall hydrolase |
| ZMO_RS01955 | 4634.954592 | 6997.427256 | -0.59427 | 0.00753 | 0.018089 | -- | ribosome biogenesis GTPase Der |
| ZMO_RS01965 | 925.2314096 | 1389.17597 | -0.58634 | 0.00786 | 0.018738 | -- | (2Fe-2S)-binding protein |
| ZMO_RS01970 | 515.7579342 | 801.6914387 | -0.63635 | 0.00759 | 0.018204 | -- | glutamate uptake regulatory protein |
| ZMO_RS01975 | 297.9225328 | 544.9740879 | -0.87125 | 0.00079 | 0.0024795 | -- | lipoprotein-releasing system ATP-binding protein LolD |
| ZMO_RS01980 | 1014.334535 | 1700.809668 | -0.74569 | 0.00126 | 0.0037548 | -- | lipoprotein-releasing system transmembrane subunit%2C LolC/LolE family |
| ZMO_RS01985 | 3072.762867 | 4533.70425 | -0.56115 | 0.0154 | 0.033572 | -- | proline--tRNA ligase |
| ZMO_RS01990 | 3119.363593 | 10060.31398 | -1.6894 | 9.59E-14 | 2.15E-12 | -- | CTP synthetase |
| ZMO_RS01995 | 1522.828355 | 3295.721694 | -1.1138 | 1.01E-06 | 6.40E-06 | -- | preprotein translocase subunit SecG |
| ZMO_RS02015 | 4269.536894 | 1168.024332 | 1.87 | 1.31E-08 | 1.19E-07 | -- | hypothetical protein |
| ZMO_RS02020 | 1045.143435 | 1577.388061 | -0.59384 | 0.01185 | 0.026679 | -- | LysR family transcriptional regulator |
| ZMO_RS02030 | 2683.22032 | 1647.012151 | 0.70411 | 0.00165 | 0.0047614 | -- | 6%2C7-dimethyl-8-ribityllumazine synthase |
| ZMO_RS02035 | 7624.783378 | 3524.080771 | 1.1134 | 3.26E-07 | 2.21E-06 | -- | 3%2C4-dihydroxy-2-butanone-4-phosphate synthase |
| ZMO_RS02040 | 2994.575205 | 1654.623477 | 0.85585 | 8.85E-05 | 0.00034672 | -- | riboflavin synthase |
| ZMO_RS02070 | 211.5551227 | 330.907606 | -0.64539 | 0.02044 | 0.042656 | -- | hypothetical protein |
| ZMO_RS02080 | 3010.906057 | 4505.348033 | -0.58144 | 0.01256 | 0.028148 | -- | homoserine dehydrogenase |
| ZMO_RS02085 | 735.8908676 | 355.8487235 | 1.0482 | 2.02E-05 | 9.28E-05 | -- | MgtC/SapB family protein |
| ZMO_RS02090 | 4329.625947 | 2896.501958 | 0.57993 | 0.00666 | 0.016272 | -- | acyl-CoA dehydrogenase |
| ZMO_RS02095 | 1987.309332 | 1178.74149 | 0.75357 | 0.00097 | 0.0029697 | -- | hypothetical protein |
| ZMO_RS02100 | 1387.973836 | 872.6470368 | 0.66951 | 0.0041 | 0.010563 | -- | CoA ester lyase |
| ZMO_RS02105 | 332.0045324 | 738.7587574 | -1.1539 | 7.16E-06 | 3.66E-05 | -- | hypothetical protein |
| ZMO_RS02120 | 1999.438669 | 919.933349 | 1.12 | 6.31E-07 | 4.07E-06 | -- | S9 family peptidase |
| ZMO_RS02140 | 831.9727117 | 3405.396652 | -2.0332 | 1.70E-18 | 7.82E-17 | -- | Bcr/CflA family drug resistance efflux transporter |
| ZMO_RS02165 | 4616.719018 | 9202.605465 | -0.99517 | 5.83E-06 | 3.04E-05 | -- | penicillin-binding protein |
| ZMO_RS02170 | 1111.438864 | 2259.413732 | -1.0235 | 9.25E-06 | 4.62E-05 | -- | hypothetical protein |
| ZMO_RS02175 | 223.2645869 | 481.044176 | -1.1074 | 3.03E-05 | 0.00013473 | -- | GtrA family protein |
| ZMO_RS02180 | 711.2971206 | 2195.029913 | -1.6257 | 6.02E-13 | 1.18E-11 | -- | hypothetical protein |
| ZMO_RS02185 | 2407.206975 | 5164.904792 | -1.1014 | 9.10E-07 | 5.79E-06 | -- | glycosyltransferase family 2 protein |
| ZMO_RS02190 | 524.5526558 | 1605.340038 | -1.6137 | 1.57E-11 | 2.39E-10 | -- | RNA pseudouridine synthase |
| ZMO_RS02195 | 460.9055739 | 678.9263068 | -0.55878 | 0.02401 | 0.048929 | -- | aminoacyl-tRNA hydrolase |
| ZMO_RS02220 | 1613.444201 | 745.9223283 | 1.113 | 2.86E-06 | 1.62E-05 | -- | acyl-CoA thioesterase |
| ZMO_RS02230 | 1033.637519 | 1489.286542 | -0.52689 | 0.01573 | 0.034088 | -- | phosphatase PAP2 family protein |
| ZMO_RS02235 | 17836.52883 | 29841.21886 | -0.74247 | 0.00093 | 0.0028517 | -- | 30S ribosomal protein S12 |
| ZMO_RS02240 | 10119.74032 | 16366.46643 | -0.69357 | 0.00213 | 0.0060047 | -- | 30S ribosomal protein S7 |
| ZMO_RS02245 | 61122.38273 | 121473.3034 | -0.99087 | 9.64E-06 | 4.80E-05 | -- | elongation factor G |
| ZMO_RS02250 | 87684.71089 | 158267.01 | -0.85196 | 0.00098 | 0.0029904 | -- | elongation factor Tu |
| ZMO_RS02255 | 1581.064135 | 9661.816094 | -2.6114 | 1.03E-29 | 1.12E-27 | -- | 30S ribosomal protein S10 |
| ZMO_RS02260 | 23331.08197 | 48226.92128 | -1.0476 | 2.19E-06 | 1.28E-05 | -- | 50S ribosomal protein L3 |
| ZMO_RS02265 | 8749.159589 | 18955.72385 | -1.1154 | 5.44E-07 | 3.57E-06 | -- | 50S ribosomal protein L4 |
| ZMO_RS02270 | 1917.152754 | 4864.788472 | -1.3434 | 2.55E-09 | 2.72E-08 | -- | 50S ribosomal protein L23 |
| ZMO_RS02275 | 20020.23272 | 37677.76123 | -0.91225 | 3.70E-05 | 0.00016059 | -- | 50S ribosomal protein L2 |
| ZMO_RS02280 | 699.0976357 | 2021.942351 | -1.5322 | 3.94E-11 | 5.53E-10 | -- | 30S ribosomal protein S19 |
| ZMO_RS02285 | 3642.954453 | 8053.870124 | -1.1446 | 2.30E-07 | 1.60E-06 | -- | 50S ribosomal protein L22 |
| ZMO_RS02290 | 16476.81337 | 29068.43522 | -0.81902 | 0.00019 | 0.00070462 | -- | 30S ribosomal protein S3 |
| ZMO_RS02295 | 6859.485933 | 15098.32602 | -1.1382 | 3.12E-07 | 2.12E-06 | -- | 50S ribosomal protein L16 |
| ZMO_RS02300 | 151.5368132 | 265.8840349 | -0.81113 | 0.00632 | 0.015488 | -- | 50S ribosomal protein L29 |
| ZMO_RS02305 | 287.2926889 | 659.8307012 | -1.1996 | 1.08E-05 | 5.30E-05 | -- | 30S ribosomal protein S17 |
| ZMO_RS02310 | 3950.595942 | 9035.628922 | -1.1936 | 1.32E-05 | 6.33E-05 | -- | 50S ribosomal protein L14 |
| ZMO_RS02315 | 2346.906461 | 5839.945755 | -1.3152 | 4.04E-09 | 4.11E-08 | -- | 50S ribosomal protein L24 |
| ZMO_RS02320 | 11043.1253 | 23357.54658 | -1.0807 | 0.00019 | 0.00070462 | -- | 50S ribosomal protein L5 |
| ZMO_RS02325 | 4041.329363 | 10327.78995 | -1.3536 | 3.93E-09 | 4.01E-08 | -- | 30S ribosomal protein S14 |
| ZMO_RS02330 | 3851.844853 | 9839.436171 | -1.353 | 1.14E-05 | 5.57E-05 | -- | 30S ribosomal protein S8 |
| ZMO_RS02335 | 6189.37862 | 12946.60723 | -1.0647 | 3.92E-05 | 0.00016807 | -- | 50S ribosomal protein L6 |
| ZMO_RS02340 | 2233.329393 | 3982.799864 | -0.83459 | 0.00017 | 0.00062051 | -- | 50S ribosomal protein L18 |
| ZMO_RS02350 | 353.0253937 | 918.9447286 | -1.3802 | 2.39E-08 | 2.01E-07 | -- | 50S ribosomal protein L30 |
| ZMO_RS02355 | 10721.43938 | 19399.58646 | -0.85553 | 0.00019 | 0.00070334 | -- | 50S ribosomal protein L15 |
| ZMO_RS02360 | 7446.490207 | 15815.59153 | -1.0867 | 1.93E-06 | 1.14E-05 | -- | preprotein translocase subunit SecY |
| ZMO_RS02365 | 2621.838152 | 6212.661882 | -1.2446 | 6.20E-08 | 4.77E-07 | -- | adenylate kinase |
| ZMO_RS02370 | 9135.804188 | 31991.07921 | -1.8081 | 1.46E-15 | 4.48E-14 | -- | 30S ribosomal protein S13 |
| ZMO_RS02375 | 3918.276577 | 14753.55891 | -1.9128 | 5.91E-17 | 2.33E-15 | -- | 30S ribosomal protein S11 |
| ZMO_RS02380 | 12794.07402 | 43671.46461 | -1.7712 | 6.26E-15 | 1.72E-13 | -- | DNA-directed RNA polymerase subunit alpha |
| ZMO_RS02385 | 5572.908049 | 18625.50904 | -1.7408 | 1.60E-14 | 4.20E-13 | -- | 50S ribosomal protein L17 |
| ZMO_RS02390 | 95.36635767 | 227.947601 | -1.2571 | 3.57E-05 | 0.00015616 | -- | tRNA-Ser |
| ZMO_RS02420 | 1245.426403 | 740.7137772 | 0.74965 | 0.00179 | 0.0051256 | -- | voltage-gated chloride channel protein |
| ZMO_RS02430 | 21417.1639 | 63321.52636 | -1.5639 | 3.50E-12 | 5.69E-11 | -- | polyribonucleotide nucleotidyltransferase |
| ZMO_RS02435 | 2740.444865 | 6775.777708 | -1.306 | 7.44E-09 | 7.20E-08 | -- | 30S ribosomal protein S15 |
| ZMO_RS02440 | 1545.554654 | 4558.925057 | -1.5606 | 1.22E-12 | 2.23E-11 | -- | tRNA pseudouridine(55) synthase TruB |
| ZMO_RS02450 | 577.1854099 | 1001.813024 | -0.79551 | 0.00081 | 0.0025217 | -- | ribosome-binding factor A |
| ZMO_RS02455 | 18523.35022 | 54061.50856 | -1.5453 | 2.35E-12 | 4.04E-11 | -- | translation initiation factor IF-2 |
| ZMO_RS02460 | 2510.263797 | 9342.577185 | -1.896 | 2.12E-17 | 8.84E-16 | -- | DUF448 domain-containing protein |
| ZMO_RS02470 | 3148.232339 | 4573.706243 | -0.53882 | 0.00979 | 0.022671 | -- | ribosome maturation factor |
| ZMO_RS02490 | 2921.982234 | 1505.521905 | 0.95668 | 0.00128 | 0.0037867 | -- | TonB-dependent receptor |
| ZMO_RS02500 | 1243.846072 | 1976.213569 | -0.66793 | 0.00621 | 0.015255 | -- | chorismate mutase |
| ZMO_RS02510 | 1918.014617 | 2772.964034 | -0.53182 | 0.0221 | 0.045591 | -- | ATP-dependent helicase HrpB |
| ZMO_RS02525 | 113.6927155 | 217.7717689 | -0.93768 | 0.0028 | 0.0076484 | -- | hypothetical protein |
| ZMO_RS02530 | 253.429298 | 436.7771405 | -0.78531 | 0.00416 | 0.010695 | -- | succinate dehydrogenase cytochrome b subunit |
| ZMO_RS02545 | 9784.375805 | 3302.920971 | 1.5667 | 7.01E-13 | 1.33E-11 | -- | sulfonate ABC transporter permease |
| ZMO_RS02550 | 5553.896412 | 2154.661633 | 1.366 | 4.65E-10 | 5.74E-09 | -- | nitrate ABC transporter ATP-binding protein |
| ZMO_RS02565 | 430.2354086 | 1336.207236 | -1.6349 | 9.27E-12 | 1.44E-10 | -- | 16S rRNA (guanine(966)-N(2))-methyltransferase RsmD |
| ZMO_RS02570 | 1738.597001 | 5789.854805 | -1.7356 | 1.55E-14 | 4.12E-13 | -- | RNA-binding protein S4 |
| ZMO_RS02575 | 1676.539063 | 2483.365683 | -0.56681 | 0.01798 | 0.038165 | -- | dicarboxylate/amino acid:cation symporter |
| ZMO_RS02580 | 848.5647251 | 1453.501597 | -0.77644 | 0.0008 | 0.0025123 | -- | MFS transporter |
| ZMO_RS02585 | 1776.968974 | 3661.691378 | -1.0431 | 3.89E-06 | 2.13E-05 | -- | bifunctional folylpolyglutamate synthase/dihydrofolate synthase |
| ZMO_RS02630 | 828.324977 | 476.724566 | 0.79704 | 0.00092 | 0.0028302 | -- | iron-sulfur cluster assembly scaffold protein |
| ZMO_RS02635 | 238.9706351 | 143.8135748 | 0.73263 | 0.01309 | 0.029207 | -- | DUF423 domain-containing protein |
| ZMO_RS02645 | 1941.228309 | 3411.079159 | -0.81326 | 0.00034 | 0.0011647 | -- | shikimate kinase |
| ZMO_RS02655 | 1902.231361 | 1193.254345 | 0.67279 | 0.00393 | 0.010197 | -- | tyrosine recombinase XerD |
| ZMO_RS02665 | 696.2376644 | 1227.900809 | -0.81854 | 0.00038 | 0.0012939 | -- | hybrid sensor histidine kinase/response regulator |
| ZMO_RS02675 | 1209.77356 | 2228.951918 | -0.88163 | 0.00052 | 0.0017026 | -- | flagellar motor protein MotB |
| ZMO_RS02680 | 1808.29234 | 3335.165615 | -0.88313 | 0.00124 | 0.0036881 | -- | flagellar motor stator protein MotA |
| ZMO_RS02695 | 479.0811311 | 1192.203775 | -1.3153 | 0.00023 | 0.00080683 | -- | flagellar protein FlgJ |
| ZMO_RS02700 | 2205.453866 | 4687.33694 | -1.0877 | 0.00268 | 0.0073877 | -- | flagellar P-ring protein |
| ZMO_RS02705 | 1114.165339 | 2489.290099 | -1.1598 | 0.00168 | 0.0048325 | -- | flagellar L-ring protein |
| ZMO_RS02710 | 2143.744469 | 4747.554592 | -1.1471 | 4.80E-05 | 0.00020174 | -- | flagellar basal-body rod protein FlgG |
| ZMO_RS02715 | 3563.105729 | 7682.748737 | -1.1085 | 9.23E-05 | 0.00036097 | -- | flagellar basal-body rod protein FlgF |
| ZMO_RS02720 | 10563.23368 | 18689.08167 | -0.82314 | 0.01418 | 0.031257 | -- | flagellar hook protein FlgE |
| ZMO_RS02725 | 4340.249836 | 6642.501534 | -0.61395 | 0.00834 | 0.01977 | -- | flagellar hook capping protein |
| ZMO_RS02730 | 1010.67241 | 1726.508464 | -0.77254 | 0.00089 | 0.0027587 | -- | flagellar basal body rod protein FlgC |
| ZMO_RS02735 | 2340.448928 | 3531.873294 | -0.59365 | 0.00897 | 0.02102 | -- | flagellar basal body rod protein FlgB |
| ZMO_RS02760 | 919.4296898 | 631.8792842 | 0.54109 | 0.02313 | 0.047443 | -- | flagellar biosynthesis anti-sigma factor FlgM |
| ZMO_RS02770 | 9371.993457 | 6590.552395 | 0.50796 | 0.01991 | 0.041676 | -- | flagellar biosynthesis protein FlhA |
| ZMO_RS02795 | 4938.599606 | 1053.030995 | 2.2296 | 1.97E-15 | 5.85E-14 | -- | flagellin |
| ZMO_RS02800 | 11700.69268 | 7353.157158 | 0.67016 | 0.00547 | 0.0136 | -- | sigma-54-dependent Fis family transcriptional regulator |
| ZMO_RS02875 | 605.058803 | 364.1755504 | 0.73244 | 0.00318 | 0.0085507 | -- | flagellar biosynthetic protein FliR |
| ZMO_RS02880 | 1105.847258 | 607.2527596 | 0.86478 | 0.0003 | 0.0010457 | -- | flagellar biosynthesis protein FlhB |
| ZMO_RS02885 | 3223.072511 | 1963.903022 | 0.71471 | 0.00123 | 0.0036881 | -- | flagellar hook protein FliD |
| ZMO_RS02900 | 3043.007664 | 5622.717022 | -0.88577 | 7.22E-05 | 0.00028849 | -- | polysaccharide deacetylase |
| ZMO_RS02905 | 1038.59556 | 3216.925699 | -1.631 | 2.47E-12 | 4.21E-11 | -- | adenosine deaminase |
| ZMO_RS02910 | 1914.451995 | 2879.701265 | -0.58899 | 0.01062 | 0.024344 | -- | phosphoribosyltransferase |
| ZMO_RS02930 | 119001.9208 | 18348.04083 | 2.6973 | 1.87E-32 | 2.46E-30 | -- | molecular chaperone DnaK |
| ZMO_RS02935 | 12194.87248 | 2556.448713 | 2.2541 | 8.74E-08 | 6.58E-07 | -- | molecular chaperone DnaJ |
| ZMO_RS02940 | 3095.369504 | 6439.519493 | -1.0568 | 6.15E-05 | 0.0002519 | -- | adenylosuccinate lyase |
| ZMO_RS02960 | 4675.737954 | 1567.338122 | 1.5769 | 2.85E-12 | 4.72E-11 | -- | DUF1318 domain-containing protein |
| ZMO_RS02990 | 1575.416539 | 1082.019333 | 0.54201 | 0.01961 | 0.041226 | -- | excinuclease ABC subunit C |
| ZMO_RS03000 | 578.8700829 | 197.5071115 | 1.5513 | 3.35E-08 | 2.71E-07 | -- | hypothetical protein |
| ZMO_RS03005 | 1817.702035 | 1188.631499 | 0.61281 | 0.00476 | 0.012023 | -- | LysR family transcriptional regulator |
| ZMO_RS03025 | 832.1585587 | 489.3698546 | 0.76593 | 0.0012 | 0.0036049 | -- | type I-F CRISPR-associated endonuclease Cas1 |
| ZMO_RS03030 | 3355.3057 | 1821.011561 | 0.8817 | 6.73E-05 | 0.0002719 | -- | type I-F CRISPR-associated helicase Cas3 |
| ZMO_RS03060 | 5634.312291 | 14168.61465 | -1.3304 | 0.01514 | 0.033082 | -- | acetolactate synthase large subunit |
| ZMO_RS03065 | 121.409018 | 244.2457039 | -1.0085 | 0.00116 | 0.0034995 | -- | transporter |
| ZMO_RS03075 | 12521.68 | 34735.20136 | -1.472 | 1.99E-05 | 9.19E-05 | -- | glucose-fructose oxidoreductase |
| ZMO_RS03080 | 2213.000717 | 4263.87926 | -0.94616 | 3.45E-05 | 0.00015218 | -- | FADH(2)-oxidizing methylenetetrahydrofolate--tRNA-(uracil(54)-C(5))-methyltransferase TrmFO |
| ZMO_RS03090 | 9533.795236 | 1607.943953 | 2.5678 | 4.57E-06 | 2.44E-05 | -- | organic hydroperoxide resistance protein |
| ZMO_RS03095 | 8918.623271 | 3300.853616 | 1.434 | 3.70E-11 | 5.31E-10 | -- | hypothetical protein |
| ZMO_RS03105 | 540.1291592 | 1434.634451 | -1.4093 | 4.91E-09 | 4.88E-08 | -- | hypothetical protein |
| ZMO_RS03115 | 98.73324162 | 186.6397938 | -0.91865 | 0.00531 | 0.013225 | -- | QacE family quaternary ammonium compound efflux SMR transporter |
| ZMO_RS03135 | 6857.176453 | 13310.75979 | -0.95691 | 2.32E-05 | 0.00010523 | -- | acyl-CoA synthetase |
| ZMO_RS03140 | 690.0350345 | 319.3732966 | 1.1114 | 1.55E-05 | 7.37E-05 | -- | recombinase RecX |
| ZMO_RS03175 | 5789.459505 | 2963.596324 | 0.96608 | 1.15E-05 | 5.59E-05 | -- | RNA degradosome polyphosphate kinase |
| ZMO_RS03185 | 1094.015285 | 1910.083883 | -0.804 | 0.0009 | 0.0027816 | -- | ribonuclease D |
| ZMO_RS03190 | 4338.321993 | 9739.878878 | -1.1668 | 2.32E-07 | 1.61E-06 | -- | aspartate--tRNA(Asp/Asn) ligase |
| ZMO_RS03195 | 969.6942309 | 1939.225127 | -0.99988 | 1.62E-05 | 7.69E-05 | -- | hypothetical protein |
| ZMO_RS03225 | 1318.943618 | 827.3105231 | 0.67288 | 0.00966 | 0.02244 | -- | tRNA-Trp |
| ZMO_RS03240 | 9596.384007 | 14144.04892 | -0.55963 | 0.01106 | 0.025044 | -- | 50S ribosomal protein L11 |
| ZMO_RS03245 | 13274.55527 | 19590.69097 | -0.5615 | 0.01081 | 0.024624 | -- | 50S ribosomal protein L1 |
| ZMO_RS03250 | 16186.99358 | 46947.20646 | -1.5362 | 3.46E-12 | 5.68E-11 | -- | 50S ribosomal protein L10 |
| ZMO_RS03255 | 12876.58885 | 44420.34796 | -1.7865 | 3.56E-14 | 8.50E-13 | -- | 50S ribosomal protein L7/L12 |
| ZMO_RS03265 | 108661.9512 | 65988.90294 | 0.71955 | 0.00095 | 0.0029166 | -- | DNA-directed RNA polymerase subunit beta |
| ZMO_RS03275 | 107.0966829 | 52.35193263 | 1.0326 | 0.00978 | 0.022659 | -- | acetyltransferase |
| ZMO_RS03285 | 4429.669632 | 7408.71742 | -0.74202 | 0.001 | 0.0030357 | -- | acetyl-CoA carboxylase biotin carboxylase subunit |
| ZMO_RS03290 | 1333.865851 | 2465.741121 | -0.88641 | 9.39E-05 | 0.0003664 | -- | acetyl-CoA carboxylase biotin carboxyl carrier protein |
| ZMO_RS03295 | 812.422712 | 1505.262752 | -0.88971 | 0.00015 | 0.00056759 | -- | type II 3-dehydroquinate dehydratase |
| ZMO_RS03310 | 7893.099318 | 1797.737811 | 2.1344 | 5.71E-11 | 7.79E-10 | -- | CsbD family protein |
| ZMO_RS03315 | 1788.575731 | 3271.466528 | -0.87113 | 0.00013 | 0.00049699 | -- | peptide chain release factor 3 |
| ZMO_RS03320 | 358.0398356 | 704.7853307 | -0.97706 | 8.66E-05 | 0.00034021 | -- | transporter |
| ZMO_RS03345 | 11188.52998 | 1471.998915 | 2.9262 | 3.39E-08 | 2.73E-07 | -- | cysteine synthase A |
| ZMO_RS03350 | 6167.454525 | 1805.491668 | 1.7723 | 2.35E-15 | 6.74E-14 | -- | RNA polymerase sigma factor RpoH |
| ZMO_RS03370 | 3234.36992 | 1283.574664 | 1.3333 | 5.23E-09 | 5.17E-08 | -- | glutaredoxin 3 |
| ZMO_RS03375 | 4724.567786 | 1335.44218 | 1.8229 | 2.49E-15 | 7.04E-14 | -- | sterol-binding protein |
| ZMO_RS03380 | 1020.602541 | 3181.866145 | -1.6405 | 1.20E-12 | 2.21E-11 | -- | tetratricopeptide repeat protein |
| ZMO_RS03405 | 2020.833554 | 1011.257663 | 0.9988 | 1.36E-05 | 6.53E-05 | -- | hypothetical protein |
| ZMO_RS03410 | 2457.211447 | 1406.479413 | 0.80493 | 0.00021 | 0.00076017 | -- | alpha/beta hydrolase |
| ZMO_RS03420 | 2220.2265 | 1180.644054 | 0.91113 | 5.18E-05 | 0.00021436 | -- | bifunctional uridylyltransferase/uridylyl-removing protein |
| ZMO_RS03430 | 237.1841462 | 136.8074055 | 0.79386 | 0.00782 | 0.018671 | -- | hypothetical protein |
| ZMO_RS03435 | 1417.600858 | 2205.866918 | -0.63789 | 0.00491 | 0.012357 | -- | tRNA 2-thiouridine(34) synthase MnmA |
| ZMO_RS03440 | 998.8504681 | 1814.783997 | -0.86146 | 0.00011 | 0.0004236 | -- | magnesium transporter |
| ZMO_RS03445 | 2790.721242 | 4613.005211 | -0.72507 | 0.00159 | 0.0046115 | -- | peptidylprolyl isomerase |
| ZMO_RS03450 | 800.5201961 | 491.2270578 | 0.70455 | 0.00318 | 0.0085453 | -- | LysR family transcriptional regulator |
| ZMO_RS03455 | 1105.98404 | 1610.99566 | -0.54262 | 0.01919 | 0.0405 | -- | methyltransferase type 12 |
| ZMO_RS03470 | 705.055854 | 1236.435788 | -0.81038 | 0.00053 | 0.0017234 | -- | efflux RND transporter periplasmic adaptor subunit |
| ZMO_RS03475 | 743.3632185 | 1750.890478 | -1.2359 | 2.30E-07 | 1.60E-06 | -- | RND transporter |
| ZMO_RS03480 | 2006.843971 | 4189.424134 | -1.0618 | 3.73E-06 | 2.05E-05 | -- | LysR family transcriptional regulator |
| ZMO_RS03490 | 5630.000139 | 8262.578772 | -0.55346 | 0.01768 | 0.03762 | gatA | Asp-tRNA(Asn)/Glu-tRNA(Gln) amidotransferase GatCAB subunit A |
| ZMO_RS03495 | 715.5434626 | 1103.253352 | -0.62465 | 0.00708 | 0.017168 | -- | Asp-tRNA(Asn)/Glu-tRNA(Gln) amidotransferase subunit GatC |
| ZMO_RS03500 | 410.0974564 | 673.140005 | -0.71494 | 0.00682 | 0.016593 | -- | Holliday junction resolvase RuvX |
| ZMO_RS03505 | 380.705802 | 590.1407611 | -0.63238 | 0.02045 | 0.042656 | -- | hypothetical protein |
| ZMO_RS03520 | 1246.302018 | 419.5770349 | 1.5706 | 4.74E-05 | 0.00019966 | -- | TonB-dependent receptor |
| ZMO_RS03550 | 301.8755233 | 668.352522 | -1.1467 | 6.69E-06 | 3.44E-05 | -- | NAD(P)-dependent oxidoreductase |
| ZMO_RS03555 | 289.3317273 | 586.7313859 | -1.02 | 4.50E-05 | 0.00019043 | -- | TolC family protein |
| ZMO_RS03560 | 75.69395284 | 258.6204611 | -1.7726 | 5.12E-08 | 4.02E-07 | -- | ABC transporter permease |
| ZMO_RS03565 | 262.8444095 | 836.2618234 | -1.6697 | 3.46E-11 | 5.01E-10 | -- | ABC transporter ATP-binding protein/permease |
| ZMO_RS03570 | 233.5198439 | 765.7497695 | -1.7133 | 5.65E-11 | 7.79E-10 | -- | secretion protein HlyD |
| ZMO_RS03585 | 1056.038974 | 1715.580587 | -0.70003 | 0.00313 | 0.0084284 | -- | N-acetyl-gamma-glutamyl-phosphate reductase |
| ZMO_RS03600 | 1448.10975 | 3856.170907 | -1.413 | 6.58E-10 | 7.61E-09 | -- | 30S ribosomal protein S12 methylthiotransferase RimO |
| ZMO_RS03650 | 1441.410992 | 2092.239371 | -0.53757 | 0.01347 | 0.029935 | -- | UDP-glucose/GDP-mannose dehydrogenase family protein |
| ZMO_RS03655 | 4268.02115 | 10474.97695 | -1.2953 | 8.44E-09 | 8.08E-08 | -- | phosphoribosylformylglycinamidine synthase subunit PurL |
| ZMO_RS03685 | 2032.368497 | 3139.656355 | -0.62744 | 0.00352 | 0.0092424 | -- | penicillin-binding protein 2 |
| ZMO_RS03695 | 1522.88174 | 2847.45114 | -0.90287 | 6.29E-05 | 0.00025698 | -- | UDP-N-acetylmuramoyl-tripeptide--D-alanyl-D-alanine ligase |
| ZMO_RS03700 | 683.2495117 | 1763.786499 | -1.3682 | 4.90E-09 | 4.88E-08 | -- | phospho-N-acetylmuramoyl-pentapeptide-transferase |
| ZMO_RS03705 | 1434.484254 | 2959.721802 | -1.0449 | 5.10E-06 | 2.69E-05 | -- | UDP-N-acetylmuramoyl-L-alanine--D-glutamate ligase |
| ZMO_RS03710 | 922.9453383 | 1898.257672 | -1.0404 | 0.00031 | 0.0010666 | -- | putative lipid II flippase FtsW |
| ZMO_RS03715 | 1512.193234 | 2687.431151 | -0.82959 | 0.0003 | 0.0010433 | -- | undecaprenyldiphospho-muramoylpentapeptide beta-N- acetylglucosaminyltransferase |
| ZMO_RS03720 | 1741.94307 | 4244.162445 | -1.2848 | 7.96E-07 | 5.10E-06 | -- | UDP-N-acetylmuramate--L-alanine ligase |
| ZMO_RS03725 | 1239.084938 | 2597.41302 | -1.0678 | 1.09E-05 | 5.33E-05 | -- | UDP-N-acetylenolpyruvoylglucosamine reductase |
| ZMO_RS03730 | 1324.559102 | 2390.543198 | -0.85183 | 0.00672 | 0.016373 | -- | D-alanine--D-alanine ligase |
| ZMO_RS03735 | 746.7891075 | 1652.920374 | -1.1462 | 1.19E-06 | 7.46E-06 | -- | cell division protein FtsQ |
| ZMO_RS03765 | 3556.703672 | 5600.114201 | -0.65492 | 0.00354 | 0.0092671 | -- | arginine--tRNA ligase |
| ZMO_RS03775 | 6506.314791 | 13605.90083 | -1.0643 | 3.08E-06 | 1.73E-05 | -- | alanine--tRNA ligase |
| ZMO_RS03785 | 4873.365286 | 10985.0786 | -1.1726 | 3.69E-05 | 0.00016059 | -- | carbohydrate porin |
| ZMO_RS03805 | 934.8315894 | 1583.379903 | -0.76023 | 0.00123 | 0.0036809 | -- | tRNA preQ1(34) S-adenosylmethionine ribosyltransferase-isomerase QueA |
| ZMO_RS03815 | 482.4100013 | 1329.670933 | -1.4627 | 1.29E-09 | 1.42E-08 | -- | phosphopantetheine adenylyltransferase |
| ZMO_RS03820 | 3328.054591 | 9371.263053 | -1.4936 | 5.72E-11 | 7.79E-10 | -- | polyprenyl synthetase family protein |
| ZMO_RS03825 | 588.6054161 | 1380.857586 | -1.2302 | 2.76E-07 | 1.89E-06 | -- | exodeoxyribonuclease 7 small subunit |
| ZMO_RS03830 | 2513.352874 | 8162.784886 | -1.6994 | 5.76E-14 | 1.32E-12 | -- | DUF1013 domain-containing protein |
| ZMO_RS03850 | 4893.303406 | 2983.224473 | 0.71394 | 0.00305 | 0.0082496 | -- | nucleoid-associated protein%2C YbaB/EbfC family |
| ZMO_RS03855 | 718.0387476 | 1501.402487 | -1.0642 | 5.71E-06 | 2.98E-05 | -- | dCTP deaminase |
| ZMO_RS03865 | 2827.043967 | 1618.861519 | 0.80431 | 0.00028 | 0.00098635 | -- | replicative DNA helicase |
| ZMO_RS03870 | 1996.865623 | 5969.678008 | -1.5799 | 2.17E-12 | 3.80E-11 | -- | cation transporter |
| ZMO_RS03875 | 1941.124577 | 4710.368323 | -1.2789 | 1.94E-05 | 8.99E-05 | -- | NAD-dependent dehydratase |
| ZMO_RS03880 | 1023.189834 | 1889.491985 | -0.88492 | 0.0002 | 0.00071866 | -- | glycosyl transferase family 2 |
| ZMO_RS03885 | 1200.28058 | 1810.042662 | -0.59265 | 0.02373 | 0.048515 | -- | squalene synthase HpnC |
| ZMO_RS03890 | 1081.498841 | 1949.899489 | -0.85037 | 0.00066 | 0.002133 | -- | squalene synthase HpnD |
| ZMO_RS03895 | 1040.000552 | 2095.208504 | -1.0105 | 0.00079 | 0.0024795 | -- | hypothetical protein |
| ZMO_RS03900 | 1291.68925 | 3830.429278 | -1.5682 | 5.59E-09 | 5.50E-08 | -- | squalene--hopene cyclase |
| ZMO_RS03910 | 7083.044827 | 14583.61983 | -1.0419 | 3.01E-06 | 1.70E-05 | -- | hopanoid biosynthesis associated radical SAM protein HpnH |
| ZMO_RS03915 | 5575.179386 | 9583.826295 | -0.78158 | 0.00045 | 0.0015109 | -- | 4-hydroxy-3-methylbut-2-enyl diphosphate reductase |
| ZMO_RS03920 | 4205.375558 | 2723.347977 | 0.62685 | 0.0193 | 0.040682 | -- | hopanoid biosynthesis associated membrane protein HpnM |
| ZMO_RS03930 | 7064.759165 | 3847.871562 | 0.87658 | 3.12E-05 | 0.0001382 | -- | chemotaxis-specific protein-glutamate methyltransferase CheB |
| ZMO_RS03945 | 6402.786223 | 21055.1975 | -1.7174 | 5.52E-14 | 1.30E-12 | -- | 30S ribosomal protein S9 |
| ZMO_RS03950 | 6341.383204 | 21682.20012 | -1.7736 | 5.34E-15 | 1.49E-13 | -- | 50S ribosomal protein L13 |
| ZMO_RS03955 | 1384.841119 | 177.5818396 | 2.9632 | 0.00055 | 0.0018135 | -- | MerC domain-containing protein |
| ZMO_RS03980 | 1055.378909 | 396.5958524 | 1.412 | 1.01E-08 | 9.58E-08 | -- | ATP-dependent DNA helicase |
| ZMO_RS03990 | 309.1042307 | 473.7036153 | -0.61589 | 0.01655 | 0.035546 | -- | NERD domain-containing protein |
| ZMO_RS03995 | 153.9109631 | 49.81582782 | 1.6274 | 1.63E-05 | 7.70E-05 | -- | hypothetical protein |
| ZMO_RS04030 | 30142.25861 | 6033.705573 | 2.3207 | 0.00236 | 0.0065866 | -- | 2-isopropylmalate synthase |
| ZMO_RS04035 | 2328.126531 | 949.8929383 | 1.2933 | 1.38E-08 | 1.23E-07 | -- | beta-galactosidase |
| ZMO_RS04040 | 4763.642884 | 2059.573739 | 1.2097 | 1.42E-07 | 1.02E-06 | -- | glucan biosynthesis protein D |
| ZMO_RS04055 | 2901.235876 | 6789.434579 | -1.2266 | 3.01E-08 | 2.48E-07 | -- | LPS biosynthesis protein |
| ZMO_RS04070 | 3302.207382 | 5634.878512 | -0.77095 | 0.00056 | 0.0018288 | -- | sugar transporter |
| ZMO_RS04075 | 716.3369383 | 1586.378477 | -1.147 | 6.25E-07 | 4.05E-06 | -- | DUF885 domain-containing protein |
| ZMO_RS04090 | 2517.964434 | 569.4872184 | 2.1445 | 1.24E-19 | 6.93E-18 | -- | copper-translocating P-type ATPase |
| ZMO_RS04095 | 585.7496639 | 59.68636475 | 3.2948 | 1.77E-07 | 1.25E-06 | -- | heavy metal transport/detoxification protein |
| ZMO_RS04105 | 5962.645215 | 1045.998796 | 2.5111 | 0.00137 | 0.0040462 | -- | catalase |
| ZMO_RS04110 | 1796.276946 | 4496.344619 | -1.3237 | 3.35E-09 | 3.46E-08 | -- | GGDEF domain-containing protein |
| ZMO_RS04115 | 926.799203 | 345.8655829 | 1.422 | 1.91E-08 | 1.64E-07 | -- | hypothetical protein |
| ZMO_RS04120 | 1491.435152 | 745.1906921 | 1.001 | 1.73E-05 | 8.11E-05 | -- | hypothetical protein |
| ZMO_RS04125 | 600.8953744 | 325.8628153 | 0.88285 | 0.0005 | 0.0016523 | -- | ASCH domain-containing protein |
| ZMO_RS04135 | 11154.51201 | 7031.83643 | 0.66565 | 0.00209 | 0.0059221 | -- | protein translocase subunit SecA |
| ZMO_RS04150 | 744.2331291 | 439.9712178 | 0.75835 | 0.00155 | 0.0045083 | -- | leucyl/phenylalanyl-tRNA--protein transferase |
| ZMO_RS04180 | 2439.372715 | 1122.595535 | 1.1197 | 0.00022 | 0.00079881 | -- | glutathione S-transferase |
| ZMO_RS04210 | 422.397547 | 180.2126884 | 1.2289 | 1.81E-05 | 8.44E-05 | -- | NAD-dependent epimerase |
| ZMO_RS04220 | 1942.818237 | 909.0202921 | 1.0958 | 1.56E-06 | 9.51E-06 | -- | sucrose-6-phosphate hydrolase |
| ZMO_RS04240 | 11417.06055 | 32433.8867 | -1.5063 | 1.81E-11 | 2.73E-10 | -- | trigger factor |
| ZMO_RS04245 | 886.0162626 | 3363.403505 | -1.9245 | 1.49E-16 | 5.47E-15 | -- | glycosyl transferase 2 protein |
| ZMO_RS04250 | 17329.80103 | 5399.283856 | 1.6824 | 3.46E-14 | 8.37E-13 | -- | ATP-dependent Clp protease proteolytic subunit |
| ZMO_RS04255 | 31626.89379 | 8957.584551 | 1.82 | 1.26E-06 | 7.80E-06 | -- | ATP-dependent Clp protease ATP-binding subunit ClpX |
| ZMO_RS04315 | 1649.795991 | 2877.080215 | -0.80232 | 0.00038 | 0.0012802 | -- | hemolysin D |
| ZMO_RS04320 | 1506.329425 | 2286.435359 | -0.60206 | 0.01041 | 0.023925 | emrB | MFS transporter |
| ZMO_RS04325 | 251.4009174 | 454.3060997 | -0.85367 | 0.00095 | 0.0029166 | -- | nucleoside transporter |
| ZMO_RS04335 | 692.4258513 | 1281.562672 | -0.88817 | 0.00015 | 0.00055802 | -- | NCS2 family permease |
| ZMO_RS04340 | 743.5992314 | 1210.341932 | -0.70282 | 0.00293 | 0.0079686 | -- | purine nucleoside permease |
| ZMO_RS04350 | 3310.227062 | 5804.73645 | -0.8103 | 0.00037 | 0.001252 | -- | hopanoid biosynthesis associated glycosyl transferase HpnI |
| ZMO_RS04365 | 498.9014705 | 796.9118609 | -0.67567 | 0.00427 | 0.010937 | -- | membrane protein |
| ZMO_RS04375 | 5946.05711 | 1526.357114 | 1.9618 | 3.76E-05 | 0.00016204 | -- | aldo/keto reductase |
| ZMO_RS04385 | 409.0616359 | 1230.363164 | -1.5887 | 0.00132 | 0.0039181 | -- | TonB-dependent receptor |
| ZMO_RS04415 | 1035.20367 | 1865.512686 | -0.84966 | 0.00078 | 0.002458 | -- | 5'-nucleotidase SurE |
| ZMO_RS04420 | 2751.159867 | 5020.322072 | -0.86774 | 0.00018 | 0.00066872 | -- | serine--tRNA ligase |
| ZMO_RS04430 | 69813.09061 | 983.7733036 | 6.149 | 3.07E-14 | 7.56E-13 | -- | heat-shock protein IbpA |
| ZMO_RS04460 | 751.0797238 | 401.8182553 | 0.90242 | 0.0002 | 0.00071956 | -- | peptide-methionine (S)-S-oxide reductase |
| ZMO_RS04470 | 3946.323005 | 32716.60854 | -3.0514 | 1.92E-38 | 4.42E-36 | -- | 5-methyltetrahydropteroyltriglutamate--homocysteine S-methyltransferase |
| ZMO_RS04500 | 966.683582 | 1645.986971 | -0.76784 | 0.0008 | 0.0025027 | -- | dihydropteroate synthase |
| ZMO_RS04510 | 1961.804569 | 1118.833616 | 0.81019 | 0.00038 | 0.0012724 | -- | FAD-binding oxidoreductase |
| ZMO_RS04515 | 2333.670714 | 6150.34666 | -1.3981 | 5.38E-10 | 6.42E-09 | -- | multidrug transporter |
| ZMO_RS04520 | 929.0534171 | 379.0071402 | 1.2935 | 1.53E-07 | 1.10E-06 | -- | DUF4186 domain-containing protein |
| ZMO_RS04530 | 188.7279405 | 75.86233585 | 1.3149 | 0.00014 | 0.00052421 | -- | DNA gyrase inhibitor YacG |
| ZMO_RS04535 | 291.5619261 | 844.335728 | -1.534 | 6.15E-10 | 7.16E-09 | -- | hypothetical protein |
| ZMO_RS04540 | 626.5705474 | 1571.508724 | -1.3266 | 1.53E-08 | 1.35E-07 | -- | septum formation inhibitor Maf |
| ZMO_RS04545 | 201.6444646 | 570.1458442 | -1.4995 | 7.72E-09 | 7.43E-08 | -- | translation initiation factor IF-1 |
| ZMO_RS04565 | 1973.645763 | 3163.868858 | -0.68083 | 0.00432 | 0.011003 | -- | ABC transporter permease |
| ZMO_RS04570 | 5504.439002 | 20486.04536 | -1.896 | 8.69E-17 | 3.33E-15 | -- | deoxyhypusine synthase |
| ZMO_RS04575 | 4453.077218 | 17509.31593 | -1.9752 | 8.19E-19 | 4.07E-17 | -- | type III PLP-dependent enzyme |
| ZMO_RS04580 | 4667.090961 | 2654.412574 | 0.81413 | 0.00021 | 0.0007689 | -- | alpha/beta hydrolase |
| ZMO_RS04590 | 1638.625252 | 1066.731649 | 0.61929 | 0.01436 | 0.031575 | -- | 7-cyano-7-deazaguanine synthase |
| ZMO_RS04595 | 11889.87698 | 19713.69399 | -0.72946 | 0.00081 | 0.0025232 | -- | ribonucleoside triphosphate reductase |
| ZMO_RS04605 | 1335.150157 | 2641.42113 | -0.98431 | 0.00017 | 0.00061198 | -- | anaerobic ribonucleoside-triphosphate reductase activating protein |
| ZMO_RS04610 | 1913.54171 | 3456.250998 | -0.85296 | 0.00021 | 0.00075916 | -- | ABC transporter ATP-binding protein |
| ZMO_RS04615 | 837.1750982 | 1919.106947 | -1.1968 | 1.14E-07 | 8.36E-07 | -- | ABC transporter permease |
| ZMO_RS04625 | 11533.89287 | 3119.319678 | 1.8866 | 1.77E-17 | 7.58E-16 | -- | class I SAM-dependent methyltransferase |
| ZMO_RS04630 | 29685.41837 | 3409.171372 | 3.1223 | 1.48E-41 | 4.53E-39 | -- | calcium-binding protein |
| ZMO_RS04635 | 3573.496937 | 7973.110689 | -1.1578 | 3.82E-07 | 2.56E-06 | -- | argininosuccinate synthase |
| ZMO_RS04645 | 10747.63066 | 15809.07673 | -0.55673 | 0.01554 | 0.03385 | -- | ribonucleotide-diphosphate reductase subunit alpha |
| ZMO_RS04650 | 880.5095714 | 312.1749274 | 1.496 | 0.00014 | 0.00052948 | -- | TonB-dependent receptor |
| ZMO_RS04660 | 831.4076437 | 363.8441807 | 1.1922 | 1.97E-06 | 1.16E-05 | -- | NUDIX hydrolase |
| ZMO_RS04665 | 1024.012124 | 437.0557077 | 1.2283 | 4.57E-07 | 3.05E-06 | -- | TPM domain-containing protein |
| ZMO_RS04670 | 2232.704769 | 1188.435632 | 0.90973 | 6.11E-05 | 0.00025129 | -- | LemA family protein |
| ZMO_RS04675 | 764.9356259 | 2228.273869 | -1.5425 | 1.07E-10 | 1.40E-09 | -- | large conductance mechanosensitive channel protein MscL |
| ZMO_RS04690 | 2982.553505 | 1498.744141 | 0.99279 | 6.99E-06 | 3.58E-05 | -- | phosphate ABC transporter substrate-binding protein PstS |
| ZMO_RS04710 | 11810.69138 | 6483.423725 | 0.86527 | 0.00016 | 0.00059308 | -- | phosphoribosylaminoimidazolesuccinocarboxamide synthase |
| ZMO_RS04725 | 1031.881104 | 1899.215485 | -0.88013 | 0.00016 | 0.00058514 | -- | CCA tRNA nucleotidyltransferase |
| ZMO_RS04735 | 895.5580823 | 1596.913109 | -0.83443 | 0.00071 | 0.0022663 | -- | dihydroneopterin aldolase |
| ZMO_RS04740 | 28852.32493 | 3413.537646 | 3.0793 | 8.39E-28 | 8.12E-26 | -- | superoxide dismutase |
| ZMO_RS04745 | 290.9288529 | 122.7535517 | 1.2449 | 0.02082 | 0.043341 | -- | phage shock protein operon transcriptional activator |
| ZMO_RS04755 | 1777.91402 | 264.8210975 | 2.7471 | 4.27E-08 | 3.38E-07 | -- | phage shock protein PspA |
| ZMO_RS04765 | 546.7805714 | 73.5693432 | 2.8938 | 0.00087 | 0.0026867 | -- | envelope stress response membrane protein PspC |
| ZMO_RS04770 | 309.7911929 | 83.293533 | 1.895 | 0.00069 | 0.002212 | -- | hypothetical protein |
| ZMO_RS04810 | 4254.25858 | 7171.914695 | -0.75345 | 0.00063 | 0.0020223 | -- | 30S ribosomal protein S16 |
| ZMO_RS04815 | 961.065129 | 2181.083504 | -1.1823 | 1.39E-07 | 1.01E-06 | -- | ribosome maturation factor RimM |
| ZMO_RS04820 | 3695.634877 | 5652.743681 | -0.61313 | 0.00404 | 0.010445 | -- | tRNA (guanosine(37)-N1)-methyltransferase TrmD |
| ZMO_RS04840 | 438.4844086 | 640.6942396 | -0.54711 | 0.02111 | 0.043698 | -- | cellulose synthase catalytic subunit (UDP-forming) |
| ZMO_RS04845 | 443.4174065 | 740.4223165 | -0.73968 | 0.00214 | 0.006026 | -- | cellulose biosynthesis cyclic di-GMP-binding regulatory protein BcsB |
| ZMO_RS04850 | 3239.584488 | 2244.3528 | 0.52951 | 0.01407 | 0.031048 | -- | cellulose synthase operon C domain-containing protein |
| ZMO_RS04860 | 1588.640551 | 707.9247664 | 1.1661 | 5.81E-07 | 3.79E-06 | -- | lytic murein transglycosylase |
| ZMO_RS04865 | 717.4540777 | 244.7196571 | 1.5518 | 2.15E-09 | 2.31E-08 | -- | septal ring lytic transglycosylase RlpA family protein |
| ZMO_RS04910 | 9250.077662 | 2367.642218 | 1.966 | 9.22E-09 | 8.78E-08 | -- | thioredoxin |
| ZMO_RS04930 | 538.9694011 | 348.7219177 | 0.62813 | 0.01102 | 0.024983 | -- | tRNA (adenosine(37)-N6)-threonylcarbamoyltransferase complex ATPase subunit type 1 TsaE |
| ZMO_RS04935 | 1210.938062 | 809.3872579 | 0.58122 | 0.01274 | 0.028512 | -- | two-component sensor histidine kinase |
| ZMO_RS04945 | 2193.046639 | 1246.601143 | 0.81494 | 0.00015 | 0.0005627 | -- | sensor histidine kinase |
| ZMO_RS04950 | 8060.073649 | 15270.34949 | -0.92187 | 0.00252 | 0.0069739 | -- | Lrp/AsnC family transcriptional regulator |
| ZMO_RS04960 | 558.3567827 | 1131.359303 | -1.0188 | 1.72E-05 | 8.07E-05 | -- | MFS transporter |
| ZMO_RS04970 | 10716.41403 | 3992.452067 | 1.4245 | 5.10E-11 | 7.10E-10 | -- | NAD(P)/FAD-dependent oxidoreductase |
| ZMO_RS04990 | 3046.822077 | 855.7893869 | 1.832 | 5.92E-11 | 8.00E-10 | -- | dihydropyrimidine dehydrogenase subunit A |
| ZMO_RS04995 | 7094.889007 | 3159.521642 | 1.1671 | 0.02413 | 0.049114 | -- | glutamate synthase large subunit |
| ZMO_RS05005 | 761.6205693 | 396.5946356 | 0.94141 | 0.00012 | 0.00044686 | -- | MerR family transcriptional regulator |
| ZMO_RS05010 | 1511.756836 | 800.2673135 | 0.91767 | 6.14E-05 | 0.00025172 | -- | integration host factor subunit alpha |
| ZMO_RS05035 | 2560.129291 | 1622.39779 | 0.65809 | 0.01061 | 0.024344 | -- | tRNA dihydrouridine synthase DusB |
| ZMO_RS05045 | 2904.992676 | 1057.566333 | 1.4578 | 1.59E-10 | 2.01E-09 | -- | hypothetical protein |
| ZMO_RS05050 | 6377.654361 | 2577.6398 | 1.307 | 2.79E-09 | 2.93E-08 | -- | CinA family protein |
| ZMO_RS05065 | 2333.661447 | 767.2834256 | 1.6048 | 0.00228 | 0.0063902 | -- | carbonic anhydrase |
| ZMO_RS05070 | 744.9513927 | 407.152389 | 0.87158 | 0.00348 | 0.0091884 | -- | hypothetical protein |
| ZMO_RS05080 | 5074.464834 | 3616.017144 | 0.48885 | 0.02076 | 0.043261 | -- | cytochrome b6 |
| ZMO_RS05095 | 8197.408148 | 5525.810033 | 0.56898 | 0.01329 | 0.029609 | -- | acetolactate synthase%2C large subunit%2C biosynthetic type |
| ZMO_RS05105 | 9871.380266 | 18037.21416 | -0.86965 | 0.00642 | 0.015704 | -- | ketol-acid reductoisomerase |
| ZMO_RS05115 | 247.371742 | 408.4101384 | -0.72334 | 0.00582 | 0.014382 | -- | CatA-like O-acetyltransferase |
| ZMO_RS05120 | 99.71598636 | 249.5847012 | -1.3236 | 1.16E-05 | 5.62E-05 | -- | teicoplanin resistance protein VanZ |
| ZMO_RS05125 | 3484.15471 | 12702.18078 | -1.8662 | 3.49E-16 | 1.19E-14 | -- | 50S ribosomal protein L31 |
| ZMO_RS05140 | 6257.103944 | 9216.512282 | -0.55873 | 0.01288 | 0.028768 | -- | outer membrane protein assembly factor BamA |
| ZMO_RS05175 | 10016.40415 | 17988.87041 | -0.84474 | 0.00013 | 0.00048041 | -- | elongation factor Ts |
| ZMO_RS05185 | 525.0784439 | 66.00448234 | 2.9919 | 6.31E-05 | 0.00025698 | -- | hypothetical protein |
| ZMO_RS05190 | 1783.465286 | 618.5133176 | 1.5278 | 1.77E-08 | 1.53E-07 | -- | aspartate/glutamate racemase family protein |
| ZMO_RS05195 | 991.7661775 | 1865.491797 | -0.91148 | 9.68E-05 | 0.00037542 | -- | CDP-diacylglycerol--serine O-phosphatidyltransferase |
| ZMO_RS05220 | 344.9897302 | 212.8693999 | 0.69658 | 0.02094 | 0.043534 | -- | ComF family protein |
| ZMO_RS05225 | 6593.044233 | 3310.925362 | 0.99371 | 4.42E-05 | 0.00018791 | -- | DNA recombination/repair protein RecA |
| ZMO_RS05235 | 671.6728497 | 453.362517 | 0.56709 | 0.02237 | 0.046041 | -- | ATPase |
| ZMO_RS05240 | 568.1088953 | 298.9661039 | 0.92619 | 0.00029 | 0.0010089 | -- | antibiotic biosynthesis monooxygenase |
| ZMO_RS05245 | 857.7835856 | 556.8885728 | 0.62323 | 0.01065 | 0.02438 | -- | beta-N-acetylhexosaminidase |
| ZMO_RS05250 | 2117.501097 | 1100.53349 | 0.94416 | 2.50E-05 | 0.00011234 | -- | succinylarginine dihydrolase |
| ZMO_RS05255 | 1923.088079 | 3712.825717 | -0.94909 | 1.95E-05 | 9.04E-05 | -- | translocation/assembly module TamB |
| ZMO_RS05270 | 2995.294744 | 1320.443544 | 1.1817 | 1.45E-07 | 1.04E-06 | -- | DNA-binding response regulator |
| ZMO_RS05275 | 1955.232579 | 913.8731133 | 1.0973 | 1.18E-06 | 7.41E-06 | -- | phosphoribosyl-AMP cyclohydrolase |
| ZMO_RS05280 | 1852.596964 | 1269.513651 | 0.54527 | 0.01278 | 0.028586 | -- | RNA methyltransferase |
| ZMO_RS05285 | 4300.518869 | 2148.183069 | 1.0014 | 0.00016 | 0.00057821 | -- | bifunctional ADP-dependent NAD(P)H-hydrate dehydratase/NAD(P)H-hydrate epimerase |
| ZMO_RS05315 | 9454.276837 | 1577.901275 | 2.583 | 2.92E-14 | 7.36E-13 | -- | lytic transglycosylase domain-containing protein |
| ZMO_RS05320 | 1924.534246 | 498.6871377 | 1.9483 | 3.00E-16 | 1.04E-14 | -- | formamidopyrimidine-DNA glycosylase |
| ZMO_RS05395 | 249.7950852 | 530.0934558 | -1.0855 | 8.05E-05 | 0.00031958 | -- | transcriptional regulator NrdR |
| ZMO_RS05400 | 488.8247415 | 1063.983399 | -1.1221 | 5.62E-06 | 2.94E-05 | -- | rRNA methyltransferase |
| ZMO_RS05405 | 800.6133995 | 342.5856674 | 1.2246 | 6.62E-07 | 4.25E-06 | -- | hypothetical protein |
| ZMO_RS05415 | 657.935802 | 285.0544899 | 1.2067 | 3.40E-06 | 1.88E-05 | -- | nitrilase |
| ZMO_RS05430 | 4765.255408 | 9970.080575 | -1.0651 | 2.32E-06 | 1.34E-05 | trkD | potassium transporter Kup |
| ZMO_RS05440 | 2012.284017 | 1383.753125 | 0.54025 | 0.01654 | 0.035545 | -- | glutathione-disulfide reductase |
| ZMO_RS05450 | 514.4944743 | 330.1734712 | 0.63993 | 0.01582 | 0.034201 | -- | hypothetical protein |
| ZMO_RS05505 | 12253.60331 | 32397.75716 | -1.4027 | 5.76E-10 | 6.74E-09 | -- | 30S ribosomal protein S6 |
| ZMO_RS05510 | 250.1613349 | 750.9453682 | -1.5858 | 8.82E-10 | 1.01E-08 | -- | 30S ribosomal protein S18 |
| ZMO_RS05515 | 9552.696219 | 25330.3547 | -1.4069 | 4.79E-10 | 5.83E-09 | -- | 50S ribosomal protein L9 |
| ZMO_RS05525 | 1401.896144 | 2523.622773 | -0.84812 | 0.0003 | 0.0010555 | -- | GTP cyclohydrolase I FolE |
| ZMO_RS05530 | 1717.196577 | 989.3645574 | 0.79548 | 0.00053 | 0.0017468 | -- | single-stranded-DNA-specific exonuclease RecJ |
| ZMO_RS05540 | 853.1735322 | 573.6522833 | 0.57266 | 0.01584 | 0.034202 | -- | glycosyltransferase family 1 protein |
| ZMO_RS05555 | 4444.881125 | 1555.980012 | 1.5143 | 1.91E-11 | 2.86E-10 | -- | transcriptional repressor |
| ZMO_RS05560 | 53136.04691 | 8502.64816 | 2.6437 | 6.90E-06 | 3.54E-05 | -- | zinc-dependent alcohol dehydrogenase |
| ZMO_RS05585 | 387.2798993 | 1038.240753 | -1.4227 | 5.96E-09 | 5.82E-08 | -- | 50S ribosomal protein L36 |
| ZMO_RS05605 | 283.7067417 | 881.7632968 | -1.636 | 8.76E-11 | 1.16E-09 | -- | DUF3617 domain-containing protein |
| ZMO_RS05610 | 286.0818879 | 481.5520546 | -0.75126 | 0.00412 | 0.010605 | -- | cytochrome c biosynthesis protein |
| ZMO_RS05625 | 557.0693802 | 844.6306428 | -0.60046 | 0.01502 | 0.032872 | -- | heme lyase CcmF/NrfE family subunit |
| ZMO_RS05670 | 6880.853792 | 10120.67838 | -0.55665 | 0.01737 | 0.037074 | -- | GMP synthase (glutamine-hydrolyzing) |
| ZMO_RS05675 | 1155.4358 | 3404.857551 | -1.5592 | 1.52E-11 | 2.32E-10 | -- | hypothetical protein |
| ZMO_RS05680 | 520.9299542 | 816.2074525 | -0.64785 | 0.01124 | 0.025407 | -- | acyl carrier protein |
| ZMO_RS05685 | 3516.417158 | 5874.703178 | -0.74041 | 0.00144 | 0.0042371 | -- | aminotransferase class V-fold PLP-dependent enzyme |
| ZMO_RS05690 | 3602.013857 | 891.993839 | 2.0137 | 2.66E-18 | 1.19E-16 | -- | uroporphyrinogen-III C-methyltransferase |
| ZMO_RS05715 | 8.492848398 | 32.83542357 | -1.9509 | 0.00514 | 0.01287 | -- | hypothetical protein |
| ZMO_RS05720 | 792.548487 | 1633.80698 | -1.0437 | 4.87E-05 | 0.00020436 | -- | endolytic transglycosylase MltG |
| ZMO_RS05725 | 8608.876223 | 16495.95899 | -0.93822 | 2.01E-05 | 9.26E-05 | -- | beta-ketoacyl-[acyl-carrier-protein] synthase II |
| ZMO_RS05730 | 7370.789161 | 14790.41582 | -1.0048 | 5.96E-06 | 3.09E-05 | -- | acyl carrier protein |
| ZMO_RS05755 | 3587.419606 | 5600.438367 | -0.64259 | 0.00335 | 0.0089106 | -- | glycosyl transferase group 1 protein |
| ZMO_RS05785 | 9447.798138 | 5879.276962 | 0.68434 | 0.00145 | 0.0042602 | -- | hypothetical protein |
| ZMO_RS05790 | 4198.313902 | 2434.473671 | 0.7862 | 0.00029 | 0.001025 | -- | ComF family protein |
| ZMO_RS05805 | 415.4375237 | 221.3190462 | 0.9085 | 0.00165 | 0.0047724 | -- | TonB-dependent receptor |
| ZMO_RS05815 | 2757.989927 | 4205.204559 | -0.60856 | 0.00952 | 0.022187 | -- | capsular polysaccharide biosynthesis protein |
| ZMO_RS05820 | 1213.431298 | 1780.582029 | -0.55326 | 0.02111 | 0.043698 | -- | hypothetical protein |
| ZMO_RS05830 | 684.8525922 | 446.000288 | 0.61875 | 0.01159 | 0.026166 | -- | lipoate--protein ligase B |
| ZMO_RS05835 | 4453.202106 | 1538.89042 | 1.533 | 8.61E-12 | 1.35E-10 | -- | pyrroline-5-carboxylate reductase |
| ZMO_RS05840 | 2828.630891 | 1305.200201 | 1.1158 | 1.31E-06 | 8.05E-06 | -- | hypothetical protein |
| ZMO_RS05845 | 1404.109308 | 758.1720601 | 0.88906 | 9.41E-05 | 0.00036642 | -- | hypothetical protein |
| ZMO_RS05855 | 925.6630942 | 2103.201185 | -1.184 | 3.88E-07 | 2.59E-06 | -- | fumarate hydratase |
| ZMO_RS05865 | 4942.072807 | 3051.70632 | 0.6955 | 0.0014 | 0.0041272 | -- | leucyl aminopeptidase |
| ZMO_RS05870 | 1913.751954 | 3745.87702 | -0.9689 | 1.04E-05 | 5.13E-05 | -- | LPS-assembly protein LptD |
| ZMO_RS05875 | 8484.575985 | 4570.649372 | 0.89244 | 7.30E-05 | 0.00029027 | -- | peptidyl-prolyl cis-trans isomerase |
| ZMO_RS05895 | 490.5346886 | 1069.712102 | -1.1248 | 2.74E-06 | 1.56E-05 | -- | polysaccharide biosynthesis protein |
| ZMO_RS05900 | 433.2689873 | 1112.623372 | -1.3606 | 1.07E-08 | 1.00E-07 | -- | hypothetical protein |
| ZMO_RS05905 | 1130.876406 | 2032.609936 | -0.84589 | 0.00021 | 0.00074016 | -- | nucleoside triphosphate hydrolase |
| ZMO_RS05915 | 644.9416365 | 1942.69494 | -1.5908 | 1.10E-11 | 1.69E-10 | -- | RNA methyltransferase |
| ZMO_RS05920 | 8196.54242 | 16178.51954 | -0.98099 | 1.09E-05 | 5.35E-05 | -- | IMP dehydrogenase |
| ZMO_RS05935 | 166.461703 | 343.5901372 | -1.0455 | 0.00017 | 0.00062055 | -- | phosphotransferase |
| ZMO_RS05940 | 1669.342425 | 901.6480743 | 0.88864 | 0.00119 | 0.0035822 | -- | RNase adaptor protein RapZ |
| ZMO_RS05945 | 642.297261 | 374.6893694 | 0.77755 | 0.0024 | 0.0066704 | -- | PTS fructose transporter subunit IIA |
| ZMO_RS05975 | 8087.775611 | 2795.214998 | 1.5328 | 0.00023 | 0.00080843 | -- | NAD(P)H:quinone oxidoreductase |
| ZMO_RS05985 | 440.7018808 | 261.162581 | 0.75485 | 0.00344 | 0.0091186 | -- | hypothetical protein |
| ZMO_RS05990 | 300.1023029 | 133.3552502 | 1.1702 | 8.13E-05 | 0.0003221 | -- | LysE family translocator |
| ZMO_RS05995 | 613.5626461 | 96.47482412 | 2.669 | 5.66E-21 | 3.59E-19 | -- | MFS transporter |
| ZMO_RS06000 | 1519.087655 | 563.9074256 | 1.4297 | 1.30E-08 | 1.19E-07 | -- | zinc metalloprotease |
| ZMO_RS06010 | 5790.58161 | 465.0321332 | 3.6383 | 3.65E-08 | 2.93E-07 | -- | aldo/keto reductase |
| ZMO_RS06015 | 2394.563144 | 5867.123352 | -1.2929 | 1.25E-08 | 1.15E-07 | -- | aminopeptidase N |
| ZMO_RS06020 | 1303.151659 | 2391.848024 | -0.87612 | 0.00024 | 0.00083328 | -- | EamA/RhaT family transporter |
| ZMO_RS06025 | 4690.188259 | 7400.739252 | -0.65802 | 0.00874 | 0.020545 | -- | low specificity L-threonine aldolase |
| ZMO_RS06035 | 1652.48021 | 955.1791639 | 0.79079 | 0.00052 | 0.0017003 | -- | competence/damage-inducible protein A |
| ZMO_RS06045 | 24186.48932 | 4701.87811 | 2.3629 | 1.13E-25 | 9.46E-24 | -- | BAX inhibitor (BI)-1/YccA family protein |
| ZMO_RS06055 | 33109.59815 | 100281.8994 | -1.5987 | 1.77E-12 | 3.14E-11 | -- | flagellar motor protein MotB |
| ZMO_RS06060 | 1868.79192 | 2678.31952 | -0.51922 | 0.01802 | 0.038197 | -- | ABC transporter ATP-binding protein |
| ZMO_RS06070 | 255.3138052 | 470.1021054 | -0.8807 | 0.02269 | 0.04665 | -- | hypothetical protein |
| ZMO_RS06075 | 13106.67595 | 23130.91892 | -0.81952 | 0.00022 | 0.00077756 | -- | 30S ribosomal protein S20 |
| ZMO_RS06085 | 296.2479869 | 793.9676815 | -1.4223 | 2.79E-08 | 2.32E-07 | -- | hypothetical protein |
| ZMO_RS06090 | 6195.543826 | 19891.6201 | -1.6829 | 5.59E-13 | 1.10E-11 | -- | hypothetical protein |
| ZMO_RS06110 | 15625.55837 | 31152.65137 | -0.99545 | 8.13E-06 | 4.09E-05 | -- | 30S ribosomal protein S4 |
| ZMO_RS06115 | 1382.409069 | 2041.878484 | -0.56271 | 0.014 | 0.030966 | -- | agmatine deiminase family protein |
| ZMO_RS06130 | 483.6567996 | 124.8356151 | 1.954 | 8.93E-06 | 4.47E-05 | -- | short-chain dehydrogenase |
| ZMO_RS06135 | 21263.86176 | 1517.791169 | 3.8084 | 4.80E-06 | 2.55E-05 | -- | hypothetical protein |
| ZMO_RS06160 | 75.70986988 | 34.35677436 | 1.1399 | 0.02034 | 0.04254 | -- | ethanolamine permease |
| ZMO_RS06165 | 331.2667285 | 55.93859028 | 2.5661 | 3.09E-06 | 1.73E-05 | -- | AraC family transcriptional regulator |
| ZMO_RS06175 | 152.2023674 | 78.9741533 | 0.94654 | 0.00871 | 0.020502 | -- | aspartate aminotransferase family protein |
| ZMO_RS06180 | 1782.893435 | 3779.991642 | -1.0842 | 2.28E-06 | 1.33E-05 | -- | GTPase Era |
| ZMO_RS06190 | 1047.931765 | 578.9039751 | 0.85615 | 0.00039 | 0.0013103 | -- | hypothetical protein |
| ZMO_RS06195 | 5076.429182 | 1874.482567 | 1.4373 | 1.12E-10 | 1.46E-09 | -- | DNA-binding response regulator |
| ZMO_RS06215 | 871.4306601 | 2502.066247 | -1.5217 | 1.51E-08 | 1.34E-07 | -- | diacylglycerol kinase |
| ZMO_RS06220 | 913.7741113 | 2171.044119 | -1.2485 | 6.73E-05 | 0.0002719 | -- | DUF2141 domain-containing protein |
| ZMO_RS06235 | 1001.699495 | 638.0137745 | 0.65079 | 0.00964 | 0.022425 | -- | N-formylglutamate amidohydrolase |
| ZMO_RS06240 | 12142.48585 | 6753.445277 | 0.84637 | 6.63E-05 | 0.00026943 | -- | hypothetical protein |
| ZMO_RS06245 | 556.9206339 | 249.5173761 | 1.1583 | 1.18E-05 | 5.68E-05 | -- | hypothetical protein |
| ZMO_RS06255 | 784.6374028 | 1787.076198 | -1.1875 | 4.74E-07 | 3.15E-06 | -- | hypothetical protein |
| ZMO_RS06260 | 2695.905022 | 709.7498861 | 1.9254 | 9.05E-17 | 3.40E-15 | -- | exodeoxyribonuclease III |
| ZMO_RS06265 | 1925.472357 | 442.5570867 | 2.1213 | 1.30E-13 | 2.85E-12 | -- | iron-sulfur cluster assembly accessory protein |
| ZMO_RS06270 | 4381.542695 | 1453.676794 | 1.5917 | 8.50E-06 | 4.27E-05 | -- | M23 family peptidase |
| ZMO_RS06280 | 7129.086569 | 4830.216721 | 0.56163 | 0.00745 | 0.017922 | -- | two-component response regulator |
| ZMO_RS06300 | 3239.119611 | 1907.084686 | 0.76423 | 0.00046 | 0.0015282 | -- | inositol monophosphatase |
| ZMO_RS06305 | 144.8755001 | 78.90743659 | 0.87658 | 0.01358 | 0.030141 | -- | (2Fe-2S)-binding protein |
| ZMO_RS06310 | 7124.771649 | 1211.903011 | 2.5556 | 0.00028 | 0.00099818 | -- | bacterioferritin |
| ZMO_RS06320 | 5562.855906 | 12188.79858 | -1.1317 | 5.35E-07 | 3.52E-06 | -- | MucR family transcriptional regulator |
| ZMO_RS06325 | 581.1893418 | 202.9559835 | 1.5178 | 2.42E-08 | 2.04E-07 | -- | ribosomal-protein-alanine N-acetyltransferase |
| ZMO_RS06330 | 1881.527387 | 579.2658195 | 1.6996 | 3.54E-10 | 4.40E-09 | -- | tRNA (adenosine(37)-N6)-threonylcarbamoyltransferase complex dimerization subunit type 1 TsaB |
| ZMO_RS06335 | 2949.243693 | 946.0333471 | 1.6404 | 6.80E-13 | 1.30E-11 | -- | NifU family protein |
| ZMO_RS06345 | 9762.052166 | 43199.59521 | -2.1458 | 9.96E-22 | 6.54E-20 | -- | ATP-dependent helicase |
| ZMO_RS06350 | 962.0764345 | 452.2992156 | 1.0889 | 7.60E-06 | 3.85E-05 | -- | 4-hydroxybenzoate octaprenyltransferase |
| ZMO_RS06355 | 746.9569032 | 1371.252915 | -0.8764 | 0.00024 | 0.00083848 | -- | 5-(carboxyamino)imidazole ribonucleotide mutase |
| ZMO_RS06360 | 1983.494139 | 3210.915696 | -0.69494 | 0.003 | 0.0081275 | -- | 5-(carboxyamino)imidazole ribonucleotide synthase |
| ZMO_RS06375 | 101238.2537 | 4507.468749 | 4.4893 | 2.52E-74 | 4.64E-71 | -- | ATP-dependent chaperone ClpB |
| ZMO_RS06385 | 1504.613981 | 409.0653989 | 1.879 | 2.21E-14 | 5.63E-13 | -- | DNA repair protein RadC |
| ZMO_RS06395 | 3079.358577 | 5146.014915 | -0.74083 | 0.00528 | 0.013191 | -- | TolC family protein |
| ZMO_RS06415 | 380.5410498 | 635.9266516 | -0.74081 | 0.00437 | 0.011118 | -- | DNA polymerase III subunit delta |
| ZMO_RS06420 | 761.8146917 | 1689.634758 | -1.1492 | 1.57E-06 | 9.51E-06 | -- | hypothetical protein |
| ZMO_RS06425 | 6541.374442 | 10507.69745 | -0.68378 | 0.00261 | 0.0072059 | -- | leucine--tRNA ligase |
| ZMO_RS06435 | 1060.649726 | 439.6486713 | 1.2705 | 0.00333 | 0.008875 | -- | LysE family translocator |
| ZMO_RS06440 | 331.3543445 | 205.1546103 | 0.69166 | 0.01533 | 0.033468 | -- | Lrp/AsnC family transcriptional regulator |
| ZMO_RS06455 | 1229.71069 | 2455.733549 | -0.99784 | 3.50E-05 | 0.00015369 | -- | FAD-binding oxidoreductase |
| ZMO_RS06470 | 3607.371226 | 7634.656534 | -1.0816 | 2.36E-06 | 1.36E-05 | -- | glycine--tRNA ligase subunit beta |
| ZMO_RS06475 | 2341.088846 | 3956.696868 | -0.75712 | 0.00114 | 0.0034494 | -- | glycine--tRNA ligase subunit alpha |
| ZMO_RS06480 | 381.1175348 | 221.7257569 | 0.78146 | 0.00425 | 0.010892 | -- | TraB/GumN family protein |
| ZMO_RS06495 | 1545.653754 | 186.7267352 | 3.0492 | 1.41E-09 | 1.54E-08 | -- | mannitol dehydrogenase family protein |
| ZMO_RS06500 | 1272.507029 | 683.8897106 | 0.89584 | 0.00015 | 0.00057229 | -- | deoxyribodipyrimidine photo-lyase |
| ZMO_RS06505 | 620.743124 | 1931.97333 | -1.638 | 4.17E-12 | 6.66E-11 | -- | purine nucleoside permease |
| ZMO_RS06510 | 1005.879689 | 2075.98888 | -1.0453 | 4.21E-06 | 2.27E-05 | -- | MFS transporter |
| ZMO_RS06525 | 816.7441334 | 4735.779336 | -2.5356 | 0.00094 | 0.0028919 | -- | MFS transporter |
| ZMO_RS06530 | 642.6205458 | 1739.650587 | -1.4368 | 0.00556 | 0.013799 | -- | hypothetical protein |
| ZMO_RS06540 | 1331.992045 | 606.8619701 | 1.1341 | 1.01E-06 | 6.40E-06 | -- | sulfurtransferase |
| ZMO_RS06555 | 650.5471886 | 268.3499408 | 1.2775 | 0.00076 | 0.0024172 | -- | TonB-dependent receptor |
| ZMO_RS06560 | 192.8625513 | 62.799973 | 1.6187 | 3.66E-06 | 2.01E-05 | -- | hypothetical protein |
| ZMO_RS06565 | 388.6373325 | 1783.723912 | -2.1984 | 2.22E-19 | 1.20E-17 | -- | membrane protein |
| ZMO_RS06570 | 3914.949567 | 1093.218382 | 1.8404 | 7.33E-15 | 1.98E-13 | -- | alpha/beta hydrolase |
| ZMO_RS06580 | 4202.808389 | 7692.165051 | -0.87204 | 0.00295 | 0.0080095 | -- | hypothetical protein |
| ZMO_RS06590 | 19945.76075 | 32441.02417 | -0.70174 | 0.00175 | 0.0050068 | -- | translational GTPase TypA |
| ZMO_RS06595 | 1218.081315 | 712.9972063 | 0.77264 | 0.01569 | 0.034074 | -- | hypothetical protein |
| ZMO_RS06620 | 3969.689612 | 2774.555383 | 0.51677 | 0.01854 | 0.039264 | -- | electron transfer flavoprotein subunit alpha |
| ZMO_RS06625 | 3248.973559 | 1909.467525 | 0.76681 | 0.00059 | 0.0018965 | -- | electron transfer flavoprotein subunit beta/FixA family protein |
| ZMO_RS06635 | 872.9968972 | 460.5644987 | 0.92257 | 0.0002 | 0.00072419 | -- | autotransporter domain-containing protein |
| ZMO_RS06645 | 1656.806059 | 875.9069922 | 0.91956 | 0.00012 | 0.00044324 | -- | helicase UvrD |
| ZMO_RS06650 | 2995.927632 | 342.1528028 | 3.1303 | 2.58E-37 | 4.74E-35 | -- | HD domain-containing protein |
| ZMO_RS06665 | 5047.954325 | 3213.746434 | 0.65144 | 0.00207 | 0.0058779 | -- | 3-deoxy-8-phosphooctulonate synthase |
| ZMO_RS06670 | 1611.848925 | 1134.501399 | 0.50666 | 0.02107 | 0.043698 | -- | 3-deoxy-manno-octulosonate cytidylyltransferase |
| ZMO_RS06675 | 18128.65701 | 31180.5132 | -0.78237 | 0.00461 | 0.011679 | -- | membrane protein |
| ZMO_RS06680 | 19571.93783 | 37957.13596 | -0.95558 | 2.35E-05 | 0.00010622 | -- | porin family protein |
| ZMO_RS06700 | 1426.070593 | 568.9701756 | 1.3256 | 0.00029 | 0.0010259 | -- | ATP:cob(I)alamin adenosyltransferase |
| ZMO_RS06715 | 434.4678842 | 1515.16329 | -1.8022 | 5.50E-08 | 4.28E-07 | -- | histidine triad nucleotide-binding protein |
| ZMO_RS06725 | 834.097419 | 1527.526842 | -0.87291 | 0.00223 | 0.0062425 | -- | imidazole glycerol phosphate synthase cyclase subunit |
| ZMO_RS06730 | 1092.901448 | 1876.957394 | -0.78023 | 0.0081 | 0.01925 | -- | 1-(5-phosphoribosyl)-5-((5-phosphoribosylamino)methylideneamino)imidazole-4-carboxamide isomerase |
| ZMO_RS06735 | 922.059942 | 1613.614523 | -0.80736 | 0.001 | 0.0030369 | -- | imidazole glycerol phosphate synthase subunit HisH |
| ZMO_RS06755 | 1623.557909 | 951.1217909 | 0.77146 | 0.00073 | 0.0023161 | -- | hypothetical protein |
| ZMO_RS06760 | 1878.160459 | 6024.295106 | -1.6815 | 3.78E-13 | 7.64E-12 | -- | inorganic diphosphatase |
| ZMO_RS06765 | 2498.551453 | 4011.279908 | -0.68297 | 0.00246 | 0.0068187 | -- | histidine--tRNA ligase |
| ZMO_RS06770 | 1860.031986 | 3295.547217 | -0.82519 | 0.00023 | 0.00083328 | -- | peptide chain release factor 1 |
| ZMO_RS06775 | 639.6661286 | 1371.258964 | -1.1001 | 4.05E-06 | 2.20E-05 | -- | peptide chain release factor N(5)-glutamine methyltransferase |
| ZMO_RS06790 | 6543.65224 | 12836.18048 | -0.97205 | 1.47E-05 | 7.02E-05 | -- | phenylalanine--tRNA ligase subunit beta |
| ZMO_RS06795 | 3829.267863 | 7224.572236 | -0.91584 | 4.62E-05 | 0.00019529 | -- | phenylalanine--tRNA ligase subunit alpha |
| ZMO_RS06800 | 5808.015547 | 17410.30278 | -1.5838 | 2.26E-12 | 3.92E-11 | -- | 50S ribosomal protein L20 |
| ZMO_RS06805 | 1347.630001 | 4423.627228 | -1.7148 | 1.08E-05 | 5.30E-05 | -- | histidinol phosphate phosphatase |
| ZMO_RS06810 | 5114.959295 | 12812.28037 | -1.3247 | 4.70E-09 | 4.75E-08 | -- | ribose-phosphate pyrophosphokinase |
| ZMO_RS06815 | 1410.820853 | 2081.63483 | -0.56118 | 0.02274 | 0.046697 | -- | DUF2093 domain-containing protein |
| ZMO_RS06835 | 596.2228954 | 1580.886974 | -1.4068 | 2.65E-09 | 2.80E-08 | -- | RND transporter |
| ZMO_RS06840 | 717.8525837 | 1583.71349 | -1.1416 | 1.31E-06 | 8.05E-06 | -- | nodulation protein |
| ZMO_RS06845 | 871.5904936 | 1805.075959 | -1.0503 | 6.24E-06 | 3.22E-05 | -- | acriflavin resistance protein |
| ZMO_RS06850 | 845.4787102 | 1282.913722 | -0.60158 | 0.00731 | 0.017641 | -- | efflux RND transporter periplasmic adaptor subunit |
| ZMO_RS06855 | 3584.149549 | 2309.208466 | 0.63423 | 0.00349 | 0.0091943 | -- | KpsF/GutQ family sugar-phosphate isomerase |
| ZMO_RS06860 | 764.7108539 | 1266.895874 | -0.72831 | 0.00139 | 0.0041016 | -- | phosphoribosylformylglycinamidine synthase subunit PurS |
| ZMO_RS06865 | 3053.643889 | 4959.508827 | -0.69967 | 0.00207 | 0.0058779 | -- | phosphoribosylformylglycinamidine synthase subunit PurQ |
| ZMO_RS06870 | 2870.142569 | 462.6794393 | 2.633 | 2.76E-07 | 1.89E-06 | -- | hypothetical protein |
| ZMO_RS06890 | 243.1955054 | 443.3340668 | -0.86628 | 0.00252 | 0.0069739 | -- | ferrous iron transport protein A |
| ZMO_RS06905 | 4566.92396 | 2004.83118 | 1.1877 | 9.21E-08 | 6.88E-07 | -- | cobaltochelatase subunit CobT |
| ZMO_RS06910 | 3477.255492 | 1523.789053 | 1.1903 | 9.37E-08 | 6.97E-07 | -- | cobaltochelatase subunit CobS |
| ZMO_RS06915 | 2073.945984 | 781.3971794 | 1.4083 | 1.38E-09 | 1.51E-08 | -- | J domain-containing protein |
| ZMO_RS06925 | 2097.899172 | 3110.872437 | -0.56837 | 0.01442 | 0.031656 | -- | squalene--hopene cyclase |
| ZMO_RS06930 | 1239.66298 | 357.4765152 | 1.794 | 1.91E-13 | 3.98E-12 | -- | BolA family transcriptional regulator |
| ZMO_RS06955 | 715.245555 | 1079.540135 | -0.59391 | 0.00847 | 0.020073 | -- | 16S rRNA (uracil(1498)-N(3))-methyltransferase |
| ZMO_RS06965 | 25965.82354 | 11544.78848 | 1.1694 | 5.27E-08 | 4.12E-07 | -- | amidophosphoribosyltransferase |
| ZMO_RS06970 | 547.4803659 | 833.6526256 | -0.60664 | 0.01623 | 0.034977 | -- | NAD(P)-dependent oxidoreductase |
| ZMO_RS06975 | 524.2373077 | 1385.406428 | -1.402 | 1.36E-08 | 1.23E-07 | -- | RNA pyrophosphohydrolase |
| ZMO_RS06980 | 442.7929024 | 1460.306352 | -1.7216 | 1.04E-12 | 1.94E-11 | -- | DUF481 domain-containing protein |
| ZMO_RS06995 | 781.9759453 | 259.4159402 | 1.5919 | 5.28E-10 | 6.35E-09 | -- | YnfA family protein |
| ZMO_RS07000 | 281.0428385 | 450.8511364 | -0.68186 | 0.00729 | 0.017617 | -- | aminoglycoside phosphotransferase |
| ZMO_RS07010 | 2607.83767 | 931.4194234 | 1.4854 | 2.27E-10 | 2.86E-09 | -- | pyruvate formate-lyase 1-activating enzyme |
| ZMO_RS07015 | 36746.33019 | 9533.226405 | 1.9466 | 2.51E-12 | 4.23E-11 | -- | formate C-acetyltransferase |
| ZMO_RS07025 | 5952.211186 | 8785.705193 | -0.56173 | 0.01331 | 0.02962 | -- | cytochrome ubiquinol oxidase subunit I |
| ZMO_RS07045 | 427.4739977 | 244.3495839 | 0.80689 | 0.00267 | 0.0073703 | -- | LysR family transcriptional regulator |
| ZMO_RS07050 | 3220.19365 | 287.0855927 | 3.4876 | 8.39E-08 | 6.37E-07 | -- | NAD(P)-dependent oxidoreductase |
| ZMO_RS07055 | 133.1112791 | 41.24603656 | 1.6903 | 2.20E-05 | 0.00010046 | -- | MFS transporter |
| ZMO_RS07060 | 122.9178932 | 53.20632592 | 1.208 | 0.00269 | 0.0074102 | -- | 3-oxoacyl-ACP reductase |
| ZMO_RS07065 | 428.943584 | 267.0286749 | 0.68379 | 0.00974 | 0.022606 | -- | alpha/beta hydrolase |
| ZMO_RS07100 | 6048.261559 | 2939.449496 | 1.041 | 2.21E-06 | 1.29E-05 | -- | antibiotic biosynthesis monooxygenase |
| ZMO_RS07115 | 309.650396 | 169.0501295 | 0.87319 | 0.00283 | 0.0077332 | -- | DNA replication and repair protein RecF |
| ZMO_RS07120 | 1862.310856 | 752.4786979 | 1.3074 | 0.01253 | 0.028115 | -- | bacterioferritin |
| ZMO_RS07125 | 329.7571212 | 89.69832742 | 1.8783 | 1.02E-07 | 7.58E-07 | -- | hypothetical protein |
| ZMO_RS07130 | 13306.94079 | 778.2691627 | 4.0958 | 1.78E-16 | 6.43E-15 | -- | excinuclease ABC subunit A |
| ZMO_RS07135 | 2773.773343 | 1563.264433 | 0.82729 | 0.00459 | 0.011646 | -- | toll/interleukin-1 receptor domain-containing protein |
| ZMO_RS07150 | 3831.436662 | 2560.56754 | 0.58142 | 0.01575 | 0.034088 | -- | alanine racemase |
| ZMO_RS07165 | 102111.157 | 63869.72815 | 0.67694 | 0.00923 | 0.021574 | -- | alcohol dehydrogenase |
| ZMO_RS07175 | 4267.662501 | 7445.873221 | -0.80299 | 0.00032 | 0.001113 | -- | hopanoid biosynthesis-associated RND transporter HpnN |
| ZMO_RS07180 | 1739.233595 | 2992.459409 | -0.78288 | 0.00207 | 0.0058814 | -- | homoserine kinase |
| ZMO_RS07185 | 182.8718135 | 425.956662 | -1.2199 | 1.77E-05 | 8.27E-05 | -- | ribonuclease H |
| ZMO_RS07190 | 1418.195282 | 916.4557443 | 0.62992 | 0.00618 | 0.015211 | -- | peptidase |
| ZMO_RS07195 | 3272.245197 | 727.2227224 | 2.1698 | 3.13E-20 | 1.85E-18 | -- | TIGR01244 family phosphatase |
| ZMO_RS07200 | 412.6574546 | 1231.021155 | -1.5768 | 3.81E-11 | 5.38E-10 | -- | alkaline phosphatase family protein |
| ZMO_RS07205 | 4651.466057 | 7385.674328 | -0.66704 | 0.00384 | 0.0099814 | -- | pyruvate dehydrogenase E1 component subunit beta |
| ZMO_RS07210 | 4643.614917 | 6818.740358 | -0.55426 | 0.01477 | 0.032403 | -- | pyruvate dehydrogenase E1 component subunit alpha |
| ZMO_RS07255 | 16316.13211 | 11014.83213 | 0.56685 | 0.00822 | 0.019517 | -- | carbamoyl phosphate synthase large subunit |
| ZMO_RS07260 | 7808.187368 | 3859.299572 | 1.0166 | 4.45E-06 | 2.40E-05 | -- | carbamoyl-phosphate synthase small subunit |
| ZMO_RS07265 | 2006.388314 | 3172.086037 | -0.66083 | 0.00351 | 0.009237 | -- | GatB/YqeY domain-containing protein |
| ZMO_RS07275 | 13802.17839 | 8191.244537 | 0.75274 | 0.00693 | 0.016856 | -- | RNA polymerase sigma factor RpoD |
| ZMO_RS07290 | 4235.400528 | 7965.046198 | -0.91118 | 4.05E-05 | 0.00017301 | -- | membrane protein insertase YidC |
| ZMO_RS07320 | 1738.443994 | 3133.643245 | -0.85004 | 0.00017 | 0.00062055 | -- | succinyl-diaminopimelate desuccinylase |
| ZMO_RS07325 | 219.9697736 | 1373.216954 | -2.6422 | 2.72E-25 | 2.17E-23 | -- | hypothetical protein |
| ZMO_RS07335 | 2002.303087 | 759.3874933 | 1.3988 | 0.00159 | 0.0046115 | -- | transglycosylase |
| ZMO_RS07340 | 533.553453 | 189.6574414 | 1.4922 | 3.28E-06 | 1.82E-05 | -- | hypothetical protein |
| ZMO_RS07345 | 13187.27807 | 3563.829709 | 1.8876 | 3.09E-17 | 1.26E-15 | -- | Tim44 domain-containing protein |
| ZMO_RS07400 | 203.1493108 | 694.908222 | -1.7743 | 5.79E-12 | 9.17E-11 | -- | uracil-DNA glycosylase |
| ZMO_RS07410 | 537.7298276 | 815.3761263 | -0.60058 | 0.0157 | 0.034074 | -- | serine hydrolase |
| ZMO_RS07415 | 2327.470748 | 3762.226108 | -0.69282 | 0.00218 | 0.0061397 | -- | phosphoenolpyruvate--protein phosphotransferase |
| ZMO_RS07420 | 3161.576048 | 5222.579134 | -0.72412 | 0.00138 | 0.004076 | -- | 2-nitropropane dioxygenase |
| ZMO_RS07425 | 5254.406827 | 8903.991371 | -0.76092 | 0.00089 | 0.0027541 | -- | aspartate kinase |
| ZMO_RS07430 | 1423.899629 | 585.9800339 | 1.2809 | 4.06E-08 | 3.23E-07 | -- | bifunctional 2-polyprenyl-6-hydroxyphenol methylase/3-demethylubiquinol 3-O-methyltransferase UbiG |
| ZMO_RS07455 | 27024.3894 | 7608.171496 | 1.8286 | 2.52E-16 | 8.89E-15 | -- | ATP-dependent metalloprotease |
| ZMO_RS07465 | 3463.125239 | 2253.86504 | 0.61967 | 0.005 | 0.012562 | -- | gamma-glutamyl-phosphate reductase |
| ZMO_RS07480 | 1147.835745 | 2482.106305 | -1.1126 | 4.94E-07 | 3.27E-06 | -- | 23S rRNA (pseudouridine(1915)-N(3))-methyltransferase RlmH |
| ZMO_RS07495 | 1776.053117 | 937.7468388 | 0.9214 | 4.99E-05 | 0.00020842 | -- | disulfide bond formation protein B |
| ZMO_RS07515 | 347.3959659 | 526.6983071 | -0.6004 | 0.00987 | 0.022819 | -- | hypothetical protein |
| ZMO_RS07525 | 4137.878914 | 2364.742515 | 0.80721 | 0.00014 | 0.00054102 | -- | protein disulfide-isomerase |
| ZMO_RS07530 | 597.8656255 | 329.8649725 | 0.85795 | 0.00494 | 0.012411 | -- | DUF721 domain-containing protein |
| ZMO_RS07545 | 823.7426722 | 218.0617202 | 1.9175 | 1.70E-13 | 3.64E-12 | -- | LOG family protein |
| ZMO_RS07565 | 722.1993899 | 1049.104409 | -0.53869 | 0.02319 | 0.047508 | ansB | asparaginase |
| ZMO_RS07570 | 3323.612561 | 6379.762477 | -0.94075 | 3.50E-05 | 0.00015369 | -- | phosphoserine transaminase |
| ZMO_RS07575 | 1891.763909 | 6153.417204 | -1.7017 | 7.58E-14 | 1.72E-12 | -- | phosphoglycerate dehydrogenase |
| ZMO_RS07580 | 3829.136038 | 7131.322655 | -0.89715 | 8.20E-05 | 0.00032402 | -- | ATP phosphoribosyltransferase regulatory subunit |
| ZMO_RS07585 | 4021.549095 | 9144.541724 | -1.1852 | 2.24E-07 | 1.57E-06 | -- | adenylosuccinate synthetase |
| ZMO_RS07590 | 2024.722431 | 924.0601817 | 1.1317 | 3.06E-06 | 1.73E-05 | -- | folate-binding protein |
| ZMO_RS07595 | 3526.470434 | 1770.054537 | 0.99443 | 4.92E-06 | 2.60E-05 | -- | dihydroorotase |
| ZMO_RS07600 | 6510.233971 | 803.8315776 | 3.0177 | 3.57E-25 | 2.73E-23 | -- | molecular chaperone DnaJ |
| ZMO_RS07620 | 4279.891962 | 13045.94151 | -1.608 | 1.78E-06 | 1.07E-05 | -- | TonB-dependent receptor |
| ZMO_RS07630 | 2037.132594 | 183.9755454 | 3.469 | 1.05E-41 | 3.86E-39 | -- | zinc-binding alcohol dehydrogenase family protein |
| ZMO_RS07635 | 1126.105582 | 99.42746329 | 3.5016 | 9.52E-38 | 1.94E-35 | -- | transcriptional regulator |
| ZMO_RS07640 | 1836.891071 | 678.0482723 | 1.4378 | 1.11E-09 | 1.24E-08 | -- | GTP cyclohydrolase II |
| ZMO_RS07650 | 3367.39707 | 1048.813276 | 1.6829 | 1.34E-13 | 2.90E-12 | -- | hypothetical protein |
| ZMO_RS07665 | 2437.904333 | 1308.170206 | 0.89809 | 5.14E-05 | 0.00021381 | -- | ubiquinone biosynthesis protein UbiH |
| ZMO_RS07670 | 4096.452887 | 732.5175582 | 2.4834 | 5.63E-14 | 1.31E-12 | -- | peptidase S16 |
| ZMO_RS07675 | 9188.207889 | 429.321336 | 4.4197 | 7.82E-70 | 7.19E-67 | -- | thioredoxin |
| ZMO_RS07680 | 624.6765526 | 1220.296665 | -0.96605 | 7.19E-05 | 0.00028849 | -- | orotate phosphoribosyltransferase |
| ZMO_RS07690 | 177.5978363 | 391.1811844 | -1.1392 | 5.67E-05 | 0.00023414 | -- | holo-ACP synthase |
| ZMO_RS07695 | 2066.891448 | 3260.06192 | -0.65744 | 0.00431 | 0.011003 | -- | signal peptidase I |
| ZMO_RS07705 | 2540.197902 | 4619.720564 | -0.86286 | 4.47E-05 | 0.0001898 | -- | FKBP-type peptidyl-prolyl cis-trans isomerase |
| ZMO_RS07715 | 1146.807576 | 3323.572898 | -1.5351 | 3.20E-11 | 4.71E-10 | -- | biopolymer transporter ExbD |
| ZMO_RS07720 | 746.2619082 | 2147.467055 | -1.5249 | 1.36E-10 | 1.74E-09 | -- | biopolymer transporter ExbD |
| ZMO_RS07725 | 2235.335737 | 5436.034427 | -1.2821 | 1.66E-08 | 1.44E-07 | -- | MotA/TolQ/ExbB proton channel family protein |
| ZMO_RS07730 | 2851.905755 | 6792.15386 | -1.2519 | 3.09E-08 | 2.52E-07 | -- | energy transducer TonB |
| ZMO_RS07735 | 568.0037116 | 1401.448602 | -1.3029 | 8.83E-08 | 6.63E-07 | -- | hypothetical protein |
| ZMO_RS07745 | 9593.577184 | 4732.700551 | 1.0194 | 0.00329 | 0.0087754 | -- | DNA-directed RNA polymerase subunit omega |
| ZMO_RS07750 | 14324.67573 | 2110.858522 | 2.7626 | 2.72E-33 | 3.85E-31 | -- | VOC family protein |
| ZMO_RS07755 | 2414.592807 | 1400.681874 | 0.78565 | 0.00041 | 0.0013764 | -- | S-(hydroxymethyl)glutathione dehydrogenase/class III alcohol dehydrogenase |
| ZMO_RS07775 | 1150.661509 | 452.5952611 | 1.3462 | 1.63E-08 | 1.42E-07 | -- | ATP-dependent Clp protease adaptor ClpS |
| ZMO_RS07780 | 23293.72873 | 8262.224971 | 1.4953 | 3.69E-12 | 5.95E-11 | -- | pyridoxal phosphate-dependent aminotransferase |
| ZMO_RS07800 | 33045.03074 | 11404.85081 | 1.5348 | 8.22E-05 | 0.00032402 | -- | peroxiredoxin |
| ZMO_RS07815 | 939.9973698 | 510.5987319 | 0.88047 | 0.0004 | 0.0013497 | -- | glycosyl transferase family 1 |
| ZMO_RS07820 | 2137.328592 | 924.1756109 | 1.2096 | 1.75E-07 | 1.24E-06 | -- | DNA-binding response regulator |
| ZMO_RS07830 | 2352.964408 | 3669.606704 | -0.64115 | 0.00518 | 0.012953 | -- | DUF3297 domain-containing protein |
| ZMO_RS07835 | 3554.363327 | 5784.035846 | -0.70249 | 0.00126 | 0.0037594 | -- | elongation factor 4 |
| ZMO_RS07840 | 1219.229282 | 792.4895534 | 0.62151 | 0.01897 | 0.040068 | -- | biotin transporter BioY |
| ZMO_RS07845 | 1931.862278 | 3548.367146 | -0.87716 | 0.00011 | 0.00043635 | -- | methionine synthase |
| ZMO_RS07850 | 520.0711054 | 1161.316091 | -1.159 | 8.21E-07 | 5.24E-06 | -- | 5-methyltetrahydrofolate--homocysteine methyltransferase |
| ZMO_RS07855 | 383.2531334 | 1126.961029 | -1.5561 | 1.48E-10 | 1.89E-09 | -- | methylenetetrahydrofolate reductase [NAD(P)H] |
| ZMO_RS07860 | 579.1014708 | 1476.720221 | -1.3505 | 1.15E-08 | 1.06E-07 | -- | methyltransferase domain-containing protein |
| ZMO_RS07865 | 1166.751286 | 2067.516607 | -0.8254 | 0.00017 | 0.00062055 | -- | ABC transporter |
| ZMO_RS07870 | 614.2144173 | 371.0214416 | 0.72724 | 0.00366 | 0.0095594 | -- | proteinase IV |
| ZMO_RS07885 | 9279.482616 | 3442.384405 | 1.4306 | 2.17E-05 | 9.90E-05 | -- | ferredoxin--NADP reductase |
| ZMO_RS07890 | 5613.418737 | 986.1784483 | 2.509 | 1.97E-27 | 1.81E-25 | -- | NAD-dependent succinate-semialdehyde dehydrogenase |
| ZMO_RS07900 | 2650.30015 | 6741.003325 | -1.3468 | 3.34E-09 | 3.46E-08 | -- | 2-keto-3-deoxygluconate permease |
| ZMO_RS07925 | 734.3523812 | 470.0010369 | 0.64381 | 0.00866 | 0.020412 | -- | 16S rRNA (cytidine(1402)-2'-O)-methyltransferase |
| ZMO_RS07935 | 1362.274378 | 2830.434373 | -1.055 | 3.94E-06 | 2.15E-05 | -- | hypothetical protein |
| ZMO_RS07940 | 1700.960824 | 3660.508326 | -1.1057 | 1.33E-06 | 8.12E-06 | -- | peptidoglycan-binding protein |
| ZMO_RS07945 | 917.617137 | 594.5339724 | 0.62613 | 0.0089 | 0.020899 | -- | zinc transporter ZntB |
| ZMO_RS07950 | 3146.156453 | 2195.803821 | 0.51884 | 0.01706 | 0.036463 | -- | UTP--glucose-1-phosphate uridylyltransferase |
| ZMO_RS07975 | 911.7098764 | 346.3437744 | 1.3964 | 3.72E-07 | 2.51E-06 | -- | NAD(P)H quinone oxidoreductase |
| ZMO_RS07980 | 3004.688414 | 968.928709 | 1.6328 | 6.11E-13 | 1.18E-11 | -- | aldo/keto reductase |
| ZMO_RS07990 | 591.0189763 | 284.5004799 | 1.0548 | 3.83E-05 | 0.00016467 | -- | hypothetical protein |
| ZMO_RS07995 | 11218.99643 | 4402.548097 | 1.3495 | 0.00856 | 0.02023 | -- | aminopeptidase N |
| ZMO_RS08005 | 464.5787369 | 814.1529557 | -0.80938 | 0.00099 | 0.0030108 | -- | DUF475 domain-containing protein |
| ZMO_RS08030 | 1448.519082 | 2766.251993 | -0.93335 | 3.82E-05 | 0.00016445 | -- | hypothetical protein |
| ZMO_RS08050 | 1495.315936 | 898.1247994 | 0.73546 | 0.00586 | 0.014466 | -- | hypothetical protein |
| ZMO_RS08065 | 186.697636 | 95.82089383 | 0.96229 | 0.00309 | 0.0083504 | -- | LysR family transcriptional regulator |
| ZMO_RS08075 | 2561.256777 | 6823.130164 | -1.4136 | 5.45E-10 | 6.42E-09 | -- | hypothetical protein |
| ZMO_RS08080 | 2713.276143 | 4281.709744 | -0.65815 | 0.00429 | 0.010958 | -- | 3-phosphoshikimate 1-carboxyvinyltransferase |
| ZMO_RS08085 | 4785.347634 | 7212.300972 | -0.59184 | 0.00854 | 0.020198 | -- | (d)CMP kinase |
| ZMO_RS08090 | 54293.31049 | 112010.0556 | -1.0448 | 2.72E-06 | 1.55E-05 | -- | 30S ribosomal protein S1 |
| ZMO_RS08100 | 2223.166764 | 1387.428653 | 0.6802 | 0.00186 | 0.0052901 | -- | integration host factor subunit beta |
| ZMO_RS08110 | 1730.223145 | 1150.434369 | 0.58878 | 0.00669 | 0.016321 | -- | haloacid dehalogenase |
| ZMO_RS08115 | 389.216004 | 115.0770735 | 1.758 | 2.90E-09 | 3.03E-08 | -- | sugar O-acetyltransferase |
| ZMO_RS08165 | 167.3214104 | 53.1747171 | 1.6538 | 9.81E-06 | 4.87E-05 | -- | nif-specific transcriptional activator NifA |
| ZMO_RS08170 | 559.7418068 | 213.0522677 | 1.3936 | 0.0093 | 0.021709 | -- | nitrogenase cofactor biosynthesis protein NifB |
| ZMO_RS08175 | 147.1213895 | 321.1809176 | -1.1264 | 0.00059 | 0.0018965 | -- | ferredoxin |
| ZMO_RS08190 | 263.397653 | 160.9811845 | 0.71035 | 0.02379 | 0.048586 | -- | SIR2 family protein |
| ZMO_RS08200 | 163.799039 | 35.08241575 | 2.2231 | 1.31E-08 | 1.19E-07 | -- | nitrogenase iron protein |
| ZMO_RS08205 | 299.8869571 | 44.8853973 | 2.7401 | 1.58E-07 | 1.12E-06 | -- | nitrogenase molybdenum-iron protein alpha chain |
| ZMO_RS08210 | 291.164538 | 72.64465158 | 2.0029 | 4.86E-10 | 5.88E-09 | -- | nitrogenase molybdenum-iron protein subunit beta |
| ZMO_RS08215 | 160.6073169 | 70.96686547 | 1.1783 | 0.0032 | 0.0085711 | -- | nitrogenase iron-molybdenum cofactor biosynthesis protein NifE |
| ZMO_RS08220 | 146.9941853 | 65.82591802 | 1.159 | 0.00183 | 0.0052191 | -- | nitrogenase iron-molybdenum cofactor biosynthesis protein NifN |
| ZMO_RS08245 | 40.17821756 | 13.84584945 | 1.537 | 0.01563 | 0.033987 | -- | iron-sulfur cluster assembly accessory protein |
| ZMO_RS08260 | 48.72404911 | 17.26281426 | 1.497 | 0.0169 | 0.03616 | -- | homocitrate synthase |
| ZMO_RS08275 | 495.9129959 | 79.87558178 | 2.6343 | 7.02E-05 | 0.00028253 | -- | transporter |
| ZMO_RS08280 | 1147.182374 | 272.0088353 | 2.0764 | 1.12E-05 | 5.49E-05 | -- | cysteine hydrolase |
| ZMO_RS08285 | 1442.473546 | 437.114505 | 1.7225 | 1.48E-12 | 2.67E-11 | -- | sigma E positive regulator RseC/MucC |
| ZMO_RS08290 | 9086.962935 | 3538.249032 | 1.3608 | 2.64E-07 | 1.82E-06 | -- | FAD:protein FMN transferase |
| ZMO_RS08300 | 741.5009254 | 2242.394189 | -1.5965 | 1.99E-11 | 2.95E-10 | -- | PhnA protein |
| ZMO_RS08310 | 70.90480082 | 15.9605081 | 2.1514 | 7.25E-05 | 0.00028886 | -- | iron ABC transporter permease |
| ZMO_RS08315 | 319.2683658 | 89.57981145 | 1.8335 | 5.03E-08 | 3.97E-07 | -- | ABC transporter substrate-binding protein |
| ZMO_RS08320 | 170.0987909 | 55.73572811 | 1.6097 | 0.0243 | 0.049345 | -- | hypothetical protein |
| ZMO_RS08325 | 102.7805043 | 52.70429719 | 0.96357 | 0.01756 | 0.037406 | -- | DUF2023 domain-containing protein |
| ZMO_RS08335 | 232.3595114 | 688.241194 | -1.5666 | 1.03E-09 | 1.17E-08 | -- | dihydrodipicolinate synthase family protein |
| ZMO_RS08340 | 774.6824211 | 344.439667 | 1.1694 | 0.01102 | 0.024983 | -- | PLP-dependent aminotransferase family protein |
| ZMO_RS08345 | 155.3444995 | 829.1331301 | -2.4161 | 7.36E-20 | 4.23E-18 | -- | GMP synthase |
| ZMO_RS08355 | 896.7121971 | 189.5555 | 2.242 | 2.97E-05 | 0.00013238 | -- | LysR family transcriptional regulator |
| ZMO_RS08395 | 2493.971228 | 1103.803354 | 1.176 | 2.27E-07 | 1.59E-06 | -- | VIT family protein |
| ZMO_RS08400 | 2629.934239 | 798.6560172 | 1.7194 | 1.02E-08 | 9.58E-08 | -- | nitronate monooxygenase |
| ZMO_RS08460 | 1619.599045 | 880.78132 | 0.87878 | 0.00501 | 0.012571 | -- | biotin--[acetyl-CoA-carboxylase] ligase |
| ZMO_RS08465 | 999.310463 | 670.4914785 | 0.57571 | 0.01958 | 0.041216 | -- | ribonuclease I |
| ZMO_RS08470 | 3335.882354 | 2000.708795 | 0.73756 | 0.02142 | 0.044277 | -- | nicotinate-nucleotide diphosphorylase (carboxylating) |
| ZMO_RS08475 | 5477.687198 | 2893.956537 | 0.92052 | 0.00038 | 0.001283 | -- | quinolinate synthase NadA |
| ZMO_RS08480 | 3154.454849 | 855.6350097 | 1.8823 | 1.99E-06 | 1.17E-05 | -- | DUF4230 domain-containing protein |
| ZMO_RS08485 | 4333.124131 | 961.3278673 | 2.1723 | 7.47E-21 | 4.57E-19 | -- | monothiol glutaredoxin%2C Grx4 family |
| ZMO_RS08490 | 334.7841158 | 104.794158 | 1.6757 | 6.20E-08 | 4.77E-07 | -- | BolA family transcriptional regulator |
| ZMO_RS08495 | 3012.406602 | 952.2114764 | 1.6616 | 3.59E-13 | 7.33E-12 | -- | DUF1476 domain-containing protein |
| ZMO_RS08505 | 1822.972251 | 1092.311677 | 0.73891 | 0.00173 | 0.0049825 | -- | glutamine cyclotransferase |
| ZMO_RS08510 | 4018.644338 | 7442.46693 | -0.88907 | 0.00011 | 0.0004248 | -- | valine--tRNA ligase |
| ZMO_RS08520 | 975.57873 | 1432.451615 | -0.55416 | 0.01673 | 0.035839 | -- | hypothetical protein |
| ZMO_RS08530 | 409.5923334 | 715.7461789 | -0.80526 | 0.00068 | 0.0021762 | -- | ammonia monooxygenase |
| ZMO_RS08535 | 774.0032959 | 454.3565205 | 0.76851 | 0.00238 | 0.0066324 | -- | glyoxylate/hydroxypyruvate reductase A |
| ZMO_RS08540 | 1263.689923 | 608.7641873 | 1.0537 | 7.87E-06 | 3.97E-05 | -- | hypothetical protein |
| ZMO_RS08545 | 13570.35126 | 1804.621633 | 2.9107 | 6.38E-09 | 6.20E-08 | -- | alkene reductase |
| ZMO_RS08565 | 614.5118793 | 1095.95311 | -0.83467 | 0.00058 | 0.0018796 | -- | SAM-dependent methyltransferase |
| ZMO_RS08570 | 1245.716521 | 2087.812254 | -0.74502 | 0.00178 | 0.0050922 | -- | insulinase family protein |
| ZMO_RS08590 | 1288.267089 | 2563.013067 | -0.99241 | 2.70E-05 | 0.00012031 | -- | adenosine kinase |
| ZMO_RS08595 | 2617.556898 | 4066.314712 | -0.6355 | 0.00466 | 0.011769 | -- | protein translocase subunit SecF |
| ZMO_RS08600 | 3593.715209 | 6211.094105 | -0.78937 | 0.00045 | 0.001514 | -- | protein translocase subunit SecD |
| ZMO_RS08605 | 916.0954789 | 2257.642709 | -1.3012 | 2.67E-08 | 2.23E-07 | -- | preprotein translocase subunit YajC |
| ZMO_RS08615 | 2728.555456 | 4534.65621 | -0.73286 | 0.0012 | 0.0036049 | -- | ketoacyl-ACP synthase III |
| ZMO_RS08620 | 6921.581294 | 15431.31039 | -1.1567 | 1.62E-07 | 1.15E-06 | -- | phosphate acyltransferase |
| ZMO_RS08625 | 1383.259099 | 2168.835845 | -0.64885 | 0.00444 | 0.011295 | -- | hypothetical protein |
| ZMO_RS08640 | 1452.206759 | 2164.276144 | -0.57564 | 0.01779 | 0.037794 | -- | tRNA (adenosine(37)-N6)-threonylcarbamoyltransferase complex transferase subunit TsaD |
| ZMO_RS08650 | 8194.472467 | 19309.95469 | -1.2366 | 1.98E-08 | 1.68E-07 | -- | translation initiation factor IF-3 |
| ZMO_RS08655 | 10797.14399 | 7059.328264 | 0.61305 | 0.00402 | 0.010398 | -- | DNA mismatch repair protein MutS |
| ZMO_RS08660 | 4742.876956 | 3342.802446 | 0.5047 | 0.02237 | 0.046041 | -- | outer membrane protein assembly factor BamE |
| ZMO_RS08665 | 2782.950946 | 1427.127239 | 0.9635 | 2.28E-05 | 0.00010341 | -- | DUF177 domain-containing protein |
| ZMO_RS08670 | 10103.99334 | 21554.95457 | -1.0931 | 1.22E-06 | 7.55E-06 | -- | 50S ribosomal protein L25 |
| ZMO_RS08675 | 1234.742138 | 2158.46099 | -0.80579 | 0.00051 | 0.0016745 | -- | peptidyl-tRNA hydrolase |
| ZMO_RS08710 | 1726.933721 | 683.3342198 | 1.3375 | 1.85E-05 | 8.61E-05 | -- | adenosylmethionine--8-amino-7-oxononanoate transaminase |
| ZMO_RS08720 | 3497.936272 | 964.1789779 | 1.8591 | 3.43E-05 | 0.00015146 | -- | hypothetical protein |
| ZMO_RS08725 | 1624.691338 | 821.1532611 | 0.98444 | 3.72E-05 | 0.00016114 | -- | primosomal protein N' |
| ZMO_RS08730 | 2543.838002 | 5308.295494 | -1.0612 | 3.34E-06 | 1.85E-05 | -- | capsular polysaccharide biosynthesis protein |
| ZMO_RS08735 | 1000.145742 | 1684.706892 | -0.75229 | 0.00165 | 0.0047685 | -- | LTA synthase family protein |
| ZMO_RS08755 | 21302.88959 | 1359.709354 | 3.9697 | 9.44E-10 | 1.07E-08 | -- | molecular chaperone GroES |
| ZMO_RS08760 | 126126.8851 | 12497.10194 | 3.3352 | 5.99E-06 | 3.10E-05 | -- | molecular chaperone GroEL |
| ZMO_RS08780 | 73.41485244 | 151.0075237 | -1.0405 | 0.0017 | 0.0049004 | -- | DNA repair protein RadC |
| ZMO_RS08785 | 437.4054605 | 1196.195026 | -1.4514 | 1.18E-09 | 1.31E-08 | -- | hypothetical protein |
| ZMO_RS08790 | 978.8369879 | 2327.566222 | -1.2497 | 2.30E-08 | 1.95E-07 | -- | hypothetical protein |
| ZMO_RS08855 | 4058.362406 | 468.604743 | 3.1145 | 0.00018 | 0.00066383 | -- | PLP-dependent aminotransferase family protein |
| ZMO_RS08860 | 583.7403182 | 46.24855621 | 3.6578 | 5.44E-10 | 6.42E-09 | -- | PhzF family phenazine biosynthesis protein |
| ZMO_RS08865 | 247.2970228 | 38.35303343 | 2.6888 | 0.00075 | 0.002369 | -- | KR domain-containing protein |
| ZMO_RS08870 | 72.42916376 | 15.39366695 | 2.2342 | 0.0056 | 0.013892 | -- | RidA family protein |
| ZMO_RS08875 | 117.5959946 | 29.92958929 | 1.9742 | 0.01664 | 0.035683 | -- | YbaK/EbsC family protein |
| ZMO_RS08880 | 167.7838799 | 37.52175553 | 2.1608 | 1.22E-05 | 5.88E-05 | -- | flavodoxin family protein |
| ZMO_RS08885 | 223.4288771 | 92.0610443 | 1.2792 | 0.00274 | 0.0075265 | -- | pyridoxal phosphate-dependent aminotransferase |
| ZMO_RS08890 | 80.02351401 | 38.01037902 | 1.074 | 0.01881 | 0.039788 | -- | PaaI family thioesterase |
| ZMO_RS08950 | 1423.616736 | 898.4052226 | 0.66412 | 0.00347 | 0.0091602 | -- | hypothetical protein |
| ZMO_RS08960 | 1742.839455 | 1132.0547 | 0.6225 | 0.00704 | 0.017103 | -- | sensor histidine kinase |
| ZMO_RS08985 | 403.5165037 | 599.8382538 | -0.57195 | 0.01963 | 0.041226 | -- | class A beta-lactamase |
| ZMO_RS08995 | 1447.881851 | 717.0570752 | 1.0138 | 0.00359 | 0.0093776 | -- | 3-methyl-2-oxobutanoate hydroxymethyltransferase |
| ZMO_RS09000 | 2154.53151 | 1076.19122 | 1.0014 | 0.00057 | 0.0018633 | -- | pantoate--beta-alanine ligase |
| ZMO_RS09010 | 268.7730602 | 146.328355 | 0.87718 | 0.00284 | 0.0077528 | -- | ArgP/LysG family DNA-binding transcriptional regulator |
| ZMO_RS09020 | 365.3937236 | 847.4280914 | -1.2136 | 1.87E-06 | 1.12E-05 | -- | hypothetical protein |
| ZMO_RS09025 | 1346.096142 | 829.0406685 | 0.69927 | 0.00337 | 0.0089394 | -- | MFS transporter |
| ZMO_RS09040 | 224.0424246 | 538.9865497 | -1.2665 | 3.12E-06 | 1.74E-05 | -- | ribosomal RNA small subunit methyltransferase G |
| ZMO_RS09045 | 1000.15861 | 2226.208187 | -1.1544 | 5.98E-07 | 3.88E-06 | -- | tRNA uridine 5-carboxymethylaminomethyl modification protein |
| ZMO_RS09050 | 1827.406321 | 272.5937806 | 2.745 | 9.20E-24 | 6.50E-22 | -- | SMP-30/gluconolactonase/LRE family protein |
| ZMO_RS09055 | 2333.632345 | 605.0837862 | 1.9474 | 1.14E-08 | 1.06E-07 | -- | aldo/keto reductase |
| ZMO_RS09070 | 1464.88963 | 695.7467598 | 1.0742 | 4.20E-06 | 2.27E-05 | -- | methylated-DNA--[protein]-cysteine S-methyltransferase |
| ZMO_RS09075 | 1861.421813 | 431.9846805 | 2.1074 | 1.70E-18 | 7.82E-17 | -- | dienelactone hydrolase family protein |
| ZMO_RS09080 | 3212.550455 | 897.255719 | 1.8401 | 1.70E-15 | 5.11E-14 | -- | quinone oxidoreductase |
| ZMO_RS09085 | 4107.029876 | 14336.81135 | -1.8036 | 7.82E-16 | 2.48E-14 | -- | transcription termination factor Rho |
| ZMO_RS09090 | 925.9898141 | 1347.220884 | -0.54092 | 0.02422 | 0.049253 | -- | CopD family protein |
| ZMO_RS09110 | 152.0560758 | 66.37851526 | 1.1958 | 0.00488 | 0.012297 | -- | hypothetical protein |
| ZMO_RS09120 | 808528.1248 | 115902.1882 | 2.8024 | 0.00626 | 0.015358 | rnpB | RNase P RNA component class A |
| ZMO_RS09130 | 592.0636482 | 187.296467 | 1.6604 | 2.65E-06 | 1.52E-05 | -- | redox-sensitive transcriptional activator SoxR |
| ZMO_RS09140 | 22239.75882 | 3374.970678 | 2.7202 | 2.92E-32 | 3.58E-30 | ssrA | transfer-messenger RNA |
| ZMO_RS09155 | 1537.032517 | 1033.015823 | 0.57329 | 0.01966 | 0.041256 | -- | hypothetical protein |
| ZMO_RS09165 | 561.7772966 | 358.0101045 | 0.65 | 0.01072 | 0.02445 | -- | IS5/IS1182 family transposase |
| ZMO_RS09170 | 404.0289998 | 107.8597789 | 1.9053 | 6.29E-11 | 8.44E-10 | -- | DUF465 domain-containing protein |
| ZMO_RS09175 | 226.6413026 | 131.9577395 | 0.78033 | 0.01011 | 0.023307 | -- | entericidin%2C EcnA/B family |
| ZMO_RS09185 | 3045.880735 | 10696.03899 | -1.8121 | 4.89E-16 | 1.60E-14 | -- | 50S ribosomal protein L35 |
| ZMO_RS09190 | 61.37019535 | 106.6548119 | -0.79734 | 0.02349 | 0.048082 | -- | 50S ribosomal protein L34 |
| ZMO_RS09195 | 38.15948577 | 103.2945098 | -1.4366 | 0.00031 | 0.0010666 | -- | membrane protein insertion efficiency factor YidD |
| ZMO_RS09205 | 1624.213014 | 4035.070714 | -1.3129 | 3.60E-09 | 3.70E-08 | -- | 50S ribosomal protein L32 |
| ZMO_RS09210 | 92.90999757 | 200.0487364 | -1.1064 | 0.00033 | 0.0011449 | -- | hypothetical protein |
| sRNA00017 | 1393.369486 | 7109.653603 | -2.3512 | 1.85E-24 | 1.36E-22 | -//- |  |
| sRNA00019 | 1604.099133 | 260.7437267 | 2.6211 | 0.01492 | 0.032692 | -//- |  |
| sRNA00026 | 1221.33509 | 5208.617102 | -2.0924 | 1.14E-18 | 5.51E-17 | -//- |  |
| sRNA00048 | 205.1610135 | 1514.589714 | -2.8841 | 2.53E-29 | 2.58E-27 | -//- |  |
| sRNA00059 | 38.06349251 | 7.988936507 | 2.2523 | 0.00232 | 0.0064671 | -//- |  |
| sRNA00061 | 29.54291156 | 150.9074074 | -2.3528 | 2.08E-09 | 2.26E-08 | -//- |  |

**Table S9** Differentially Expressed proteins of ZM4 strain in rich media and media with acetic + furfural treatments

| Protein | Description | Gene | ZM4_AF_1 | ZM4_AF_2 | ZM4_AF_3 | ZM4_RM_1 | ZM4_RM_2 | ZM4_RM_3 | ZM4_AF.vs.ZM4_RM FC | ZM4_AF.vs.ZM4_RM Pvalue | ZM4_AF.vs.ZM4_RM log2FC | ZM4_AF.vs.ZM4_RM UP.DOWN |
| --- | --- | --- | --- | --- | --- | --- | --- | --- | --- | --- | --- | --- |
| WP_011241206.1 | ATP-dependent chaperone ClpB | ZMO_RS06375*clpB | 1.2E+10 | 9.2E+09 | 1E+10 | 4.5E+09 | 6.3E+09 | 7.2E+09 | 1.77831 | 0.0204 | 0.83051 | up |
| WP_011240321.1 | ATP-dependent Clp protease ATP-binding subunit ClpA | ZMO_RS01740*clpA | 2E+09 | 1.6E+09 | 1.6E+09 | 9.6E+08 | 1E+09 | 1.2E+09 | 1.62878 | 0.01155 | 0.70379 | up |
| WP_011240872.1 | ribonucleoside triphosphate reductase | ZMO_RS04595*- | 1.1E+09 | 9.4E+08 | 9.2E+08 | 1.8E+09 | 2E+09 | 2E+09 | 0.5146 | 0.00038 | -0.95847 | down |
| WP_011240131.1 | transketolase | ZMO_RS00750*tkt | 1.1E+09 | 1.2E+09 | 1.1E+09 | 1.8E+09 | 2E+09 | 2E+09 | 0.58152 | 0.00112 | -0.78211 | down |
| WP_011241246.1 | hypothetical protein | ZMO_RS06580*- | 9.6E+08 | 9.2E+08 | 8.8E+08 | 1.5E+09 | 1.5E+09 | 1.6E+09 | 0.5966 | 0.00165 | -0.74516 | down |
| WP_011240174.1 | DNA polymerase I | ZMO_RS00975*polA | 4.7E+08 | 5E+08 | 4.7E+08 | 7.4E+08 | 6.3E+08 | 8.2E+08 | 0.6583 | 0.04334 | -0.60319 | down |
| WP_011240946.1 | NAD(P)-dependent oxidoreductase | ZMO_RS04990*- | 7.4E+08 | 7.3E+08 | 7.8E+08 | 1.1E+09 | 1.2E+09 | 1.2E+09 | 0.63839 | 0.00621 | -0.6475 | down |
| WP_011241050.1 | 1-deoxy-D-xylulose-5-phosphate synthase | ZMO_RS05550*- | 867918 | 2395577 | 2404251 | 7656805 | 4553611 | 7045187 | 0.29434 | 0.02351 | -1.76443 | down |
| WP_011240712.1 | SPOR domain-containing protein | ZMO_RS03770*- | 6.8E+08 | 4.7E+08 | 4.4E+08 | 7.4E+08 | 7.7E+08 | 9.8E+08 | 0.63733 | 0.04666 | -0.6499 | down |
| WP_011240404.1 | PBP1A family penicillin-binding protein | ZMO_RS02165*- | 4.9E+08 | 4.4E+08 | 4.7E+08 | 7.1E+08 | 7.1E+08 | 8.2E+08 | 0.62341 | 0.01087 | -0.68175 | down |
| WP_011241052.1 | alcohol dehydrogenase AdhP | ZMO_RS05560*adhP | 1.1E+10 | 9.8E+09 | 1E+10 | 5.1E+09 | 5.7E+09 | 7.1E+09 | 1.75538 | 0.0055 | 0.81179 | up |
| WP_011240838.1 | serine--tRNA ligase | ZMO_RS04420*serS | 7.5E+08 | 7.5E+08 | 6.7E+08 | 1.3E+09 | 1.1E+09 | 1.2E+09 | 0.60805 | 0.00921 | -0.71773 | down |
| WP_012817395.1 | phosphoribosylamine--glycine ligase | ZMO_RS01290*purD | 1.1E+09 | 1.1E+09 | 1.1E+09 | 7.1E+08 | 6.4E+08 | 5.7E+08 | 1.74607 | 0.00442 | 0.80411 | up |
| WP_011240715.1 | carbohydrate porin | ZMO_RS03785*- | 1.2E+09 | 1.3E+09 | 1.4E+09 | 5.8E+08 | 8.8E+08 | 8.6E+08 | 1.69914 | 0.01579 | 0.76481 | up |
| WP_011240740.1 | adenosyl-hopene transferase HpnH | ZMO_RS03910*hpnH | 1.9E+09 | 1.9E+09 | 1.9E+09 | 2.8E+09 | 3.4E+09 | 3.2E+09 | 0.6042 | 0.01468 | -0.72689 | down |
| WP_011241321.1 | amidophosphoribosyltransferase | ZMO_RS06965*- | 1.1E+09 | 1.1E+09 | 1.3E+09 | 2.6E+09 | 2.1E+09 | 1.9E+09 | 0.52294 | 0.02764 | -0.93529 | down |
| WP_011241495.1 | NAD-dependent succinate-semialdehyde dehydrogenase | ZMO_RS07890*- | 1.6E+09 | 1.5E+09 | 1.7E+09 | 9.3E+08 | 9.1E+08 | 9.1E+08 | 1.75248 | 0.00141 | 0.8094 | up |
| WP_026059405.1 | hopanoid biosynthesis associated radical SAM protein HpnJ | ZMO_RS04355*hpnJ | 2.3E+09 | 2.3E+09 | 2.5E+09 | 3.6E+09 | 3.8E+09 | 3.8E+09 | 0.63401 | 9.47E-05 | -0.65743 | down |
| WP_011240166.1 | helicase | ZMO_RS00935*- | 5.1E+08 | 4.1E+08 | 4.5E+08 | 2.6E+08 | 2.6E+08 | 3.2E+08 | 1.63108 | 0.0095 | 0.70582 | up |
| WP_011241080.1 | threonine ammonia-lyase | ZMO_RS05705*- | 9.8E+08 | 8.5E+08 | 7.5E+08 | 1.4E+09 | 1.4E+09 | 1.5E+09 | 0.60206 | 0.00388 | -0.73202 | down |
| WP_011240829.1 | aldo/keto reductase | ZMO_RS04375*- | 1.2E+09 | 1.1E+09 | 1.1E+09 | 5.4E+08 | 6.3E+08 | 6.9E+08 | 1.77908 | 0.00157 | 0.83113 | up |
| WP_011240557.1 | hypothetical protein | ZMO_RS02950*- | 8.3E+07 | 8.5E+07 | 6.9E+07 | 1.6E+08 | 1.8E+08 | 2.2E+08 | 0.42737 | 0.01607 | -1.22646 | down |
| WP_011240512.1 | flagellar hook-basal body complex protein | ZMO_RS02720*- | 1.6E+09 | 1.9E+09 | 1.8E+09 | 8.9E+08 | 8.9E+08 | 1.1E+09 | 1.84833 | 0.00257 | 0.88622 | up |
| WP_011240127.1 | phosphomethylpyrimidine synthase ThiC | ZMO_RS00730*thiC | 4.6E+08 | 4.5E+08 | 5.4E+08 | 2.3E+08 | 2.6E+08 | 3.1E+08 | 1.82346 | 0.00433 | 0.86668 | up |
| WP_011241376.1 | membrane protein insertase YidC | ZMO_RS07290*yidC | 6.5E+08 | 6.4E+08 | 6.7E+08 | 9.6E+08 | 9.4E+08 | 1.2E+09 | 0.62889 | 0.04332 | -0.66911 | down |
| WP_011240056.1 | chemotaxis protein CheA | ZMO_RS00345*- | 3.9E+08 | 3.3E+08 | 3.7E+08 | 6E+08 | 5.5E+08 | 6.4E+08 | 0.60719 | 0.00397 | -0.71979 | down |
| WP_011240963.1 | c-type cytochrome | ZMO_RS05080*- | 4.3E+08 | 4E+08 | 3.4E+08 | 6.3E+08 | 6.1E+08 | 8.5E+08 | 0.55832 | 0.04775 | -0.84085 | down |
| WP_011241477.1 | peroxiredoxin | ZMO_RS07800*- | 5.6E+09 | 4.9E+09 | 4.9E+09 | 2.9E+09 | 3.2E+09 | 3.9E+09 | 1.52114 | 0.01313 | 0.60515 | up |
| WP_011241169.1 | toxic anion resistance protein | ZMO_RS06185*- | 8.2E+08 | 7.6E+08 | 7.9E+08 | 4.7E+08 | 4.4E+08 | 6.2E+08 | 1.54994 | 0.02822 | 0.63222 | up |
| WP_011241530.1 | 3-phosphoshikimate 1-carboxyvinyltransferase | ZMO_RS08080*aroA | 1.9E+09 | 1.7E+09 | 1.8E+09 | 1.2E+09 | 1.1E+09 | 1.1E+09 | 1.59019 | 0.00127 | 0.6692 | up |
| WP_011241200.1 | DEAD/DEAH box helicase | ZMO_RS06345*- | 7.2E+08 | 8.3E+08 | 8E+08 | 2E+09 | 2.4E+09 | 1.9E+09 | 0.37301 | 0.01023 | -1.42273 | down |
| WP_011241478.1 | LysR family transcriptional regulator | ZMO_RS07805*- | 9.9E+08 | 1E+09 | 9.3E+08 | 1.6E+09 | 1.8E+09 | 1.9E+09 | 0.55546 | 0.01301 | -0.84824 | down |
| WP_011241507.1 | UTP--glucose-1-phosphate uridylyltransferase GalU | ZMO_RS07950*galU | 5.5E+08 | 5.8E+08 | 5.5E+08 | 8.4E+08 | 8.5E+08 | 8.8E+08 | 0.6527 | 4.95E-05 | -0.6155 | down |
| WP_011240073.1 | serine hydrolase | ZMO_RS00430*- | 4.4E+08 | 3.4E+08 | 3.8E+08 | 5.6E+08 | 6.1E+08 | 6.3E+08 | 0.6399 | 0.00495 | -0.64408 | down |
| WP_011241155.1 | 30S ribosomal protein S4 | ZMO_RS06110*rpsD | 1E+10 | 1E+10 | 1E+10 | 1.6E+10 | 1.7E+10 | 1.7E+10 | 0.62004 | 0.00555 | -0.68957 | down |
| WP_011240777.1 | catalase | ZMO_RS04105*- | 1E+09 | 9.7E+08 | 9.8E+08 | 4.8E+08 | 5.9E+08 | 6.6E+08 | 1.71718 | 0.0102 | 0.78004 | up |
| WP_011240547.1 | glycosyltransferase | ZMO_RS02900*- | 2E+08 | 1.7E+08 | 1.4E+08 | 2.8E+08 | 2.4E+08 | 2.7E+08 | 0.64145 | 0.01162 | -0.64059 | down |
| WP_011240992.1 | CocE/NonD family hydrolase | ZMO_RS05230*- | 1.5E+08 | 1.6E+08 | 1.8E+08 | 3.2E+08 | 2.7E+08 | 3.4E+08 | 0.52384 | 0.0117 | -0.93281 | down |
| WP_011241351.1 | M61 family metallopeptidase | ZMO_RS07155*- | 1.2E+08 | 1.4E+08 | 1.3E+08 | 2.5E+08 | 1.9E+08 | 2.6E+08 | 0.56376 | 0.0327 | -0.82686 | down |
| WP_011240767.1 | LPS biosynthesis protein | ZMO_RS04055*- | 5E+08 | 4.8E+08 | 4.8E+08 | 8.2E+08 | 8.7E+08 | 8.6E+08 | 0.57586 | 6.90E-05 | -0.79621 | down |
| WP_011240677.1 | N-acetyl-gamma-glutamyl-phosphate reductase | ZMO_RS03585*argC | 1.5E+08 | 1.3E+08 | 1.2E+08 | 2.7E+08 | 2.1E+08 | 2.4E+08 | 0.56234 | 0.0199 | -0.83049 | down |
| WP_011240554.1 | molecular chaperone DnaJ | ZMO_RS02935*dnaJ | 3.9E+08 | 3.4E+08 | 3.5E+08 | 5.8E+08 | 5.3E+08 | 5.9E+08 | 0.63427 | 0.00121 | -0.65682 | down |
| WP_011240605.1 | lytic transglycosylase domain-containing protein | ZMO_RS03205*- | 1.6E+08 | 1.4E+08 | 1.6E+08 | 2.2E+08 | 2.7E+08 | 2.6E+08 | 0.61377 | 0.01668 | -0.70423 | down |
| WP_011241211.1 | HlyD family secretion protein | ZMO_RS06400*- | 4.1E+08 | 4.9E+08 | 5.1E+08 | 8.5E+08 | 8.1E+08 | 9.7E+08 | 0.53498 | 0.00363 | -0.90244 | down |
| WP_011240733.1 | NAD-dependent epimerase/dehydratase family protein | ZMO_RS03875*- | 2.3E+08 | 2E+08 | 2.3E+08 | 4.2E+08 | 3.9E+08 | 3.9E+08 | 0.55907 | 0.00024 | -0.83891 | down |
| WP_011240216.1 | LPS export ABC transporter ATP-binding protein | ZMO_RS01200*lptB | 1.9E+08 | 1.7E+08 | 1.7E+08 | 3.1E+08 | 2.4E+08 | 3E+08 | 0.62764 | 0.03615 | -0.67199 | down |
| WP_011241681.1 | glycosyltransferase family 1 protein | ZMO_RS08935*- | 1.1E+08 | 1E+08 | 1.3E+08 | 2.1E+08 | 2.9E+08 | 2.7E+08 | 0.43142 | 0.01512 | -1.21282 | down |
| WP_011241454.1 | orotate phosphoribosyltransferase | ZMO_RS07680*- | 1.9E+09 | 1.8E+09 | 1.5E+09 | 1E+09 | 9.1E+08 | 7.1E+08 | 1.96386 | 0.00495 | 0.97369 | up |
| WP_011240340.1 | SufD family Fe-S cluster assembly protein | ZMO_RS01835*- | 1.8E+08 | 1.8E+08 | 1.6E+08 | 2.6E+08 | 2.7E+08 | 2.8E+08 | 0.64318 | 0.00191 | -0.63671 | down |
| WP_011240960.1 | carbonic anhydrase | ZMO_RS05065*- | 8E+08 | 7.6E+08 | 8.5E+08 | 4.7E+08 | 4.1E+08 | 4.9E+08 | 1.75646 | 0.00064 | 0.81267 | up |
| WP_011240585.1 | YfdX family protein | ZMO_RS03095*- | 2E+09 | 1.3E+09 | 1.7E+09 | 5E+08 | 4.7E+08 | 7.1E+08 | 2.99262 | 0.02233 | 1.58141 | up |
| WP_011241363.1 | hypothetical protein | ZMO_RS07225*- | 3.1E+09 | 3.4E+09 | 3E+09 | 1.6E+09 | 1.7E+09 | 1.6E+09 | 1.94749 | 0.00159 | 0.96162 | up |
| WP_011240355.1 | ribonucleotide-diphosphate reductase subunit beta | ZMO_RS01910*- | 3.5E+08 | 3E+08 | 2.6E+08 | 6.3E+08 | 5.5E+08 | 5.9E+08 | 0.51575 | 0.00094 | -0.95524 | down |
| WP_011241274.1 | 1-(5-phosphoribosyl)-5 | ZMO_RS06730*hisA | 1.4E+09 | 1.4E+09 | 1.3E+09 | 7E+08 | 5.7E+08 | 7.7E+08 | 1.99139 | 0.00119 | 0.99378 | up |
| WP_011240654.1 | efflux RND transporter periplasmic adaptor subunit | ZMO_RS03470*- | 2.1E+08 | 1.6E+08 | 1.5E+08 | 2.6E+08 | 2.3E+08 | 2.8E+08 | 0.66572 | 0.02212 | -0.58701 | down |
| WP_011241213.1 | FUSC family protein | ZMO_RS06410*- | 3.2E+07 | 3.3E+07 | 3.8E+07 | 1.3E+08 | 1.3E+08 | 1.8E+08 | 0.23388 | 0.02067 | -2.09616 | down |
| WP_017466469.1 | hypothetical protein | ZMO_RS08625*- | 1.7E+08 | 1.5E+08 | 1.3E+08 | 2.2E+08 | 2.4E+08 | 2.8E+08 | 0.58943 | 0.01136 | -0.7626 | down |
| WP_011240432.1 | 30S ribosomal protein S17 | ZMO_RS02305*rpsQ | 5.6E+09 | 5.6E+09 | 6E+09 | 1E+10 | 9.3E+09 | 9.6E+09 | 0.59039 | 0.00107 | -0.76025 | down |
| WP_011240959.1 | lipoyl synthase | ZMO_RS05060*lipA | 2.9E+08 | 3.2E+08 | 4.2E+08 | 6.3E+08 | 6.5E+08 | 5E+08 | 0.58035 | 0.01481 | -0.785 | down |
| WP_017466360.1 | adenosine deaminase | ZMO_RS02905*- | 1.7E+08 | 1.8E+08 | 2.5E+08 | 3.9E+08 | 3.7E+08 | 3.1E+08 | 0.56758 | 0.0111 | -0.8171 | down |
| WP_011241612.1 | alkene reductase | ZMO_RS08545*- | 2.1E+09 | 1.4E+09 | 1.7E+09 | 5E+08 | 4.1E+08 | 4.7E+08 | 3.80196 | 0.02664 | 1.92674 | up |
| WP_011240093.1 | S1/P1 Nuclease | ZMO_RS00535*- | 3.1E+08 | 2.8E+08 | 2.4E+08 | 5.1E+08 | 5.6E+08 | 5.5E+08 | 0.51079 | 0.00052 | -0.96919 | down |
| WP_011241498.1 | gluconokinase | ZMO_RS07905*- | 1.2E+09 | 1.2E+09 | 1.1E+09 | 7E+08 | 7E+08 | 6.6E+08 | 1.68511 | 0.00347 | 0.75284 | up |
| WP_011240242.1 | SDR family oxidoreductase | ZMO_RS01330*- | 7.6E+08 | 6.1E+08 | 6.9E+08 | 3.7E+08 | 3.5E+08 | 5.8E+08 | 1.58985 | 0.04958 | 0.66889 | up |
| WP_011240736.1 | presqualene diphosphate synthase HpnD | ZMO_RS03890*hpnD | 2.8E+08 | 2.6E+08 | 2.6E+08 | 4.6E+08 | 4.3E+08 | 4.7E+08 | 0.59072 | 0.00142 | -0.75947 | down |
| WP_011240894.1 | bifunctional diguanylate cyclase/phosphodiesterase | ZMO_RS04720*- | 4.7E+07 | 4.5E+07 | 5.8E+07 | 9E+07 | 1.2E+08 | 9.7E+07 | 0.49723 | 0.00891 | -1.00801 | down |
| WP_011240586.1 | hypothetical protein | ZMO_RS03105*- | 9.2E+07 | 9.5E+07 | 1.2E+08 | 6.3E+07 | 4.3E+07 | 6.2E+07 | 1.84972 | 0.0185 | 0.88731 | up |
| WP_011240969.1 | thioredoxin-disulfide reductase | ZMO_RS05110*trxB | 2.4E+08 | 2.2E+08 | 1.9E+08 | 4.7E+08 | 3.6E+08 | 4.2E+08 | 0.51429 | 0.01123 | -0.95935 | down |
| WP_011240503.1 | OmpA family protein | ZMO_RS02675*- | 1.1E+08 | 9.8E+07 | 9E+07 | 1.5E+08 | 1.6E+08 | 1.7E+08 | 0.63055 | 0.0061 | -0.66532 | down |
| WP_011240529.1 | flagellar M-ring protein FliF | ZMO_RS02810*fliF | 8.3E+07 | 8.6E+07 | 8.7E+07 | 1.8E+08 | 2.4E+08 | 2.5E+08 | 0.38123 | 0.02134 | -1.39127 | down |
| WP_011240693.1 | 16S rRNA (cytosine(1402)-N(4))-methyltransferase RsmH | ZMO_RS03675*rsmH | 7.9E+07 | 8.3E+07 | 6.7E+07 | 1.2E+08 | 1.2E+08 | 1.2E+08 | 0.65638 | 0.0114 | -0.6074 | down |
| WP_011241468.1 | S-(hydroxymethyl)glutathione dehydrogenase/class III alcohol dehydrogenase | ZMO_RS07755*- | 2.4E+08 | 2E+08 | 1.9E+08 | 2.9E+08 | 3.6E+08 | 3.7E+08 | 0.61918 | 0.02134 | -0.69157 | down |
| WP_011241368.1 | transcription elongation factor GreA | ZMO_RS07250*greA | 2E+09 | 1.8E+09 | 1.7E+09 | 7.3E+08 | 7.8E+08 | 8.1E+08 | 2.38449 | 0.00546 | 1.25368 | up |
| WP_011241180.1 | hypothetical protein | ZMO_RS06240*- | 5.1E+07 | 1.3E+07 | 2.7E+07 | 6.7E+08 | 6.8E+08 | 6.4E+08 | 0.04577 | 2.14E-06 | -4.4495 | down |
| WP_011240284.1 | excinuclease ABC subunit UvrB | ZMO_RS01550*uvrB | 2.7E+08 | 2.4E+08 | 2.2E+08 | 1.3E+08 | 1.5E+08 | 1.9E+08 | 1.53404 | 0.02634 | 0.61733 | up |
| WP_011240898.1 | superoxide dismutase | ZMO_RS04740*- | 1.8E+09 | 1.5E+09 | 1.4E+09 | 4.5E+08 | 2.4E+08 | 2.8E+08 | 4.74869 | 0.00301 | 2.24753 | up |
| WP_011240748.1 | 50S ribosomal protein L13 | ZMO_RS03950*rplM | 2.4E+09 | 1.8E+09 | 2E+09 | 4.6E+09 | 5.9E+09 | 6.7E+09 | 0.36387 | 0.01831 | -1.4585 | down |
| WP_011240879.1 | calcium-binding protein | ZMO_RS04630*- | 1.2E+09 | 1.1E+09 | 1.2E+09 | 6.9E+08 | 6.8E+08 | 7.7E+08 | 1.61896 | 0.00161 | 0.69507 | up |
| WP_011241323.1 | RNA pyrophosphohydrolase | ZMO_RS06975*- | 4.5E+08 | 4.7E+08 | 4E+08 | 2.2E+08 | 1.8E+08 | 3.3E+08 | 1.81268 | 0.03185 | 0.85812 | up |
| WP_011240623.1 | CsbD family protein | ZMO_RS03310*- | 3.3E+09 | 3.2E+09 | 2.7E+09 | 9.3E+08 | 6.8E+08 | 1.5E+09 | 2.97039 | 0.00303 | 1.57065 | up |
| WP_011240023.1 | ribosome-associated translation inhibitor RaiA | ZMO_RS00175*raiA | 9.3E+08 | 8.6E+08 | 7.9E+08 | 2E+08 | 2.2E+08 | 3.7E+08 | 3.23496 | 0.00111 | 1.69375 | up |
| WP_011241494.1 | ferredoxin--NADP reductase | ZMO_RS07885*- | 6.3E+08 | 6.8E+08 | 6.7E+08 | 2.8E+08 | 2.3E+08 | 2.6E+08 | 2.58206 | 2.47E-05 | 1.36852 | up |
| WP_011240842.1 | heat-shock protein IbpA | ZMO_RS04430*- | 2.3E+09 | 1.6E+09 | 1.9E+09 | 9.5E+08 | 1.1E+09 | 1E+09 | 1.87718 | 0.04907 | 0.90857 | up |
| WP_011241192.1 | inositol monophosphatase | ZMO_RS06300*- | 6.3E+08 | 6.2E+08 | 5.1E+08 | 3.7E+08 | 2.9E+08 | 3E+08 | 1.83276 | 0.00551 | 0.87402 | up |
| WP_011241543.1 | electron transport complex subunit RsxG | ZMO_RS08135*rsxG | 7.5E+07 | 5.7E+07 | 1.1E+08 | 1.7E+08 | 2.5E+08 | 2.5E+08 | 0.36095 | 0.01523 | -1.47012 | down |
| WP_011240439.1 | 50S ribosomal protein L18 | ZMO_RS02340*rplR | 2.6E+09 | 2.8E+09 | 3.4E+09 | 5.4E+09 | 5.5E+09 | 5.1E+09 | 0.54838 | 0.00371 | -0.86676 | down |
| WP_011240200.1 | response regulator transcription factor | ZMO_RS01115*- | 1.5E+09 | 1.4E+09 | 1.4E+09 | 5.5E+08 | 5.6E+08 | 7E+08 | 2.371 | 0.00075 | 1.24549 | up |
| WP_011240964.1 | phosphoserine phosphatase SerB | ZMO_RS05085*serB | 3.2E+09 | 2.9E+09 | 3E+09 | 2.1E+09 | 1.8E+09 | 2E+09 | 1.55754 | 0.00118 | 0.63927 | up |
| WP_011240504.1 | flagellar motor stator protein MotA | ZMO_RS02680*motA | 7.5E+07 | 7.8E+07 | 6.8E+07 | 1.5E+08 | 1.7E+08 | 1.7E+08 | 0.4592 | 0.00076 | -1.1228 | down |
| WP_011241196.1 | MucR family transcriptional regulator | ZMO_RS06320*- | 9.5E+08 | 7.4E+08 | 8.2E+08 | 2.8E+09 | 2.6E+09 | 2.7E+09 | 0.31152 | 2.35E-05 | -1.68258 | down |
| WP_011241086.1 | c-type cytochrome | ZMO_RS05740*- | 3.1E+08 | 3.1E+08 | 9.3E+08 | 1.4E+09 | 2.1E+09 | 1.4E+09 | 0.3168 | 0.02172 | -1.65834 | down |
| WP_011241006.1 | tetratricopeptide repeat protein | ZMO_RS05300*- | 4.5E+07 | 2.8E+07 | 4.1E+07 | 8.1E+07 | 6.4E+07 | 9E+07 | 0.48458 | 0.01624 | -1.0452 | down |
| WP_011241009.1 | lytic transglycosylase domain-containing protein | ZMO_RS05315*- | 1.5E+07 | 1.5E+07 | 9088586 | 1.2E+08 | 1.1E+08 | 1.3E+08 | 0.10793 | 0.00021 | -3.21185 | down |
| WP_011240428.1 | 50S ribosomal protein L22 | ZMO_RS02285*rplV | 3.9E+09 | 4.2E+09 | 3.8E+09 | 7.3E+09 | 6.5E+09 | 7.5E+09 | 0.55562 | 0.00479 | -0.84784 | down |
| WP_011241132.1 | polyisoprenoid-binding protein | ZMO_RS05970*- | 4.2E+08 | 4.3E+08 | 5.2E+08 | 2.1E+08 | 2.1E+08 | 2.6E+08 | 2.00651 | 0.00613 | 1.00469 | up |
| WP_011241300.1 | KpsF/GutQ family sugar-phosphate isomerase | ZMO_RS06855*- | 1E+08 | 9.7E+07 | 1E+08 | 1.3E+08 | 1.5E+08 | 1.7E+08 | 0.65689 | 0.03938 | -0.60628 | down |
| WP_011241647.1 | capsular biosynthesis protein | ZMO_RS08730*- | 7.9E+07 | 4.7E+07 | 5.7E+07 | 1.4E+08 | 1.3E+08 | 1.7E+08 | 0.41147 | 0.00416 | -1.28113 | down |
| WP_011241005.1 | 4-(cytidine 5\-diphospho)-2-C-methyl-D-erythritol kinase | ZMO_RS05295*- | 3.5E+08 | 3.6E+08 | 3.7E+08 | 1.9E+08 | 1.5E+08 | 1.8E+08 | 2.06459 | 0.00088 | 1.04585 | up |
| WP_011241253.1 | 6-phosphogluconolactonase | ZMO_RS06615*pgl | 5.2E+09 | 5.2E+09 | 5.3E+09 | 2.4E+09 | 1.9E+09 | 2.8E+09 | 2.2112 | 0.00715 | 1.14483 | up |
| WP_011240354.1 | HAD family phosphatase | ZMO_RS01905*- | 3E+08 | 2.7E+08 | 2.8E+08 | 1.8E+08 | 1.7E+08 | 1.8E+08 | 1.59465 | 0.00121 | 0.67324 | up |
| WP_011241194.1 | bacterioferritin | ZMO_RS06310*bfr | 2.3E+09 | 2E+09 | 2.3E+09 | 1.1E+09 | 1.2E+09 | 1.6E+09 | 1.66569 | 0.00813 | 0.73612 | up |
| WP_011241118.1 | hypothetical protein | ZMO_RS05900*- | 6.3E+07 | 5.3E+07 | 5.9E+07 | 1E+08 | 1.2E+08 | 1.3E+08 | 0.50428 | 0.00726 | -0.98771 | down |
| WP_011241078.1 | succinylglutamate-semialdehyde dehydrogenase | ZMO_RS05695*astD | 2.1E+08 | 2.3E+08 | 2.4E+08 | 1.6E+08 | 1.3E+08 | 1.6E+08 | 1.51825 | 0.00254 | 0.60241 | up |
| WP_011240768.1 | glycosyltransferase family 4 protein | ZMO_RS04060*- | 9.2E+07 | 8.3E+07 | 1.1E+08 | 1.5E+08 | 1.3E+08 | 1.6E+08 | 0.66008 | 0.01845 | -0.59929 | down |
| WP_011240728.1 | YbaB/EbfC family nucleoid-associated protein | ZMO_RS03850*- | 1.4E+09 | 1.4E+09 | 1.3E+09 | 7E+08 | 7.5E+08 | 6.8E+08 | 1.88618 | 0.00018 | 0.91547 | up |
| WP_011240334.1 | 1-acyl-sn-glycerol-3-phosphate acyltransferase | ZMO_RS01805*- | 1.1E+08 | 9.6E+07 | 1.1E+08 | 2.2E+08 | 2.4E+08 | 2.5E+08 | 0.43754 | 0.00042 | -1.19251 | down |
| WP_011240279.1 | rod shape-determining protein MreC | ZMO_RS01525*mreC | 6.7E+07 | 5.8E+07 | 4.4E+07 | 8.8E+07 | 1.1E+08 | 1.1E+08 | 0.55738 | 0.00853 | -0.84326 | down |
| WP_011240427.1 | 30S ribosomal protein S19 | ZMO_RS02280*rpsS | 1.9E+09 | 1.9E+09 | 2.1E+09 | 4.3E+09 | 4.7E+09 | 4.5E+09 | 0.44026 | 6.42E-05 | -1.18357 | down |
| WP_011240409.1 | RNA pseudouridine synthase | ZMO_RS02190*- | 8.1E+07 | 6.9E+07 | 8E+07 | 1.7E+08 | 1.3E+08 | 1.6E+08 | 0.50333 | 0.01454 | -0.99043 | down |
| WP_011240204.1 | hypothetical protein | ZMO_RS01135*- | 6E+07 | 5.4E+07 | 6.1E+07 | 2.6E+07 | 3.7E+07 | 3.6E+07 | 1.7573 | 0.00766 | 0.81336 | up |
| WP_011241531.1 | (d)CMP kinase | ZMO_RS08085*- | 6.6E+08 | 6.1E+08 | 6.1E+08 | 3E+08 | 2.6E+08 | 2.9E+08 | 2.23335 | 6.17E-05 | 1.15921 | up |
| WP_011241038.1 | twin-arginine translocase TatA/TatE family subunit | ZMO_RS05485*- | 3.4E+08 | 3.3E+08 | 3.2E+08 | 1.4E+08 | 2.1E+08 | 2.2E+08 | 1.74248 | 0.029 | 0.80115 | up |
| WP_011241220.1 | deaminated glutathione amidase | ZMO_RS06445*- | 4.6E+07 | 4.4E+07 | 5E+07 | 6.9E+07 | 7.6E+07 | 8.5E+07 | 0.60707 | 0.0141 | -0.72007 | down |
| WP_011240980.1 | ribosome recycling factor | ZMO_RS05165*- | 2.4E+09 | 2.4E+09 | 2.4E+09 | 1.2E+09 | 1.1E+09 | 1.1E+09 | 2.09865 | 0.00016 | 1.06946 | up |
| WP_011240240.1 | glycosyltransferase family 4 protein | ZMO_RS01320*- | 7.5E+07 | 7.7E+07 | 3.4E+07 | 1.3E+08 | 1.4E+08 | 1.4E+08 | 0.46824 | 0.0291 | -1.09468 | down |
| WP_011240907.1 | signal recognition particle-docking protein FtsY | ZMO_RS04790*ftsY | 4.4E+08 | 3.9E+08 | 3.9E+08 | 2.5E+08 | 2.3E+08 | 2.6E+08 | 1.64697 | 0.00236 | 0.71982 | up |
| WP_011240357.1 | SGNH/GDSL hydrolase family protein | ZMO_RS01920*- | 2.5E+07 | 2.9E+07 | 1.5E+07 | 6.8E+07 | 8.5E+07 | 9.6E+07 | 0.27417 | 0.00681 | -1.86688 | down |
| WP_011240746.1 | globin-coupled sensor protein | ZMO_RS03940*- | 1.5E+08 | 1.6E+08 | 1.1E+08 | 6.9E+07 | 7.5E+07 | 1.1E+08 | 1.65241 | 0.04611 | 0.72457 | up |
| WP_011240639.1 | hydroxyacylglutathione hydrolase | ZMO_RS03395*gloB | 3.7E+08 | 3.7E+08 | 3.6E+08 | 1.9E+08 | 1.7E+08 | 2E+08 | 1.95122 | 0.0001 | 0.96438 | up |
| WP_011240854.1 | site-specific DNA-methyltransferase | ZMO_RS04495*- | 2.6E+07 | 2.6E+07 | 1.9E+07 | 4.3E+07 | 4.9E+07 | 6E+07 | 0.46583 | 0.01898 | -1.10211 | down |
| WP_011241120.1 | hypothetical protein | ZMO_RS05910*- | 1.3E+08 | 6.6E+07 | 1E+08 | 2.2E+08 | 2E+08 | 1.5E+08 | 0.53974 | 0.04029 | -0.88966 | down |
| WP_011240281.1 | penicillin-binding protein 2 | ZMO_RS01535*mrdA | 3.4E+07 | 2.8E+07 | 3E+07 | 4.6E+07 | 4.2E+07 | 5.3E+07 | 0.65495 | 0.01864 | -0.61053 | down |
| WP_011241000.1 | response regulator transcription factor | ZMO_RS05270*- | 1.5E+08 | 1.3E+08 | 1.3E+08 | 6.3E+07 | 8.5E+07 | 9.1E+07 | 1.71204 | 0.00746 | 0.77571 | up |
| WP_011240446.1 | 30S ribosomal protein S11 | ZMO_RS02375*rpsK | 2.2E+09 | 1.9E+09 | 2E+09 | 5.4E+09 | 6.1E+09 | 5.7E+09 | 0.36209 | 0.00062 | -1.46556 | down |
| WP_011241538.1 | HAD hydrolase-like protein | ZMO_RS08110*- | 2.7E+08 | 2.1E+08 | 2.5E+08 | 1.2E+08 | 1.4E+08 | 1.5E+08 | 1.78328 | 0.01059 | 0.83454 | up |
| WP_011241464.1 | hypothetical protein | ZMO_RS07735*- | 3.7E+08 | 3.6E+08 | 4E+08 | 2.1E+08 | 1.8E+08 | 2.4E+08 | 1.82387 | 0.00176 | 0.86701 | up |
| WP_011240729.1 | dCTP deaminase | ZMO_RS03855*- | 3.8E+08 | 3.7E+08 | 3.4E+08 | 6.2E+08 | 5.4E+08 | 5.2E+08 | 0.65087 | 0.01218 | -0.61956 | down |
| WP_011241457.1 | signal peptidase I | ZMO_RS07695*lepB | 1.8E+08 | 1.4E+08 | 1.3E+08 | 2.1E+08 | 2.7E+08 | 2.7E+08 | 0.58608 | 0.01522 | -0.77082 | down |
| WP_011240584.1 | organic hydroperoxide resistance protein | ZMO_RS03090*- | 3.2E+09 | 2.9E+09 | 3.1E+09 | 1.2E+09 | 1.3E+09 | 1.3E+09 | 2.36783 | 0.00069 | 1.24357 | up |
| WP_011241103.1 | hypothetical protein | ZMO_RS05825*- | 7.5E+07 | 8.1E+07 | 8.6E+07 | 1.2E+08 | 1.3E+08 | 1.5E+08 | 0.60139 | 0.02725 | -0.73364 | down |
| WP_011240521.1 | flagellar biosynthesis protein FlhA | ZMO_RS02770*flhA | 5.5E+07 | 4.8E+07 | 4.2E+07 | 8.4E+07 | 7.7E+07 | 8.5E+07 | 0.58636 | 0.00282 | -0.77014 | down |
| WP_017466353.1 | hypothetical protein | ZMO_RS02650*- | 3.4E+07 | 2.9E+07 | 3.9E+07 | 7.8E+07 | 7.3E+07 | 7.4E+07 | 0.45502 | 0.00131 | -1.13599 | down |
| WP_011241394.1 | ATP-dependent DNA helicase RecG | ZMO_RS07380*recG | 5E+07 | 4.9E+07 | 6.7E+07 | 3.2E+08 | 2.7E+08 | 1.9E+08 | 0.21061 | 0.02687 | -2.24734 | down |
| WP_011240238.1 | ferrochelatase | ZMO_RS01310*- | 3.3E+07 | 3.2E+07 | 2.7E+07 | 9.7E+07 | 1E+08 | 1.3E+08 | 0.27776 | 0.01481 | -1.8481 | down |
| WP_011240003.1 | heat-inducible transcriptional repressor HrcA | ZMO_RS00075*hrcA | 1.1E+08 | 9.1E+07 | 7.7E+07 | 4.9E+07 | 5.1E+07 | 5.1E+07 | 1.81163 | 0.04063 | 0.85729 | up |
| WP_011240823.1 | purine nucleoside permease | ZMO_RS04340*- | 3.4E+07 | 3.1E+07 | 3.5E+07 | 1.5E+08 | 1.1E+08 | 1E+08 | 0.28317 | 0.02447 | -1.82024 | down |
| WP_011240434.1 | 50S ribosomal protein L24 | ZMO_RS02315*rplX | 8.8E+09 | 4E+09 | 5.2E+09 | 1.1E+10 | 1.1E+10 | 1.4E+10 | 0.50967 | 0.03658 | -0.97238 | down |
| WP_011241422.1 | aldo/keto reductase | ZMO_RS07520*- | 5.8E+08 | 5E+08 | 5.7E+08 | 3.2E+08 | 3.5E+08 | 4E+08 | 1.54158 | 0.00509 | 0.62441 | up |
| WP_011241693.1 | 3-methyl-2-oxobutanoate hydroxymethyltransferase | ZMO_RS08995*panB | 7.9E+07 | 9.1E+07 | 6.3E+07 | 1.4E+08 | 1.3E+08 | 1.4E+08 | 0.57241 | 0.00762 | -0.80487 | down |
| WP_011240839.1 | RNA polymerase-binding protein DksA | ZMO_RS04425*dksA | 7.8E+08 | 8.1E+08 | 7.9E+08 | 5.6E+08 | 4.7E+08 | 5.3E+08 | 1.52548 | 0.00515 | 0.60927 | up |
| WP_011240622.1 | monooxygenase | ZMO_RS03305*- | 5.9E+08 | 5.9E+08 | 4.6E+08 | 3.4E+08 | 2E+08 | 4.1E+08 | 1.72298 | 0.04507 | 0.78491 | up |
| WP_011241385.1 | murein transglycosylase A | ZMO_RS07335*- | 2E+07 | 1E+07 | 1.1E+07 | 4.2E+07 | 4.8E+07 | 5.8E+07 | 0.27765 | 0.0049 | -1.84864 | down |
| WP_011240972.1 | 50S ribosomal protein L31 | ZMO_RS05125*rpmE | 4.2E+09 | 3.7E+09 | 4.6E+09 | 1E+10 | 8.9E+09 | 7E+09 | 0.47931 | 0.03369 | -1.06096 | down |
| WP_011241657.1 | hypothetical protein | ZMO_RS08785*- | 9.3E+07 | 9.4E+07 | 1.2E+08 | 1.6E+08 | 2.2E+08 | 2.1E+08 | 0.51159 | 0.0258 | -0.96695 | down |
| WP_011240756.1 | NERD domain-containing protein | ZMO_RS03990*- | 9188387 | 1.3E+07 | 7001982 | 4.4E+07 | 3.3E+07 | 3.4E+07 | 0.26427 | 0.00552 | -1.91992 | down |
| WP_011240257.1 | elongation factor P | ZMO_RS01410*efp | 1E+09 | 9.9E+08 | 9.6E+08 | 6.6E+08 | 7E+08 | 5.9E+08 | 1.52524 | 0.0027 | 0.60904 | up |
| WP_011240187.1 | ATP synthase F1 subunit epsilon | ZMO_RS01050*- | 5.1E+08 | 6.3E+08 | 4.9E+08 | 1.1E+09 | 1.1E+09 | 1.1E+09 | 0.50338 | 0.00464 | -0.99027 | down |
| WP_011240833.1 | ABC transporter ATP-binding protein | ZMO_RS04395*- | 3.8E+07 | 2.1E+07 | 2E+07 | 5.6E+07 | 4.8E+07 | 4.8E+07 | 0.51782 | 0.03472 | -0.94949 | down |
| WP_011240734.1 | glycosyltransferase | ZMO_RS03880*- | 4.5E+07 | 5.6E+07 | 3.7E+07 | 8.7E+07 | 9.5E+07 | 1.2E+08 | 0.45341 | 0.01767 | -1.14112 | down |
| WP_011241089.1 | glycosyltransferase family 4 protein | ZMO_RS05755*- | 7.7E+07 | 8.5E+07 | 1E+08 | 3.1E+08 | 2.5E+08 | 3E+08 | 0.31116 | 0.00217 | -1.68428 | down |
| WP_011240060.1 | hypothetical protein | ZMO_RS00365*- | 2.7E+07 | 4E+07 | 4.9E+07 | 9.8E+07 | 1E+08 | 1.3E+08 | 0.34978 | 0.00534 | -1.5155 | down |
| WP_011240241.1 | capsular polysaccharide biosynthesis protein | ZMO_RS01325*- | 2.9E+07 | 1.7E+07 | 2.4E+07 | 3.5E+07 | 3.6E+07 | 4.8E+07 | 0.59158 | 0.03762 | -0.75735 | down |
| WP_011240095.1 | phosphatase PAP2 family protein | ZMO_RS00545*- | 5.3E+07 | 7.5E+07 | 6.2E+07 | 1.1E+08 | 1.1E+08 | 9.7E+07 | 0.59377 | 0.00734 | -0.75203 | down |
| WP_011240603.1 | hypothetical protein | ZMO_RS03195*- | 8.9E+07 | 8.1E+07 | 4.6E+07 | 1.4E+08 | 1.3E+08 | 1.5E+08 | 0.51854 | 0.01927 | -0.94747 | down |
| WP_011240634.1 | glutaredoxin 3 | ZMO_RS03370*grxC | 1E+09 | 7.7E+08 | 9.7E+08 | 3E+08 | 2.4E+08 | 3.9E+08 | 2.93317 | 0.00466 | 1.55246 | up |
| WP_011241358.1 | alkaline phosphatase family protein | ZMO_RS07200*- | 8.6E+07 | 9.9E+07 | 9.4E+07 | 1.6E+08 | 1.2E+08 | 1.6E+08 | 0.62771 | 0.04119 | -0.67182 | down |
| WP_011240171.1 | methionine biosynthesis protein MetW | ZMO_RS00960*metW | 5.5E+07 | 4.1E+07 | 3.7E+07 | 7.6E+07 | 6.4E+07 | 9.4E+07 | 0.56805 | 0.04007 | -0.81592 | down |
| WP_011241161.1 | ribonuclease III | ZMO_RS06145*rnc | 2.1E+08 | 1.9E+08 | 1.9E+08 | 2.6E+08 | 3.1E+08 | 3.3E+08 | 0.65636 | 0.0271 | -0.60745 | down |
| WP_011241366.1 | ABC transporter substrate-binding protein | ZMO_RS07240*- | 3E+08 | 2.6E+08 | 2.9E+08 | 1.5E+08 | 1.1E+08 | 1.7E+08 | 1.96583 | 0.0025 | 0.97514 | up |
| WP_011240144.1 | DsbA family protein | ZMO_RS00820*- | 1.3E+08 | 8.1E+07 | 1E+08 | 2.3E+08 | 2.6E+08 | 2.8E+08 | 0.40028 | 0.0012 | -1.32091 | down |
| WP_011240651.1 | methyltransferase type 12 | ZMO_RS03455*- | 4.8E+07 | 2.4E+07 | 4.7E+07 | 1.1E+08 | 8.2E+07 | 1.1E+08 | 0.39275 | 0.00808 | -1.34832 | down |
| WP_011241370.1 | GatB/YqeY domain-containing protein | ZMO_RS07265*- | 1.3E+09 | 1.3E+09 | 1.2E+09 | 5.9E+08 | 5E+08 | 6.2E+08 | 2.20738 | 0.00053 | 1.14234 | up |
| WP_011240552.1 | HAD-IB family hydrolase | ZMO_RS02925*- | 4.8E+07 | 4.5E+07 | 5.7E+07 | 1.1E+08 | 9.4E+07 | 1.2E+08 | 0.47068 | 0.00492 | -1.08717 | down |
| WP_011241105.1 | pyrroline-5-carboxylate reductase | ZMO_RS05835*proC | 2.8E+08 | 2.7E+08 | 2.4E+08 | 1.7E+08 | 1.5E+08 | 1.7E+08 | 1.57096 | 0.00501 | 0.65164 | up |
| WP_011240213.1 | apolipoprotein N-acyltransferase | ZMO_RS01180*lnt | 9.3E+07 | 1E+08 | 1E+08 | 1.9E+08 | 1.6E+08 | 1.7E+08 | 0.56575 | 0.0071 | -0.82177 | down |
| WP_011241407.1 | tRNA lysidine(34) synthetase TilS | ZMO_RS07450*tilS | 6.8E+07 | 7.4E+07 | 6.3E+07 | 1.6E+08 | 1.1E+08 | 1.5E+08 | 0.49147 | 0.0356 | -1.02484 | down |
| WP_011240141.1 | TonB-dependent siderophore receptor | ZMO_RS00805*- | 2501135 | 1779801 | 2789556 | 1.2E+07 | 8676616 | 1.5E+07 | 0.19997 | 0.03174 | -2.32216 | down |
| WP_011241133.1 | NAD(P)H:quinone oxidoreductase | ZMO_RS05975*wrbA | 1.1E+09 | 1.3E+09 | 1.3E+09 | 5.6E+08 | 6.5E+08 | 7.2E+08 | 1.86424 | 0.00266 | 0.89859 | up |
| WP_011241636.1 | aminoacyl-tRNA hydrolase | ZMO_RS08675*- | 4.7E+07 | 6.4E+07 | 6.3E+07 | 1E+08 | 9.3E+07 | 8.2E+07 | 0.62533 | 0.01523 | -0.6773 | down |
| WP_011240167.1 | ferredoxin family protein | ZMO_RS00940*- | 6.3E+08 | 6.8E+08 | 6.1E+08 | 2.9E+08 | 2.7E+08 | 3.1E+08 | 2.2239 | 0.00044 | 1.15309 | up |
| WP_011240508.1 | flagellar basal body P-ring protein FlgI | ZMO_RS02700*flgI | 4.6E+07 | 1.2E+07 | 9233682 | 1.1E+08 | 8.9E+07 | 8.3E+07 | 0.23837 | 0.01051 | -2.06874 | down |
| WP_011240022.1 | PTS transporter subunit EIIA | ZMO_RS00170*- | 1.6E+08 | 1.9E+08 | 1.5E+08 | 9.5E+07 | 5.3E+07 | 1E+08 | 1.96737 | 0.01633 | 0.97627 | up |
| WP_011240159.1 | GNAT family N-acetyltransferase | ZMO_RS00900*- | 8.9E+07 | 7.2E+07 | 5.8E+07 | 3.6E+07 | 3.1E+07 | 4.3E+07 | 2.00498 | 0.04074 | 1.00359 | up |
| WP_011240110.1 | tRNA (guanosine(46)-N7)-methyltransferase TrmB | ZMO_RS00625*trmB | 5.9E+07 | 5.4E+07 | 4.1E+07 | 1.2E+08 | 1.4E+08 | 1.2E+08 | 0.40517 | 0.00061 | -1.30342 | down |
| WP_011241518.1 | antibiotic biosynthesis monooxygenase | ZMO_RS08010*- | 1.5E+10 | 1.6E+10 | 1.7E+10 | 6E+09 | 6.1E+09 | 7E+09 | 2.49507 | 0.00039 | 1.31908 | up |
| WP_011240766.1 | glucans biosynthesis glucosyltransferase MdoH | ZMO_RS04045*mdoH | 4.8E+07 | 4.6E+07 | 5.2E+07 | 9E+07 | 8.4E+07 | 1.2E+08 | 0.50696 | 0.03527 | -0.98006 | down |
| WP_011240916.1 | AAA family ATPase | ZMO_RS04835*- | 6.1E+07 | 5.3E+07 | 4E+07 | 1.3E+08 | 1E+08 | 1.1E+08 | 0.46535 | 0.00502 | -1.1036 | down |
| WP_011240853.1 | ribonuclease HII | ZMO_RS04490*- | 2.5E+07 | 2.9E+07 | 3.5E+07 | 5.7E+07 | 5.9E+07 | 7.7E+07 | 0.4567 | 0.01908 | -1.13067 | down |
| WP_011241001.1 | phosphoribosyl-AMP cyclohydrolase | ZMO_RS05275*hisI | 3.3E+08 | 2.7E+08 | 2.9E+08 | 2E+08 | 1.7E+08 | 2E+08 | 1.5432 | 0.01384 | 0.62593 | up |
| WP_011240825.1 | glycosyltransferase | ZMO_RS04350*- | 3.2E+07 | 2.4E+07 | 3.6E+07 | 5.3E+07 | 5.4E+07 | 6.6E+07 | 0.53124 | 0.00795 | -0.91257 | down |
| WP_011241476.1 | serine acetyltransferase | ZMO_RS07795*- | 1.9E+08 | 1.8E+08 | 1.8E+08 | 3.5E+08 | 3.2E+08 | 3.5E+08 | 0.54018 | 0.00042 | -0.8885 | down |
| WP_011241546.1 | RnfABCDGE type electron transport complex subunit B | ZMO_RS08150*- | 4.5E+07 | 4.9E+07 | 4.3E+07 | 8.7E+07 | 1.2E+08 | 1.3E+08 | 0.39962 | 0.03663 | -1.32331 | down |
| WP_011241329.1 | pyruvate formate lyase-activating protein | ZMO_RS07010*pflA | 6.3E+07 | 5.4E+07 | 6.2E+07 | 3.5E+07 | 3.9E+07 | 4.3E+07 | 1.52343 | 0.00656 | 0.60732 | up |
| WP_011240994.1 | antibiotic biosynthesis monooxygenase | ZMO_RS05240*- | 2.7E+07 | 2.8E+07 | 3E+07 | 6.6E+07 | 5.3E+07 | 6.1E+07 | 0.47647 | 0.01216 | -1.06953 | down |
| WP_011241008.1 | uracil-DNA glycosylase | ZMO_RS05310*- | 1.9E+07 | 1.3E+07 | 1.7E+07 | 3.6E+07 | 2.9E+07 | 3.6E+07 | 0.49119 | 0.00639 | -1.02564 | down |
| WP_011241357.1 | TIGR01244 family phosphatase | ZMO_RS07195*- | 1.3E+08 | 1.1E+08 | 9.9E+07 | 3E+07 | 2.7E+07 | 3.1E+07 | 3.84326 | 0.0085 | 1.94233 | up |
| WP_011240040.1 | aldo/keto reductase | ZMO_RS00265*- | 3E+08 | 2.7E+08 | 3.1E+08 | 2.2E+08 | 1.8E+08 | 1.9E+08 | 1.51294 | 0.00603 | 0.59735 | up |
| WP_011241014.1 | dUTP diphosphatase | ZMO_RS05345*- | 4.8E+07 | 2.7E+07 | 2.8E+07 | 9.1E+07 | 7.3E+07 | 5.8E+07 | 0.46284 | 0.033 | -1.1114 | down |
| WP_011240104.1 | low molecular weight phosphotyrosine protein phosphatase | ZMO_RS00590*- | 2.8E+08 | 3.1E+08 | 3.3E+08 | 1.7E+08 | 2.3E+08 | 2.1E+08 | 1.50846 | 0.01248 | 0.59308 | up |
| WP_011240798.1 | guanine deaminase | ZMO_RS04200*guaD | 2.5E+07 | 2.8E+07 | 2.1E+07 | 3.4E+07 | 4.2E+07 | 4.1E+07 | 0.63402 | 0.01267 | -0.6574 | down |
| WP_011240164.1 | 5-formyltetrahydrofolate cyclo-ligase | ZMO_RS00925*- | 1.4E+08 | 1.3E+08 | 1.3E+08 | 5.4E+07 | 5.8E+07 | 5.7E+07 | 2.33744 | 0.0028 | 1.22493 | up |
| WP_011241519.1 | monooxygenase | ZMO_RS08015*- | 3.8E+08 | 3.4E+08 | 3.8E+08 | 1.5E+08 | 2.4E+08 | 1.9E+08 | 1.86809 | 0.00782 | 0.90156 | up |
| WP_011240483.1 | dicarboxylate/amino acid:cation symporter | ZMO_RS02575*- | 6.8E+07 | 9.1E+07 | 1E+08 | 1.3E+08 | 1.5E+08 | 1.9E+08 | 0.54526 | 0.04901 | -0.87498 | down |
| WP_011241573.1 | FAD:protein FMN transferase | ZMO_RS08290*- | 3.2E+07 | 3.4E+07 | 2.5E+07 | 8.5E+07 | 9E+07 | 9.1E+07 | 0.34269 | 0.00011 | -1.54502 | down |
| WP_011241108.1 | TlyA family RNA methyltransferase | ZMO_RS05850*- | 2.3E+07 | 1.8E+07 | 1.5E+07 | 3.8E+07 | 3.9E+07 | 5.5E+07 | 0.42437 | 0.02816 | -1.23662 | down |
| WP_011241467.1 | VOC family protein | ZMO_RS07750*- | 9.1E+08 | 8.9E+08 | 1E+09 | 3.1E+08 | 3.3E+08 | 2.3E+08 | 3.21488 | 0.0002 | 1.68476 | up |
| WP_011241150.1 | chromosomal replication initiator protein DnaA | ZMO_RS06065*dnaA | 3.9E+07 | 4.4E+07 | 3.5E+07 | 7.2E+07 | 7.3E+07 | 7.5E+07 | 0.53713 | 0.00241 | -0.89666 | down |
| WP_011241625.1 | 50S ribosomal protein L32 | ZMO_RS09205*rpmF | 1.5E+08 | 5.3E+07 | 5.8E+07 | 6.4E+08 | 7.1E+08 | 6.7E+08 | 0.12836 | 0.00026 | -2.96168 | down |
| WP_011240868.1 | type III PLP-dependent enzyme | ZMO_RS04575*- | 3288591 | 2110900 | 1898810 | 8.6E+07 | 6.8E+07 | 8.4E+07 | 0.03059 | 0.00502 | -5.03081 | down |
| WP_017466302.1 | uracil-DNA glycosylase | ZMO_RS07400*- | 9167531 | 5391062 | 3651161 | 2.3E+07 | 1.5E+07 | 2.6E+07 | 0.28652 | 0.02348 | -1.80327 | down |
| WP_011241199.1 | NifU family protein | ZMO_RS06335*- | 7.9E+08 | 6.9E+08 | 6.5E+08 | 4.6E+08 | 3.9E+08 | 4.9E+08 | 1.58645 | 0.00786 | 0.6658 | up |
| WP_011240845.1 | mechanosensitive ion channel family protein | ZMO_RS04450*- | 1.9E+07 | 1.5E+07 | 2.2E+07 | 2.7E+07 | 3.3E+07 | 4E+07 | 0.55746 | 0.03403 | -0.84306 | down |
| WP_011240013.1 | sel1 repeat family protein | ZMO_RS00125*- | 0 | 0 | 0 | 1.7E+07 | 1.2E+07 | 4093065 | #NAME? | 0 | #NAME? | down |
| WP_011241175.1 | acylglycerol kinase family protein | ZMO_RS06215*- | 7E+07 | 7.5E+07 | 6.7E+07 | 9.5E+07 | 1.2E+08 | 1.3E+08 | 0.62619 | 0.04269 | -0.67533 | down |
| WP_011241182.1 | sterol desaturase family protein | ZMO_RS06250*- | 5E+07 | 3.4E+07 | 3.6E+07 | 8E+07 | 1E+08 | 8.1E+07 | 0.45561 | 0.00896 | -1.13414 | down |
| WP_012817354.1 | glycoside hydrolase family 68 protein | ZMO_RS01605*- | 6266063 | 9199235 | 9235642 | 2.9E+07 | 2E+07 | 2.2E+07 | 0.35022 | 0.02558 | -1.51368 | down |
| WP_011240376.1 | LysR family transcriptional regulator | ZMO_RS02020*- | 2.5E+07 | 1.9E+07 | 1.8E+07 | 3.2E+07 | 3.5E+07 | 3.5E+07 | 0.6027 | 0.01359 | -0.73048 | down |
| WP_011241709.1 | dienelactone hydrolase family protein | ZMO_RS09075*- | 4E+07 | 3E+07 | 2.6E+07 | 2E+07 | 1.1E+07 | 1.9E+07 | 1.92347 | 0.04462 | 0.94371 | up |
| WP_011241084.1 | acyl carrier protein | ZMO_RS05730*- | 2.9E+09 | 2.9E+09 | 2.5E+09 | 1.4E+09 | 1.2E+09 | 1.3E+09 | 2.13586 | 0.00446 | 1.09482 | up |
| WP_011240948.1 | glutathione S-transferase family protein | ZMO_RS05000*- | 1.3E+08 | 1.2E+08 | 2.1E+08 | 2.8E+08 | 2.4E+08 | 2.7E+08 | 0.58448 | 0.04237 | -0.77478 | down |
| WP_011240160.1 | 50S ribosomal protein L27 | ZMO_RS00905*rpmA | 6.4E+08 | 7.4E+08 | 5.8E+08 | 2.6E+09 | 3.6E+09 | 3.1E+09 | 0.21228 | 0.01133 | -2.23594 | down |
| WP_011240522.1 | P-loop NTPase | ZMO_RS02775*- | 9.6E+07 | 1.1E+08 | 1.2E+08 | 1.5E+08 | 1.7E+08 | 1.9E+08 | 0.64037 | 0.01714 | -0.64302 | down |
| WP_011240115.1 | Holliday junction branch migration protein RuvA | ZMO_RS00655*ruvA | 6.1E+08 | 9.1E+08 | 7.7E+08 | 1.1E+08 | 1.1E+08 | 1.4E+08 | 6.31638 | 0.01569 | 2.6591 | up |
| WP_011241444.1 | zinc-binding alcohol dehydrogenase family protein | ZMO_RS07630*- | 6.1E+07 | 4.4E+07 | 4.4E+07 | 2.9E+07 | 1.2E+07 | 2.1E+07 | 2.42517 | 0.01732 | 1.27809 | up |
| WP_011241513.1 | aldo/keto reductase | ZMO_RS07980*- | 8.3E+07 | 7.4E+07 | 6.4E+07 | 3.7E+07 | 3.4E+07 | 4.5E+07 | 1.91218 | 0.00852 | 0.93522 | up |
| WP_011240502.1 | response regulator | ZMO_RS02665*- | 7943183 | 5280282 | 3277856 | 1.5E+07 | 1.4E+07 | 2E+07 | 0.34256 | 0.01439 | -1.54556 | down |
| WP_011241641.1 | hypothetical protein | ZMO_RS08700*- | 2.5E+07 | 1.8E+07 | 2.3E+07 | 4E+07 | 4E+07 | 3.4E+07 | 0.58142 | 0.00488 | -0.78235 | down |
| WP_011240343.1 | iron-sulfur cluster assembly accessory protein | ZMO_RS01850*- | 1.2E+08 | 9.5E+07 | 1.1E+08 | 4.6E+07 | 4.4E+07 | 4.1E+07 | 2.45537 | 0.00631 | 1.29594 | up |
| WP_011241271.1 | histidine triad nucleotide-binding protein | ZMO_RS06715*- | 4E+08 | 4.2E+08 | 4.8E+08 | 2.2E+08 | 2.3E+08 | 2.3E+08 | 1.91718 | 0.01268 | 0.93898 | up |
| WP_011240742.1 | HpnM family protein | ZMO_RS03920*- | 9.7E+08 | 9.9E+08 | 1E+09 | 4.3E+08 | 3.6E+08 | 3.9E+08 | 2.53507 | 2.18E-05 | 1.34203 | up |
| WP_011241452.1 | peptidase S16 | ZMO_RS07670*- | 1.7E+08 | 1.7E+08 | 1.4E+08 | 1E+08 | 1E+08 | 1E+08 | 1.59273 | 0.03178 | 0.6715 | up |
| WP_011241541.1 | RnfH family protein | ZMO_RS08125*- | 8.1E+07 | 9.7E+07 | 8.5E+07 | 1.2E+08 | 1.5E+08 | 1.7E+08 | 0.6052 | 0.04306 | -0.72451 | down |
| WP_011240984.1 | aspartate/glutamate racemase family protein | ZMO_RS05190*- | 7E+07 | 6.1E+07 | 6.5E+07 | 2.7E+07 | 1.3E+07 | 2.8E+07 | 2.89172 | 0.00373 | 1.53193 | up |
| WP_011241128.1 | HPr family phosphocarrier protein | ZMO_RS05950*- | 1.9E+08 | 1.8E+08 | 1.3E+08 | 9.5E+07 | 3.8E+07 | 1E+08 | 2.10215 | 0.0341 | 1.07186 | up |
| WP_011240090.1 | hypothetical protein | ZMO_RS00515*- | 1.5E+08 | 1.6E+08 | 1.3E+08 | 4.4E+07 | 4.8E+07 | 5E+07 | 3.18177 | 0.00649 | 1.66983 | up |
| WP_011240913.1 | tRNA (guanosine(37)-N1)-methyltransferase TrmD | ZMO_RS04820*trmD | 1.1E+07 | 1.2E+07 | 1.2E+07 | 2.9E+07 | 3.5E+07 | 2.9E+07 | 0.3802 | 0.0095 | -1.39518 | down |
| WP_011240443.1 | preprotein translocase subunit SecY | ZMO_RS02360*secY | 1.7E+08 | 1.7E+08 | 1.7E+08 | 3.2E+08 | 3.7E+08 | 3.9E+08 | 0.47271 | 0.01128 | -1.08097 | down |
| WP_011240559.1 | YdbL family protein | ZMO_RS02960*- | 5.1E+08 | 4E+08 | 5E+08 | 2E+08 | 1.8E+08 | 2E+08 | 2.46083 | 0.01199 | 1.29915 | up |
| WP_011240332.1 | cell division protein | ZMO_RS01795*- | 1054516 | 534063 | 1551829 | 1.1E+07 | 1.2E+07 | 1.8E+07 | 0.07546 | 0.0276 | -3.72818 | down |
| WP_011241389.1 | murein biosynthesis integral membrane protein MurJ | ZMO_RS07355*murJ | 1.6E+07 | 1.8E+07 | 2.4E+07 | 3.2E+07 | 4.5E+07 | 4.6E+07 | 0.46997 | 0.02616 | -1.08935 | down |
| WP_011240694.1 | hypothetical protein | ZMO_RS03680*- | 2.7E+07 | 0 | 1.5E+07 | 3.8E+07 | 6.4E+07 | 6E+07 | 0.39509 | 0.04958 | -1.33973 | down |
| WP_011240245.1 | L-2-amino-thiazoline-4-carboxylic acid hydrolase | ZMO_RS01345*- | 2.2E+07 | 2.1E+07 | 2E+07 | 3019393 | 6463759 | 1.2E+07 | 2.96727 | 0.03036 | 1.56914 | up |
| WP_011241668.1 | PhzF family phenazine biosynthesis protein | ZMO_RS08860*- | 1E+07 | 7215598 | 8478990 | 3212627 | 3396515 | 1139005 | 3.36893 | 0.00763 | 1.75229 | up |
| WP_011240348.1 | glycine zipper 2TM domain-containing protein | ZMO_RS01875*- | 2.3E+08 | 2.1E+08 | 2.8E+08 | 3.6E+08 | 4.6E+08 | 3.7E+08 | 0.60856 | 0.02118 | -0.71653 | down |
| WP_011241601.1 | Grx4 family monothiol glutaredoxin | ZMO_RS08485*grxD | 2.7E+08 | 3.3E+08 | 3.6E+08 | 1.1E+08 | 1.3E+08 | 1.1E+08 | 2.72467 | 0.01635 | 1.44608 | up |
| WP_011241572.1 | SoxR reducing system RseC family protein | ZMO_RS08285*- | 1.2E+07 | 6440243 | 1.1E+07 | 4.1E+07 | 3.6E+07 | 2.7E+07 | 0.28862 | 0.01586 | -1.79277 | down |
| WP_011240079.1 | glycosyltransferase | ZMO_RS00460*- | 4778876 | 5693217 | 1.5E+07 | 2.4E+07 | 2.3E+07 | 3E+07 | 0.32515 | 0.01416 | -1.62084 | down |
| WP_011240533.1 | hypothetical protein | ZMO_RS02830*- | 8838640 | 6361166 | 7288311 | 1.7E+07 | 1.3E+07 | 1.4E+07 | 0.50627 | 0.01283 | -0.98202 | down |
| WP_011241270.1 | amino acid permease | ZMO_RS06710*- | 0 | 0 | 0 | 4858806 | 2.2E+07 | 2E+07 | #NAME? | 0 | #NAME? | down |
| WP_014501114.1 | septation ring formation regulator EzrA | ZMO_RS07215*- | 4154051 | 0 | 926145 | 4.3E+07 | 2.9E+07 | 4.2E+07 | 0.06699 | 0.00882 | -3.89996 | down |
| WP_080502943.1 | redox-sensitive transcriptional activator SoxR | ZMO_RS09130*soxR | 0 | 0 | 0 | 1359856 | 2982375 | 2855865 | #NAME? | 0 | #NAME? | down |
| WP_011240627.1 | hypothetical protein | ZMO_RS03335*- | 3.6E+07 | 4.2E+07 | 4.6E+07 | 8.2E+07 | 6.5E+07 | 6.5E+07 | 0.58943 | 0.02004 | -0.76261 | down |
| WP_011241697.1 | delta(1)-pyrroline-2-carboxylate reductase family protein | ZMO_RS09015*- | 3.8E+07 | 3.7E+07 | 2.5E+07 | 5E+07 | 6.8E+07 | 6E+07 | 0.55738 | 0.02088 | -0.84326 | down |
| WP_011241333.1 | DUF2474 family protein | ZMO_RS07035*- | 3.5E+07 | 3.2E+07 | 2.6E+07 | 6E+07 | 4.5E+07 | 6.9E+07 | 0.53426 | 0.04425 | -0.90439 | down |
| WP_011240085.1 | Rrf2 family transcriptional regulator | ZMO_RS00490*- | 3.1E+07 | 2.7E+07 | 5E+07 | 1E+08 | 8.5E+07 | 8.9E+07 | 0.39165 | 0.00449 | -1.35237 | down |
| WP_012817615.1 | epoxyqueuosine reductase QueH | ZMO_RS08540*- | 5477370 | 6787217 | 5547346 | 1.4E+07 | 1.4E+07 | 1.5E+07 | 0.40581 | 0.00063 | -1.30111 | down |
| WP_011241266.1 | DedA family protein | ZMO_RS06690*- | 3135282 | 4117432 | 3656654 | 1.8E+07 | 2E+07 | 2.5E+07 | 0.17489 | 0.01434 | -2.5155 | down |
| WP_011240322.1 | ABC transporter permease | ZMO_RS01745*- | 3.1E+07 | 4.1E+07 | 2.8E+07 | 5E+07 | 5.4E+07 | 4.8E+07 | 0.65612 | 0.03703 | -0.60796 | down |
| WP_011241544.1 | RnfABCDGE type electron transport complex subunit D | ZMO_RS08140*- | 5E+07 | 4.4E+07 | 5.4E+07 | 7.6E+07 | 8.6E+07 | 8.9E+07 | 0.59069 | 0.00282 | -0.75952 | down |
| WP_011239990.1 | aminotransferase class I/II-fold pyridoxal phosphate-dependent enzyme | ZMO_RS00010*- | 1.4E+07 | 1.3E+07 | 1.4E+07 | 3E+07 | 2.6E+07 | 2.6E+07 | 0.48637 | 0.00489 | -1.03986 | down |
| WP_011240316.1 | tail fiber domain-containing protein | ZMO_RS01710*- | 1569276 | 3294038 | 2179400 | 7286655 | 4828712 | 6371224 | 0.38096 | 0.01538 | -1.39228 | down |
| WP_011241474.1 | hypothetical protein | ZMO_RS07785*- | 5814200 | 6348507 | 2847994 | 8330292 | 1.5E+07 | 1.3E+07 | 0.41898 | 0.04539 | -1.25505 | down |
| WP_011240175.1 | lipopolysaccharide biosynthesis protein | ZMO_RS00980*- | 3.3E+07 | 4.1E+07 | 4.5E+07 | 8.1E+07 | 9E+07 | 9.1E+07 | 0.45015 | 0.00058 | -1.15153 | down |
| WP_011240436.1 | 30S ribosomal protein S14 | ZMO_RS02325*rpsN | 1.3E+09 | 1.5E+09 | 1.6E+09 | 6.3E+08 | 9.4E+08 | 9.6E+08 | 1.7292 | 0.01192 | 0.7901 | up |
| WP_011240906.1 | J domain-containing protein | ZMO_RS04785*- | 9371729 | 7947324 | 9120573 | 1.8E+07 | 1.5E+07 | 1.3E+07 | 0.58447 | 0.03871 | -0.7748 | down |
| WP_011241677.1 | 3-methyl-2-oxobutanoate hydroxymethyltransferase | ZMO_RS08905*panB | 0 | 0 | 0 | 3013111 | 1585541 | 2747498 | #NAME? | 0 | #NAME? | down |
| WP_011240681.1 | DUF167 domain-containing protein | ZMO_RS03605*- | 6.5E+07 | 5.4E+07 | 6.4E+07 | 4.1E+07 | 3.4E+07 | 3.7E+07 | 1.63605 | 0.00724 | 0.71021 | up |
| WP_011240923.1 | septal ring lytic transglycosylase RlpA family protein | ZMO_RS04865*- | 0 | 0 | 0 | 4454659 | 2554322 | 4401740 | #NAME? | 0 | #NAME? | down |
| WP_011240896.1 | N-acetyltransferase | ZMO_RS04730*- | 1.6E+08 | 1.8E+08 | 1.5E+08 | 9E+07 | 7.4E+07 | 1.1E+08 | 1.73726 | 0.01175 | 0.79682 | up |
| WP_011240069.1 | MFS transporter | ZMO_RS00410*- | 1025472 | 931522 | 749055 | 1.5E+07 | 1.3E+07 | 2.6E+07 | 0.05025 | 0.04955 | -4.31468 | down |
| WP_011241251.1 | hypothetical protein | ZMO_RS06605*- | 3.4E+07 | 3.6E+07 | 3.4E+07 | 2.6E+07 | 2E+07 | 2.3E+07 | 1.52477 | 0.00985 | 0.60859 | up |
| WP_011241311.1 | J domain-containing protein | ZMO_RS06915*- | 2322109 | 0 | 3479891 | 2.1E+07 | 1.3E+07 | 1.9E+07 | 0.16345 | 0.01702 | -2.61306 | down |
| WP_011240536.1 | flagellar basal body-associated FliL family protein | ZMO_RS02845*- | 2.6E+07 | 2E+07 | 2.9E+07 | 5.2E+07 | 3.9E+07 | 4.3E+07 | 0.56655 | 0.01811 | -0.81972 | down |
| WP_011241245.1 | ABC transporter permease | ZMO_RS06575*- | 1.8E+07 | 2.4E+07 | 2.7E+07 | 5.7E+07 | 5E+07 | 6.2E+07 | 0.40269 | 0.00155 | -1.31224 | down |
| WP_011240876.1 | ABC transporter permease subunit | ZMO_RS04615*- | 1.6E+07 | 1.6E+07 | 9736750 | 2.5E+07 | 3E+07 | 2.5E+07 | 0.52053 | 0.00933 | -0.94195 | down |
| WP_011240358.1 | MBOAT family protein | ZMO_RS01925*- | 4.1E+07 | 4.3E+07 | 3.8E+07 | 6.2E+07 | 6.5E+07 | 7E+07 | 0.61889 | 0.00256 | -0.69225 | down |
| WP_011240337.1 | SUF system Fe-S cluster assembly regulator | ZMO_RS01820*- | 3.2E+07 | 2.5E+07 | 2.7E+07 | 2.1E+07 | 1.5E+07 | 1.9E+07 | 1.5283 | 0.02506 | 0.61193 | up |
| WP_011240958.1 | type II toxin-antitoxin system RatA family toxin | ZMO_RS05055*- | 2.4E+07 | 2.3E+07 | 2.3E+07 | 3.2E+07 | 3.7E+07 | 3.6E+07 | 0.65851 | 0.01121 | -0.60272 | down |
| WP_011240192.1 | 50S ribosomal protein L33 | ZMO_RS01075*rpmG | 3.9E+08 | 4E+08 | 6.5E+08 | 1.3E+09 | 2.1E+09 | 1.6E+09 | 0.28866 | 0.02029 | -1.79254 | down |
| WP_011240688.1 | 6-carboxytetrahydropterin synthase QueD | ZMO_RS03645*queD | 8.8E+07 | 8E+07 | 9.7E+07 | 1.7E+08 | 1.6E+08 | 1.5E+08 | 0.5544 | 0.00045 | -0.851 | down |
| WP_011240781.1 | ASCH domain-containing protein | ZMO_RS04125*- | 3.5E+07 | 3E+07 | 3.2E+07 | 1.8E+07 | 1.8E+07 | 2.5E+07 | 1.56786 | 0.01958 | 0.6488 | up |
| WP_011241043.1 | 30S ribosomal protein S18 | ZMO_RS05510*rpsR | 1.7E+09 | 1.8E+09 | 1.7E+09 | 4.9E+08 | 5.5E+08 | 5.3E+08 | 3.31839 | 1.69E-05 | 1.73048 | up |
| WP_011240762.1 | TonB-dependent receptor plug domain-containing protein | ZMO_RS04025*- | 0 | 0 | 0 | 2309754 | 5866654 | 2121883 | #NAME? | 0 | #NAME? | down |
| WP_011241218.1 | LysE family translocator | ZMO_RS06435*- | 9675868 | 9220757 | 5317715 | 1.8E+07 | 1.2E+07 | 1.7E+07 | 0.5211 | 0.03026 | -0.94037 | down |
| WP_011240253.1 | signal peptidase II | ZMO_RS01390*lspA | 0 | 0 | 0 | 2.4E+07 | 1.8E+07 | 1.8E+07 | #NAME? | 0 | #NAME? | down |
| WP_011240869.1 | alpha/beta hydrolase | ZMO_RS04580*- | 1.6E+08 | 1.4E+08 | 1.4E+08 | 7.8E+07 | 7.6E+07 | 7.1E+07 | 1.96536 | 0.00854 | 0.97479 | up |
| WP_011240985.1 | CDP-diacylglycerol--serine O-phosphatidyltransferase | ZMO_RS05195*pssA | 1.5E+07 | 1.1E+07 | 1.6E+07 | 2.9E+07 | 2.9E+07 | 3.6E+07 | 0.45502 | 0.00516 | -1.13599 | down |
| WP_011241061.1 | cytochrome c biosynthesis protein | ZMO_RS05610*- | 8535132 | 6139989 | 8185069 | 1.6E+07 | 2.3E+07 | 2.3E+07 | 0.36976 | 0.02705 | -1.43533 | down |
| WP_011241712.1 | CopD family protein | ZMO_RS09090*- | 1.3E+07 | 1.1E+07 | 1.3E+07 | 1.8E+07 | 2.3E+07 | 2.6E+07 | 0.56396 | 0.04707 | -0.82634 | down |
| WP_011240333.1 | YdcF family protein | ZMO_RS01800*- | 0 | 0 | 0 | 1944894 | 3720293 | 8714792 | #NAME? | 0 | #NAME? | down |
| WP_011241064.1 | heme lyase CcmF/NrfE family subunit | ZMO_RS05625*- | 5677407 | 3524370 | 2514478 | 1E+07 | 8988084 | 9995907 | 0.40016 | 0.01374 | -1.32134 | down |
| WP_011240993.1 | ATPase | ZMO_RS05235*- | 2.3E+07 | 2.9E+07 | 2.7E+07 | 1.6E+07 | 1.7E+07 | 1.8E+07 | 1.52095 | 0.01782 | 0.60498 | up |
| WP_011240180.1 | hypothetical protein | ZMO_RS01010*- | 4.6E+07 | 3.8E+07 | 4E+07 | 1.9E+07 | 1.4E+07 | 2.7E+07 | 2.04521 | 0.01442 | 1.03225 | up |
| WP_011241289.1 | 50S ribosomal protein L35 | ZMO_RS09185*rpmI | 1.3E+09 | 1.2E+09 | 8.5E+08 | 1.2E+08 | 1.1E+08 | 3.6E+08 | 5.59131 | 0.00775 | 2.48319 | up |
| WP_011240359.1 | hypothetical protein | ZMO_RS01930*- | 1.6E+07 | 2.1E+07 | 1.7E+07 | 2.9E+07 | 2.6E+07 | 3.4E+07 | 0.61075 | 0.02011 | -0.71135 | down |
| WP_011240813.1 | monofunctional biosynthetic peptidoglycan transglycosylase | ZMO_RS04290*mtgA | 0 | 315925 | 1065557 | 2920439 | 3505317 | 3958659 | 0.19955 | 0.02211 | -2.32517 | down |
| WP_011240578.1 | histidine kinase | ZMO_RS03055*- | 0 | 0 | 0 | 3624100 | 2803009 | 5004310 | #NAME? | 0 | #NAME? | down |
| WP_011240562.1 | F0F1 ATP synthase subunit C | ZMO_RS02975*- | 1E+09 | 8.1E+08 | 5.1E+08 | 5.5E+07 | 3.5E+07 | 2E+08 | 7.98012 | 0.03296 | 2.99641 | up |
| WP_011240814.1 | cytochrome c | ZMO_RS04295*- | 5.7E+07 | 5.3E+07 | 6.1E+07 | 2.2E+07 | 2.3E+07 | 3.5E+07 | 2.11334 | 0.00545 | 1.07953 | up |
| WP_011240978.1 | phosphatidate cytidylyltransferase | ZMO_RS05155*- | 2E+07 | 1.7E+07 | 2.9E+07 | 4.7E+07 | 4.7E+07 | 5.5E+07 | 0.44077 | 0.00418 | -1.18192 | down |
| WP_011241589.1 | VIT family protein | ZMO_RS08395*- | 4904041 | 2662181 | 0 | 2.4E+07 | 1.4E+07 | 2E+07 | 0.19255 | 0.01995 | -2.37669 | down |
| WP_011240078.1 | hypothetical protein | ZMO_RS00455*- | 1.2E+07 | 1.6E+07 | 1.6E+07 | 4.4E+07 | 4.5E+07 | 5.6E+07 | 0.2953 | 0.00769 | -1.75976 | down |
| WP_011240515.1 | flagellar basal body rod protein FlgB | ZMO_RS02735*flgB | 4874183 | 3588861 | 6250558 | 9860926 | 6799374 | 1E+07 | 0.55135 | 0.0419 | -0.85895 | down |
| WP_011241201.1 | 4-hydroxybenzoate octaprenyltransferase | ZMO_RS06350*- | 4341966 | 2422314 | 3020203 | 6052081 | 4532657 | 6079374 | 0.58716 | 0.04034 | -0.76818 | down |
| WP_011240897.1 | dihydroneopterin aldolase | ZMO_RS04735*- | 2.2E+07 | 2E+07 | 2E+07 | 3.8E+07 | 3.5E+07 | 3.3E+07 | 0.57986 | 0.00477 | -0.78621 | down |
| WP_080502949.1 | 50S ribosomal protein L34 | ZMO_RS09190*rpmH | 6.5E+08 | 8.3E+08 | 8.8E+08 | 1.2E+09 | 1.6E+09 | 1.1E+09 | 0.60134 | 0.04487 | -0.73375 | down |
| WP_011240318.1 | EAL domain-containing protein | ZMO_RS01720*- | 0 | 0 | 0 | 1266828 | 1506915 | 3291417 | #NAME? | 0 | #NAME? | down |
| WP_011240873.1 | hypothetical protein | ZMO_RS04600*- | 2.1E+07 | 2219007 | 4236743 | 3.9E+07 | 4.2E+07 | 3.4E+07 | 0.23911 | 0.02586 | -2.06422 | down |
| WP_011241581.1 | flavodoxin FldA | ZMO_RS08330*fldA | 1.4E+07 | 1.3E+07 | 1.1E+07 | 2340877 | 2225111 | 1823894 | 5.90866 | 0.00257 | 2.56283 | up |
| WP_011241179.1 | N-formylglutamate amidohydrolase | ZMO_RS06235*- | 1478207 | 2014475 | 1277517 | 3957988 | 0 | 4277508 | 0.38615 | 0.00264 | -1.37277 | down |
| WP_011241332.1 | cytochrome d ubiquinol oxidase subunit II | ZMO_RS07030*cydB | 8.7E+07 | 9.3E+07 | 1.2E+08 | 4.6E+07 | 6.7E+07 | 6.1E+07 | 1.71278 | 0.03283 | 0.77634 | up |
| WP_011240953.1 | Fis family transcriptional regulator | ZMO_RS05030*- | 3363581 | 3338708 | 5184573 | 9826854 | 1.2E+07 | 1.1E+07 | 0.36263 | 0.00113 | -1.46344 | down |
| WP_011240081.1 | hypothetical protein | ZMO_RS00470*- | 5763053 | 9497116 | 9346311 | 3.3E+07 | 3.5E+07 | 4.4E+07 | 0.21868 | 0.00833 | -2.19307 | down |
| WP_011241383.1 | hypothetical protein | ZMO_RS07325*- | 9208222 | 3507983 | 3591172 | 1.2E+07 | 1.6E+07 | 1.4E+07 | 0.38181 | 0.02298 | -1.38907 | down |
| WP_011240539.1 | flagellar biosynthetic protein FliO | ZMO_RS02860*fliO | 2874266 | 1318110 | 2931897 | 4891718 | 8318587 | 6244941 | 0.36619 | 0.03482 | -1.44934 | down |
| WP_011240698.1 | phospho-N-acetylmuramoyl-pentapeptide-transferase | ZMO_RS03700*- | 3523358 | 1.4E+07 | 9612037 | 2.6E+07 | 2.4E+07 | 3.1E+07 | 0.33848 | 0.01164 | -1.56285 | down |
| WP_011240822.1 | NCS2 family permease | ZMO_RS04335*- | 1.1E+07 | 1.3E+07 | 8960018 | 4.1E+07 | 5.6E+07 | 3.8E+07 | 0.25055 | 0.01936 | -1.99683 | down |
| WP_011240493.1 | CvpA family protein | ZMO_RS02625*- | 5948166 | 4565414 | 6219114 | 1.2E+07 | 1.1E+07 | 1.6E+07 | 0.42522 | 0.03448 | -1.2337 | down |
| WP_011241579.1 | hypothetical protein | ZMO_RS08320*- | 0 | 0 | 0 | 1122399 | 1349248 | 1460409 | #NAME? | 0 | #NAME? | down |
| WP_011241097.1 | hypothetical protein | ZMO_RS05795*- | 1.4E+07 | 9620618 | 1.2E+07 | 1.7E+07 | 2E+07 | 2E+07 | 0.62929 | 0.01514 | -0.6682 | down |
| WP_011240468.1 | pyridoxal phosphate-dependent aminotransferase | ZMO_RS02495*- | 0 | 3381772 | 2391955 | 1.3E+07 | 1.1E+07 | 1.5E+07 | 0.21696 | 0.00536 | -2.20449 | down |

**Table S10** Differentially Expressed proteins of ZM532 strain in rich media and media with acetic + furfural treatments

| Protein | Description | Gene | ZM532_AF_1 | ZM532_AF_2 | ZM532_AF_3 | ZM532_RM_1 | ZM532_RM_2 | ZM532_RM_3 | ZM532_AF.vs.ZM532_RM FC | ZM532_AF.vs.ZM532_RM Pvalue | ZM532_AF.vs.ZM532_RM log2FC | ZM532_AF.vs.ZM532_RM UP.DOWN |
| --- | --- | --- | --- | --- | --- | --- | --- | --- | --- | --- | --- | --- |
| WP_011241206.1 | ATP-dependent chaperone ClpB | ZMO_RS06375*clpB | 10853105763 | 9190254193 | 9589944445 | 5.161E+09 | 5.302E+09 | 4.156E+09 | 2.02701523 | 0.0018747 | 1.019356928 | up |
| WP_011240321.1 | ATP-dependent Clp protease ATP-binding subunit ClpA | ZMO_RS01740*clpA | 1219394731 | 1328214905 | 1567663911 | 930679649 | 872543224 | 933164186 | 1.503907692 | 0.0423018 | 0.588716019 | up |
| WP_011241442.1 | TonB-dependent receptor | ZMO_RS07620*- | 928140415.7 | 1353788775 | 1202378643 | 1.886E+09 | 1.919E+09 | 2.017E+09 | 0.598530181 | 0.0173308 | -0.740504098 | down |
| WP_011240872.1 | ribonucleoside triphosphate reductase | ZMO_RS04595*- | 486559142.3 | 908685404 | 926639089 | 2.215E+09 | 2.159E+09 | 2.199E+09 | 0.353250169 | 0.0094283 | -1.501237843 | down |
| WP_011240982.1 | elongation factor Ts | ZMO_RS05175*- | 11418027421 | 1.0559E+10 | 1.0875E+10 | 6.98E+09 | 7.335E+09 | 6.078E+09 | 1.610992232 | 0.0014233 | 0.687949538 | up |
| WP_011240133.1 | phosphoglycerate kinase | ZMO_RS00760*pgk | 51370767484 | 3.8773E+10 | 3.7421E+10 | 2.378E+10 | 2.318E+10 | 1.825E+10 | 1.956131722 | 0.0296863 | 0.968003522 | up |
| WP_011240940.1 | hypothetical protein | ZMO_RS04955*- | 7523045359 | 6038437500 | 6453519724 | 4.026E+09 | 3.698E+09 | 3.505E+09 | 1.782466213 | 0.0143203 | 0.83387473 | up |
| WP_011240131.1 | transketolase | ZMO_RS00750*tkt | 734117958.9 | 1112414662 | 1055150653 | 2.11E+09 | 1.841E+09 | 2.139E+09 | 0.476477755 | 0.002542 | -1.069519235 | down |
| WP_011241246.1 | sulfurtransferase | ZMO_RS06540*- | 191245698.9 | 756255084 | 793247216 | 1.538E+09 | 1.491E+09 | 1.657E+09 | 0.371488704 | 0.0308161 | -1.428609753 | down |
| WP_011240263.1 | phosphomannomutase/phosphoglucomutase | ZMO_RS01445*- | 5995914639 | 5112036500 | 5699592259 | 3.261E+09 | 3.084E+09 | 2.617E+09 | 1.87525776 | 0.0017822 | 0.907088912 | up |
| WP_011240174.1 | DNA polymerase I | ZMO_RS00975*polA | 321875187.5 | 426585344 | 431207764 | 787296803 | 609731761 | 781951146 | 0.541385626 | 0.0130621 | -0.88527151 | down |
| WP_011241659.1 | molecular chaperone GroEL | ZMO_RS08760*-GroEL | 270403299 | 203722530 | 269066723 | 165856744 | 169375107 | 141991152 | 1.557327597 | 0.0422136 | 0.639072459 | up |
| WP_011240946.1 | NAD(P)-dependent oxidoreductase | ZMO_RS04990*- | 676674532.2 | 647196739 | 845984369 | 1.138E+09 | 1.093E+09 | 1.106E+09 | 0.650314248 | 0.0205233 | -0.620791063 | down |
| WP_011240404.1 | PBP1A family penicillin-binding protein | ZMO_RS02165*- | 304793291.6 | 508644797 | 553817973 | 811569429 | 727007294 | 864420307 | 0.568979506 | 0.0278234 | -0.813551407 | down |
| WP_011241052.1 | alcohol dehydrogenase AdhP | ZMO_RS05560*adhP | 7701975580 | 8248784018 | 9303877265 | 4.252E+09 | 4.337E+09 | 3.001E+09 | 2.178847595 | 0.0020972 | 1.123565287 | up |
| WP_011241465.1 | fructokinase | ZMO_RS07740*- | 6231308222 | 6533764940 | 5748566663 | 4.365E+09 | 4.114E+09 | 3.78E+09 | 1.510164349 | 0.0025191 | 0.594705565 | up |
| WP_012817395.1 | phosphoribosylamine--glycine ligase | ZMO_RS01290*purD | 836395334.3 | 1175435680 | 1071276411 | 653281996 | 638016843 | 552871682 | 1.671812552 | 0.0439747 | 0.741413098 | up |
| WP_011240740.1 | adenosyl-hopene transferase HpnH | ZMO_RS03910*hpnH | 1709034709 | 2218595302 | 2170718595 | 3.247E+09 | 3.202E+09 | 3.204E+09 | 0.631771384 | 0.0176367 | -0.662525503 | down |
| WP_011241294.1 | TonB-dependent receptor | ZMO_RS06825*- | 109008148.2 | 111814175 | 135342825 | 294752734 | 277809948 | 391807692 | 0.369324025 | 0.0243401 | -1.437040979 | down |
| WP_011241321.1 | glutamine--fructose-6-phosphate aminotransferase | ZMO_RS00235*- | 36724809.69 | 33743698.2 | 22979148.5 | 15058956 | 11626355 | 14538427 | 2.266840933 | 0.0455784 | 1.180683159 | up |
| WP_011241495.1 | NAD-dependent succinate-semialdehyde dehydrogenase | ZMO_RS07890*- | 1974174905 | 1424150287 | 1707129391 | 711937970 | 879388506 | 684483103 | 2.243357542 | 0.0170941 | 1.165659573 | up |
| WP_026059405.1 | hopanoid biosynthesis associated radical SAM protein HpnJ | ZMO_RS04355*hpnJ | 770351565.4 | 2138966720 | 1812394805 | 3.822E+09 | 3.676E+09 | 3.711E+09 | 0.421208153 | 0.0332806 | -1.247394735 | down |
| WP_011241007.1 | electron transfer flavoprotein-ubiquinone oxidoreductase | ZMO_RS05305*- | 271226561.2 | 354227804 | 429465464 | 610131799 | 634428255 | 554486899 | 0.586377041 | 0.0170078 | -0.770099477 | down |
| WP_011241054.1 | 2,3-diphosphoglycerate-dependent phosphoglycerate mutase | ZMO_RS05570*gpmA | 24045075836 | 1.9599E+10 | 1.9463E+10 | 1.525E+10 | 1.426E+10 | 1.177E+10 | 1.528653001 | 0.0206713 | 0.612260957 | up |
| WP_011241080.1 | threonine ammonia-lyase | ZMO_RS05705*- | 752900424 | 676458327 | 842932601 | 1.367E+09 | 1.362E+09 | 1.315E+09 | 0.561964675 | 0.0032137 | -0.831448649 | down |
| WP_011240829.1 | aldo/keto reductase | ZMO_RS04375*- | 1220039074 | 1103608393 | 1175656157 | 584753429 | 570514838 | 348929766 | 2.326358332 | 0.0055523 | 1.218073334 | up |
| WP_011240557.1 | hypothetical protein | ZMO_RS02950*- | 39903132.69 | 74448795 | 77820317.4 | 176770697 | 166634715 | 160329384 | 0.381494879 | 0.0066947 | -1.390264403 | down |
| WP_011240512.1 | flagellar hook-basal body complex protein | ZMO_RS02720*- | 3035324153 | 2297146003 | 2063477484 | 1.122E+09 | 1.082E+09 | 1.147E+09 | 2.206786918 | 0.0435798 | 1.141947333 | up |
| WP_011241376.1 | membrane protein insertase YidC | ZMO_RS07290*yidC | 177598234.7 | 612383443 | 525788048 | 1.053E+09 | 995070990 | 1.095E+09 | 0.418632387 | 0.0391649 | -1.256244167 | down |
| WP_011240744.1 | chemotaxis-specific protein-glutamate methyltransferase CheB | ZMO_RS03930*cheB | 1291921805 | 1478278436 | 1526337152 | 937136199 | 906265761 | 634931191 | 1.733639963 | 0.0088125 | 0.793804315 | up |
| WP_011240963.1 | c-type cytochrome | ZMO_RS05080*- | 436918982.7 | 319797687 | 491768329 | 660030137 | 626178760 | 669418064 | 0.638406518 | 0.0362837 | -0.647452713 | down |
| WP_011240770.1 | polysaccharide export protein | ZMO_RS04070*- | 400921499 | 682049603 | 594930740 | 1.478E+09 | 1.311E+09 | 1.403E+09 | 0.400245882 | 0.002441 | -1.321041536 | down |
| WP_011241169.1 | toxic anion resistance protein | ZMO_RS06185*- | 944299928.9 | 695075199 | 819823641 | 527009001 | 476239221 | 454388938 | 1.687113114 | 0.0345662 | 0.754556704 | up |
| WP_011241530.1 | 3-phosphoshikimate 1-carboxyvinyltransferase | ZMO_RS08080*aroA | 1388871694 | 1829764351 | 1754842401 | 1.11E+09 | 1.101E+09 | 917086168 | 1.589565217 | 0.0296128 | 0.668632209 | up |
| WP_011241200.1 | DEAD/DEAH box helicase | ZMO_RS06345*- | 346305634.8 | 973359625 | 769986973 | 2.673E+09 | 2.715E+09 | 2.493E+09 | 0.265163661 | 0.0044149 | -1.915045019 | down |
| WP_011241478.1 | LysR family transcriptional regulator | ZMO_RS07805*- | 647725224.7 | 936633665 | 901850566 | 1.578E+09 | 1.65E+09 | 1.701E+09 | 0.504382723 | 0.0060909 | -0.987409235 | down |
| WP_011240046.1 | hypothetical protein | ZMO_RS00295*- | 125386954.8 | 152638167 | 160238843 | 310349705 | 262348524 | 309519056 | 0.496775535 | 0.0025616 | -1.009333968 | down |
| WP_011240274.1 | DUF4011 domain-containing protein | ZMO_RS01500*- | 59653443.18 | 96233074.2 | 87447329.4 | 126225668 | 110342441 | 144863226 | 0.637949283 | 0.0368457 | -0.64848636 | down |
| WP_011240073.1 | serine hydrolase | ZMO_RS00430*- | 288203062.3 | 349762166 | 339243695 | 594237170 | 573194022 | 578928929 | 0.559568963 | 0.0027285 | -0.837612151 | down |
| WP_011240082.1 | aminodeoxychorismate synthase component I | ZMO_RS00475*pabB | 100003284.5 | 182724068 | 179387106 | 291394390 | 289899419 | 248557314 | 0.556864292 | 0.0274815 | -0.844602308 | down |
| WP_011240547.1 | glycosyltransferase | ZMO_RS02900*- | 137214881.8 | 134148642 | 167901365 | 259534931 | 254048800 | 273943116 | 0.557777669 | 0.0021722 | -0.842237919 | down |
| WP_011240992.1 | CocE/NonD family hydrolase | ZMO_RS05230*- | 149627361.6 | 156960563 | 234565142 | 336254612 | 277435928 | 342022969 | 0.566229379 | 0.0177764 | -0.820541489 | down |
| WP_011240767.1 | LPS biosynthesis protein | ZMO_RS04055*- | 307539539.9 | 558205260 | 578286150 | 943372004 | 836866213 | 962198648 | 0.526550298 | 0.0237403 | -0.925356744 | down |
| WP_011240554.1 | molecular chaperone Dnak | ZMO_RS02930*dnak | 700266951.1 | 954314807 | 904948142 | 552734385 | 542790135 | 487054164 | 1.617316047 | 0.0448541 | 0.69360163 | up |
| WP_011240605.1 | lytic transglycosylase domain-containing protein | ZMO_RS03205*- | 64345984.42 | 109737663 | 161272904 | 284977933 | 281941440 | 267332248 | 0.401984896 | 0.0237436 | -1.314786799 | down |
| WP_011240424.1 | 50S ribosomal protein L4 | ZMO_RS02265*rplD | 12060869639 | 1.2526E+10 | 1.5543E+10 | 2.062E+10 | 2.115E+10 | 2.314E+10 | 0.618214466 | 0.0049081 | -0.693820682 | down |
| WP_011241211.1 | HlyD family secretion protein | ZMO_RS06400*- | 664182339.2 | 722131146 | 735725745 | 1.391E+09 | 1.303E+09 | 1.475E+09 | 0.509015301 | 0.001628 | -0.97421907 | down |
| WP_011241041.1 | ACP S-malonyltransferase | ZMO_RS05500*fabD | 566651085.3 | 695111725 | 488583767 | 327608531 | 360762351 | 246174045 | 1.872940003 | 0.0266343 | 0.905304685 | up |
| WP_011241112.1 | LPS-assembly protein LptD | ZMO_RS05870*lptD | 93164398.6 | 115116376 | 96065112.9 | 152329971 | 149064695 | 171950490 | 0.64296821 | 0.0047721 | -0.637180687 | down |
| WP_011240419.1 | 30S ribosomal protein S7 | ZMO_RS02240*rpsG | 8808196779 | 1.1802E+10 | 1.2364E+10 | 1.853E+10 | 1.946E+10 | 1.931E+10 | 0.575586444 | 0.0135257 | -0.796895479 | down |
| WP_011240438.1 | 50S ribosomal protein L6 | ZMO_RS02335*rplF | 5204281500 | 4437433130 | 6604857274 | 9.186E+09 | 9.184E+09 | 9.979E+09 | 0.573092117 | 0.0134806 | -0.803161043 | down |
| WP_011241372.1 | DNA mismatch repair endonuclease MutL | ZMO_RS01515*- | 82122484.53 | 73092128.9 | 68416983.7 | 50679983 | 44168271 | 50961464 | 1.533722171 | 0.0099337 | 0.617037167 | up |
| WP_011241681.1 | glycosyltransferase family 1 protein | ZMO_RS08935*- | 82619146.94 | 101289032 | 150047378 | 291931332 | 257303190 | 231423850 | 0.427787069 | 0.0053279 | -1.225035221 | down |
| WP_011240248.1 | SDR family oxidoreductase | ZMO_RS01360*- | 1468297763 | 1626524773 | 1612068095 | 859900937 | 954827029 | 773013627 | 1.818918336 | 0.0006379 | 0.863080772 | up |
| WP_011240703.1 | UDP-N-acetylmuramate dehydrogenase | ZMO_RS03725*murB | 662239250 | 841303163 | 922592840 | 627636069 | 542850164 | 430330365 | 1.515561031 | 0.0499371 | 0.59985195 | up |
| WP_011241454.1 | orotate phosphoribosyltransferase | ZMO_RS07680*- | 1823259013 | 1553333798 | 1586607330 | 786045834 | 789161788 | 691363927 | 2.189739011 | 0.0041672 | 1.130758929 | up |
| WP_011240960.1 | carbonic anhydrase | ZMO_RS05065*- | 661249170.2 | 669192104 | 825517555 | 388710949 | 410063031 | 360539257 | 1.859686202 | 0.019241 | 0.895059206 | up |
| WP_011240585.1 | YfdX family protein | ZMO_RS03095*- | 1621009310 | 1048668259 | 1760378283 | 475820475 | 374146048 | 254212140 | 4.012082466 | 0.0284615 | 2.00435126 | up |
| WP_011241363.1 | hypothetical protein | ZMO_RS07225*- | 3278244933 | 3087866052 | 3216018031 | 1.627E+09 | 1.652E+09 | 1.398E+09 | 2.048632392 | 0.0001664 | 1.03466113 | up |
| WP_011240444.1 | adenylate kinase | ZMO_RS02365*- | 1615820693 | 1495629890 | 1591422008 | 1.141E+09 | 903242636 | 666664484 | 1.734494393 | 0.0328986 | 0.794515177 | up |
| WP_011240355.1 | ribonucleotide-diphosphate reductase subunit beta | ZMO_RS01910*- | 215245108.2 | 280342553 | 295587982 | 665944031 | 607557917 | 600433890 | 0.422199964 | 0.0004213 | -1.244001639 | down |
| WP_011241274.1 | 1-(5-phosphoribosyl)-5 | ZMO_RS06730*hisA | 1520803878 | 1200633781 | 1540284880 | 669616302 | 521234300 | 380391282 | 2.712327481 | 0.0036954 | 1.439531377 | up |
| WP_011241188.1 | response regulator | ZMO_RS06280*- | 495765634.5 | 491975997 | 522681058 | 333922668 | 339984495 | 274268960 | 1.59297693 | 0.0049281 | 0.671725373 | up |
| WP_011241213.1 | FUSC family protein | ZMO_RS06410*- | 25897307.4 | 76664132.2 | 66741144.7 | 300280115 | 265034631 | 297227706 | 0.19628319 | 0.0004547 | -2.34899147 | down |
| WP_011241304.1 | hypothetical protein | ZMO_RS06880*- | 109149883.5 | 59328468.2 | 116270107 | 190574532 | 145134959 | 187552432 | 0.54417959 | 0.0280787 | -0.877845248 | down |
| WP_017466469.1 | hypothetical protein | ZMO_RS08625*- | 198855308 | 136776800 | 170603837 | 261918559 | 274927355 | 222712859 | 0.666486864 | 0.0246741 | -0.585351654 | down |
| WP_011240432.1 | 30S ribosomal protein S17 | ZMO_RS02305*rpsQ | 5580116368 | 4694901926 | 6650773677 | 9.567E+09 | 9.903E+09 | 9.944E+09 | 0.575421268 | 0.0148016 | -0.797309549 | down |
| WP_011240802.1 | galactose-1-epimerase | ZMO_RS03970*- | 587373300.8 | 534852666 | 671958375 | 407167888 | 304504408 | 209920641 | 1.946829527 | 0.0174523 | 0.961126561 | up |
| WP_011240695.1 | penicillin-binding protein 2 | ZMO_RS03685*- | 116783532.3 | 171728825 | 164004096 | 250673322 | 227084829 | 269532969 | 0.605542393 | 0.0121901 | -0.723700131 | down |
| WP_017466360.1 | adenosine deaminase | ZMO_RS02905*- | 187511324.8 | 245379511 | 210742436 | 342012978 | 379189370 | 341825967 | 0.605471428 | 0.0034747 | -0.723869213 | down |
| WP_011241415.1 | S41 family peptidase | ZMO_RS07490*- | 145137304.4 | 129842159 | 202260472 | 294273175 | 289482637 | 298012322 | 0.541230644 | 0.0244913 | -0.885684568 | down |
| WP_011241250.1 | TonB-dependent receptor | ZMO_RS06600*- | 45771722.91 | 41544646.2 | 54964409.2 | 90735475 | 69900969 | 102496805 | 0.540717599 | 0.0367193 | -0.887052782 | down |
| WP_011240102.1 | SEL1-like repeat protein | ZMO_RS00580*- | 32751762.3 | 17080122.8 | 33598758.9 | 95742761 | 105587460 | 84153290 | 0.292243302 | 0.0013027 | -1.774758134 | down |
| WP_011241612.1 | alkene reductase | ZMO_RS08545*- | 1440925128 | 1056137907 | 1410564895 | 453427563 | 439908974 | 313794696 | 3.237119399 | 0.0109826 | 1.694710579 | up |
| WP_011240093.1 | S1/P1 Nuclease | ZMO_RS00535*- | 314906481.4 | 200077940 | 315261860 | 525861970 | 485623914 | 509017351 | 0.54603388 | 0.0194366 | -0.872937626 | down |
| WP_011241498.1 | gluconokinase | ZMO_RS07905*- | 1288502998 | 1007160051 | 1026567119 | 789551362 | 625705362 | 511135125 | 1.724586911 | 0.019078 | 0.786250836 | up |
| WP_011240736.1 | presqualene diphosphate synthase HpnD | ZMO_RS03890*hpnD | 223992166.1 | 225660883 | 298041041 | 450461588 | 352919580 | 417997473 | 0.612172234 | 0.0145233 | -0.707990485 | down |
| WP_011240894.1 | bifunctional diguanylate cyclase/phosphodiesterase | ZMO_RS04720*- | 22277573.37 | 52221267.5 | 60475567.3 | 83442275 | 99878693 | 89714056 | 0.494348331 | 0.0427505 | -1.016400134 | down |
| WP_011240529.1 | flagellar M-ring protein FliF | ZMO_RS02810*fliF | 118631230 | 130330511 | 146908526 | 278600477 | 270993358 | 297156610 | 0.467517046 | 0.000188 | -1.096909126 | down |
| WP_011240747.1 | 30S ribosomal protein S9 | ZMO_RS03945*rpsI | 8095951809 | 1.1271E+10 | 1.0656E+10 | 1.878E+10 | 1.984E+10 | 1.956E+10 | 0.516001919 | 0.0061501 | -0.954551663 | down |
| WP_011241468.1 | S-(hydroxymethyl)glutathione dehydrogenase/class III alcohol dehydrogenase | ZMO_RS07755*- | 148931723.2 | 186131219 | 235592243 | 345609847 | 318992917 | 305502784 | 0.588240306 | 0.0194525 | -0.765522453 | down |
| WP_011241368.1 | transcription elongation factor GreA | ZMO_RS07250*greA | 2718662309 | 1856365900 | 2308870567 | 892909060 | 576399065 | 533142465 | 3.437737146 | 0.011578 | 1.781459239 | up |
| WP_011240356.1 | DUF459 domain-containing protein | ZMO_RS01915*- | 279477822.8 | 388097825 | 304655019 | 494398906 | 461527325 | 509155216 | 0.663601788 | 0.0240031 | -0.591610322 | down |
| WP_011241180.1 | hypothetical protein | ZMO_RS06240*- | 3242728.412 | 2920947.12 | 17380074.5 | 584882293 | 627836084 | 552795265 | 0.013335354 | 0.0008932 | -6.228600026 | down |
| WP_011240898.1 | superoxide dismutase | ZMO_RS04740*- | 1577203936 | 955006270 | 1865564595 | 170936169 | 166402246 | 235023700 | 7.683553261 | 0.0408233 | 2.94177364 | up |
| WP_011240748.1 | 50S ribosomal protein L13 | ZMO_RS03950*rplM | 1407695817 | 2093322935 | 2511951695 | 6.269E+09 | 6.579E+09 | 5.864E+09 | 0.321339118 | 0.0008621 | -1.637831477 | down |
| WP_011240879.1 | calcium-binding protein | ZMO_RS04630*- | 1418355265 | 854074914 | 1266451399 | 628707306 | 454862552 | 353374320 | 2.46278292 | 0.0357968 | 1.300289468 | up |
| WP_011241323.1 | RNA pyrophosphohydrolase | ZMO_RS06975*- | 482408158.7 | 378913279 | 518680168 | 333714129 | 200907231 | 201222048 | 1.875401196 | 0.0244386 | 0.907199258 | up |
| WP_011240623.1 | CsbD family protein | ZMO_RS03310*- | 3557329279 | 2158013652 | 3322829428 | 961192009 | 719863817 | 893522792 | 3.510544325 | 0.0350093 | 1.811694744 | up |
| WP_011240023.1 | ribosome-associated translation inhibitor RaiA | ZMO_RS00175*raiA | 626699982.6 | 679421222 | 790837874 | 223143876 | 210493571 | 170357867 | 3.471813492 | 0.0052453 | 1.795689447 | up |
| WP_011241494.1 | ferredoxin--NADP reductase | ZMO_RS07885*- | 683243059.7 | 687401254 | 657945259 | 289745317 | 252495181 | 170021273 | 2.848095539 | 0.0042248 | 1.509997542 | up |
| WP_011240842.1 | Hsp20 family protein | ZMO_RS04435*- | 2200837754 | 1507851176 | 2334181929 | 976907842 | 1.003E+09 | 860348719 | 2.12787454 | 0.0493939 | 1.089413092 | up |
| WP_011241192.1 | inositol monophosphatase | ZMO_RS06300*- | 449367486.2 | 464195643 | 579957473 | 323725147 | 231868239 | 240142367 | 1.876905236 | 0.0128127 | 0.908355811 | up |
| WP_011240418.1 | 30S ribosomal protein S12 | ZMO_RS02235*rpsL | 654238375.1 | 1868649198 | 1289449110 | 4.863E+09 | 5.073E+09 | 4.839E+09 | 0.258019128 | 0.0070407 | -1.954450071 | down |
| WP_011241543.1 | electron transport complex subunit RsxG | ZMO_RS08135*rsxG | 102024139.8 | 126663313 | 106222845 | 238184718 | 229362396 | 184316976 | 0.513773198 | 0.0125441 | -0.960796464 | down |
| WP_011240439.1 | 50S ribosomal protein L18 | ZMO_RS02340*rplR | 2470671130 | 2337994301 | 3323245836 | 5.275E+09 | 5.301E+09 | 5.314E+09 | 0.511730145 | 0.0138532 | -0.966544874 | down |
| WP_011241619.1 | adenosine kinase | ZMO_RS08590*- | 689097572.2 | 890807651 | 830690108 | 571783433 | 550167903 | 433063313 | 1.550207474 | 0.0215094 | 0.632461314 | up |
| WP_011241574.1 | oxidoreductase | ZMO_RS08295*- | 641974823.3 | 530910967 | 615467777 | 411270443 | 385308667 | 377154564 | 1.523645104 | 0.018522 | 0.607526902 | up |
| WP_011241590.1 | nitronate monooxygenase | ZMO_RS08400*- | 264705563.1 | 242539337 | 208907892 | 142611334 | 164387150 | 154298900 | 1.552475297 | 0.0229539 | 0.634570312 | up |
| WP_011241506.1 | zinc transporter ZntB | ZMO_RS07945*- | 69700139.26 | 94628525.3 | 84776728.2 | 149888035 | 110565309 | 143926596 | 0.616018175 | 0.0312941 | -0.698955179 | down |
| WP_011240720.1 | peptidylprolyl isomerase | ZMO_RS03810*- | 433618153 | 357217777 | 396098859 | 242269681 | 190825708 | 198544828 | 1.87913112 | 0.0033975 | 0.910065737 | up |
| WP_011240200.1 | response regulator transcription factor | ZMO_RS01115*- | 1814977999 | 1315751629 | 1441427902 | 567310590 | 476303142 | 340348320 | 3.303672613 | 0.0096864 | 1.724070726 | up |
| WP_011240964.1 | phosphoserine phosphatase SerB | ZMO_RS05085*serB | 4284728149 | 2774229171 | 3368674268 | 1.89E+09 | 1.558E+09 | 1.266E+09 | 2.212608445 | 0.0348052 | 1.145748166 | up |
| WP_011240504.1 | flagellar motor stator protein MotA | ZMO_RS02680*motA | 73569387.82 | 69700205.5 | 94628403.5 | 156491429 | 158909125 | 168264377 | 0.4918653 | 0.0030823 | -1.023664815 | down |
| WP_011241196.1 | MucR family transcriptional regulator | ZMO_RS06320*- | 1204232589 | 624599061 | 1200689414 | 2.581E+09 | 2.726E+09 | 2.444E+09 | 0.390880666 | 0.0069829 | -1.355199867 | down |
| WP_011241226.1 | glycine--tRNA ligase subunit alpha | ZMO_RS06475*- | 398488641.7 | 435486547 | 546083405 | 779865823 | 664853562 | 763926561 | 0.62484374 | 0.0093385 | -0.678432647 | down |
| WP_011240505.1 | flagellin | ZMO_RS02685*- | 337857493.2 | 235445984 | 260771045 | 129911884 | 145023513 | 141634649 | 2.002243155 | 0.0429916 | 1.001617188 | up |
| WP_011241006.1 | tetratricopeptide repeat protein | ZMO_RS05300*- | 32102572.19 | 46296696.1 | 46578151.9 | 71241552 | 77464987 | 83444990 | 0.538344162 | 0.0050079 | -0.893399315 | down |
| WP_011241499.1 | outer membrane protein assembly factor BamD | ZMO_RS07910*bamD | 326539752 | 401579530 | 445259431 | 602651923 | 579261753 | 656819104 | 0.638145317 | 0.0087884 | -0.648043106 | down |
| WP_011241009.1 | lytic transglycosylase domain-containing protein | ZMO_RS05315*- | 49385133.47 | 8978211.2 | 6346506.4 | 131428613 | 117552741 | 104515751 | 0.18305624 | 0.0080291 | -2.449641139 | down |
| WP_011240428.1 | 50S ribosomal protein L22 | ZMO_RS02285*rplV | 3823212508 | 3978721618 | 4402888933 | 7.144E+09 | 7.018E+09 | 7.275E+09 | 0.569327815 | 0.0008686 | -0.81266851 | down |
| WP_011241132.1 | polyisoprenoid-binding protein | ZMO_RS05970*- | 374301806.2 | 370171423 | 349515029 | 190936789 | 148392609 | 150149621 | 2.235005416 | 0.0008745 | 1.160278327 | up |
| WP_011240089.1 | replication-associated recombination protein A | ZMO_RS00510*- | 157950858.3 | 186532059 | 183488398 | 116429489 | 103336034 | 130175744 | 1.508742655 | 0.0080993 | 0.593346747 | up |
| WP_011240246.1 | cardiolipin synthase | ZMO_RS01350*cls | 44452332.45 | 59788256 | 43575542.8 | 89022944 | 64398735 | 94441386 | 0.596362075 | 0.0480582 | -0.74573958 | down |
| WP_011241082.1 | endolytic transglycosylase MltG | ZMO_RS05720*mltG | 26678359.06 | 50665078.4 | 59685097.1 | 78170751 | 86966746 | 90116077 | 0.536832971 | 0.0442048 | -0.897454813 | down |
| WP_011241453.1 | tetratricopeptide repeat protein | ZMO_RS07675*- | 271154709 | 204189140 | 269426136 | 166006688 | 169659324 | 142183705 | 1.558586223 | 0.0420095 | 0.640237969 | up |
| WP_011241039.1 | hypothetical protein | ZMO_RS05490*- | 55035525.35 | 123500067 | 104283483 | 160206049 | 179901201 | 186739446 | 0.536814745 | 0.0431858 | -0.897503795 | down |
| WP_011241300.1 | KpsF/GutQ family sugar-phosphate isomerase | ZMO_RS06855*- | 48548306.2 | 94053772.7 | 87038306.1 | 155519000 | 163756534 | 140313938 | 0.499664154 | 0.0179419 | -1.000969372 | down |
| WP_011241647.1 | capsular biosynthesis protein | ZMO_RS08730*- | 6830848.579 | 55370863.8 | 60323862.3 | 172736982 | 130733649 | 145585250 | 0.272851509 | 0.0085661 | -1.873812074 | down |
| WP_011241253.1 | 6-phosphogluconolactonase | ZMO_RS06615*pgl | 4876131368 | 4499452693 | 4909146287 | 2.306E+09 | 1.946E+09 | 1.538E+09 | 2.466998847 | 0.0011188 | 1.302757042 | up |
| WP_011240217.1 | organic solvent tolerance protein OstA | ZMO_RS01205*- | 287216085.9 | 285087703 | 212051317 | 157952364 | 155872586 | 144408283 | 1.711694066 | 0.0444877 | 0.775424869 | up |
| WP_011240718.1 | pyridoxamine 5\-phosphate oxidase | ZMO_RS03800*pdxH | 1036145042 | 898624982 | 970576524 | 626452635 | 614511328 | 542090254 | 1.629421315 | 0.002494 | 0.704359686 | up |
| WP_011240354.1 | HAD family phosphatase | ZMO_RS01905*- | 306986240.6 | 302228141 | 333732879 | 192205301 | 176329512 | 150177079 | 1.817863205 | 0.0010409 | 0.86224364 | up |
| WP_011241194.1 | bacterioferritin | ZMO_RS06310*bfr | 2331706078 | 2087681059 | 2320954486 | 1.155E+09 | 992258739 | 1.047E+09 | 2.110338728 | 0.0006656 | 1.077474583 | up |
| WP_011241118.1 | hypothetical protein | ZMO_RS05900*- | 56572214.37 | 58434088.6 | 60208683.6 | 140281514 | 116319549 | 127968555 | 0.455613179 | 0.0085102 | -1.134118616 | down |
| WP_011240728.1 | YbaB/EbfC family nucleoid-associated protein | ZMO_RS03850*- | 1709915827 | 1584002200 | 1244477470 | 715276898 | 643572921 | 527463684 | 2.405960351 | 0.0141682 | 1.266612868 | up |
| WP_011240461.1 | DUF448 domain-containing protein | ZMO_RS02460*- | 412495722.1 | 445719814 | 704675809 | 980300427 | 742029126 | 1.001E+09 | 0.573897001 | 0.0362842 | -0.80113626 | down |
| WP_011240279.1 | rod shape-determining protein MreC | ZMO_RS01525*mreC | 52469154.18 | 64182436.7 | 80154861 | 101337740 | 100194183 | 105350244 | 0.641309508 | 0.0403057 | -0.640907297 | down |
| WP_011240427.1 | 30S ribosomal protein S19 | ZMO_RS02280*rpsS | 1696124977 | 1610036789 | 1876353103 | 4.516E+09 | 4.68E+09 | 4.954E+09 | 0.366259698 | 0.0001432 | -1.449061136 | down |
| WP_011240409.1 | RNA pseudouridine synthase | ZMO_RS02190*- | 45852890.9 | 59407079.8 | 83530989.8 | 130646199 | 140593296 | 125943253 | 0.475325178 | 0.0145321 | -1.073013271 | down |
| WP_011240875.1 | ABC transporter ATP-binding protein | ZMO_RS04610*- | 44361626.9 | 95847065 | 118822756 | 191580524 | 169865616 | 204082361 | 0.458034293 | 0.0279303 | -1.126472479 | down |
| WP_011240204.1 | hypothetical protein | ZMO_RS01135*- | 49857493.87 | 53660689.7 | 59477212.4 | 28545502 | 39216660 | 34845213 | 1.588534916 | 0.0087238 | 0.667696801 | up |
| WP_011241531.1 | (d)CMP kinase | ZMO_RS08085*- | 610825211.4 | 660190965 | 697313905 | 332199596 | 245496592 | 163827221 | 2.65444092 | 0.0049976 | 1.408408031 | up |
| WP_011241447.1 | exodeoxyribonuclease III | ZMO_RS07645*xth | 21215444.86 | 42954961.7 | 39195436.5 | 73280495 | 68479412 | 73104728 | 0.481074247 | 0.0259506 | -1.055668523 | down |
| WP_011240980.1 | ribosome recycling factor | ZMO_RS05165*- | 3617813697 | 2786521411 | 2869874120 | 1.387E+09 | 1.029E+09 | 875596644 | 2.817567029 | 0.0060122 | 1.494449932 | up |
| WP_011240240.1 | glycosyltransferase family 4 protein | ZMO_RS01320*- | 44540351.28 | 62559673.4 | 76576760.7 | 127811971 | 115890663 | 116073806 | 0.510530332 | 0.0133983 | -0.969931417 | down |
| WP_011240907.1 | signal recognition particle-docking protein FtsY | ZMO_RS04790*ftsY | 440271873.6 | 412840732 | 490585175 | 270219042 | 226810456 | 196128065 | 1.938517088 | 0.0023086 | 0.954953452 | up |
| WP_011240357.1 | SGNH/GDSL hydrolase family protein | ZMO_RS01920*- | 22148363.44 | 12494318 | 25066527.1 | 79435669 | 62252094 | 60601439 | 0.295167552 | 0.0046953 | -1.760393962 | down |
| WP_011240639.1 | hydroxyacylglutathione hydrolase | ZMO_RS03395*gloB | 386588929.9 | 334461132 | 339910118 | 144237993 | 132601606 | 111062663 | 2.735122434 | 0.0009366 | 1.451605415 | up |
| WP_011240109.1 | DUF2141 domain-containing protein | ZMO_RS00620*- | 228656611.4 | 228052494 | 256111936 | 148658548 | 149308536 | 136986465 | 1.638844062 | 0.0039817 | 0.712678586 | up |
| WP_011240854.1 | site-specific DNA-methyltransferase | ZMO_RS04495*- | 4913885.465 | 16233081.9 | 32769983.6 | 55960261 | 46201746 | 69388337 | 0.314292295 | 0.0215512 | -1.66982119 | down |
| WP_011240281.1 | penicillin-binding protein 2 | ZMO_RS01535*mrdA | 10772958.99 | 30938860.5 | 22183749.8 | 44567490 | 43505650 | 49119684 | 0.465735506 | 0.0433242 | -1.102417224 | down |
| WP_011240346.1 | arginase family protein | ZMO_RS03765*- | 369957689.5 | 277922292 | 353302361 | 257674881 | 204669664 | 187960925 | 1.539557006 | 0.0332328 | 0.622515288 | up |
| WP_011241000.1 | response regulator transcription factor | ZMO_RS05270*- | 134386852.6 | 135471739 | 138621292 | 90524912 | 81941991 | 64225193 | 1.725785906 | 0.0158005 | 0.787253501 | up |
| WP_011240446.1 | 30S ribosomal protein S11 | ZMO_RS02375*rpsK | 2518030774 | 1229075595 | 2609375621 | 5.88E+09 | 6.095E+09 | 5.989E+09 | 0.353848086 | 0.0117905 | -1.498797979 | down |
| WP_011241538.1 | HAD hydrolase-like protein | ZMO_RS08110*- | 240483895.2 | 290432148 | 242888404 | 142702741 | 134294876 | 73385948 | 2.208449606 | 0.0081532 | 1.143033912 | up |
| WP_011241464.1 | hypothetical protein | ZMO_RS07735*- | 283065071.8 | 361107570 | 372327236 | 222168620 | 161499231 | 148703391 | 1.909381644 | 0.0121956 | 0.933105495 | up |
| WP_011240052.1 | response regulator | ZMO_RS00325*- | 129031732 | 103097698 | 110209456 | 90865942 | 65626036 | 59140607 | 1.587602759 | 0.0291273 | 0.666849975 | up |
| WP_011241457.1 | signal peptidase I | ZMO_RS07695*lepB | 119860285 | 147891647 | 156836858 | 256667001 | 260202271 | 236704461 | 0.563433639 | 0.0021822 | -0.827682395 | down |
| WP_011241349.1 | ABC transporter permease | ZMO_RS07145*- | 35576833.82 | 99192455.1 | 92660221.7 | 171918579 | 142997691 | 177548198 | 0.461819126 | 0.0299282 | -1.114600172 | down |
| WP_011240672.1 | TolC family protein | ZMO_RS03555*- | 30223060.25 | 45286593.8 | 45155510.7 | 57924729 | 70765372 | 59373452 | 0.641619084 | 0.0268964 | -0.640211042 | down |
| WP_011240584.1 | organic hydroperoxide resistance protein | ZMO_RS03090*- | 3800760447 | 2360741336 | 2974656016 | 1.133E+09 | 927861650 | 698400044 | 3.310926472 | 0.0281232 | 1.727234972 | up |
| WP_011240113.1 | YebC/PmpR family DNA-binding transcriptional regulator | ZMO_RS00645*- | 2805394527 | 2243570354 | 2981096411 | 1.85E+09 | 1.681E+09 | 1.28E+09 | 1.668924784 | 0.020984 | 0.738918936 | up |
| WP_011241103.1 | hypothetical protein | ZMO_RS05825*- | 44276747.72 | 64524708.6 | 71299947.6 | 145127697 | 149051983 | 127456452 | 0.427148886 | 0.0018072 | -1.227189074 | down |
| WP_011240521.1 | flagellar biosynthesis protein FlhA | ZMO_RS02770*flhA | 30431063.54 | 48750393.5 | 29823803.8 | 89670285 | 72121122 | 67980886 | 0.474405593 | 0.0115865 | -1.075807076 | down |
| WP_011240194.1 | RidA family protein | ZMO_RS01085*- | 1606075051 | 1209170355 | 1342222185 | 942634498 | 905956009 | 742059708 | 1.604796961 | 0.028136 | 0.682390779 | up |
| WP_017466353.1 | hypothetical protein | ZMO_RS02650*- | 11367999.91 | 19354296.5 | 21323891.2 | 63620196 | 59459183 | 67841504 | 0.272606048 | 0.0003763 | -1.875110523 | down |
| WP_011241394.1 | ATP-dependent DNA helicase RecG | ZMO_RS07380*recG | 16585251.24 | 37932717.7 | 49484612.3 | 237292211 | 238924615 | 276907650 | 0.13809481 | 0.0002868 | -2.856269 | down |
| WP_011240238.1 | ferrochelatase | ZMO_RS01310*- | 27066892.38 | 18747808.5 | 27064719.1 | 116321208 | 129689550 | 90415713 | 0.216628079 | 0.0130068 | -2.206707842 | down |
| WP_011240823.1 | purine nucleoside permease | ZMO_RS04340*- | 40484281.44 | 33767741.8 | 48591230.6 | 123846659 | 107352419 | 138698385 | 0.332100828 | 0.0045224 | -1.590306776 | down |
| WP_011240925.1 | dTMP kinase | ZMO_RS04875*- | 200509157.6 | 202871252 | 225057851 | 131928755 | 127485189 | 127008555 | 1.626298321 | 0.0073895 | 0.701591923 | up |
| WP_011241010.1 | bifunctional DNA-formamidopyrimidine glycosylase/DNA-(apurinic or apyrimidinic site) lyase | ZMO_RS05320*mutM | 22950895.89 | 31578591.7 | 24377123.7 | 46848077 | 43305038 | 54713343 | 0.54468517 | 0.0079316 | -0.876505507 | down |
| WP_011240188.1 | hypothetical protein | ZMO_RS01055*- | 38993964.86 | 52055227.8 | 36457301.4 | 99519117 | 122880882 | 107259775 | 0.386782083 | 0.0020411 | -1.37040713 | down |
| WP_011241422.1 | aldo/keto reductase | ZMO_RS07520*- | 505648245.3 | 491435266 | 589706905 | 344684244 | 290811347 | 220183952 | 1.854421354 | 0.0072254 | 0.890969084 | up |
| WP_011240861.1 | hypothetical protein | ZMO_RS04535*- | 17979310.72 | 17786792.2 | 59861584.4 | 142488092 | 146166308 | 154817670 | 0.21563407 | 0.0103584 | -2.213342956 | down |
| WP_011240839.1 | RNA polymerase-binding protein DksA | ZMO_RS04425*dksA | 1138406815 | 853596503 | 823736836 | 515264309 | 471908640 | 386222689 | 2.050203217 | 0.0287227 | 1.035766917 | up |
| WP_011240622.1 | monooxygenase | ZMO_RS03305*- | 628437675 | 411620202 | 579968899 | 376214170 | 269481946 | 184609697 | 1.951120601 | 0.0389861 | 0.964302956 | up |
| WP_011241385.1 | murein transglycosylase A | ZMO_RS07335*- | 8935675.059 | 9037863.15 | 9435272.71 | 68118846 | 52983974 | 78050320 | 0.137626808 | 0.0157894 | -2.861166583 | down |
| WP_011240972.1 | 50S ribosomal protein L31 | ZMO_RS05125*rpmE | 4391013212 | 3745139035 | 4570639011 | 8.187E+09 | 9.325E+09 | 9.724E+09 | 0.466549519 | 0.0023867 | -1.09989788 | down |
| WP_011241657.1 | hypothetical protein | ZMO_RS08785*- | 70950616.35 | 93804486.3 | 115325677 | 197874119 | 218642593 | 198336188 | 0.455524858 | 0.0042923 | -1.134398309 | down |
| WP_011240756.1 | NERD domain-containing protein | ZMO_RS03990*- | 330097.1541 | 6243123.09 | 9603822.39 | 40906452 | 31582204 | 47388417 | 0.134946927 | 0.0058247 | -2.889535969 | down |
| WP_011240257.1 | elongation factor P | ZMO_RS01410*efp | 1539119176 | 1182612320 | 1323270995 | 708870627 | 630141138 | 448299590 | 2.263177303 | 0.0055177 | 1.178349613 | up |
| WP_011240734.1 | glycosyltransferase | ZMO_RS03880*- | 26193401.23 | 29792086 | 42231802.9 | 107183399 | 79959292 | 115040400 | 0.325025765 | 0.0121999 | -1.621374009 | down |
| WP_011241089.1 | glycosyltransferase family 4 protein | ZMO_RS05755*- | 38397813.14 | 74554740.8 | 76278443.2 | 328491501 | 274111591 | 381182154 | 0.192349903 | 0.0066172 | -2.378194991 | down |
| WP_011240550.1 | arginine N-succinyltransferase | ZMO_RS02915*- | 61142977.62 | 57110344 | 60474048.5 | 100407965 | 94647285 | 92206964 | 0.622175006 | 0.0009548 | -0.684607655 | down |
| WP_011240060.1 | hypothetical protein | ZMO_RS00365*- | 20214003.52 | 45433455.8 | 43427382.3 | 102136573 | 83154032 | 97608042 | 0.385561553 | 0.005891 | -1.374966898 | down |
| WP_011240241.1 | capsular polysaccharide biosynthesis protein | ZMO_RS01325*- | 9744996.565 | 20056926.1 | 30441215.1 | 44583909 | 37148851 | 49074703 | 0.460548168 | 0.0381326 | -1.118576042 | down |
| WP_011240603.1 | RNA degradosome polyphosphate kinase | ZMO_RS03175*- | 45894050.41 | 37925317.5 | 39697394.4 | 19466356 | 13897575 | 27132707 | 2.041712843 | 0.0145898 | 1.029779973 | up |
| WP_011240634.1 | glutaredoxin 3 | ZMO_RS03370*grxC | 1029399130 | 595460803 | 842175076 | 346468907 | 246921398 | 220805344 | 3.030027255 | 0.0387765 | 1.599330771 | up |
| WP_011240171.1 | methionine biosynthesis protein MetW | ZMO_RS00960*metW | 34623709.27 | 39967718.3 | 46509890.1 | 80280142 | 76763311 | 77738883 | 0.515802506 | 0.0048055 | -0.955109313 | down |
| WP_011241366.1 | ABC transporter substrate-binding protein | ZMO_RS07240*- | 316514719.2 | 244843899 | 296301882 | 155018102 | 132774548 | 98703479 | 2.219066212 | 0.0052305 | 1.149952715 | up |
| WP_011240144.1 | DsbA family protein | ZMO_RS00820*- | 119435954.1 | 91361151.4 | 118804343 | 248372224 | 226638924 | 234271009 | 0.464697224 | 0.0006671 | -1.105637069 | down |
| WP_011241370.1 | GatB/YqeY domain-containing protein | ZMO_RS07265*- | 1761224129 | 1365834234 | 1216811731 | 711683224 | 586405885 | 421682671 | 2.525841011 | 0.0173991 | 1.336763832 | up |
| WP_011240552.1 | HAD-IB family hydrolase | ZMO_RS02925*- | 13003820.39 | 58495455.7 | 54114969 | 126719417 | 98185364 | 92777167 | 0.395408823 | 0.0271093 | -1.338583033 | down |
| WP_011241685.1 | DUF2336 domain-containing protein | ZMO_RS08955*- | 12169447.37 | 28365468.5 | 39584808.8 | 56878874 | 60434453 | 89019509 | 0.388303316 | 0.0338209 | -1.364744067 | down |
| WP_011240213.1 | apolipoprotein N-acyltransferase | ZMO_RS01180*lnt | 82089817.71 | 88247375.1 | 111196273 | 183599624 | 134141372 | 191565639 | 0.552777927 | 0.0337799 | -0.855228085 | down |
| WP_011241407.1 | tRNA lysidine(34) synthetase TilS | ZMO_RS07450*tilS | 67541241.25 | 60176435.9 | 65925538.1 | 110638354 | 86960787 | 116674260 | 0.616161644 | 0.040587 | -0.698619217 | down |
| WP_011240230.1 | 50S ribosomal protein L28 | ZMO_RS01270*rpmB | 745400827.2 | 923072313 | 1085184219 | 2.779E+09 | 2.61E+09 | 3.832E+09 | 0.298618669 | 0.0241826 | -1.74362373 | down |
| WP_011241133.1 | NAD(P)H:quinone oxidoreductase | ZMO_RS05975*wrbA | 915538906.9 | 940779538 | 712177020 | 523122728 | 517866740 | 474640261 | 1.694672133 | 0.0351452 | 0.761006183 | up |
| WP_011240631.1 | RluA family pseudouridine synthase | ZMO_RS03355*- | 9485400.887 | 21945986.9 | 31047884.7 | 50292507 | 40984217 | 46514387 | 0.45343471 | 0.0406527 | -1.141033261 | down |
| WP_011240914.1 | 50S ribosomal protein L19 | ZMO_RS04825*rplS | 4300266863 | 6014630609 | 5695365080 | 7.818E+09 | 7.749E+09 | 9.088E+09 | 0.649355708 | 0.0145075 | -0.622919112 | down |
| WP_011240167.1 | ferredoxin family protein | ZMO_RS00940*- | 974599856.8 | 606160850 | 861802470 | 358490470 | 236261085 | 202790735 | 3.062612737 | 0.023503 | 1.614762952 | up |
| WP_011240508.1 | flagellar basal body P-ring protein FlgI | ZMO_RS02700*flgI | 15684274.89 | 15377904.5 | 16452107.3 | 97490774 | 82537633 | 104861390 | 0.166781286 | 0.0067156 | -2.583970676 | down |
| WP_011241692.1 | sel1 repeat family protein | ZMO_RS08990*- | 73369067.6 | 96133553.1 | 133887350 | 191317431 | 192651689 | 148400391 | 0.569886074 | 0.0304944 | -0.811254555 | down |
| WP_011240022.1 | PTS transporter subunit EIIA | ZMO_RS00170*- | 165756436.8 | 117099884 | 147372924 | 69777666 | 57880491 | 46367725 | 2.472214134 | 0.0137825 | 1.305803709 | up |
| WP_011241520.1 | mechanosensitive ion channel family protein | ZMO_RS08020*- | 47598140.03 | 67348905.7 | 101417038 | 172550124 | 183936526 | 195641691 | 0.391872809 | 0.0099468 | -1.351542624 | down |
| WP_011241159.1 | hypothetical protein | ZMO_RS06135*- | 23730761.34 | 19654110 | 10205741.3 | 0 | 0 | 0 | Inf | 0 | Inf | up |
| WP_011240526.1 | flagellin | ZMO_RS02795*- | 173004891.7 | 179471080 | 151129094 | 80208475 | 78093019 | 72920769 | 2.178012865 | 0.0061502 | 1.123012476 | up |
| WP_011241215.1 | hypothetical protein | ZMO_RS06420*- | 21190271.39 | 4481221.22 | 5369448.68 | 41412924 | 51176686 | 44432868 | 0.226539046 | 0.0100261 | -2.142168364 | down |
| WP_011240110.1 | tRNA (guanosine(46)-N7)-methyltransferase TrmB | ZMO_RS00625*trmB | 40799616.82 | 46583037.2 | 66275088.1 | 112911906 | 122967532 | 106845497 | 0.448341299 | 0.0043685 | -1.157330697 | down |
| WP_011241518.1 | antibiotic biosynthesis monooxygenase | ZMO_RS08010*- | 19246540578 | 1.5231E+10 | 1.5703E+10 | 6.731E+09 | 5.327E+09 | 5.007E+09 | 2.9403168 | 0.0060132 | 1.555971604 | up |
| WP_011241244.1 | alpha/beta hydrolase | ZMO_RS06570*- | 218106953.2 | 256401679 | 272194832 | 169840149 | 181380251 | 119456973 | 1.586444364 | 0.0219259 | 0.665796927 | up |
| WP_011240766.1 | glucans biosynthesis glucosyltransferase MdoH | ZMO_RS04045*mdoH | 20335717.67 | 42405360.2 | 47081436.7 | 106460600 | 78566617 | 106803102 | 0.376323183 | 0.0085841 | -1.409955926 | down |
| WP_011241292.1 | DUF2093 domain-containing protein | ZMO_RS06815*- | 527369804.6 | 188084169 | 725177841 | 1.272E+09 | 953808868 | 1.308E+09 | 0.407746201 | 0.0265333 | -1.294256658 | down |
| WP_011240916.1 | AAA family ATPase | ZMO_RS04835*- | 50705022.53 | 49363595.1 | 69856869.1 | 101302433 | 113881090 | 106605353 | 0.528065136 | 0.006046 | -0.921212199 | down |
| WP_011240751.1 | recombinase family protein | ZMO_RS03965*- | 43603651.49 | 34297078.8 | 44360674.1 | 77554864 | 64934145 | 74840345 | 0.562562776 | 0.0035319 | -0.829914 | down |
| WP_011240519.1 | flagellar biosynthesis anti-sigma factor FlgM | ZMO_RS02760*flgM | 129443024.7 | 111834132 | 129348160 | 76757497 | 66213897 | 71074556 | 1.731522214 | 0.0041663 | 0.792040897 | up |
| WP_011241001.1 | phosphoribosyl-AMP cyclohydrolase | ZMO_RS05275*hisI | 259085460.7 | 250018486 | 318109469 | 179526282 | 148614871 | 128967581 | 1.809664426 | 0.0119066 | 0.855722197 | up |
| WP_011240883.1 | NUDIX hydrolase | ZMO_RS04660*- | 58447658.14 | 58254632.7 | 65357701.1 | 37508040 | 29227183 | 28559009 | 1.910503795 | 0.0017232 | 0.933953123 | up |
| WP_017466250.1 | TonB-dependent receptor | ZMO_RS07005*- | 4143005.714 | 3590895.27 | 3946682.29 | 10472332 | 8691699.1 | 13101855 | 0.362010314 | 0.0314286 | -1.465897292 | down |
| WP_011241546.1 | RnfABCDGE type electron transport complex subunit B | ZMO_RS08150*- | 14369001.4 | 53862170.1 | 42687716 | 126774274 | 89485199 | 137163092 | 0.31384212 | 0.0134598 | -1.671889109 | down |
| WP_011240261.1 | AAA family ATPase | ZMO_RS01435*- | 350733380.1 | 255555899 | 337499364 | 207335597 | 138978402 | 137908344 | 1.949081155 | 0.0171904 | 0.962794162 | up |
| WP_011241008.1 | uracil-DNA glycosylase | ZMO_RS05310*- | 11055507.85 | 18540651.8 | 14120439 | 41527106 | 38945063 | 29939441 | 0.395942047 | 0.0095112 | -1.336638812 | down |
| WP_011241357.1 | TIGR01244 family phosphatase | ZMO_RS07195*- | 98634786.87 | 91656185.3 | 120905453 | 28394470 | 19319812 | 19557725 | 4.625942362 | 0.0066144 | 2.209747291 | up |
| WP_011240775.1 | heavy-metal-associated domain-containing protein | ZMO_RS04095*- | 92223631.28 | 73807248.1 | 99863526.7 | 46196000 | 28116221 | 26436951 | 2.639172119 | 0.0059204 | 1.400085442 | up |
| WP_011240607.1 | SsrA-binding protein SmpB | ZMO_RS03215*smpB | 47772526.42 | 86775153.9 | 58485145.3 | 141489030 | 130388595 | 147150888 | 0.460667519 | 0.0127101 | -1.118202216 | down |
| WP_011241079.1 | amidohydrolase family protein | ZMO_RS05700*- | 18573232.32 | 13846365.7 | 23929667.6 | 46228972 | 42311060 | 38982368 | 0.44187739 | 0.003785 | -1.17828198 | down |
| WP_011240812.1 | cytochrome c1 | ZMO_RS04285*- | 4706466.26 | 11172813.1 | 16107509.2 | 25552784 | 23554113 | 22770945 | 0.445015987 | 0.0491579 | -1.168070929 | down |
| WP_011241338.1 | glucose-6-phosphate isomerase | ZMO_RS05445*- | 53717158.16 | 47412972.9 | 72260119.1 | 28985070 | 22084866 | 27034048 | 2.219992405 | 0.0426937 | 1.150554741 | up |
| WP_011240164.1 | 5-formyltetrahydrofolate cyclo-ligase | ZMO_RS00925*- | 102710747.3 | 119246545 | 143084874 | 54671507 | 51047798 | 33632282 | 2.619576687 | 0.0100211 | 1.389333697 | up |
| WP_011240442.1 | 50S ribosomal protein L15 | ZMO_RS02355*- | 1618331169 | 3271053033 | 3087647171 | 4.516E+09 | 4.457E+09 | 5.124E+09 | 0.565869286 | 0.0446361 | -0.82145926 | down |
| WP_011241573.1 | FAD:protein FMN transferase | ZMO_RS08290*- | 27763119.97 | 30241180.2 | 28104926.2 | 71173735 | 68859940 | 55522512 | 0.440329849 | 0.0155465 | -1.183343449 | down |
| WP_011241467.1 | VOC family protein | ZMO_RS07750*- | 1177386778 | 992654789 | 689494917 | 413555402 | 239212104 | 152184154 | 3.552432554 | 0.022897 | 1.828807259 | up |
| WP_011241150.1 | chromosomal replication initiator protein DnaA | ZMO_RS06065*dnaA | 51719978.41 | 37174879.3 | 39564550.5 | 91111993 | 75818768 | 72768281 | 0.535919575 | 0.0078853 | -0.899911582 | down |
| WP_011241625.1 | 50S ribosomal protein L32 | ZMO_RS09205*rpmF | 378792285.2 | 17422474.7 | 189284040 | 653134647 | 657144146 | 581437021 | 0.309506743 | 0.0464905 | -1.691957255 | down |
| WP_014500625.1 | 50S ribosomal protein L21 | ZMO_RS00910*rplU | 655967237.4 | 873565284 | 869467770 | 1.387E+09 | 1.417E+09 | 1.826E+09 | 0.518214902 | 0.0189461 | -0.948377591 | down |
| WP_011240868.1 | type III PLP-dependent enzyme | ZMO_RS04575*- | 3668699.17 | 0 | 4444027.79 | 78899624 | 79070540 | 74706274 | 0.052300485 | 0.000164 | -4.257031878 | down |
| WP_011240096.1 | metallophosphoesterase | ZMO_RS00550*- | 4550764.125 | 13649558.7 | 13156277.4 | 53420949 | 45432316 | 46389198 | 0.215891411 | 0.0006916 | -2.211622244 | down |
| WP_011241199.1 | NifU family protein | ZMO_RS06335*- | 702259081.2 | 554111257 | 710329163 | 422486964 | 373008504 | 368349005 | 1.689830166 | 0.0244611 | 0.756878258 | up |
| WP_011240845.1 | mechanosensitive ion channel family protein | ZMO_RS04450*- | 10996857.04 | 18773660.9 | 15993489.1 | 39002363 | 34812785 | 34577074 | 0.422207482 | 0.0028914 | -1.243975951 | down |
| WP_011241174.1 | hypothetical protein | ZMO_RS06210*- | 10386130.77 | 14801598.2 | 1135626.03 | 32457493 | 44151620 | 32896381 | 0.240383875 | 0.007587 | -2.056587971 | down |
| WP_011240509.1 | flagellar basal body L-ring protein FlgH | ZMO_RS02705*- | 21763956.24 | 3892328.53 | 20236346.2 | 70259911 | 50124869 | 88787353 | 0.219401267 | 0.0228683 | -2.18835624 | down |
| WP_011241182.1 | sterol desaturase family protein | ZMO_RS06250*- | 27355828.04 | 29913125 | 45782135.3 | 102633352 | 69335046 | 84329836 | 0.402074906 | 0.0164493 | -1.314463798 | down |
| WP_011241709.1 | dienelactone hydrolase family protein | ZMO_RS09075*- | 24376821.21 | 22444115.6 | 31722438.7 | 9850708.6 | 12145497 | 4556783.2 | 2.957986279 | 0.0097789 | 1.56461536 | up |
| WP_011241084.1 | acyl carrier protein | ZMO_RS05730*- | 4492896910 | 2899428868 | 2807063668 | 1.479E+09 | 1.061E+09 | 1.04E+09 | 2.848894913 | 0.0485438 | 1.510402406 | up |
| WP_011240160.1 | 50S ribosomal protein L27 | ZMO_RS00905*rpmA | 295270519.7 | 539197873 | 452975057 | 3.177E+09 | 3.066E+09 | 2.876E+09 | 0.141185022 | 2.92E-05 | -2.824341053 | down |
| WP_011241306.1 | ferrous iron transport protein A | ZMO_RS06890*- | 50356388.29 | 45093490.9 | 45958689.1 | 35455010 | 18803676 | 23446514 | 1.819808302 | 0.0396719 | 0.863786485 | up |
| WP_011241127.1 | PTS sugar transporter subunit IIA | ZMO_RS05945*- | 78190927.68 | 101808779 | 90842868.1 | 53424058 | 50073850 | 41169754 | 1.872170816 | 0.0116146 | 0.904712071 | up |
| WP_011240522.1 | P-loop NTPase | ZMO_RS02775*- | 62767360.96 | 93581515.6 | 88986706.3 | 164847748 | 170588816 | 171150935 | 0.484290638 | 0.0095202 | -1.04605498 | down |
| WP_011240115.1 | Holliday junction branch migration protein RuvA | ZMO_RS00655*ruvA | 570082323.1 | 561226092 | 701243920 | 131912438 | 103504593 | 116494345 | 5.207425676 | 0.0068483 | 2.380570343 | up |
| WP_011240932.1 | thioredoxin | ZMO_RS04910*trxA | 966748973 | 778835537 | 1009873243 | 583878087 | 328776236 | 426836592 | 2.057093275 | 0.0101187 | 1.040607211 | up |
| WP_011241317.1 | transcription antitermination factor NusB | ZMO_RS06945*nusB | 1546061159 | 1404948775 | 1658983936 | 1.086E+09 | 1.025E+09 | 946576636 | 1.507671768 | 0.0077461 | 0.592322377 | up |
| WP_011240575.1 | type I-F CRISPR-associated protein Csy2 | ZMO_RS03040*csy2 | 19041189.36 | 30064759.5 | 24044954.9 | 66518578 | 63884150 | 53748549 | 0.397232672 | 0.0021102 | -1.331943807 | down |
| WP_011240989.1 | phosphate regulon transcriptional regulatory protein PhoB | ZMO_RS05215*phoB | 34798788.95 | 30802538.3 | 30099068.5 | 15378353 | 13123773 | 16106466 | 2.145335484 | 0.0014036 | 1.101203272 | up |
| WP_011240343.1 | iron-sulfur cluster assembly accessory protein | ZMO_RS01850*- | 89718171.16 | 76144021.9 | 93498387.7 | 47707122 | 14996984 | 36093617 | 2.625167588 | 0.0149708 | 1.392409526 | up |
| WP_011240863.1 | hypothetical protein | ZMO_RS04550*- | 0 | 0 | 0 | 12377128 | 9598486.3 | 13269515 | #NAME? | 0 | #NAME? | down |
| WP_011241271.1 | histidine triad nucleotide-binding protein | ZMO_RS06715*- | 380699371.4 | 448183522 | 365763111 | 260235820 | 229258615 | 227519096 | 1.666141506 | 0.0138546 | 0.736510935 | up |
| WP_011240742.1 | HpnM family protein | ZMO_RS03920*- | 986406550.6 | 862777007 | 935875555 | 417954273 | 376335192 | 236238425 | 2.702555786 | 0.0016999 | 1.434324398 | up |
| WP_011240479.1 | glutaredoxin, GrxB family | ZMO_RS02555*grxB | 291120332.4 | 124761255 | 225357128 | 527741986 | 361897562 | 651426153 | 0.416100828 | 0.0490246 | -1.264994934 | down |
| WP_011241603.1 | DUF1476 domain-containing protein | ZMO_RS08495*- | 123880178.6 | 112006474 | 112963835 | 37775813 | 20156927 | 23396614 | 4.28935519 | 0.0003341 | 2.100760787 | up |
| WP_011240545.1 | flagellar protein FliS | ZMO_RS02890*- | 156471280.7 | 173819456 | 139721701 | 99201537 | 93395747 | 77886880 | 1.737670811 | 0.007518 | 0.7971548 | up |
| WP_011240984.1 | aspartate/glutamate racemase family protein | ZMO_RS05190*- | 44653940.97 | 39986009.4 | 52981245.2 | 26721266 | 23492108 | 15253620 | 2.10214624 | 0.0095141 | 1.071863037 | up |
| WP_011240956.1 | hypothetical protein | ZMO_RS05045*- | 0 | 0 | 0 | 7967277.5 | 10115861 | 8990862.6 | #NAME? | 0 | #NAME? | down |
| WP_011240090.1 | hypothetical protein | ZMO_RS00515*- | 237012147.5 | 116325659 | 174445508 | 36185268 | 47412298 | 16055536 | 5.296205577 | 0.0469284 | 2.404959123 | up |
| WP_011240804.1 | glycosyltransferase | ZMO_RS04245*- | 15813637.26 | 34826983.4 | 37803748.6 | 93533507 | 86793208 | 67839807 | 0.356391218 | 0.0069352 | -1.488466307 | down |
| WP_011240577.1 | type I-F CRISPR-associated endoribonuclease Cas6/Csy4 | ZMO_RS03050*cas6f | 19631793.78 | 27694612.7 | 27915917.7 | 63760526 | 53324555 | 48894030 | 0.453324057 | 0.007418 | -1.141385371 | down |
| WP_011240443.1 | preprotein translocase subunit SecY | ZMO_RS02360*secY | 39988113.52 | 150335589 | 114434596 | 340139031 | 352339724 | 368432167 | 0.287260968 | 0.0122193 | -1.799566116 | down |
| WP_011240332.1 | cell division protein | ZMO_RS01795*- | 1013404.151 | 0 | 1237472.01 | 11862030 | 11597644 | 13360138 | 0.091698301 | 0.0017252 | -3.446961185 | down |
| WP_011241389.1 | murein biosynthesis integral membrane protein MurJ | ZMO_RS07355*murJ | 12269612.14 | 26548960.6 | 24073051.2 | 35591827 | 38149586 | 41518768 | 0.54564919 | 0.0438904 | -0.873954386 | down |
| WP_011241095.1 | hypothetical protein | ZMO_RS05785*- | 43876372.75 | 74588138.2 | 71905341.7 | 114906910 | 110997206 | 102888697 | 0.578996392 | 0.0308754 | -0.788373736 | down |
| WP_011240694.1 | hypothetical protein | ZMO_RS03680*- | 1535206.015 | 16786363.7 | 15132395.8 | 45385693 | 31876484 | 48132963 | 0.266788373 | 0.0117602 | -1.906232301 | down |
| WP_011240251.1 | bifunctional riboflavin kinase/FAD synthetase | ZMO_RS01380*- | 1965857240 | 1498703070 | 1467115537 | 1.027E+09 | 821198147 | 1.014E+09 | 1.722826016 | 0.0358183 | 0.784777015 | up |
| WP_011240245.1 | L-2-amino-thiazoline-4-carboxylic acid hydrolase | ZMO_RS01345*- | 14311284.65 | 15586498.8 | 15632693.5 | 5803971.1 | 3181021.9 | 5675558.9 | 3.105645494 | 0.0018137 | 1.634893157 | up |
| WP_011240348.1 | glycine zipper 2TM domain-containing protein | ZMO_RS01875*- | 109511910.5 | 90140019.9 | 142301662 | 330223580 | 415898508 | 377008142 | 0.304464775 | 0.0019407 | -1.715652771 | down |
| WP_011241601.1 | Grx4 family monothiol glutaredoxin | ZMO_RS08485*grxD | 274757886.3 | 369350468 | 238512941 | 153236546 | 130517901 | 102177442 | 2.286987215 | 0.0379958 | 1.193448301 | up |
| WP_011241217.1 | DUF3576 domain-containing protein | ZMO_RS06430*- | 187990233.1 | 283318242 | 269083741 | 381260057 | 363137105 | 371760576 | 0.663340127 | 0.0480449 | -0.592179294 | down |
| WP_011241572.1 | SoxR reducing system RseC family protein | ZMO_RS08285*- | 6551140.581 | 3279244.73 | 11723581.2 | 34255060 | 29491670 | 21782215 | 0.252007864 | 0.0114211 | -1.988459342 | down |
| WP_017466460.1 | sel1 repeat family protein | ZMO_RS00555*- | 1806854.756 | 3110409.09 | 4185926.73 | 8898335 | 7867546.7 | 10210170 | 0.337454526 | 0.0035082 | -1.56723499 | down |
| WP_011240048.1 | rRNA maturation RNase YbeY | ZMO_RS00305*ybeY | 19156967.59 | 17515878.1 | 15381432.7 | 10348723 | 12524472 | 9494348.7 | 1.608224533 | 0.0106611 | 0.685468843 | up |
| WP_011241117.1 | lipopolysaccharide biosynthesis protein | ZMO_RS05895*- | 3329809.523 | 20744663.6 | 19223280.9 | 42601596 | 33426894 | 45650298 | 0.355836498 | 0.0227176 | -1.4907136 | down |
| WP_011240019.1 | cysteine synthase A | ZMO_RS03345*- | 82122484.53 | 73092128.9 | 68416983.7 | 50679983 | 44168271 | 50961464 | 1.533722171 | 0.0099337 | 0.617037167 | up |
| WP_014501114.1 | septation ring formation regulator EzrA | ZMO_RS07215*- | 0 | 0 | 0 | 33466716 | 32043311 | 36418750 | #NAME? | 0 | #NAME? | down |
| WP_011240660.1 | Holliday junction resolvase RuvX | ZMO_RS03500*ruvX | 77995492.39 | 75552403.2 | 84644436 | 31976890 | 47844802 | 24984229 | 2.272699199 | 0.0128252 | 1.18440675 | up |
| WP_080502943.1 | redox-sensitive transcriptional activator SoxR | ZMO_RS09130*soxR | 0 | 0 | 0 | 2875207.2 | 2559977.1 | 2646599.7 | #NAME? | 0 | #NAME? | down |
| WP_011240627.1 | hypothetical protein | ZMO_RS03335*- | 23957037.59 | 21423015.1 | 39405466.5 | 61469499 | 63971433 | 73007739 | 0.427241556 | 0.0077839 | -1.226876115 | down |
| WP_011241697.1 | delta(1)-pyrroline-2-carboxylate reductase family protein | ZMO_RS09015*- | 37399564.86 | 25309893.9 | 33069487.7 | 50234637 | 51426551 | 51609156 | 0.624902017 | 0.0308351 | -0.678298097 | down |
| WP_011241333.1 | DUF2474 family protein | ZMO_RS07035*- | 24946487.03 | 24474262.4 | 24782942.4 | 56386618 | 47116717 | 47124117 | 0.492630597 | 0.0142427 | -1.02142186 | down |
| WP_011241645.1 | hypothetical protein | ZMO_RS08720*- | 0 | 0 | 0 | 12870285 | 11180666 | 13894326 | #NAME? | 0 | #NAME? | down |
| WP_011240085.1 | Rrf2 family transcriptional regulator | ZMO_RS00490*- | 37249125.29 | 49406519.4 | 67973889.2 | 103103498 | 110858615 | 91906993 | 0.505541524 | 0.0134944 | -0.984098498 | down |
| WP_011240405.1 | hypothetical protein | ZMO_RS02170*- | 1940003.079 | 4464189.25 | 6358309.32 | 13566338 | 8853985.4 | 17413387 | 0.320394498 | 0.0479485 | -1.642078721 | down |
| WP_012817615.1 | epoxyqueuosine reductase QueH | ZMO_RS08540*- | 2759398.359 | 3025498.71 | 5581962.05 | 14122304 | 15145669 | 13480979 | 0.265897956 | 0.0017965 | -1.911055405 | down |
| WP_011241266.1 | DedA family protein | ZMO_RS06690*- | 786352.2083 | 0 | 2486089.98 | 22227643 | 15096693 | 23766561 | 0.080350158 | 0.0136424 | -3.637555333 | down |
| WP_011241544.1 | RnfABCDGE type electron transport complex subunit D | ZMO_RS08140*- | 17864951.11 | 47487573.3 | 38912220.8 | 90169769 | 68084690 | 88429293 | 0.422665637 | 0.0150179 | -1.242411269 | down |
| WP_011241474.1 | hypothetical protein | ZMO_RS07785*- | 5949186.773 | 0 | 7054060.96 | 13007502 | 9344078.6 | 10517018 | 0.59341962 | 0.0392011 | -0.752875468 | down |
| WP_011240175.1 | lipopolysaccharide biosynthesis protein | ZMO_RS00980*- | 11553704.75 | 36820769.8 | 39301804.9 | 105255407 | 74024062 | 101537876 | 0.312218176 | 0.0085212 | -1.679373567 | down |
| WP_011241293.1 | hypothetical protein | ZMO_RS06820*- | 3726025.653 | 978263.394 | 5133242.8 | 10466605 | 9979974 | 9747456.4 | 0.32581044 | 0.0278491 | -1.617895263 | down |
| WP_011240896.1 | N-acetyltransferase | ZMO_RS04730*- | 156515892.9 | 141698340 | 144897773 | 92630164 | 76731714 | 94280820 | 1.680729292 | 0.0013753 | 0.749087375 | up |
| WP_011240069.1 | MFS transporter | ZMO_RS00410*- | 3521439.771 | 1000791.15 | 0 | 20818652 | 11430026 | 15387630 | 0.142398656 | 0.0249736 | -2.81199256 | down |
| WP_011241251.1 | hypothetical protein | ZMO_RS06605*- | 29271214.85 | 34941323.7 | 34505940.7 | 19226461 | 23160678 | 23344242 | 1.501847041 | 0.0102235 | 0.586737886 | up |
| WP_011241456.1 | holo-ACP synthase | ZMO_RS07690*- | 30490874.88 | 17557084.3 | 23986710.9 | 35821306 | 46755568 | 39917604 | 0.588064635 | 0.0276804 | -0.765953361 | down |
| WP_011240990.1 | ComF family protein | ZMO_RS05220*- | 0 | 0 | 0 | 1701368 | 4116670.9 | 2782226.9 | #NAME? | 0 | #NAME? | down |
| WP_011241312.1 | TetR/AcrR family transcriptional regulator | ZMO_RS06920*- | 30936704.83 | 25160019.6 | 21829677 | 11864785 | 14636986 | 13198572 | 1.962864698 | 0.032193 | 0.97296073 | up |
| WP_011240684.1 | recombination protein RecR | ZMO_RS03620*recR | 1502285.117 | 1132259.13 | 1966283.58 | 5453752.2 | 4604384.3 | 6024020.8 | 0.286082751 | 0.0031077 | -1.805495578 | down |
| WP_011241311.1 | J domain-containing protein | ZMO_RS06915*- | 2115794.686 | 9495148.01 | 2932900.44 | 16974818 | 18913348 | 20864891 | 0.256265373 | 0.0136476 | -1.964289545 | down |
| WP_011241245.1 | ABC transporter permease | ZMO_RS06575*- | 2803151.893 | 13708063.2 | 21782197.6 | 63287215 | 66227358 | 67542727 | 0.194326284 | 0.0080466 | -2.363447047 | down |
| WP_011240876.1 | ABC transporter permease subunit | ZMO_RS04615*- | 1527723.969 | 12669291 | 12595246.6 | 28274754 | 27503794 | 29859595 | 0.312854303 | 0.0302244 | -1.676437148 | down |
| WP_014500758.1 | dihydrofolate reductase | ZMO_RS01375*- | 19326869.65 | 21818901.4 | 21219085.9 | 14602759 | 11827661 | 9589128.1 | 1.731416973 | 0.0125835 | 0.791953207 | up |
| WP_011240358.1 | MBOAT family protein | ZMO_RS01925*- | 9938752.736 | 36895358 | 35580106.5 | 58032008 | 52546940 | 69812817 | 0.456862414 | 0.0441912 | -1.130168338 | down |
| WP_017466233.1 | DNA replication and repair protein RecF | ZMO_RS07115*- | 1793880.298 | 1523945.89 | 2320153.61 | 0 | 0 | 0 | Inf | 0 | Inf | up |
| WP_011240192.1 | 50S ribosomal protein L33 | ZMO_RS01075*rpmG | 1004560899 | 658155240 | 871863434 | 2.261E+09 | 2.246E+09 | 2.058E+09 | 0.386064865 | 0.0008157 | -1.373084831 | down |
| WP_011240253.1 | signal peptidase II | ZMO_RS01390*lspA | 0 | 0 | 0 | 17118668 | 10603990 | 17366727 | #NAME? | 0 | #NAME? | down |
| WP_011240869.1 | alpha/beta hydrolase | ZMO_RS04580*- | 111701813.4 | 148976299 | 142168300 | 97486236 | 87623158 | 70124111 | 1.578344556 | 0.0293534 | 0.658412184 | up |
| WP_011240936.1 | tRNA (adenosine(37)-N6)-threonylcarbamoyltransferase complex ATPase subunit type 1 TsaE | ZMO_RS04930*tsaE | 39944448.08 | 37008953 | 47049371.1 | 32150167 | 20063589 | 19191770 | 1.736599091 | 0.0315313 | 0.796264734 | up |
| WP_011241061.1 | cytochrome c biosynthesis protein | ZMO_RS05610*- | 3827400.211 | 7178702.79 | 7596461.2 | 19561287 | 18795416 | 19799174 | 0.319874191 | 0.0055681 | -1.644423501 | down |
| WP_011240333.1 | YdcF family protein | ZMO_RS01800*- | 0 | 0 | 0 | 6834822.7 | 2917163 | 1631346.7 | #NAME? | 0 | #NAME? | down |
| WP_011240410.1 | aminoacyl-tRNA hydrolase | ZMO_RS02195*- | 4078202.272 | 2568308.46 | 7864637.69 | 16235350 | 18620506 | 16148330 | 0.284508968 | 0.006402 | -1.813453968 | down |
| WP_011240441.1 | 50S ribosomal protein L30 | ZMO_RS02350*rpmD | 1495113451 | 1619857454 | 2188410119 | 2.685E+09 | 2.455E+09 | 3.41E+09 | 0.620296071 | 0.0433957 | -0.688971109 | down |
| WP_011240814.1 | cytochrome c | ZMO_RS04295*- | 39615383.4 | 44955980.3 | 56366606.5 | 32674238 | 24349017 | 23648560 | 1.747053422 | 0.0349166 | 0.804923724 | up |
| WP_011241589.1 | carbohydrate porin | ZMO_RS08390*- | 40037085.68 | 39599315 | 39410058.5 | 18222635 | 5710427.5 | 12435286 | 3.273353415 | 0.0165734 | 1.710769374 | up |
| WP_011240078.1 | hypothetical protein | ZMO_RS00455*- | 6727066.825 | 21707079.7 | 20786825.8 | 54640693 | 42065366 | 55557092 | 0.323262536 | 0.0064023 | -1.629221775 | down |
| WP_011240262.1 | molecular chaperone DnaJ | ZMO_RS01440*- | 1890939.081 | 2879422.03 | 2774381.37 | 3827302.3 | 3825521.4 | 4491650.6 | 0.621249 | 0.019908 | -0.686756471 | down |
| WP_080502949.1 | 50S ribosomal protein L34 | ZMO_RS09190*rpmH | 118329822.3 | 661487050 | 390430746 | 1.42E+09 | 1.808E+09 | 1.795E+09 | 0.232972581 | 0.0036018 | -2.101767924 | down |
| WP_011240873.1 | hypothetical protein | ZMO_RS04600*- | 1917864.541 | 1333759.33 | 4534670.86 | 51115047 | 55785301 | 57184034 | 0.047452991 | 0.0001211 | -4.397357164 | down |
| WP_011241581.1 | flavodoxin FldA | ZMO_RS08330*fldA | 12670649.02 | 12456163 | 16720587 | 0 | 2533312.8 | 3974789.3 | 4.286697653 | 0.0078073 | 2.099866665 | up |
| WP_011241179.1 | N-formylglutamate amidohydrolase | ZMO_RS06235*- | 1932007.75 | 1231974.9 | 686772.628 | 2643352.3 | 3263516.5 | 3170622.3 | 0.42420921 | 0.0227663 | -1.237152152 | down |
| WP_011240021.1 | DUF1491 family protein | ZMO_RS00165*- | 7881243.698 | 5857131.6 | 6490720.55 | 4582176.7 | 3042771.4 | 1871659.5 | 2.130139163 | 0.0249848 | 1.090947685 | up |
| WP_011240953.1 | Fis family transcriptional regulator | ZMO_RS05030*- | 6493518.794 | 5181285.78 | 8061791.99 | 11389503 | 11225678 | 10090732 | 0.603456517 | 0.0199306 | -0.728678276 | down |
| WP_011240081.1 | hypothetical protein | ZMO_RS00470*- | 9730630.161 | 3157973.17 | 9439500.16 | 33474510 | 31716836 | 32773814 | 0.227918819 | 0.0050708 | -2.133408047 | down |
| WP_011241460.1 | 30S ribosomal protein S21 | ZMO_RS07710*rpsU | 566960560.3 | 1331771927 | 942931548 | 1.912E+09 | 2.441E+09 | 2.489E+09 | 0.41529563 | 0.0105324 | -1.267789402 | down |
| WP_011240784.1 | cold shock domain-containing protein | ZMO_RS04140*- | 3684253.274 | 3734803.43 | 6449721.47 | 10056173 | 7185458.4 | 10142678 | 0.506449792 | 0.0279973 | -0.981508843 | down |
| WP_011240860.1 | DNA gyrase inhibitor YacG | ZMO_RS04530*yacG | 16071365.43 | 6749881.87 | 10037102.6 | 30427495 | 21953195 | 24650011 | 0.426561745 | 0.0166733 | -1.229173509 | down |
| WP_011240945.1 | undecaprenyl-diphosphate phosphatase | ZMO_RS04985*- | 0 | 0 | 0 | 3840966.2 | 2460039.2 | 1222529.6 | #NAME? | 0 | #NAME? | down |
| WP_011240237.1 | ribbon-helix-helix domain-containing protein | ZMO_RS01305*- | 0 | 2095892.32 | 1791031.7 | 3962146.4 | 3476160.3 | 3708439 | 0.523057238 | 0.0064061 | -0.934959265 | down |
| WP_011240822.1 | NCS2 family permease | ZMO_RS04335*- | 8777020.564 | 12287826.4 | 11403744.1 | 57660352 | 58014304 | 55998421 | 0.189130362 | 2.11E-05 | -2.402547112 | down |
| WP_011240300.1 | hypothetical protein | ZMO_RS01635*- | 3645186.871 | 0 | 5148819.64 | 8074022.7 | 13547535 | 14082836 | 0.369450604 | 0.0468457 | -1.43654661 | down |
| WP_014500556.1 | hypothetical protein | ZMO_RS09240*- | 2581648.489 | 5328139.55 | 2911517.9 | 8233213.1 | 10463559 | 14268520 | 0.328263615 | 0.0346041 | -1.607073248 | down |
| WP_011240493.1 | CvpA family protein | ZMO_RS02625*- | 1650185.062 | 3518943.56 | 0 | 10487629 | 8047900.4 | 9353790.6 | 0.278016566 | 0.0254516 | -1.846757243 | down |
| WP_011240468.1 | pyridoxal phosphate-dependent aminotransferase | ZMO_RS02495*- | 6344920.905 | 1967030.79 | 3484683.55 | 13421715 | 19659154 | 10494134 | 0.270720239 | 0.0414129 | -1.885125346 | down |
